# Integrative multi-omic analysis identifies new drivers and pathways in molecularly distinct subtypes of ALS

**DOI:** 10.1038/s41598-019-46355-w

**Published:** 2019-07-10

**Authors:** Giovanna Morello, Maria Guarnaccia, Antonio Gianmaria Spampinato, Salvatore Salomone, Velia D’Agata, Francesca Luisa Conforti, Eleonora Aronica, Sebastiano Cavallaro

**Affiliations:** 1 Institute of Neurological Sciences, Italian National Research Council, Catania, Italy; 2 Department of Biomedical and Biotechnological Sciences, Section of Pharmacology, University of Catania, Catania, Italy; 3 Department of Biomedical and Biotechnological Sciences, Section of Human Anatomy and Histology, University of Catania, Catania, Italy; 4 Department of NeuroPathology, Academic Medical Center, Amsterdam, The Netherlands; 5 Swammerdam Institute for Life Sciences, Center for Neuroscience, University of Amsterdam, Amsterdam, The Netherlands; 6 Department of Pharmacy, Health and Nutritional Sciences, University of Calabria, Rende, Cosenza Italy

**Keywords:** Classification and taxonomy, Genome informatics, Transcriptomics, Amyotrophic lateral sclerosis

## Abstract

Amyotrophic lateral sclerosis (ALS) is an incurable and fatal neurodegenerative disease. Increasing the chances of success for future clinical strategies requires more in-depth knowledge of the molecular basis underlying disease heterogeneity. We recently laid the foundation for a molecular taxonomy of ALS by whole-genome expression profiling of motor cortex from sporadic ALS (SALS) patients. Here, we analyzed copy number variants (CNVs) occurring in the same patients, by using a customized exon-centered comparative genomic hybridization array (aCGH) covering a large panel of ALS-related genes. A large number of novel and known disease-associated CNVs were detected in SALS samples, including several subgroup-specific loci, suggestive of a great divergence of two subgroups at the molecular level. Integrative analysis of copy number profiles with their associated transcriptomic data revealed subtype-specific genomic perturbations and candidate driver genes positively correlated with transcriptional signatures, suggesting a strong interaction between genomic and transcriptomic events in ALS pathogenesis. The functional analysis confirmed our previous pathway-based characterization of SALS subtypes and identified 24 potential candidates for genomic-based patient stratification. To our knowledge, this is the first comprehensive “omics” analysis of molecular events characterizing SALS pathology, providing a road map to facilitate genome-guided personalized diagnosis and treatments for this devastating disease.

## Introduction

Amyotrophic lateral sclerosis (ALS) is a neurodegenerative disorder, characterized by progressive loss and degeneration of motor neurons in both the motor cortex, brainstem and spinal cord, and is usually fatal due to respiratory failure within 3–5 years of onset^1^. The disease has an incidence of 2.6 per 100,000 individuals-years and prevalence rates of around 6–7/100,000 in Europe, making it the most common adult-onset motor neuron disease^2^. About 5–10% of ALS cases show a family history (FALS), while the remainder of cases are classified as sporadic (SALS), and are probably associated to a polygenic and multifactorial etiology^3–5^.

The remarkable advances in genome technologies over the last years have led to a huge progress in deciphering the genes and pathways involved in ALS pathogenesis. From the discovery of the first ALS-associated gene *SOD1*, several candidate-gene or genome-wide association studies (GWAS) have identified multiple single-nucleotide polymorphisms (SNPs) affecting potentially ALS-associated genes, including *C9orf72*, *TDP43*, *FUS*, *MATR3*, *UBQLN2*, *VCP* and *OPTN*^6–9^. In this context, a recent large-scale genome-wide association study identified a common missense variant and several rare loss-of-function (LOF) mutations within the microtubule motor protein-encoding gene, *KIF5A*, as candidate ALS risk factors, further supporting perturbations in cytoskeletal function play an important role in ALS and offering a potential target for drug development^10,11^.

In addition to the contribution of SNPs, which account for only a limited number of familial and sporadic ALS cases, evidence suggests that other genomic variants, such as copy-number variations (CNVs), that change gene dose rather than gene function, may exert a more pronounced effect on the onset and rate of disease progression^5,12,13^. In particular, the involvement of CNVs in ALS susceptibility has been clearly highlighted in two ALS genome-wide association studies, where multiple rare CNVs were shown to represent a more important risk factor for SALS than common CNVs^14,15^.

The complexity of its molecular architecture has completely transformed the way we think about ALS, leading us to reconsider the traditional classification and therapeutic systems. In fact, despite intensive research efforts, the precise causes of ALS remain unknown and there is no cure for this devastating disease. The absence of effective treatments can be due in part by the complex and heterogeneous clinical, biochemical and molecular features of ALS, which is also supported by clinical studies on Edaravone (MCI-186), a free radical scavenger recently approved by FDA for ALS treatment showing effectiveness only in specific sub-cohorts of patients^16^. Developing a robust molecular disease portrait that can explain the heterogeneity of ALS is thus fundamental to improve our understanding of the precise molecular mechanisms underlying disease pathogenesis and develop effective treatments for patients.

Our research group has recently characterized the transcriptional profiles of motor cortex samples from control and SALS patients, grouping these on the basis of their similarities measured over the most “hypervariable genes” (9.646 genes with a standard deviation >1.5). Unsupervised hierarchical clustering analysis allowed to discriminate controls from SALS patients and clearly distinguished two greatly divergent SALS subtypes, each associated with differentially expressed genes (DEGs) and biological pathways^5,17,18^. In particular, the most representative functional processes deregulated in SALS1 were annotated as involved in the regulation of chemotaxis, immunity, and cell adhesion and communication. Deregulated genes in SALS2, in turn, were selectively associated with cytoskeleton organization, regulation of transport and mitochondrial oxidative phosphorylation^5,17^. While these findings are consistent with previous evidence about the crucial role of these pathogenetic mechanisms in ALS^17,19–24^, they suggest for the first time the differential involvement of these mechanisms in specific subsets of ALS patients, offering a useful starting point for the further development of personalized diagnostics and targeted therapies.

While our work lays the foundation for a molecular taxonomy of ALS, very little information is so far available from the single-omic analysis, which makes difficult to discriminate genes critical to ALS pathogenesis (driver genes) from non-relevant genes (passenger genes). An integrated and comprehensive view of multiple genomic data types (such as genome and transcriptome) may provide a powerful potential for defining disease subgroups and their molecular drivers, allowing for an overall understanding of the complex molecular networks that drive ALS pathogenesis at yet another level of systemic complexity.

In this study, we applied the customized exon-centric comparative genomic hybridization array (aCGH) *NeuroArray* platform, designed to target genes associated with ALS as well as genes associated with other neurological disorders^25^, to analyze copy number variants (CNVs) in 40 motor cortex samples of control (10) and SALS (30) patients, clinically and transcriptomically characterized in our previous work^17,25^. Next, we provided the first comprehensive integrative analysis of genomic aberrations with expression data derived from the same patients to identify specific chromosomal regions and genes with concordant alterations in DNA and RNA profiles that may represent promising key molecular candidates for SALS. Finally, functional pathway and network analyses were carried out to gain further insights into the molecular complexity of ALS and reveal novel and yet unrecognized biomarkers and therapeutic targets, potentially useful for the development of personalized medicine in ALS.

## Results

### Transcriptomically distinct SALS patient subgroups show specific copy number alterations

**Figure 1:**
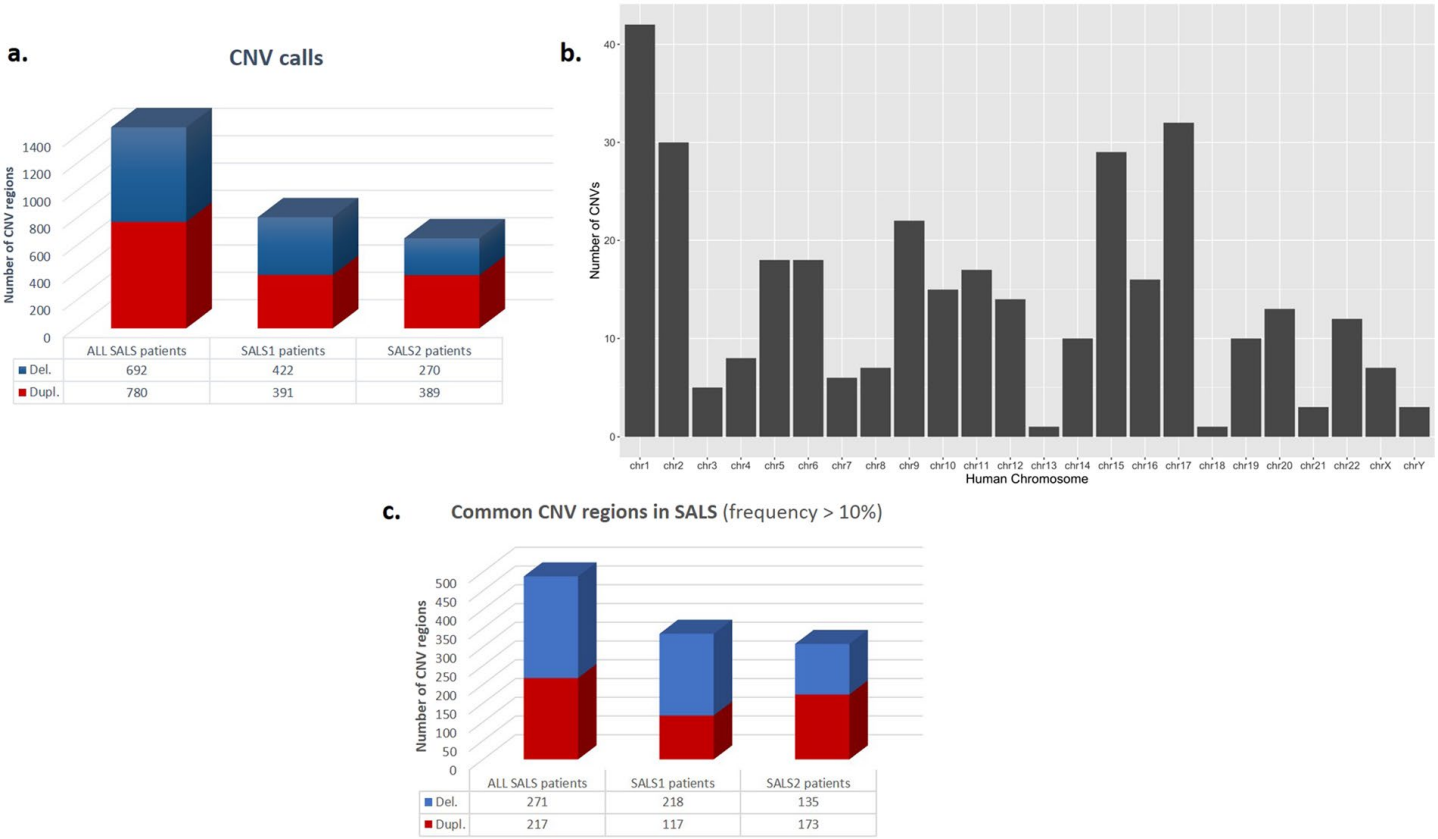
Significant copy number regions in SALS patient subgroups. (**a**) Graphical overview of CNV regions detected in SALS patients by *NeuroArray* platform. The bars represents the number of regions that may be involved in CNV detected in SALS patients (ADM-2 aberration filter: threshold = 6; Log2 ratio ≥ 0.5 and 3 consecutive interval probes), both with and without the assignment into the hierarchically determined two sets (SALS1 and SALS2). The blue bar represents the number of deleted regions and the red bar represents the number of amplified regions. (**b**) Chromosome distribution of CNVs detected with high-resolution custom exon-centered *NeuroArray* aCGH from SALS patients. The horizontal axis represents different chromosomes and the vertical axis represents the number of regions of each chromosome that may be involved in CNV. (**c**) Graphical overview of recurrent gains or losses (occurred in at least 10% of the SALS patients), both with and without the assignment into the hierarchically determined two sets (SALS1 and SALS2). The blue bar represents the number of deleted regions and the red bar represents the number of amplified regions that occurred at a high frequency (≥10%) in our cohort of SALS patients.

The customized exon-centric *NeuroArray* aCGH platform was used to identify DNA copy number alterations in 30 SALS patients and 10 controls. A total of 1472 CNVs were detected in SALS, including 780 losses and 692 gains (Fig. 1a). The chromosomal distribution of all CNVs across the 30 SALS genomes tested is plotted in Fig. 1b. To reduce individual heterogeneities and identify ALS-related significant CNVs, we focused on those that occurred in at least 10% SALS samples. Accordingly, a total of 488 significant CNVs ranging in size from 7 bp to 5.9 Mb were identified in SALS patients, including 271 losses and 217 gains **(**Fig. 1c, Supplementary Table 1). Recurrent CNVs were dispersed in the chromosome 1 to 22, with the most frequent amplifications (76.7%) found in chromosome 14, followed by amplifications in chromosome 17 (70%) (Supplementary Table 1). The most common linkage rate in SALS mapped to chromosome 20 with a frequency of 80%, followed by chromosome 1 with a frequency of 76.6% (Supplementary Table 1).

To identify subgroup-specific genomic signatures, we analyzed CNV events taking into account the previously characterized transcriptome-based stratification of SALS patients in the two subgroups, SALS1 and SALS2. Overall, 813 aberrant regions were associated with SALS1 and 659 with SALS2 patients (Fig. 1a). Among these, 335 CNVs (218 losses and 117 gains) were detected as frequently altered in SALS1 patients, while 308 (135 losses and 173 gains) were frequently associated with the SALS2 subgroup (Fig. 1c, Table 1 and Supplementary Tables 2–3). Interestingly, a large number of these recurrent amplifications and deletions were detected exclusively in SALS patients (absent in the control samples) (Table 2).

**Table 1:** The top most frequent copy number gain and loss in both SALS patient subgroups.

	SALS1 patients						SALS2 patients					
	Chr.	Start	Stop	Aberration Size (bps)	Frequency (%)	NeuroArray genes	Chr.	Start	Stop	Aberration Size (bps)	Frequency (%)	NeuroArray genes
DUPLICATIONS	14	31552632	31552690	59	76.47	AP4S1	14	31552632	31552690	59	76.92	AP4S1
	17	17716576	17720711	4136	70.59	SREBF1	17	17716576	17720711	4136	69.23	SREBF1
	X	122318451	122336599	18149	70.59	GRIA3	22	24376158	24384300	8143	69.23	GSTT1
	X	122318291	122318451	161	64.71	GRIA3	17	17720711	17726812	6102	61.54	SREBF1
	7	100493729	100493862	134	58.82	ACHE	9	129272014	129458220	186207	53.85	LMX1B
	X	122318031	122318291	261	58.82	GRIA3	9	131388073	131394672	6600	53.85	—
	X	122336599	122459975	123377	58.82	GRIA3	17	17715816	17716576	761	53.85	SREBF1
	17	17720711	17726812	6102	52.94	SREBF1	1	55331123	55527185	196063	46.15	DHCR24, PCSK9
	7	100488043	100490289	2247	47.06	ACHE	2	127807997	128439169	631173	46.15	BIN1, LIMS2
	7	100493373	100493729	357	47.06	ACHE	2	241657356	241728764	71409	46.15	KIF1A
	9	131388073	131394672	6600	47.06	—	5	176853852	176869527	15676	46.15	GRK6
DELETIONS	1	47716828	47775972	59145	88.24	STIL	3	155547586	155560259	12674	69.23	SLC33A1
	20	33986975	35569474	1582500	88.24	UQCC, NFS1, PHF20, EPB41L1, DLGAP4, NDRG3, TLDC2, SAMHD1	20	33986975	35575306	1588332	69.23	UQCC, NFS1, PHF20, EPB41L1, DLGAP4, NDRG3, TLDC2, SAMHD1
	20	35569474	35575306	5833	82.35	SAMHD1	1	47767175	47770585	3411	61.54	STIL
	1	47435653	47716828	281176	76.47	STIL	18	9117815	9134199	16385	61.54	NDUFV2
	2	32339724	32429834	90111	76.47	**SPAST**, SLC30A6	3	155560259	155560361	103	53.85	SLC33A1
	3	155551254	155560202	8949	76.47	SLC33A1	12	111956174	111993684	37511	53.85	**ATXN2**
	9	128001127	128001733	607	76.47	HSPA5	1	47435653	47767175	331523	46.15	STIL
	1	47775972	47776133	162	70.59	STIL	1	47770585	47770755	171	46.15	STIL
	2	32323849	32339724	15876	70.59	**SPAST**, SLC30A6	2	32314495	32409410	94916	46.15	**SPAST**, SLC30A6
	2	32429834	32432100	2267	70.59	**SPAST**, SLC30A6	10	70432579	70441196	8618	46.15	TET1
	3	155547586	155551254	3669	70.59	SLC33A1	14	92527804	92562372	34569	46.15	ATXN3

The table lists the gains and losses that occurred in at least 10% of the two previously characterized transcriptome-based SALS subgroups. The chromosomal regions, including the start and end positions, aberration size, frequency in SALS patients and CNV embedded NeuroArray genes are listed. Chromosomal positions are referred to the human reference sequence hg19 assembly. Genes previously identified as potential risk factors in ALS are in bold.

**Table 2:** Chromosomal distribution of the most frequent CNVs exclusively detected in SALS patients.

Chr.	Start	Stop	Lenght (bps)	SALS patients	*NeuroArray*-related genes
**Duplications**					
1	19229182	19826022	596841	6	ALDH4A1, UBR4
1	22216964	22222489	5526	5	CLCNKA, CLCNKB, **FBXO42**, RCC2, IGSF21, HTR6, VWA5B1, CDA, PINK1, DDOST, EIF4G3, ECE1, USP48, LDLRAD2, HSPG2, APL
1	22222489	22222904	416	4	CLCNKA, CLCNKB, **FBXO42**, RCC2, IGSF21, HTR6, VWA5B1, CDA, PINK1, DDOST, EIF4G3, ECE1, USP48, LDLRAD2, HSPG2, APL, CDC42
1	22222904	22379326	156423	3	CLCNKA, CLCNKB, **FBXO42**, RCC2, IGSF21, HTR6, VWA5B1, CDA, PINK1, DDOST, EIF4G3, ECE1, USP48, LDLRAD2, HSPG2, APL
1	110280992	110467801	186810	3	GSTM3, CSF1
1	165377539	165378926	1388	5	LMX1A, RXRG
1	165378926	165406335	27410	3	LMX1A, RXRG
2	127805579	127806098	520	3	BIN1, LIMS2
2	152954807	152955141	335	3	CACNB4
5	37824072	37834861	10790	4	GDNF
9	135810367	136390729	580363	3	TSC1, RXRA, EDF1, TRAF2, ABCA2, MAN1B1, GRIN1
11	117263667	117265845	2179	3	CEP164
17	8790922	8791519	598	3	PIK3R5
17	8791519	8794231	2713	4	PIK3R5
17	34198604	34415754	217151	3 (only SALS1)	CCL5, CCL3
17	56349278	56350271	994	3	MPO
19	50364607	50364748	142	5	PNKP
22	18907140	18918487	11348	3 (only SALS1)	PRODH
X	62886007	63005325	119319	3	ARHGEF9
**Chr**.	**Start**	**Stop**	**Size**	**Ratio SALS/Control**	***NeuroArray*** **-related genes**
**Deletions**					
1	173797400	173826691	29292	5	DARS2
1	173827623	174553313	725691	3	ZBTB37, RABGAP1L
1	207791343	207791566	224	3	CR1
1	207793150	207795258	2109	6	CR1
1	207795258	207815117	19860	7	CR1
3	155560361	155572231	11871	4	SLC33A1
3	155572231	156645173	1072943	3	SLC33A1, KCNAB1, TIPARP, LEKR1
3	156645399	156660486	15088	3	LEKR1
4	1810306	3434075	1623770	4	FGFR3, POLN, ZFYVE28, FAM193A, NOP14, RGS12
6	31625489	31777500	152012	3	HSPA1L
6	31777500	31783348	5849	4	HSPA1L, HSPA1A
6	31783348	31797880	14533	6	HSPA1A, HSPA1B
6	74354308	74530628	176321	3 (only SALS2)	SLC17A5
8	94767910	94777676	9767	4	TMEM67
8	94805417	94830376	24960	4	TMEM67
9	39140211	41979303	2839093	3	CNTNAP3, KGFLP2
9	125947382	126690362	742981	5	STRBP, DENND2A
10	101934013	101938026	4014	3	ERLIN1
11	108124607	108155047	30441	3	ATM
15	63579805	63673002	93198	3	APH1B, CA12
15	63673002	64226370	553369	4	HERC1, DAPK2
16	70551635	70553577	1943	3	COG4
21	44496313	44496400	88	3	CBS
22	42373034	42373060	27	4	SEPT3
X	40460110	41599792	1139683	3	ATP6AP2, MED14, DDX3X, CASK
X	119673121	119676960	3840	3	CUL4B

The table lists the duplications and deletions that occurred in at least 10% of SALS patients and absent in the control samples. The chromosomal regions, including the start and end positions, number of SALS patients and CNV embedded *NeuroArray* genes are listed. Chromosomal positions are referred to the human reference sequence hg19 assembly. Genes previously identified as potential risk factors in ALS are in bold.

To investigate the reliability of our results and further confirm the potential functional implications of the detected CNVs in ALS pathogenesis, we interrogated our data for overlap with genomic aberrations previously associated to ALS cases available in publicly available databases (i.e., CNVD) and published PubMed literature. Highly similar genomic altered patterns were observed, supporting the functional importance of these regions in disease etiopathogenesis (Table 3).

**Table 3:** Characteristics of the most frequent CNV regions detected in SALS patients and previously associated to ALS from different database and/or published literature.

Chr.	Start	Stop	CNV type	Overlapped Genes*	SALS (n = 30)	Control (n = 10)	Previously reported aberrations	Reference/Database
5	70320678	71554990	Loss	**SMN1**, **SMN2**, **NAIP**, BDP1, MRPS27	7 (6 SALS1 and 1 SALS2)	2	Loss n = 27/167 ALS n = 6/167 controls	Corcia P. *et al*., *Annals of neurology* 2002
8	144635580	145024441	Gain	**GSDMD**, **EEF1D**, PLEC1	5 (2 SALS1 and 3 SALS2)	1	Gain n = 12/781 ALS n = 0/621 controls	Wain L.V. *et al*., *PlosOne*, 2009; Cronin S. *et al*., *Hum Mol Gen*, 2008
11	424565	792284	Gain	ANO9, DRD4, **DEAF1**, SLC25A22, **OR4A5**, **OR4C12**	3 (SALS2)	1	Gain n = 11/575 ALS n = 0/621 controls	Wain L.V. *et al*., *PlosOne*, 2009
11	4056596	7324468	Loss	STIM1, HBB, TRIM5, CCKBR, APBB1, RRP8, TPP1, **SYT9**	4 (1 SALS1 and 3 SALS2)	1	Loss n = 5/575 ALS n = 0/621 controls	Wain L.V. *et al*., *PlosOne*, 2009
15	20575646	23060821	Loss and Gain	**NIPA1**, NIPA2	10 (6 SALS1 and 4 SALS2)	3	Loss and Gain n = 15/4434 ALS n = 8/14618 controls	CNVD; Blauw HM *et al*., *Hum Mol Gen*, 2010
15	27017550	27018935	Loss and Gain	GABRB3	3 (1 SALS1 and 2 SALS2)	1	Loss and Gain n = 15/4434 ALS n = 8/14618 controls	CNVD; Blauw HM *et al*., *Hum Mol Gen*, 2010
15	41535920	42703427	Loss and Gain	CHP1, NUSAP1, NDUFAF1, MGA, PLA2G4E, CAPN3	5 (2 SALS1 and 3 SALS2)	3	Loss and Gain n = 15/4434 ALS n = 8/14618 controls	CNVD; Blauw HM *et al*., *Hum Mol Gen*, 2010
15	63579805	64226370	Loss	APH1B, CA12, HERC1, DAPK2, **DPP8**, **PTPLAD1**, **C15orf44**, **SLC24A1**	4 (3 SALS1 and 1 SALS2)	0	Loss n = 1/1875 ALS n = 0/8731 controls	CNVD; Blauw HM *et al*., *Hum Mol Gen*, 2010
17	2580007	4605227	Loss	**OR1D5**, **OR1D4**, **OR1D2**, **OR1G1**, **OR1A2**, **OR1A1**, **SPNS2**, **ALOX15**, **PELP1**, **ARRB2**, **MED11**, **CXCL16**, **ZMYND15**, PAFAH1B1, RAP1GAP2, ITGAE	9 (5 SALS1 and 4 SALS2)	4	Loss and Gain n = 1/1875 ALS n = 0/8731 controls	CNVD; Blauw HM *et al*., *Hum Mol Gen*, 2010
17	75277604	78092622	Gain	SEPT9, TNRC6C, DNAH17, CYTH1, **TIMP2**, C1QTNF1-AS1, C1QTNF1, RBFOX3, TBC1D16, **CHMP6**, **AATK**, **BAIAP2**	7 (4 SALS1 and 4 SALS2)	2	Gain n = 1/12 ALS n = 0/24 controls	CNVD, Pamphlett R. *et al*., *Journal of Neuroscience*, 2011
X	153127628	153602907	Gain	L1CAM, MECP2, FLNA	9 (4 SALS1 and 5 SALS2)	2	Loss	Schoichet S.A. *et al*., *Amy Lat Scl*, 2009

The table shows the most frequent CNV loci and relative *NeuroArray* genes that partially or completely overlap with genomic aberrations previously associated to ALS cases and reported in publicly available databases (i.e., CNVD) and/or published PubMed literature. The chromosomal regions, including the start and end positions, aberration type and CNV embedded *NeuroArray* genes are listed. In addition, the number of controls and ALS cases from both our experiment and previous scientific reports of these CNV loci was also shown. Chromosomal positions are referred to the human reference sequence hg19 assembly. Genes that may be reasonable ALS candidates are in bold. Chr: Chromosome.

### Identifying CNV signature genes in SALS

**Figure 2:**
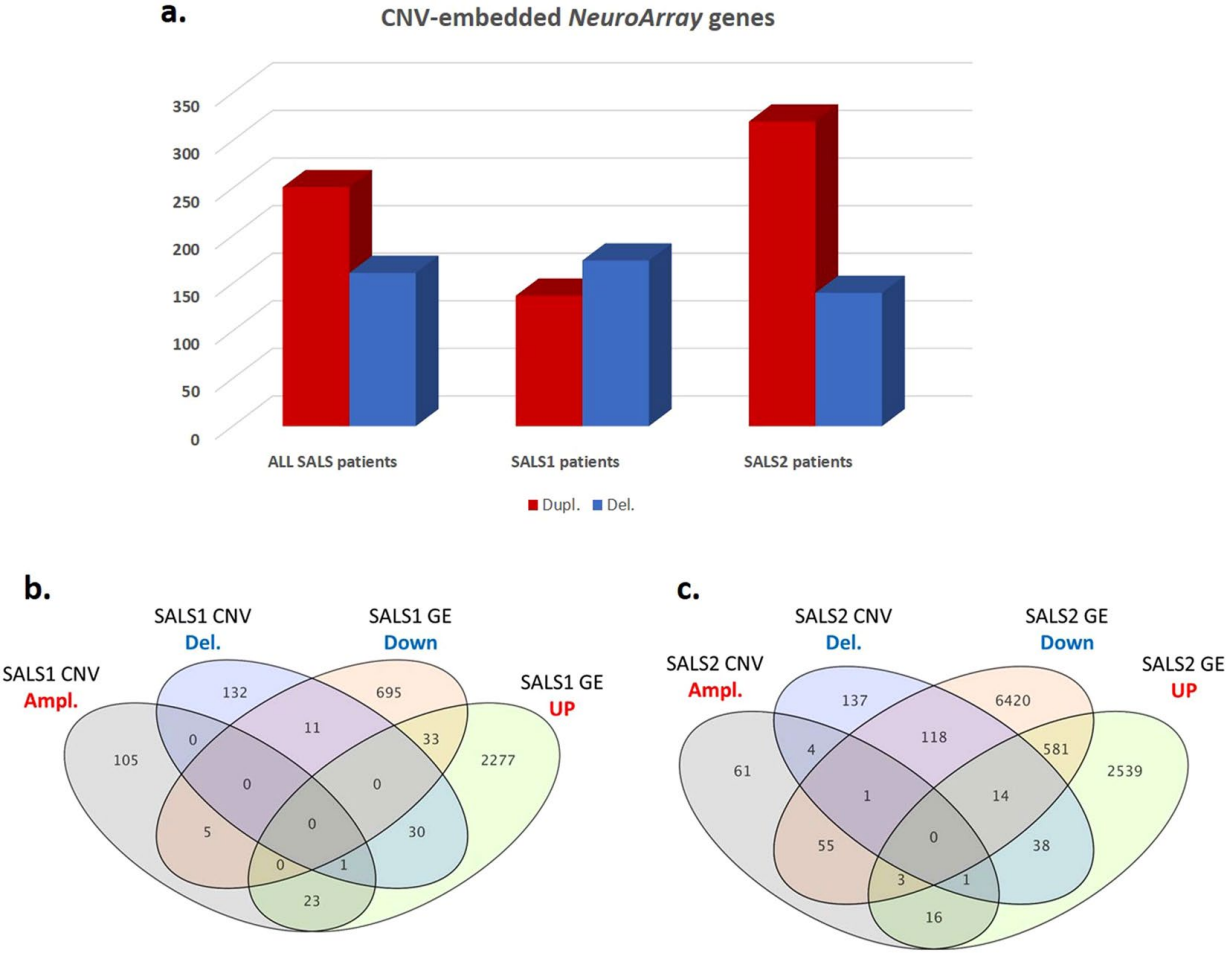
Integrative analysis of DNA copy number and expression variation in SALS patients reveals a good number of overlapping ALS candidate genes. (**a**) Graphical overview of the most frequent (≥10%) CNV-embedded genes detected in SALS patients, both with and without the assignment into the hierarchically determined two sets (SALS1 and SALS2). (**b**,**c**) Venn diagrams compare the number of protein-coding genes obtained from CNV analyses with the genes found to be differentially expressed in SALS1 (**b**) and SALS2 (**c**) patients.

To identify ALS driver genes from aberrant regions, the recurrent CNVs in SALS were annotated and filtered out for genes previously linked to ALS and other neurological diseases as causative and/or susceptibility factors and included in the *NeuroArray* design. A total of 406 significant CNV genes were obtained, including 251 duplications and 161 deletions (Fig. 2a, Supplementary Table 4). Among these, 36 were previously identified as ALS-linked genes (Supplementary Table 4). The same analysis was also performed on the most frequent subgroup-specific CNV regions, revealing 310 genes as the most significantly altered CNV-genes in SALS1 (137 duplicated and 174 deleted) and 454 genes in SALS2 patients (320 duplicated and 140 deleted). Among these, 28/310 SALS1 and 39/454 SALS2 CNV genes were already associated to ALS (Fig. 2a, Supplementary Tables 5 and 6).

### Integrated analysis of CNVs and gene expression profiling identify candidate ALS-driver genes

To determine whether genomic aberrations contribute to global gene expression patterns in SALS, the identified CNV genes were checked for overlap with the DEGs previously detected in the same patient cohort^17^. We identified 70 overlapping CNV genes (29 duplications and 42 deletions) that were also differentially expressed in SALS1 patients and 246 CNV-driven DEGs in SALS2 patients (173 duplicated and 76 deleted) (Fig. 2b,c, Supplementary Tables 7 and 8). Among these, 35 CNV-driven genes (50%) in SALS1 and 112 CNV-driven genes (45%) in SALS2 showed a positive association between gene expression and DNA copy number changes, including 77 up-regulated genes (24 in SALS1 and 53 in SALS2) and 70 down-regulated genes (11 in SALS1 and 59 in SALS2) (Fig. 2b,c, Supplementary Tables 7 and 8). Interestingly, several CNV-driven genes were SALS-patient specific (not detected in 10 controls) and most of them were previously linked to ALS.

To demonstrate that the correlation found in our work is meaningful, we also performed a “control experiment” in which we evaluated the overlap between the genomic and transcriptomic data between two random groups within the total SALS samples (different from SALS1 and SALS2). We observed a very low overlap between CNV genes and differentially expressed genes in these randomized disease-related subgroups, confirming the appropriateness/accuracy of our analysis (Supplementary Fig. 2 and Supplementary Table 14).

### Computational systems biology analysis identified distinct drivers and pathways in SALS molecular subtypes

To gain further insights into the biological role of identified CNV-driven DEGs in SALS, functional annotation and pathway enrichment analyses were performed by using specialized bioinformatics tools and databases (i.e., Enrichr, IPA, Metacore).

**Figure 3:**
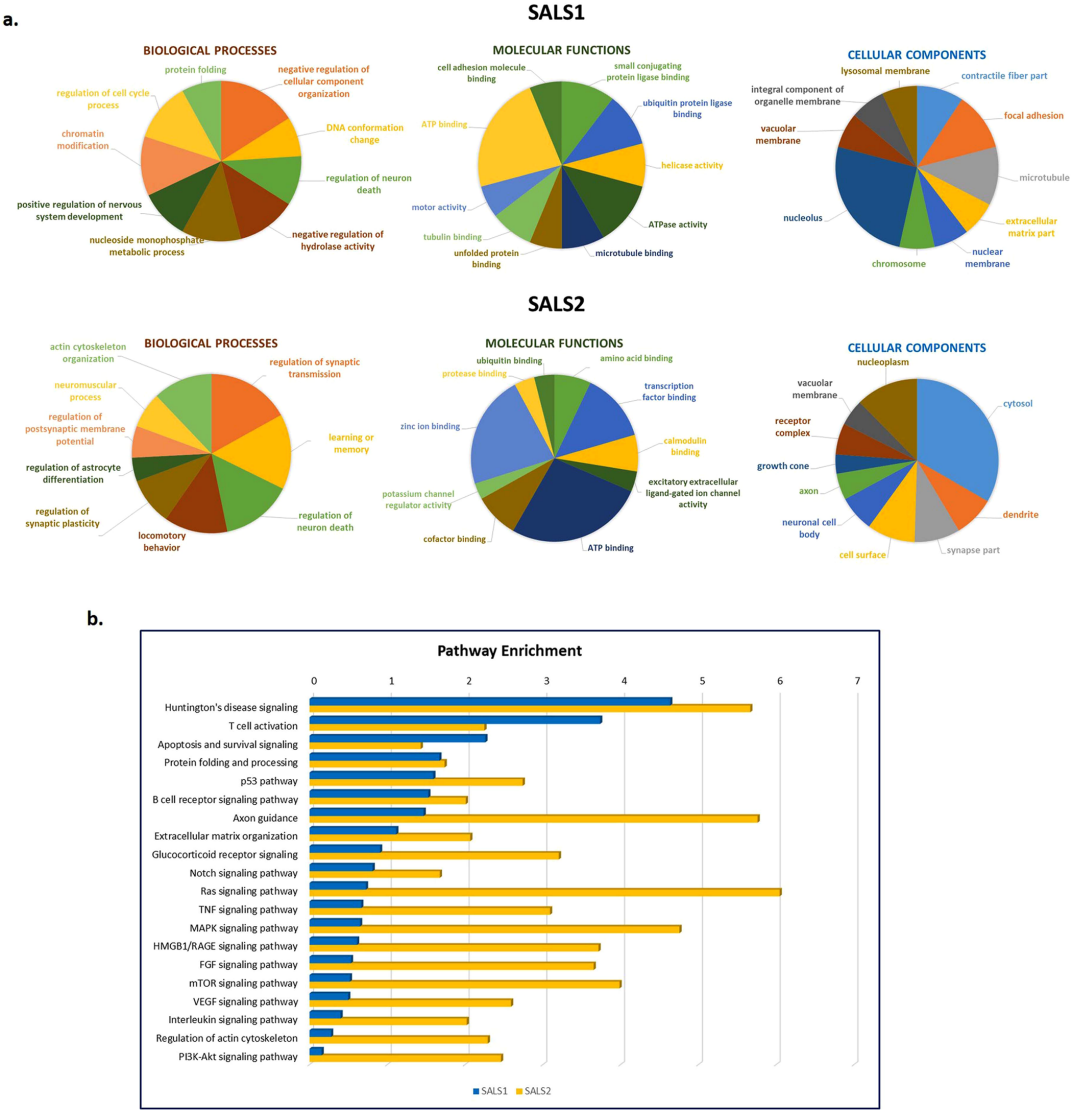
Functional enrichment analysis for GO and pathway map ontologies revealed significant biological processes associated with the candidate CNV-driven genes in SALS. (**a**) Pie charts represent the top 10 enriched (P < 0.05) GO terms for the 70 CNV-encompassed DEGs in SALS1 and SALS2 patients. The GO terms were subdivided into three GO categories: biological processes, molecular functions and cellular components. Enrichment analyses were performed using the Enrichment Analysis tool in Enrichr. For each category, GO terms or biological features represented in CNV-driven differently expressed genes are indicated. (**b**) Representation of the top 20 most significantly enriched (P value < 0.05) canonical pathway maps associated with the candidate CNV-driven genes in SALS1 and SALS2 patients. A histogram of statistical significance (−log P value) is shown: the list is arranged in descending order with the most significant pathways at the top. The analysis was performed using the MetaCore™ pathway analysis suite.

According to GO analysis, the CNV-driven DEGs in SALS1 were significantly enriched in biological processes such as *regulation of cellular component organization* (GO:0051129, P value = 0.0002), *DNA conformation change* (GO:0071103, P value = 0.0004) and *regulation of neuron death* (GO:1901214, P value = 0.0012), whereas *regulation of synaptic transmission* (GO:0050804, P value = 3.45E^−12^) and *learning and memory* (GO:0007611, P value = 5.26E^−12^) were the most enriched in SALS2 (Fig. 3a, Supplementary Table 9). On the basis of molecular function, the CNV-driven DEGs in SALS1 prominently accumulated in *small conjugating protein ligase binding* (GO:0044389, P value = 0.0004) and *ubiquitin protein ligase binding* (GO:0031625, P value = 0.0004), while the CNV-driven DEGs in SALS2 were enriched in *amino acid binding* (GO:0016597, P value = 7.00126E^−06^) and *transcription factor binding* (GO:0008134, P value = 6.6232E-05) (Fig. 3a, Supplementary Table 9).

**Figure 4:**
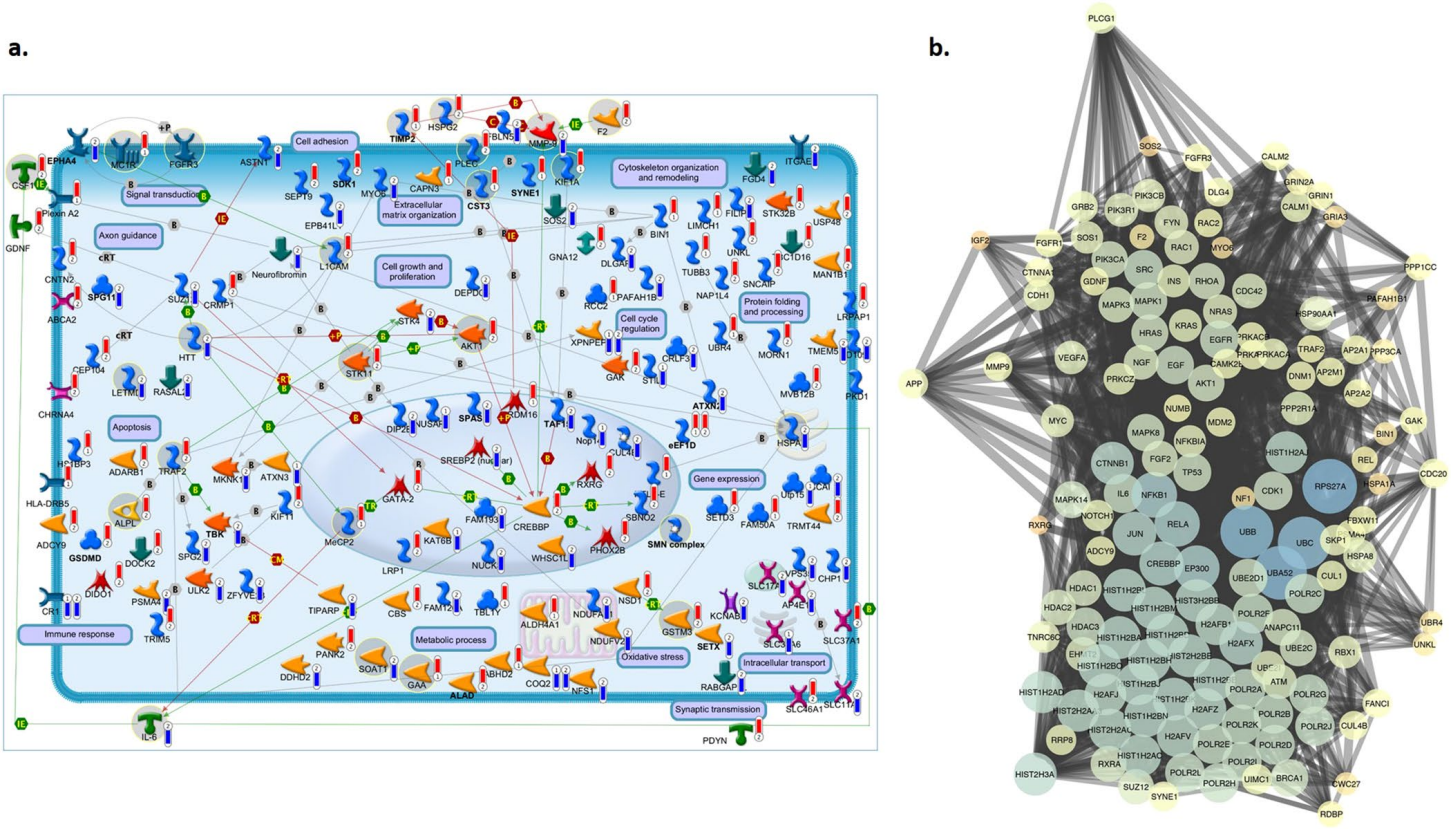
Definition of SALS subtype-specific genomic signature using pathway and network analyses. (**a**) A representative illustration showing the functional correlation between SALS associated CNV-driven genes and their biological processes. Interaction map represents the most promising candidate genes showing a positive correlation between gene expression and underlying genomic changes, grouped on the basis of the main biological processes associated with them. The map was created using the MetaCore Pathway Map Creator tool (GeneGo). Gene expression and CNV values are presented on the map as ‘thermometer-like’ figures with SALS1 patients data represented as thermometer #1 and SALS2 patients as #2. Genes associated with overexpression and CNV gain regions are labeled with red dots while genes associated with downregulated expression and homozygous or heterozygous deleted CNVs are labeled with blue dots. A detailed legend for the network objects is shown in the Supplementary Fig. 1. (**b**) Functional network of known and predicted interactions of the most promising candidate CNV-driven genes. The network was produced by the Search Tool for the Retrieval of Interacting Genes/Proteins (STRING) v10 (http://string-db.org/) using default settings. Proteins are represented by spheres. Lines linking proteins indicate evidence for interactions: a red line indicates the presence of gene fusion (genes that are sometimes fused into single open reading frames); a green line – gene neighborhood (genes that reside within 300 bp on the same strand in the genome); a blue line – co-occurrence (gene families whose occurrence patterns across genomes show similarities); a purple line - experimental evidence (interaction extracted from protein-protein interaction databases); a yellow line – text mining (interaction extracted from scientific literature); a light blue line - database (interaction extracted from curated databases); a black line – co-expression (proteins whose genes are co-expressed in the same or in other species).

Further pathways enrichment analysis identified an immune/inflammatory response, cytoskeleton remodeling and apoptotic processes as the major deregulated processes in SALS (Fig. 3b, Supplementary Table 10). In particular, *Huntington’s disease signaling*, *T cell activation*, *Apoptosis and survival signaling* were the most significantly enriched pathways in both SALS patient subgroups, with *AKT1*, *NFKB1* and *SOS* as the major key involved genes. In addition, *ubiquitin-proteasome pathway*, *immune-cell mediated inflammatory response* and *apoptosis* were significantly up-regulated in SALS1, while *axonal guidance*, *oxidative stress* and *inflammatory intracellular signaling* cascades were mainly up-regulated in SALS2 (Fig. 4a). Notably, the majority of the enriched pathway for CNV-driven DEGs in SALS patients represented processes already associated with ALS pathogenesis, such as *immune response*, *cell adhesion* and *cell communication* (Fig. 4a).

To better understand the interactions of the CNV-driven genes and identify the best candidate genes in SALS, a protein-protein interaction (PPI) network analysis of their encoding products was performed, revealing a highly interconnected functional network, also including a greater number of ALS-associated genes (Fig. 4b). The PPI network consisted of 147 nodes and 2787 edges, including 46 CNV-driven DEGs in SALS. Node degree ≥10 was selected as the threshold. *UBA52*, *RPS27A* and *HIST2H3A* were selected as the hub genes.

To assess the value of CNV-driven genes as potential biomarkers for patient-specific diagnosis and prognosis, we reviewed and analyzed the literature on the CNV-driven genes exhibiting the same expression tendencies. A total of 24 candidate gene markers were selected, including 6 up-regulated CNV-driven DEGs in SALS1 and 18 deregulated (10 up-regulated and 8 down-regulated) CNV-driven genes in SALS2. Interestingly, some of these candidate genes (*TIMP2*, *AKT1*, *MMP9*, *CST3*, *SMN1* and *SMN2*) were previously associated with susceptibility to ALS while the remaining 18 genes (*GAA*, *KIF1A*, *MC1R*, *MECP2*, *ALPL*, *HSPG2*, *L1CAM*, *PLEC*, *STK11*, *CSF1*, *F2*, *GSTM3*, *TRAF2*, *HSPA5*, *HTT*, *IL6*, *LETMD1*, *SOAT1*) represent novel candidate mediators for disease progression. The set of CNV-driver genes also included many patient-specific ‘druggable’ genes that may represent good candidates for the development of personalized, molecularly targeted therapies for SALS patients (Table 4).

**Table 4:** The most promising candidate CNV-driven genes and their utility as potential biomarkers/targets for SALS.

Symbol	Entrez Gene Name	Location	Family	Molecular aberration	Tissue/Cells	Biological Fluid detectability	Biomarker Application(s)	Diseases	Drug(s)
**SALS1-specific Biomarkers/Targets**									
**CST3**	cystatin C	Extracellular Space	other	Gain/UP	Brain, Cerebral Cortex	Blood, Plasma/Serum, Bronchoalveolar Lavage Fluid, Cerebral Spinal Fluid, Saliva, Sputum, Synovium/Synovial Fluid, Tears, Urine	diagnosis, efficacy, prognosis, safety	Immunological/Inflammatory Disease, Neurological Disease, Skeletal and Muscular Disorders, *et al*.	—
GAA	glucosidase alpha, acid	Cytoplasm	enzyme	Gain/UP	Brain, Cerebral Cortex, Spinal Cord	Bronchoalveolar Lavage Fluid, Urine	diagnosis	Immunological/Inflammatory Disease, Neurological Disease, Skeletal and Muscular Disorders, *et al*.	Voglibose, Miglustat
KIF1A	kinesin family member 1A	Cytoplasm	other	Gain/UP	Cerebral Cortex	Blood, Plasma/Serum	diagnosis, prognosis	Immunological Disease, Neurological Disease, *et al*.	—
MC1R	melanocortin 1 receptor	Plasma Membrane	G-protein coupled receptor	Gain/UP	Brain	Blood	diagnosis	Immunological/Inflammatory Disease, Neurological Disease, Skeletal and Muscular Disorders	—
MECP2	methyl-CpG binding protein 2	Nucleus	transcription regulator	Gain/UP	Brain, Cerebral Cortex, Spinal Cord	Not detected in biofluid	unspecified application	Immunological/Inflammatory Disease, Neurological Disease, Skeletal and Muscular Disorders, *et al*.	—
**TIMP2**	TIMP metallopeptidase inhibitor 2	Extracellular Space	other	Gain/UP	Brain, Cerebral Cortex, Spinal Cord	Blood, Plasma/Serum, Cerebral Spinal Fluid, Urine	efficacy	Immunological/Inflammatory Disease, Skeletal and Muscular Disorders, *et al*.	Pravastatin, ABT751 (inhibitors)
**SALS2-specific Biomarkers/Targets**									
**AKT1**	AKT serine/threonine kinase 1	Cytoplasm	kinase	Gain/UP	Brain, Cerebral Cortex	Blood	diagnosis, efficacy, response to therapy	Immunological/Inflammatory Disease, Neurological Disease, Skeletal and Muscular Disorders, *et al*.	Thalidomide, XL418, ABT100 (inhibitors)
ALPL	alkaline phosphatase, liver/bone/kidney	Plasma Membrane	phosphatase	Gain/UP	Spinal Cord	Blood, Plasma/Serum, Bronchoalveolar Lavage Fluid, Urine	efficacy, safety	Immunological/Inflammatory Disease, Neurological Disease, Skeletal and Muscular Disorders, *et al*.	Alendronic acid, Zoledronic acid, Dexamethasone, Zaprinast (inhibitors)
HSPG2	heparan sulfate proteoglycan 2	Extracellular Space	enzyme	Gain/UP	Spinal Cord	Blood, Plasma/Serum, Cerebral Spinal Fluid, Bronchoalveolar Lavage Fluid Tears, Urine	unspecified application	Immunological/Inflammatory Disease, Neurological Disease, Skeletal and Muscular Disorders, *et al*.	—
L1CAM	L1 cell adhesion molecule	Plasma Membrane	other	Gain/UP	Brain, Cerebral Cortex	Blood, Plasma/Serum, Synovium/Synovial Fluid, Urine	unspecified application	Immunological/Inflammatory Disease, Neurological Disease, Skeletal and Muscular Disorders, *et al*.	Ethanol, Rivanicline, Anabaseine, Ketamine, Mecamylamine, Bupropion (inhibitors)
PLEC	plectin	Cytoplasm	other	Gain/UP	Brain, Cerebral Cortex, Spinal Cord	Blood, Plasma/Serum	unspecified application	Immunological/Inflammatory Disease, Neurological Disease, Skeletal and Muscular Disorders, *et al*.	—
STK11	serine/threonine kinase 11	Cytoplasm	kinase	Gain/UP	Brain, Cerebral Cortex, Spinal Cord	Blood	efficacy	Immunological/Inflammatory Disease, Neurological Disease, Skeletal and Muscular Disorders, *et al*.	—
CSF1	colony stimulating factor 1	Extracellular Space	cytokine	Gain/UP	Brain, Cerebral Cortex, Spinal Cord	Blood, Plasma/Serum, Cerebral Spinal Fluid, Bronchoalveolar Lavage Fluid, Synovium/Synovial Fluid, Urine	diagnosis	Immunological/Inflammatory Disease, Neurological Disease, Skeletal and Muscular Disorders, *et al*.	—
F2	coagulation factor II, thrombin	Extracellular Space	peptidase	Gain/UP	Brain, Cerebral Cortex	Blood, Plasma/Serum, Cerebral Spinal Fluid, Bronchoalveolar Lavage Fluid, Tears, Urine	diagnosis, unspecified application	Immunological/Inflammatory Disease, Neurological Disease, Skeletal and Muscular Disorders, *et al*.	enoxaparin, desirudin, dabigatran etexilate, Fibrinogen, ximelagatran, thrombin inhibitor, antithrombin alfa, aspirin/dabigatran etexilate, dabigatran, ulinastatin, aspirin/bivalirudin, argatroban, bivalirudin, lepirudin
GSTM3	glutathione S-transferase mu 3	Cytoplasm	enzyme	Gain/UP	Cerebral Cortex	Blood, Plasma/Serum, Urine	diagnosis, prognosis	Neurological Disease, *et al*.	—
TRAF2	TNF receptor associated factor 2	Cytoplasm	enzyme	Gain/UP	Brain	Blood, Plasma/Serum	diagnosis	Immunological/Inflammatory Disease, *et al*.	—
HSPA5	heat shock protein family A (Hsp70) member 5	Cytoplasm	enzyme	Loss/DOWN	Amygdala, Brain, Cerebellum, Cerebral Cortex	Blood, Plasma/Serum,Synovium/Synovial Fluid, Tears, Urine	efficacy, unspecified application	Immunological/Inflammatory Disease, Neurological Disease, Skeletal and Muscular Disorders, *et al*.	Mefloquine (activation)
HTT	huntingtin	Cytoplasm	transcription regulator	Loss/DOWN	Brain, Cerebral Cortex	Blood, Plasma/Serum	unspecified application	Immunological/Inflammatory Disease, Neurological Disease, Skeletal and Muscular Disorders, *et al*.	
IL6	interleukin 6	Extracellular Space	cytokine	Loss/DOWN	Cortical neurons, Brain, Cerebral Cortex, Spinal Cord	Blood, Plasma/Serum, Synovium/Synovial Fluid, Tears, Urine	diagnosis, disease progression, efficacy, prognosis, response to therapy, safety, unspecified application	Immunological/Inflammatory Disease, Neurological Disease, Skeletal and Muscular Disorders, *et al*.	Tocilizumab, Siltuximab, Mifamurtide, Mycophenolate mofetil, Acetaminophen, Rifampicin, Prostaglandin E2, Morphine, Hyaluronic acid (activators)
LETMD1	LETM1 domain containing 1	Plasma Membrane	other	Loss/DOWN	Brain, Cerebral Cortex	Blood, Plasma/Serum	unspecified application	Neurological Disease, *et al*.	
**MMP9**	matrix metallopeptidase 9	Extracellular Space	peptidase	Loss/DOWN	Brain, Cerebral Cortex	Blood, Plasma/Serum, Bronchoalveolar Lavage Fluid, Saliva, Sputum, Synovium/Synovial Fluid, Tears, Urine	diagnosis, disease progression, efficacy, prognosis, unspecified application	Immunological/Inflammatory Disease, Neurological Disease, Skeletal and Muscular Disorders, *et al*.	GS-5745, Rebimastat, Marimastat, Prinomastat, Glucosamine, Ciprofloxacin, Aclarubicin, Prostaglandin E2, Phorbol 12-myristate 13-acetate (activators)
**SMN1**/**SMN2**	survival of motor neuron 1, telomeric	Nucleus	other	Loss/DOWN	Brain, Cerebral Cortex, Spinal Cord	Blood	diagnosis	Neurological Disease, Skeletal and Muscular Disorders, *et al*.	
SOAT1	sterol O-acyltransferase 1	Cytoplasm	enzyme	Loss/DOWN	Pituitary Gland	Blood	unspecified application	Immunological/Inflammatory Disease, Neurological Disease, Skeletal and Muscular Disorders, *et al*.	pactimibe, ezetimibe/fluvastatin, atorvastatin/ezetimibe, ezetimibe/rosuvastatin, ezetimibe/fenofibrate, ezetimibe/simvastatin, ezetimibe, hesperetin

The table lists CNV-driven genes showing the same expressional tendencies between DNA copy number and mRNA expression in SALS subgroups as potential candidate biomarkers and therapeutic targets for ALS. Target and biomarker assessment was performed by using dedicated tools in IPA and MetaCore.

UP: upregulation.

DOWN: downregulation.

## Discussion

In this work, we reported the first fully integrated analysis of CNVs and gene expression profiling derived from the same SALS patients to provide a more comprehensive genomic framework for dissecting molecular heterogeneity of ALS and identify the DEGs with alterations in genomic segments that may represent novel potential markers and/or therapeutic targets.

Taking advantage of the custom-made *NeuroArray* platform, designed to uncover CNVs in clinically relevant genes for ALS and other neurological diseases^25^, we performed an exon-focused evaluation of structural imbalances occurring in motor cortex samples from 30 SALS and 10 control patients trascriptomically characterized in our previous work^17^. A large number of aberrations were detected in over 10% of SALS patients, with the highest number of gains documented at chromosomes 14 and 17, and the majority of losses found on the p arm of chromosome 1 and at cytoband 20q11.22-q11.23 (Fig. 1c and Supplementary Table 1). Notably, at 17p region the *SREBF1*, a gene encoding a lipogenic transcription factor whose expression levels were increased in the spinal cords of FALS and SALS patients as well as in ALS animal models, and whose direct causative role in excitotoxicity-induced neuronal cell death, has been extensively established^26–28^.

Our analysis also revealed distinct genomic signatures associated with two previously characterized transcriptome-based SALS subgroups. In particular, the loss of chromosome 1p was the most common chromosomal aberration in SALS1 (~90%), while 60–70% of the SALS2 patients showed simultaneous deletion of precise regions of chromosomes 3q and 18p (Fig. 1c, Table 1, Supplementary Table 1)^29^. It is interesting to note that genes in these CNV regions showed the same subgroup-specific expressional tendencies. In fact, the 1p33 deletion includes *STIL*, a gene involved in neural protection and survival and whose expression was down-regulated in SALS1, suggesting that a “loss-of-function” of this gene may contribute to render motor neurons vulnerable to excitotoxic insults in these patients^30^. Likewise, SALS2 patients carried the deletion of 18p11.22 that encompassed *NDUFV2*, one of the many components of the mitochondrial oxidative phosphorylation pathway, whose expression levels were decreased in the same patient subgroup. Defects in this subunit have been associated with altered energy production, mitochondrial dysfunction and oxidative stress, representing a risk factor for several neuronal diseases, including ALS^31^.

Compared with previous CNV reports^32^, some genomic aberrations identified in our study partially or totally overlap with those previously associated with ALS, further supporting them as disease susceptibility variants (Table 3). Among these, of particular interest is the deletion of chromosomal region 15q22.2-q22.31 that was detected exclusively in SALS patients and not altered in any controls in our study nor listed in the Database of Genomic Variants – DGV.

Besides strengthening previously reported results, we identified new potential susceptibility loci that were overrepresented in SALS patients and absent in controls (Table 2). Among these, we distinguished the deletion at the chromosome 1q32.2, which encompassed *CR1* (also known as *CD35*), a member of the human regulator of complement activation gene cluster^33,34^. This gene encodes one of the major immune adherence receptors and plays an important role in immune complex processing and clearance via reducing activation of classical and alternative complement cascade activity. Deletion of *CR1* is consistent with the lower mRNA expression level of this receptor in SALS patients, providing support for aberrant complement regulation as a part of ALS process and highlighting the potential use of complement molecules as disease biomarkers^35,36^. Another interesting association is represented by a 162.362-kb duplicated region in 1p36.12 spanning several neuronal genes, including the *FBXO42* encoding an important member of the F-box protein family involved in the ubiquitin-proteasome system and already known to be associated with ALS (Table 2)^37^. Of particular interest, three CNV regions (17q12, 22q11.21; 6q13) were selectively detected in specific SALS patient subgroups and absent in all control individuals, providing the basis for a CNV-based molecular classification of the disease (Table 2). In particular, the deletion at the 6q13 region harbors the *SLC17A5* gene encoding sialin, a vesicular excitatory amino acid transporter, whose loss-of-function leads to defect in myelin structure and function, contributing to the disruption of axonal integrity and the motor phenotype^38,39^. The increase in gene copy numbers for the 17q12 region encompassing several chemokines (i.e., *CCL5* and *CCL3*) is consistent with the observation that high mRNA expression levels of these chemokines may increase activation of the inflammatory system and changes in blood-brain barrier permeability, two key mechanisms implicated in, and possibly aggravating, motor neuron damage^40,41^.

By integrating the analysis of CNVs and gene expression profiling in the same patients and tissue samples, we found that 71.2% of CNV genes were differentially expressed in SALS patients in comparison to controls, the majority of them were cluster specific, further suggestive of the great divergence of two SALS subgroups at the molecular level (Fig. 2b,c, Supplementary Tables 7 and 8). Among these genes, 49% showed a positive association between CNV and mRNA expression, suggesting them as potential driver genes in ALS. These included some genes (*SYNE1*, *SDK1*, *EEF1D*, *GSDMD*, *TIMP2*, *CST3*, *ALAD*, *AKT1*, *EPHA4*, *SPAST*, *SMN1*, *SETX*, *ATXN2*, *TBK1*, *SPG11*, *TAF15*, *MMP9*) previously reported as potential risk factors in ALS, as well as novel candidates whose association with ALS was previously unappreciated. These genes may represent overlapping genetic signatures among different neurological condition, providing additional features for exploring ALS pathogenesis. To name a few of those detected exclusively in SALS patients (i.e., absent from controls) are *ALDH4A1*, a component of the mitochondrial matrix contributing to protect cells from oxidative stress^42^; *BIN1*, the most significant late-onset susceptibility locus for Alzheimer’s disease whose alterations in expression levels and splicing seem to induce muscle weakness and T tubule alterations^43^, and *RABGAP1L*, a GTPase-activating protein whose loss-of-function exacerbates neuronal loss^44^.

On the other hand, our analysis identified a number of CNV genes, including known ALS-related genes (i.e., *NIPA1*, *NAIP*, *VPS54* and *GRIN3B*), which do not appear to exert any apparent influence on expression levels, suggesting that the expression of these genes may be not gene-dose dependent and that they are likely to represent secondary ‘passenger’ events in ALS pathogenesis. Genes differently regulated at the transcriptomic and genomic level (i.e., showing no or negative correlation), in turn, could result from other, non-CNV-related regulatory mechanisms, such as those associated with gene mutation, promoter methylation, and non-coding RNA regulation (Fig. 2b,c, Supplementary Tables 7 and 8). An example is *KIF5A*, a gene encoding a neuronal kinesin heavy chain that acts as a molecular motor and whose genetic alteration was recently associated with ALS susceptibility^10^. SALS2 patients showed a selective downregulation of *KIF5A* not overlapping with any CNV, suggesting reduced mRNA expression of this gene is probably due to defective splicing events and/or loss-of-function variants rather than to deletion within its genomic region. It is interesting to note that in the same subgroup we also observed defects in axonal transport as well as a general downregulation of genes involved in mitochondrial oxidative phosphorylation machinery, suggesting a link between loss-of-function events in *KIF5A* and impaired transport and dysfunction of mitochondria in ALS^17,45^.

Integrated analysis of CNVs and corresponding expression data represents an effective approach to elucidate mediators and mechanisms involved in ALS. However, it has become clear that the process of motor neuronal degeneration is complex and requires many genomic alterations acting in concert. This also emerged by our analysis of PPI network constructed for CNV-driven DEGs that revealed a great number of close interconnections and identified three hub genes (*UBA52*, *RPS27A* and *HIST2H3A)* involved in inflammatory and immune responses (Fig. 4b)^46^. We also put forward a ‘systems biology’ analysis to identify biological processes and signaling pathways that were overly represented by CNV-driven genes in both SALS patient subgroups. Functional enrichment analysis of these candidate genes showed that they were mainly involved in immune/inflammatory signaling, neuronal migration, differentiation and survival, and neurite outgrowth, supporting the concept that these pathways may crosstalk with each other to drive the disease pathogenesis (Fig. 4a). In particular, *Huntington’s disease (HD) signaling* and *Protein folding and processing* were among the top canonical pathways for both SALS patient subgroups, supporting the possibility of a causal link between protein aggregation, neurotoxicity and disease severity in ALS and other neurological diseases, like HD^47,48^ (Fig. 4a). Among the most important candidate genes involved in these processes is *AKT1*, a gene encoding a cAMP-dependent serine-threonine kinase that was amplified in both SALS subgroup and overexpressed in SALS2. Our results are supported by previous evidence that abnormal AKT activation is implicated in several cellular mechanisms involved in ALS, such as the altered elimination of toxic protein aggregates, increasing oxidative stress and rendering cells susceptible to ROS-triggered cell death^49^. Our PPI network analysis identified AKT as a highly interconnected node, suggesting that alterations in this protein are not mere passenger events, but may have a great impact on one or more signaling pathways that are recurrently involved in ALS. Pharmacological inhibition of AKT and its downstream pathways has already demonstrated neuroprotective effects by modulating the activation state of microglial cells during neuroinflammation, and promoting cellular clearance in neurodegenerative storage diseases, suggesting a potential role of AKT inhibitors in ALS treatment^50,51^.

It is interesting to note that although partially complementary and convergent, the two SALS patient clusters showed different significantly deregulated processes and mediators. In particular, antigen processing and presentation, and extracellular matrix organization were the most significantly enriched pathways for the CNV-driven genes in SALS1, while the pathways of highest significance in SALS2 were associated with axonal guidance, oxidative stress and inflammatory intracellular signaling cascades (Fig. 4a). Therefore, a careful monitoring of these signaling cascades may help to better diagnose the specific subtype of ALS and optimize treatment strategies. To this regard, the convergent functional analysis of CNV-driven genes also pinpointed known and novel candidate therapeutic targets and biomarkers for early diagnosis, molecular subtyping and targeted therapy in SALS (Table 4). In particular, among CNV-driven genes in SALS1 showing the same expressional tendencies between DNA copy number and mRNA expression, we distinguished some genes (*KIF1A*, *MC1R* and *MECP2*) that were not previously implicated in ALS, representing new candidates for molecularly guided diagnosis and treatments. Within the cluster of CNV-driven genes in SALS2, we found a large number of new candidate ALS genes mainly implicated in oxidative and inflammatory signaling cascades^52–57^. Among these, identification of gain and overexpression of *TRAF2* was in accordance with previous evidence that correlated elevated expression levels of this gene with inflammatory processes in PD and other neurological disorders^58,59^. On the contrary, copy number loss and reduced expression levels *HSPA5* in SALS2 may reflect the suppression of neuroprotective role of this molecule against ER stress-associated cell death, leading to oxidative stress and alterations in calcium homeostasis, and rendering neurons vulnerable to degeneration^60–64^. Concordantly, pharmacological activation of HSPA5 and its co-chaperones has demonstrated to exert neuroprotective effects on motor neurons of ALS by reducing ER stress-mediated cell death, supporting a translational potential for HSPA5 induction as a therapy against ALS and other neurologic disorders^65^.

Overall, our study provided the first comprehensive and integrated map of genomic and transcriptional events characterizing different SALS subtypes, revealing key drivers and etiopathogenic mechanisms that may have been masked by considering SALS pathology as a single entity. Despite the most obvious limitation in this research was that of a small and unbalanced number of samples, our brain tissue samples represent a vanishingly rare resource for investigating molecular mechanisms underlying neurological disorders. The importance of an integrative analysis such as the one presented here, emerges from recent published data that highlight the presence of somatic changes in brain tissue of patients affected by neurological diseases^66,67^. Moreover, the custom-designed platform used in this study has some disadvantages, including limited use for discovering novel genes or gene features and inability to detect nucleotide repeat expansions or balanced structural chromosomal abnormalities. However, the possibility to simultaneously detect multiple genes involved in neurological disorders may allow for differential diagnosis between common neurological disorders, refine the genotype-phenotype correlations and explore the potential genetic overlapping signatures among different neurological conditions.

In conclusion, the present study proposes the use of a multi-omics analysis as a promising approach for the identification of somatic alteration and candidate drivers in ALS for defining disease subtype and directing molecular targeted clinical trials that more accurately reflect inter-individual differences among patients. Future more in-depth functional and integrative omics studies will be necessary to verify our findings and explore the impact of candidate genes on the outcome of the disease.

## Material and Methods

**Figure 5:**
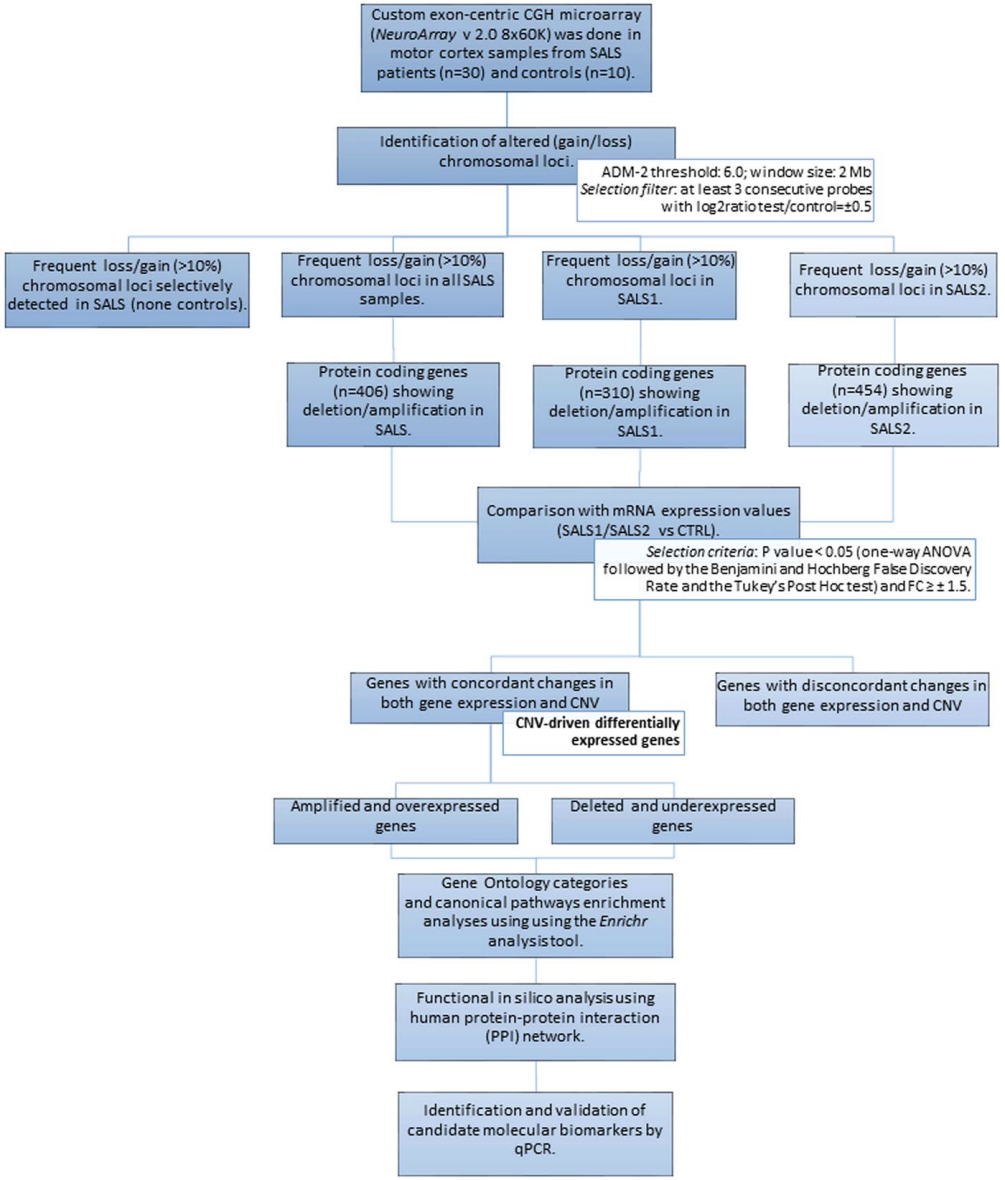
Experimental workflow of multi-omics analysis for characterization of CNV-driven differentially expressed genes in SALS patients. The workflow depicts the steps performed in this study, from data acquisition to the visualization, validation and export of results in various output formats. See *Materials and Methods* section for details.

The analysis workflow is shown in Fig. 5 and described below.

### Subject cohorts and sample preparation

All samples were provided by the Department of Pathology of the Academic Medical Center (University of Amsterdam). This cohort included motor cortex samples from 30 patients with clear SALS diagnosis and 10 control individuals collected as previously described^17^, and whose clinico-pathological parameters are detailed in the Supplementary Table 12. Informed consent was obtained for the use of brain tissues and for access to medical records for research purposes, and approval was obtained from the relevant local ethical committees for medical research. All experiments were performed in accordance with relevant guidelines and regulations of both institutions. Genomic DNA was extracted from 10μm-thick sections using the QIAamp Fast DNA Tissue Kit according to the manufacturer’s instructions (QIAGEN, Hilden, Germany). The extracted genomic DNA was quantified by using the NanoDrop ND-1000 spectrophotometer (Thermo Fisher Scientific, MA, USA), and assessed for quality by microcapillary electrophoresis on 2100 Bioanalyzer (Agilent Technologies, Palo Alto, CA).

### *NeuroArray* aCGH processing and data analysis

High-resolution exon-centered analysis of CNVs was done using an 8 × 60 K custom exon-centric *NeuroArray* platform v.2.0 (Agilent Technologies, Santa Clara, CA), tailored to detect single/multi-exon deletions and duplications in a large panel of ALS-related genes (n = 154) and to others additional neurological disorders (Supplementary Table 13)^25^. Details concerning the *NeuroArray* aCGH platform can be found in Supplementary Materials. DNA labeling and hybridization on *NeuroArray* were performed according to the manufacturer’s protocol (Agilent Technologies, Santa Clara, CA). Briefly, DNA test and a reference of the same sex (Euro Reference, Agilent Technologies, Santa Clara, CA), both at the concentration of 500 ng, were double digested with RsaI and AluI for 2 hours at 37 °C. After heat inactivation of the enzymes at 65 °C for 20 min, each digested sample was labeled by random priming by using the genomic DNA Enzymatic Labelling Kit (Agilent Technologies, Santa Clara, CA) for 2 hours using Cy5-dUTP for patient DNAs and Cy3-dUTP for reference DNAs. Labeled products were column purified by using the SureTag DNA Labeling Kit Purification Columns (Agilent Technologies, Santa Clara, CA). After probe denaturation and pre-annealing with Cot-1 DNA, hybridization was performed at 65 °C with rotation for 24 hr. After two washing steps, arrays were scanned at 3 µm resolution using an Agilent G4900DA SureScan Microarray Scanner System and aCGH image data were processed using Agilent’s Feature Extraction software to assess the array spot quality as well as check signal and background intensity statistics in the default setting. Feature extracted raw data was normalized, analyzed and visualized using Agilent CytoGenomics v. 4.0.3.12 and Genomic Workbench v. 7.0.4.0 software (Agilent Technologies, Santa Clara, CA, USA). Briefly, after filtering for saturated and non-uniform probes, data were normalized by GC correction with a window size of 2 kb and Diploid Peak Centralization. The Centralization Normalization Algorithm with a threshold of 6.0 and a bin size of 10 was also used for detecting aberrant regions or regions of constant CNVs. Aberrations were detected by the Aberration Detection Method II algorithm (ADM-2), with a sensitivity threshold of 6.0 and moving average window of 2 Mb, which permits to identify all aberrant intervals in a given sample with consistently high or low log ratios based on the statistical score. An aberration filter was applied for identifying copy number alterations; changes were considered as true positive events with a minimum log2 ratio test/control of ±0.5 and a minimum of 3 consecutive probes. Positive statistical score meant amplification, while a negative score indicated deletion. Human reference sequence hg19 assembly was used to define the genomic coordinates of detected CNVs. Raw data of the microarrays are available at NCBI’s Gene Expression Omnibus (GEO) with the accession number GSE107375.

### Identification of significantly altered genomic regions and CNV-encompassed genes

For statistical analysis, the ALS samples were divided into two groups (SALS1 and SALS2) based on their previously characterized gene expression profiles^17^. The chromosomal distribution and the frequency of the copy number gains and losses in both SALS subgroups were also investigated. Using ADM-2 generated interval based amplification and deletion data, penetrance analysis was performed to find the percentage of samples that share aberrations in a particular genomic region among multiple samples. A recurrent CNV was called when the gains or losses occurred in at least 10% of the SALS patients, both with and without the assignment into the hierarchically determined two sets. Multiple amplifications and deletions were counted as separate events. Aberrant intervals were also filtered taking into account those occurring in at least 10% of the cases and absent in individual controls. In addition, to assess the effective relations between the detected CNVs and ALS pathogenesis, we compared these aberrant regions with those previously associated with ALS via screening of publicly available databases (i.e., CNVD) and published literature.

Frequent amplifications and deletions observed in both SALS patient subgroups were reviewed and annotated to the human hg19 reference genome and then were screened out for only genes included in the *NeuroArray* aCGH design. In addition, significant probe signals were clustered for pathologies according to their location on causative or susceptibility genes through a homemade script on R-platform, in order to search for CNVs in candidate genes of ALS disease^68^.

### Source of gene expression data

Gene expression data set E-MTAB-2325 was downloaded from EBI ArrayExpress database, which was annotated using the platform of GPL6480 (Agilent-014850 Whole Human Genome Microarray 4 × 44 K G4112F). A total of 40 samples were selected out, including 10 normal and 30 SALS motor cortex samples. Raw signal values were thresholded to 1, log2 transformed, normalized to the 50th percentile, and baselined to the median of all samples using GeneSpringGX v.14.5 (Agilent Technologies, Italy). Fold change (FC) values were calculated between SALS patients and individual controls. Positive FC meant over-expression, whereas negative FC indicated under-expression. Probes not corresponding to an ENTREZ ID were removed. In cases where several probes corresponded to one ENTREZ ID, the probe showing the highest variance over all samples was chosen for further analysis. Genes that showed a significant P value < 0.05 (one-way ANOVA followed by the Benjamini and Hochberg False Discovery Rate and the Tukey’s Post Hoc test) and FC ≥ ± 1.5 were considered differentially expressed and were taken for further analysis.

### Integration of the aCGH and gene expression data

To assess the contribution of genomic aberrations to global gene expression pattern changes in SALS and identify CNV-associated DEGs, we performed an integrated analysis of differential expression values and the corresponding DNA copy number changes through a gene-by-gene approach. In particular, each gene expression measurement was assigned to the corresponding copy number probe interrogating the same named gene. A CNV-driven gene was defined when the gene expression trend was consistent with the copy number change (i.e., up-regulated gene transcript with a chromosomal amplification and down-regulated gene transcript with a chromosomal deletion).

### Functional enrichment and biological network analysis

The function of CNV-associated DEGs in SALS patients was annotated and analyzed according to the three organizing principles of Gene Ontology (BP: Biological Process, MF: Molecular Function, CC: Cellular Component) by using the enrichment analysis tool Enrichr ^69^. To interpret the biological significance of CNV-driven genes in the context of known biological pathways, we used QIAGEN’s Ingenuity Pathway Analysis (IPA®; http://www.ingenuity.com/) and MetaCore repository (Clarivate Analytics, Philadelphia, United States) ^70^. Both these programs identify significantly enriched biological pathways and signaling cascades that are associated with a given list of genes by calculating the hypergeometric distribution. P-value < 0.05 was set as the threshold to filter out significant terms. In addition, to increase the statistical power of our analysis, we compared our results with three other pathway enrichment analysis tools and databases (KEGG; Reactome; Panther) and selected signaling pathways that were identified as significantly deregulated by two or more platforms.

To better understand the interactions of the CNV-driven genes and identify the best candidate genes in SALS, an extended protein-protein interaction (PPI) network of their encoding products was predicted by using the STRING database ^71^ and visualized with the Cytoscape v.3.5.0 software ^72^. The extended network was constructed by using the CNV-driven genes as seed molecules and setting a high level of confidence between molecular interactions (high confidence score of at least 0.8) and a maximum number of interactions to 100. In order to identify the “Hub” nodes, a network topology analysis was performed by using the Cytoscape plug-in NetworkAnalyzer based on topological parameters ^73^. The relative importance of the genes in each network, meaning their ability to hold together the communicating nodes in a biological network, was determined based on the node centrality measure setting the topological parameter “node degree” ≥10. Nodes with high degree (hub genes) represented the genes having important biological functions: the higher the value, the higher the relevance of the gene in connecting regulatory molecules. Likewise, values of edge betweenness were mapped with the edge size: high values of this parameter correspond to a large edge size. The final PPI network was visualized based on node degree and edge betweenness parameters.

Finally, target and biomarker assessment tools in both IPA and Metacore were used to screen candidate CNV-driven genes in order to identify potential candidate biomarkers and therapeutic targets for ALS.

### Real-Time quantitative polymerase chain reaction (RT-qPCR) validation

To confirm the reliability of our data, we validated the *NeuroArray* CGH results performing ad hoc real-time quantitative polymerase chain reaction (RT-qPCR) assays. Briefly, we used DNA extracted from the motor cortex samples of 15 donors assayed by *NeuroArray*, including 5 controls, 4 SALS1 and 6 SALS2 (Supplementary Table 11**)**. From the list of CNV-driven DEGs genes, we selected 5 candidates (*GAA*, *KIF1A*, *CSF1*, *TRAF2*, *HSPA5*) on the basis of their potential clinical relevance as patient-specific biomarkers and therapeutic targets (Supplementary Table 11**)**. The primer sets flanking the putative exonic imbalances were designed using the PrimerBlast tool (http://www.ncbi.nlm.nih.gov/tools/primer-blast/) as described ^74^ and were available in Supplementary Table 11. RT-qPCR was performed in triplicate using the LightCycler 1.5 (Roche Diagnostics, Germany). Cycling conditions were 95 °C for 15 s followed by 40 cycles of 95 °C (5 s), 60 °C (15 s) and one cycle of 95 °C (15 s), 60 °C (60 s), 95 °C (15 s). The data were analyzed by the 2 − ΔΔCt method that requires a healthy control sample (diploid) as a calibrator in all amplifications ^75^. 2 − ΔΔCt ≥ 1.4 or ≤0.6 was defined as copy number gain or loss, respectively, whereas 2 − ΔΔCt values included from 0.8 to 1.2 were considered as normal diploid. As calibrator control, we used the same DNA reference hybridized in *NeuroArray* experiments. The specific PCR products were confirmed by the results of melting curve analysis and agarose gel electrophoresis.

### Ethics approval and consent to participate

Experiments involving human participants have been approved by an ethical committee (Ethics Committee of the Amsterdam Academic Medical Center, approved protocol: W11_073) for medical research and have been performed in accordance with ethical standards. Informed consent Informed consent was obtained from all individual participants included in the study.

## Supplementary information

Supplementary Materials

## Data Availability

All data generated during this study are included in this published article and the additional files. Transcriptional data are available at EBI ArrayExpress database with the accession number E-MTAB-2325. Raw data from *NeuroArray* aCGH analysis are available at NCBI’s Gene Expression Omnibus (GEO) with the accession number GSE107375.
