# Supplementary material for: Integrative multi-omic analysis identifies new drivers and pathways in molecularly distinct subtypes of ALS
Source: Sci Rep. 2019 Jul 10;9:9968. doi: 10.1038/s41598-019-46355-w (PMC6620285; doi:10.1038/s41598-019-46355-w)
Supplement: Supplementary file 1 — Supplementary Materials [file 41598_2019_46355_MOESM1_ESM.pdf]

Original Article

Integrative multi-omic analysis identifies new drivers and pathways in molecularly distinct subtypes of ALS

Giovanna Morello[a], Maria Guarnaccia[a], Antonio Gianmaria Spampinato[a], Salvatore Salomone[b], Velia D'Agata[c], Francesca Luisa Conforti[f], Eleonora Aronica[d,e] and Sebastiano Cavallaro[a,#]

[a] *Institute of Neurological Sciences, Italian National Research Council, Catania, Italy; Department of Biomedical and Biotechnological Sciences, Section of* [b]*Pharmacology and* [c]*Human Anatomy and Histology, University of Catania, Catania, Italy.*[d]*Department of NeuroPathology, Academic Medical Center, Amsterdam, the Netherlands;* [e]*Swammerdam Institute for Life Sciences, Center for Neuroscience; University of Amsterdam, Amsterdam, The Netherlands;* [f]*Department of Pharmacy, Health and Nutritional Sciences, University of Calabria, Rende (Cosenza), Italy*

[#]Corresponding author: Sebastiano Cavallaro, M.D., Ph.D., Institute of Neurological Sciences (ISN), CNR, Via Paolo Gaifami, 18, 95125 Catania, Italy. Phone: +39.095.7338111; Fax: +39.095.7338110; E-mail: sebastiano.cavallaro@cnr.it

Supplementary Information

Supplementary Figure and Table Legends

Supplementary Figure 1. Legend describing symbols used in the interaction map.

Supplementary Figure 2. Correlation analysis of DNA copy number and expression variation in two randomized SALS patient subgroup. Venn diagrams compare the number of protein-coding genes obtained from CNV analyses with the genes found to be differentially expressed Random group 1 (A) and Random group 2 (B) of SALS patients.

Supplementary Table 1. General information of patients and individual controls.

Supplementary Table 2. List of genes represented in the *NeuroArray* aCGH v.2.0 design.

Supplementary Table 3. Comparison of the data obtained for 5 selected genes by *NeuroArray* aCGH analysis and Real-Time RT-PCR.

Supplementary Table 4. Chromosomal regions with amplifications and deletions present in > 10% of SALS patients.

Supplementary Table 5. Chromosomal regions with gains and losses present in > 10% of SALS1 patients.

Supplementary Table 6. Chromosomal regions with gains and losses present in > 10% of SALS2 patients.

Supplementary Table 7. List of genes located in the most recurrent CNVs of SALS patients (pentrance > 10%).

Supplementary Table 8. List of genes located in the most recurrent CNVs of SALS1 patients (pentrance > 10%).

Supplementary Table 9. List of genes located in the most recurrent CNVs of SALS2 patients (pentrance > 10%).

Supplementary Table 10. List of CNV-driven differentially expressed genes in SALS1 patients.

Supplementary Table 11. List of CNV-driven differentially expressed genes in SALS2 patients.

Supplementary Table 12. The top 15 functional enrichment of CNV-associated DEGs in SALS**.**

Supplementary Table 13. The signal pathway enrichment of CNV-associated DEGs.

Supplementary Table 14. Cluster random assignment of SALS patients.

Supplementary Materials and Methods

Gene selection and aCGH design strategy. Our customized *NeuroArray v.2.0* aCGH platform was built to permit a high-density probe coverage in the coding region of clinically relevant genes associated with ALS (n=154). In order to perform a differential diagnosis with other neurological disorders, we also included genes related to other neurological conditions, such as Alzheimer's disease and other dementias, Parkinson's disease, Epilepsies, Rett Syndrome, Autosomal dominant and recessive Limb-Girdle Muscular Dystrophy, Muscular Duchenne/Becker Dystrophy, Hereditary Spastic Paraplegia, Spinocerebellar Ataxia , Neurofibromatosis, Tuberous sclerosis, Peripheral Neuropathy and Stroke.

The selection of genes included in the array, relied on our extensive expertise in clinics and genetics of neurological diseases and on updated literature data, has been extended to the entire

currently known sets of disease-linked genes collected in specific public databases available online, such as ALSgene (http://www.alsgene.org/), ALZgene (http://www.alzgene.org/), PDgene (http://www.pdgene.org/). The set of selected genes embraces known and putative risk factors, disease-causing genes, and other related genetic regions affected by different types of mutations was resported in the **Supplementary Table 2**.

The array design was carried out as previously described [1] by using the web-based Agilent SureDesign Software (Agilent Technologies, Santa Clara, CA). This web application allows to define regions of interest and select the “best-performing” probes from the High-Density (HD) Agilent probe library. Chromosomal coordinates of all RefSeq genes were extrapolated from open-source databases, Biomart (http://www.biomart.org/) and UCSC Genome Browser according to Human Feb. 2009 Assembly (GRCh37/hg19) (http://genome.ucsc.edu). Exon coordinates of neuro-related genes were selected and formatted using a homemade R script [2] and then uploaded on SureDesign. In particular, The exon coordinates were sorted and separated in to 3 bins: exons that were up to 150 bp (“small” group), 151-500 bp (“medium” group) and 500bp< (“large” group). For the small and medium groups, we have added 100 bp and 50 bp, respectively, each side of the interval in order to facilitate picking probes for smaller exons. For the large exon group, we performed search in SureDesign test server with The exon coordinates were sorted and separated in to 3 bins: exons that are up to 150 bp (“small” group), 151-500 bp (“medium” group) and 500bp< (“large” group). For the small and medium groups, we have added 100 bp and 50 bp, respectively, each side of the interval in order to facilitate accurate picking probes for smaller exons. Candidate probes (3 probes per interval) were scored and filtered using bioinformatics prediction criteria for probe sensitivity, specificity, and responsiveness under appropriate conditions. We also selected a limited number of probes (1109) with the SureDesign Genomic Tiling option to cover regions inadequately represented in the Agilent database. All probes had similar characteristics: isothermal probes, with melting temperature (Tm) of 80° C and probe length of about 60-mers, in accordance to the manufacturer's specifications. Biological probes were randomly distributed in an 8x60K array format that allows to process simultaneously eight samples in a single experiment. The routinely used the Agilent Human CGH Replicate Probe Groups (5000 features) and 8700 probes scattered along the genome, with a probe density of around 1 probe every about 355 kb, that constituted the backbone were also included in the array design. Microarray slides were produced using Agilent’s Sure-Print Inkjet technology (Agilent Technologies, Santa Clara, CA). The final design of the microarray included 44858 target-specific probes for 1099 selected genes with a median probe spacing of 310.102 kbp. Gene and locus positions are based on the Human Genome GRCh37 (hg19) assembly of UCSC genome browser.

NeuroArray v.2.0 design	
Total genes	1099
Genes in common among two or more disorders	317
Total exonic targets	16271
Target coverage	99,5%
Total target size	7.294275 Mb
Total probes	44858
Total unique probes from HD Database	43749
Total unique probes by genomic tiling	1109
Median probe spacing	310.102 Kbp
Mean target size	448.2991211Kbp
Uncovered targets	79
Globally disease- targeted gene panels	ALS, Epilepsies, RTT, LGMD, DMD/BMD, HSP, SCA, NF, TSC, PN, Stroke

Supplementary References

1. La Cognata, V. *et al.* A customized high-resolution array-comparative genomic hybridization to explore copy number variations in Parkinson's disease. *Neurogenetics* **17,** 233–244 (2016).
2. Development Core Team, R. *R: A Language and Environment for Statistical Computing*. *R. Found. Stat. Comput.* **1,** (2011). URL www.R-project.org/

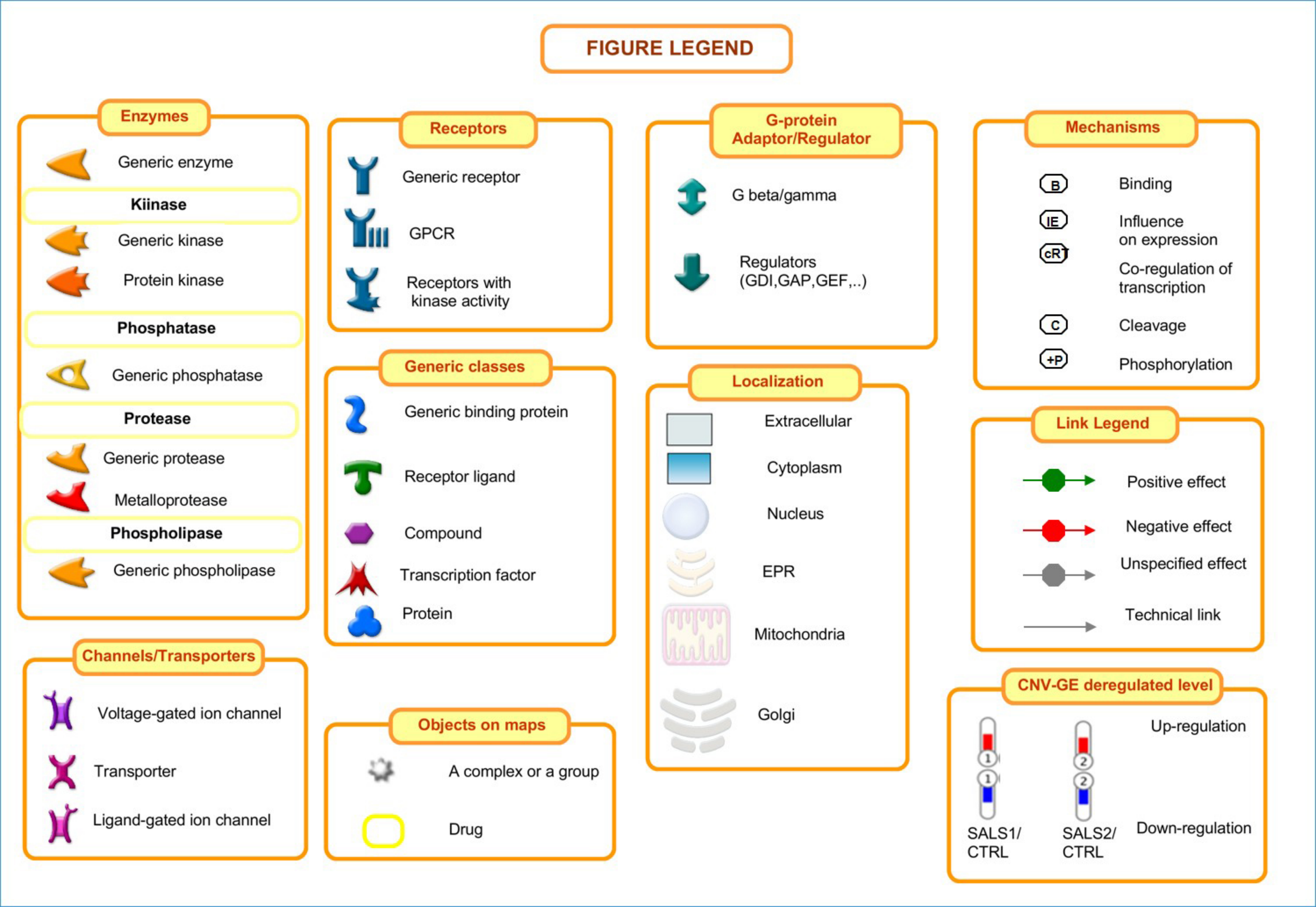
FIGURE LEGEND
Enzymes
Generic enzyme
Kiinase
Generic kinase
Protein kinase
Phosphatase
Generic phosphatase
Protease
Generic protease
Metalloprotease
Phospholipase
Generic phospholipase
Channels/Transporters
Voltage-gated ion channel
Transporter
Ligand-gated ion channel
Receptors
Generic receptor
GPCR
Receptors with kinase activity
Generic classes
Generic binding protein
Receptor ligand
Compound
Transcription factor
Protein
Objects on maps
A complex or a group
Drug
G-protein Adaptor/Regulator
G beta/gamma
Regulators (GDI,GAP,GEF,..)
Localization
Extracellular
Cytoplasm
Nucleus
EPR
Mitochondria
Golgi
Mechanisms
B
Binding
IE
Influence on expression
cRT
Co-regulation of transcription
C
Cleavage
+P
Phosphorylation
Link Legend
Positive effect
Negative effect
Unspecified effect
Technical link
CNV-GE deregulated level
Up-regulation
1
1
2
2
SALS1/ CTRL
SALS2/ CTRL
Down-regulation

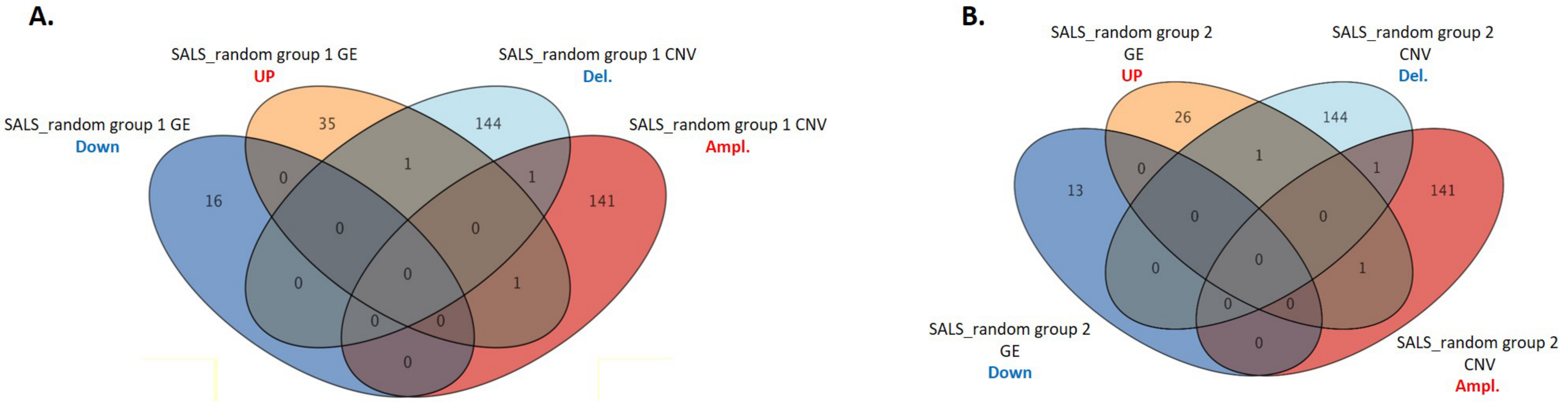
A.
SALS_random group 1 GE
UP
SALS_random group 1 CNV
Del.
SALS_random group 1 GE
Down
SALS_random group 1 CNV
Ampl.
35
144
16
141
0
1
1
0
0
0
0
1
0
0
0
B.
SALS_random group 2
GE
UP
SALS_random group 2
CNV
Del.
SALS_random group 2
GE
Down
SALS_random group 2
CNV
Ampl.
26
144
13
141
0
1
1
0
0
0
0
1
0
0
0

Supplementary Table 1. General information of patients and individual controls.

Patient number	Ethnicity	Gender	Age (years)	PMI (hours)	Disease state	Survival from initial diagnosis (months)	Cluster assignment
1	caucasian	Male	31	8	Control	n/a	Control
2	caucasian	Male	59	7	Control	n/a	Control
3	caucasian	Male	68	8	Control	n/a	Control
4	caucasian	Female	71	9	Control	n/a	Control
5	caucasian	Male	48	4	Control	n/a	Control
6	caucasian	Male	58	7	Control	n/a	Control
7	caucasian	Male	60	6.5	Control	n/a	Control
8	caucasian	Male	44	9	Control	n/a	SALS 1
9	caucasian	Male	73	10	Control	n/a	SALS 2
10	caucasian	Male	39	8	Control	n/a	SALS 2
11	caucasian	Male	67	8	SALS	90	SALS 1
12	caucasian	Male	41	10	SALS	96	SALS 1
13	caucasian	Male	65	6.5	SALS	38	SALS 1
14	caucasian	Male	68	6	SALS	30	SALS 1
15	caucasian	Female	67	8	SALS	27	SALS 1
16	caucasian	Male	43	6	SALS	38	SALS 1
17	caucasian	Male	54	3	SALS	31	SALS 1
18	caucasian	Male	38	7	SALS	42	SALS 1
19	caucasian	Male	45	6.5	SALS	38	SALS 1
20	caucasian	Female	46	7	SALS	31	SALS 1
21	caucasian	Female	65	7	SALS	52	SALS 1
22	caucasian	Male	54	8	SALS	49	SALS 1
23	caucasian	Male	51	4	SALS	60	SALS 1
24	caucasian	Male	69	10	SALS	20	SALS 1
25	caucasian	Male	68	7	SALS	18	SALS 1
26	caucasian	Female	68	8	SALS	22	SALS 1
27	caucasian	Male	61	3	SALS	11	SALS 1
29	caucasian	Female	40	5	SALS	130	SALS 2
30	caucasian	Male	41	3	SALS	72	SALS 2
31	caucasian	Female	61	6	SALS	43	SALS 2
32	caucasian	Female	61	10	SALS	29	SALS 2
33	caucasian	Female	51	7	SALS	29	SALS 2
34	caucasian	Male	63	7.5	SALS	27	SALS 2
35	caucasian	Female	70	4	SALS	30	SALS 2
36	caucasian	Female	69	9	SALS	52	SALS 2
37	caucasian	Female	64	5.3	SALS	71	SALS 2
38	caucasian	Male	46	7.5	SALS	48	SALS 2
39	caucasian	Male	55	6	SALS	18	SALS 2
40	caucasian	Male	51	8	SALS	23	SALS 2
41	caucasian	Male	59	8	SALS	13	SALS 2

Fresh-frozen motor cortex samples were obtained from the Department of Neuropathology of the Academic Medical Center, University of Amsterdam, The Netherlands. Control patients died from a non-neurological disease (myocardial infarction, renal failure, or pulmonary embolism). All patients included in the study displayed no signs of infection before death. Informed consent was obtained for the use of brain tissue and for access to medical records for research purposes, approval was obtained from the local ethical committees for medical research.

Supplementary Table 2. List of genes represented in the *NeuroArray* aCGH v.2.0 design.

Gene name	Gene description	Chromosome	Gene start (bp)	Gene end (bp)	Size (bp)
A2M	alpha-2-macroglobulin	12	9220260	9268825	48566
A2M-AS1	A2M antisense RNA 1 (head to head)	12	9217773	9220651	2879
A2MP1	alpha-2-macroglobulin pseudogene 1	12	9381129	9428413	47285
A4GALT	alpha 1,4-galactosyltransferase (P blood group)	22	43088127	43117304	29178
A4GNT	alpha-1,4-N-acetylglucosaminyltransferase	3	137842560	137851229	8670
AAK1	AP2 associated kinase 1	2	69688532	69901481	212950
AARS	alanyl-tRNA synthetase	16	70286198	70323446	37249
AATF	apoptosis antagonizing transcription factor	17	35306175	35414171	107997
**AATK**	apoptosis associated tyrosine kinase	17	79091095	79139877	48783
ABCA1	ATP binding cassette subfamily A member 1	9	107543283	107690518	147236
ABCA12	ATP binding cassette subfamily A member 12	2	215796266	216003151	206886
ABCA13	ATP binding cassette subfamily A member 13	7	48211055	48687092	476038
ABCA2	ATP binding cassette subfamily A member 2	9	139901686	139923367	21682
ABCA3	ATP binding cassette subfamily A member 3	16	2325882	2390747	64866
ABCA4	ATP binding cassette subfamily A member 4	1	94458393	94586688	128296
ABCA6	ATP binding cassette subfamily A member 6	17	67074843	67138029	63187
ABCB1	ATP binding cassette subfamily B member 1	7	87133175	87342611	209437
ABCB11	ATP binding cassette subfamily B member 11	2	169779448	169887832	108385
ABCB4	ATP binding cassette subfamily B member 4	7	87031013	87109751	78739
ABCB7	ATP binding cassette subfamily B member 7	X	74273115	74376567	103453
ABCC1	ATP binding cassette subfamily C member 1	16	16043434	16236931	193498
ABCC4	ATP binding cassette subfamily C member 4	13	95672083	95953687	281605
ABCC5	ATP binding cassette subfamily C member 5	3	183637722	183735803	98082
ABCC6	ATP binding cassette subfamily C member 6	16	16242785	16317379	74595
ABCC6P1	ATP binding cassette subfamily C member 6 pseudogene 1	16	18582484	18609650	27167
ABCC8	ATP binding cassette subfamily C member 8	11	17414432	17498449	84018
ABCC9	ATP binding cassette subfamily C member 9	12	21950335	22094336	144002
ABCD3	ATP binding cassette subfamily D member 3	1	94883933	94984222	100290
ABCE1	ATP binding cassette subfamily E member 1	4	146019084	146050331	31248
ABCG1	ATP binding cassette subfamily G member 1	21	43619799	43717354	97556
ABCG4	ATP binding cassette subfamily G member 4	11	119019722	119033360	13639
ABCG5	ATP binding cassette subfamily G member 5	2	44039611	44066004	26394
ABHD14A-ACY1	ABHD14A-ACY1 readthrough	3	52009066	52023199	14134
ABHD17B	abhydrolase domain containing 17B	9	74477368	74525847	48480
ABHD17C	abhydrolase domain containing 17C	15	80972025	81047962	75938
ABHD2	abhydrolase domain containing 2	15	89630690	89745591	114902
ABHD3	abhydrolase domain containing 3	18	19230858	19284766	53909
ABL1	ABL proto-oncogene 1, non-receptor tyrosine kinase	9	133589333	133763062	173730
ABL2	ABL proto-oncogene 2, non-receptor tyrosine kinase	1	179068462	179198819	130358
ABLIM1	actin binding LIM protein 1	10	116190872	116444762	253891
ABLIM2	actin binding LIM protein family member 2	4	7967039	8160559	193521
ACACA	acetyl-CoA carboxylase alpha	17	35441923	35766909	324987
ACAD11	acyl-CoA dehydrogenase family member 11	3	132276982	132379567	102586
ACAD8	acyl-CoA dehydrogenase family member 8	11	134123389	134135749	12361
ACADL	acyl-CoA dehydrogenase, long chain	2	211052663	211090215	37553
ACADM	acyl-CoA dehydrogenase, C-4 to C-12 straight chain	1	76190036	76253260	63225
ACAN	aggrecan	15	89346674	89418585	71912
ACAP2	ArfGAP with coiled-coil, ankyrin repeat and PH domains 2	3	194995465	195163807	168343
ACE	angiotensin I converting enzyme	17	61554422	61599205	44784
ACE2	angiotensin I converting enzyme 2	X	15579156	15620271	41116
ACER3	alkaline ceramidase 3	11	76571911	76737841	165931
ACHE	acetylcholinesterase (Cartwright blood group)	7	100487615	100494594	6980
ACKR3	atypical chemokine receptor 3	2	237476430	237491001	14572
ACMSD	aminocarboxymuconate semialdehyde decarboxylase	2	135596117	135659604	63488
ACO1	aconitase 1	9	32384618	32454767	70150
ACOX1	acyl-CoA oxidase 1	17	73937588	73975515	37928
ACOXL	acyl-CoA oxidase like	2	111490150	111875799	385650
ACSF2	acyl-CoA synthetase family member 2	17	48503519	48552206	48688
ACTA2	actin, alpha 2, smooth muscle, aorta	10	90694831	90751147	56317
ACTN4	actinin alpha 4	19	39138289	39222223	83935
ACTR3	ARP3 actin related protein 3 homolog	2	114647537	114720173	72637
ACTR3C	ARP3 actin-related protein 3 homolog C	7	149941005	150020814	79810
ACVR1B	activin A receptor type 1B	12	52345451	52390862	45412
ACVR2B	activin A receptor type 2B	3	38495342	38534633	39292
ADAM10	ADAM metallopeptidase domain 10	15	58887403	59042177	154775
ADAM12	ADAM metallopeptidase domain 12	10	127700950	128077024	376075
ADAM17	ADAM metallopeptidase domain 17	2	9628615	9695921	67307
ADAM19	ADAM metallopeptidase domain 19	5	156822542	157002783	180242
ADAM2	ADAM metallopeptidase domain 2	8	39601254	39695808	94555
ADAM22	ADAM metallopeptidase domain 22	7	87563458	87832204	268747
ADAM29	ADAM metallopeptidase domain 29	4	175750819	175899331	148513
ADAM32	ADAM metallopeptidase domain 32	8	38964509	39142430	177922
ADAM9	ADAM metallopeptidase domain 9	8	38854388	38962663	108276
ADAMTS12	ADAM metallopeptidase with thrombospondin type 1 motif 12	5	33523640	33892297	368658
ADAMTS16	ADAM metallopeptidase with thrombospondin type 1 motif 16	5	5140443	5320417	179975

ADAMTS17	ADAM metallopeptidase with thrombospondin type 1 motif 17	15	100511794	100882210	370417
ADAMTS18	ADAM metallopeptidase with thrombospondin type 1 motif 18	16	77281710	77469011	187302
ADAMTS19	ADAM metallopeptidase with thrombospondin type 1 motif 19	5	128795958	129074376	278419
ADAMTS2	ADAM metallopeptidase with thrombospondin type 1 motif 2	5	178537852	178772431	234580
ADAMTS20	ADAM metallopeptidase with thrombospondin type 1 motif 20	12	43747669	43945724	198056
ADAMTS6	ADAM metallopeptidase with thrombospondin type 1 motif 6	5	64444563	64777747	333185
ADAMTS9-AS2	ADAMTS9 antisense RNA 2	3	64670585	64997143	326559
ADAMTSL1	ADAMTS like 1	9	18473892	18910948	437057
ADAMTSL3	ADAMTS like 3	15	84322838	84708594	385757
ADAP2	ArfGAP with dual PH domains 2	17	29233362	29286340	52979
ADAR	adenosine deaminase, RNA specific	1	154554538	154600475	45938
ADARB1	adenosine deaminase, RNA specific B1	21	46493768	46646478	152711
ADARB2	adenosine deaminase, RNA specific B2 (inactive)	10	1228073	1779670	551598
ADCY1	adenylate cyclase 1	7	45613739	45762715	148977
ADCY10	adenylate cyclase 10, soluble	1	167778625	167883453	104829
ADCY2	adenylate cyclase 2	5	7396321	7830194	433874
ADCY5	adenylate cyclase 5	3	123001143	123168605	167463
ADCY8	adenylate cyclase 8	8	131792547	132054672	262126
ADCY9	adenylate cyclase 9	16	4003388	4166186	162799
ADCYAP1R1	ADCYAP receptor type I	7	31092076	31151089	59014
ADD3	adducin 3	10	111756126	111895323	139198
ADGRG1	adhesion G protein-coupled receptor G1	16	57653442	57698944	45503
ADH1C	alcohol dehydrogenase 1C (class I), gamma polypeptide	4	100257649	100274184	16536
ADH5	alcohol dehydrogenase 5 (class III), chi polypeptide	4	99992132	100009952	17821
ADIPOR2	adiponectin receptor 2	12	1797740	1897844	100105
**ADK**	adenosine kinase	10	75910960	76469061	558102
ADNP	activity dependent neuroprotector homeobox	20	49505585	49547958	42374
ADORA2B	adenosine A2b receptor	17	15848231	15879060	30830
ADRA1A	adrenoceptor alpha 1A	8	26605667	26724790	119124
ADRA1B	adrenoceptor alpha 1B	5	159343790	159399551	55762
ADRA2A	adrenoceptor alpha 2A	10	112836790	112840658	3869
ADRB1	adrenoceptor beta 1	10	115803806	115806667	2862
ADRB2	adrenoceptor beta 2	5	148206156	148208196	2041
ADRB3	adrenoceptor beta 3	8	37820516	37824483	3968
ADSL	adenylosuccinate lyase	22	40742507	40786467	43961
ADTRP	androgen dependent TFPI regulating protein	6	11712287	11807279	94993
AEBP2	AE binding protein 2	12	19556979	19873735	316757
AFAP1	actin filament associated protein 1	4	7760441	7941653	181213
AFAP1L2	actin filament associated protein 1 like 2	10	116054583	116164515	109933
AFF1	AF4/FMR2 family member 1	4	87856154	88062206	206053
AFF2	AF4/FMR2 family member 2	X	147582139	148082193	500055
AFF3	AF4/FMR2 family member 3	2	100162323	100759201	596879
AFG3L2	AFG3 like matrix AAA peptidase subunit 2	18	12328943	12377313	48371
AFM	afamin	4	74347400	74369691	22292
AGAP1	ArfGAP with GTPase domain, ankyrin repeat and PH domain 1	2	236402733	237040444	637712
AGBL1	ATP/GTP binding protein like 1	15	86685227	87572283	887057
AGBL2	ATP/GTP binding protein like 2	11	47681143	47736941	55799
AGBL3	ATP/GTP binding protein like 3	7	134671259	134832715	161457
AGBL4	ATP/GTP binding protein like 4	1	48998527	50489585	1491059
AGER	advanced glycosylation end-product specific receptor	6	32148745	32152101	3357
AGFG1	ArfGAP with FG repeats 1	2	228336868	228425930	89063
AGMO	alkylglycerol monooxygenase	7	15239943	15601640	361698
AGO3	argonaute 3, RISC catalytic component	1	36396319	36538101	141783
AGO4	argonaute 4, RISC catalytic component	1	36273773	36323491	49719
AGPAT4	1-acylglycerol-3-phosphate O-acyltransferase 4	6	161551011	161695093	144083
**AGT**	angiotensinogen	1	230838269	230850043	11775
AGTPBP1	ATP/GTP binding protein 1	9	88161455	88356944	195490
AHCY	adenosylhomocysteinase	20	32868074	32899608	31535
AHCYL1	adenosylhomocysteinase like 1	1	110527308	110566357	39050
AHCYL2	adenosylhomocysteinase like 2	7	128864864	129070052	205189
AHI1	Abelson helper integration site 1	6	135604670	135818914	214245
AHR	aryl hydrocarbon receptor	7	17338246	17385776	47531
AHRR	aryl-hydrocarbon receptor repressor	5	304291	438406	134116
AHSG	alpha 2-HS glycoprotein	3	186330712	186339107	8396
AIG1	androgen induced 1	6	143381633	143661441	279809
AIMP1	aminoacyl tRNA synthetase complex interacting multifunctional protein 1	4	107236701	107270383	33683
AK4	adenylate kinase 4	1	65613232	65697828	84597
**AK8**	adenylate kinase 8	9	135600965	135754164	153200
AKAP12	A-kinase anchoring protein 12	6	151561134	151679692	118559
AKAP13	A-kinase anchoring protein 13	15	85923802	86292586	368785
AKAP3	A-kinase anchoring protein 3	12	4724674	4758213	33540
AKAP4	A-kinase anchoring protein 4	X	49955406	49965664	10259
AKAP6	A-kinase anchoring protein 6	14	32798479	33300567	502089
AKAP8L	A-kinase anchoring protein 8 like	19	15490859	15529952	39094
AKT1	AKT serine/threonine kinase 1	14	105235686	105262088	26403
AKT3	AKT serine/threonine kinase 3	1	243651535	244014381	362847

**ALAD**	aminolevulinate dehydratase	9	116148597	116163613	15017
ALB	albumin	4	74262831	74287129	24299
ALCAM	activated leukocyte cell adhesion molecule	3	105085753	105295744	209992
ALDH1A2	aldehyde dehydrogenase 1 family member A2	15	58245622	58790065	544444
ALDH1L1-AS2	ALDH1L1 antisense RNA 2	3	125898908	125929012	30105
ALDH2	aldehyde dehydrogenase 2 family (mitochondrial)	12	112204691	112247782	43092
ALDH4A1	aldehyde dehydrogenase 4 family member A1	1	19197926	19229275	31350
ALDH5A1	aldehyde dehydrogenase 5 family member A1	6	24495080	24537435	42356
ALDH6A1	aldehyde dehydrogenase 6 family member A1	14	74523553	74551196	27644
ALDH7A1	aldehyde dehydrogenase 7 family member A1	5	125877533	125931110	53578
ALDH9A1	aldehyde dehydrogenase 9 family member A1	1	165631453	165668100	36648
ALG1	ALG1, chitobiosyldiphosphodolichol beta-mannosyltransferase	16	5083703	5137380	53678
ALG11	ALG11, alpha-1,2-mannosyltransferase	13	52586534	52603800	17267
ALG12	ALG12, alpha-1,6-mannosyltransferase	22	50293877	50312106	18230
ALG13	ALG13, UDP-N-acetylglucosaminyltransferase subunit	X	110909043	111003877	94835
ALG2	ALG2, alpha-1,3/1,6-mannosyltransferase	9	101978708	101984238	5531
ALG3	ALG3, alpha-1,3- mannosyltransferase	3	183960089	183967336	7248
ALG6	ALG6, alpha-1,3-glucosyltransferase	1	63833261	63904233	70973
ALG8	ALG8, alpha-1,3-glucosyltransferase	11	77811982	77850706	38725
ALG9	ALG9, alpha-1,2-mannosyltransferase	11	111657010	111750149	93140
ALK	ALK receptor tyrosine kinase	2	29415640	30144432	728793
ALMS1	ALMS1, centrosome and basal body associated protein	2	73612886	73837920	225035
ALOX5	arachidonate 5-lipoxygenase	10	45869661	45941561	71901
ALOX5AP	arachidonate 5-lipoxygenase activating protein	13	31309645	31338556	28912
ALPK2	alpha kinase 2	18	56148479	56296189	147711
ALPL	alkaline phosphatase, liver/bone/kidney	1	21835858	21904905	69048
**ALS2**	ALS2, alsin Rho guanine nucleotide exchange factor	2	202565277	202645912	80636
AMBRA1	autophagy and beclin 1 regulator 1	11	46417964	46615675	197712
AMD1	adenosylmethionine decarboxylase 1	6	111195973	111216916	20944
AMFR	autocrine motility factor receptor	16	56395364	56459450	64087
AMIGO2	adhesion molecule with Ig like domain 2	12	47469490	47473734	4245
AMMECR1	Alport syndrome, mental retardation, midface hypoplasia and elliptocytosis chromosomal region gene 1	X	109437414	109683461	246048
AMPH	amphiphysin	7	38423305	38671167	247863
AMT	aminomethyltransferase	3	49454211	49460186	5976
AMY2B	amylase, alpha 2B (pancreatic)	1	104096437	104122156	25720
ANAPC13	anaphase promoting complex subunit 13	3	134196548	134205558	9011
**ANG**	angiogenin	14	21152336	21167130	14795
ANGEL2	angel homolog 2	1	213165524	213189168	23645
ANGPT1	angiopoietin 1	8	108261721	108510283	248563
ANK1	ankyrin 1	8	41510739	41754280	243542
ANK2	ankyrin 2	4	113739265	114304896	565632
ANK3	ankyrin 3	10	61786056	62493248	707193
ANKH	ANKH inorganic pyrophosphate transport regulator	5	14704910	14871887	166978
ANKHD1	ankyrin repeat and KH domain containing 1	5	139781399	139929163	147765
ANKMY1	ankyrin repeat and MYND domain containing 1	2	241418839	241508626	89788
**ANKRD11**	ankyrin repeat domain 11	16	89334038	89556969	222932
ANKRD12	ankyrin repeat domain 12	18	9136226	9285983	149758
ANKRD13C	ankyrin repeat domain 13C	1	70726271	70820417	94147
ANKRD17	ankyrin repeat domain 17	4	73939093	74124515	185423
ANKRD20A2	ankyrin repeat domain 20 family member A2	9	42368303	42411410	43108
ANKRD23	ankyrin repeat domain 23	2	97490263	97523671	33409
ANKRD27	ankyrin repeat domain 27	19	33087913	33167503	79591
ANKRD28	ankyrin repeat domain 28	3	15708743	15901278	192536
ANKRD30BP2	ankyrin repeat domain 30B pseudogene 2	21	14410481	14439354	28874
ANKRD36	ankyrin repeat domain 36	2	97779233	97930258	151026
ANKRD40	ankyrin repeat domain 40	17	48770551	48785285	14735
ANKRD46	ankyrin repeat domain 46	8	101521980	101572012	50033
ANKRD6	ankyrin repeat domain 6	6	90142889	90343553	200665
ANKRD7	ankyrin repeat domain 7	7	117854727	117882785	28059
ANKS1A	ankyrin repeat and sterile alpha motif domain containing 1A	6	34857042	35059179	202138
ANKS1B	ankyrin repeat and sterile alpha motif domain containing 1B	12	99120235	100378432	1258198
ANKS6	ankyrin repeat and sterile alpha motif domain containing 6	9	101493611	101559247	65637
ANKUB1	ankyrin repeat and ubiquitin domain containing 1	3	149478892	149686172	207281
ANO10	anoctamin 10	3	43396351	43733086	336736
ANO2	anoctamin 2	12	5641035	6055398	414364
ANO3	anoctamin 3	11	26210829	26684835	474007
ANO5	anoctamin 5	11	22214722	22304903	90182
ANO6	anoctamin 6	12	45609770	45834187	224418
ANO9	anoctamin 9	11	417933	442011	24079
ANTXR1	anthrax toxin receptor 1	2	69240310	69476459	236150
ANTXRLP1	anthrax toxin receptor-like pseudogene 1	10	47605211	47644793	39583
ANXA10	annexin A10	4	169013666	169108841	95176
**ANXA5**	annexin A5	4	122589110	122618268	29159
AOAH	acyloxyacyl hydrolase	7	36552456	36764154	211699
AOC1	amine oxidase, copper containing 1	7	150521715	150558592	36878
AOX1	aldehyde oxidase 1	2	201450591	201541787	91197

AP1B1	adaptor related protein complex 1 beta 1 subunit	22	29723669	29819168	95500
AP1S2	adaptor related protein complex 1 sigma 2 subunit	X	15843929	15873054	29126
AP1S3	adaptor related protein complex 1 sigma 3 subunit	2	224616403	224702744	86342
AP2A2	adaptor related protein complex 2 alpha 2 subunit	11	924894	1012239	87346
AP2B1	adaptor related protein complex 2 beta 1 subunit	17	33905065	34053436	148372
AP3B1	adaptor related protein complex 3 beta 1 subunit	5	77296349	77590579	294231
AP3M1	adaptor related protein complex 3 mu 1 subunit	10	75881524	75910821	29298
AP4B1	adaptor related protein complex 4 beta 1 subunit	1	114437370	114447823	10454
AP4B1-AS1	AP4B1 antisense RNA 1	1	114399257	114443859	44603
AP4E1	adaptor related protein complex 4 epsilon 1 subunit	15	51200869	51298097	97229
AP4M1	adaptor related protein complex 4 mu 1 subunit	7	99699172	99707968	8797
AP4S1	adaptor related protein complex 4 sigma 1 subunit	14	31494312	31562818	68507
AP5Z1	adaptor related protein complex 5 zeta 1 subunit	7	4815253	4833943	18691
APAF1	apoptotic peptidase activating factor 1	12	99038919	99129204	90286
APBA1	amyloid beta precursor protein binding family A member 1	9	72042446	72287222	244777
APBA2	amyloid beta precursor protein binding family A member 2	15	29129629	29410518	280890
APBB1	amyloid beta precursor protein binding family B member 1	11	6416355	6440644	24290
APBB1IP	amyloid beta precursor protein binding family B member 1 interacting protein	10	26727132	26856732	129601
APBB2	amyloid beta precursor protein binding family B member 2	4	40812044	41218731	406688
**APCDD1**	APC down-regulated 1	18	10454625	10489945	35321
APCDD1L-AS1	APCDD1L antisense RNA 1 (head to head)	20	57090435	57194944	104510
**APEX1**	apurinic/apyrimidinic endodeoxyribonuclease 1	14	20923350	20925927	2578
APH1A	aph-1 homolog A, gamma-secretase subunit	1	150237804	150241980	4177
APH1B	aph-1 homolog B, gamma-secretase subunit	15	63568217	63601325	33109
APLP2	amyloid beta precursor like protein 2	11	129939732	130014699	74968
APMAP	adipocyte plasma membrane associated protein	20	24943561	24973615	30055
APOA1	apolipoprotein A1	11	116706467	116708666	2200
APOA1-AS	APOA1 antisense RNA	11	116706833	116726445	19613
APOA2	apolipoprotein A2	1	161192082	161193421	1340
APOA5	apolipoprotein A5	11	116660083	116663136	3054
APOB	apolipoprotein B	2	21224301	21266945	42645
APOC1	apolipoprotein C1	19	45417504	45422606	5103
APOC3	apolipoprotein C3	11	116700422	116703788	3367
APOC4-APOC2	APOC4-APOC2 readthrough (NMD candidate)	19	45445495	45452822	7328
APOD	apolipoprotein D	3	195295573	195311076	15504
**APOE**	apolipoprotein E	19	45409011	45412650	3640
APOM	apolipoprotein M	6	31620193	31625987	5795
APOO	apolipoprotein O	X	23851470	23926057	74588
APP	amyloid beta precursor protein	21	27252861	27543446	290586
APPBP2	amyloid beta precursor protein binding protein 2	17	58520520	58603580	83061
APTX	aprataxin	9	32972604	33025166	52563
AQR	aquarius intron-binding spliceosomal factor	15	35147732	35262040	114309
**AR**	androgen receptor	X	66764465	66950461	185997
ARAF	A-Raf proto-oncogene, serine/threonine kinase	X	47420516	47431307	10792
AREL1	apoptosis resistant E3 ubiquitin protein ligase 1	14	75120140	75179818	59679
ARFGEF1	ADP ribosylation factor guanine nucleotide exchange factor 1	8	68085747	68255912	170166
ARHGAP10	Rho GTPase activating protein 10	4	148653214	148993931	340718
ARHGAP12	Rho GTPase activating protein 12	10	32094365	32217742	123378
ARHGAP15	Rho GTPase activating protein 15	2	143848931	144525921	676991
ARHGAP17	Rho GTPase activating protein 17	16	24930706	25026987	96282
**ARHGAP18**	Rho GTPase activating protein 18	6	129897277	130031370	134094
ARHGAP20	Rho GTPase activating protein 20	11	110447766	110583912	136147
ARHGAP21	Rho GTPase activating protein 21	10	24872538	25012597	140060
ARHGAP22	Rho GTPase activating protein 22	10	49654077	49864310	210234
ARHGAP24	Rho GTPase activating protein 24	4	86396267	86923823	527557
ARHGAP26	Rho GTPase activating protein 26	5	142149949	142608576	458628
ARHGAP28	Rho GTPase activating protein 28	18	6729717	6915715	185999
ARHGAP32	Rho GTPase activating protein 32	11	128834955	129149219	314265
ARHGAP35	Rho GTPase activating protein 35	19	47421933	47508334	86402
ARHGAP44	Rho GTPase activating protein 44	17	12692856	12894960	202105
ARHGAP6	Rho GTPase activating protein 6	X	11136239	11683821	547583
ARHGEF10	Rho guanine nucleotide exchange factor 10	8	1772142	1906807	134666
ARHGEF11	Rho guanine nucleotide exchange factor 11	1	156904632	157015162	110531
ARHGEF12	Rho guanine nucleotide exchange factor 12	11	120207787	120360645	152859
ARHGEF18	Rho/Rac guanine nucleotide exchange factor 18	19	7459999	7537363	77365
**ARHGEF28**	Rho guanine nucleotide exchange factor 28	5	72921983	73237818	315836
ARHGEF3	Rho guanine nucleotide exchange factor 3	3	56761446	57113357	351912
ARHGEF38	Rho guanine nucleotide exchange factor 38	4	106473777	106629250	155474
**ARHGEF4**	Rho guanine nucleotide exchange factor 4	2	131594489	131804836	210348
ARHGEF9	Cdc42 guanine nucleotide exchange factor 9	X	62854847	63005426	150580
ARID1B	AT-rich interaction domain 1B	6	157099063	157531913	432851
ARID4B	AT-rich interaction domain 4B	1	235294949	235491534	196586
ARID5B	AT-rich interaction domain 5B	10	63661059	63856703	195645
ARIH1	ariadne RBR E3 ubiquitin protein ligase 1	15	72766667	72879692	113026
ARIH2	ariadne RBR E3 ubiquitin protein ligase 2	3	48956254	49023815	67562
ARL15	ADP ribosylation factor like GTPase 15	5	53179775	53606412	426638

ARMC3	armadillo repeat containing 3	10	23216953	23326518	109566
ARMC9	armadillo repeat containing 9	2	232063260	232239548	176289
ARNT	aryl hydrocarbon receptor nuclear translocator	1	150782181	150849244	67064
ARNT2	aryl hydrocarbon receptor nuclear translocator 2	15	80696692	80890278	193587
ARNTL2	aryl hydrocarbon receptor nuclear translocator like 2	12	27485787	27576241	90455
ARPC3	actin related protein 2/3 complex subunit 3	12	110872630	110888227	15598
ARPP21	cAMP regulated phosphoprotein 21	3	35680437	35835988	155552
ARSA	arylsulfatase A	22	51061182	51066607	5426
ARSB	arylsulfatase B	5	78073032	78281910	208879
ARSG	arylsulfatase G	17	66255323	66418872	163550
ARSH	arylsulfatase family member H	X	2924654	2951612	26959
ARX	aristaless related homeobox	X	25021811	25034065	12255
ASAP1	ArfGAP with SH3 domain, ankyrin repeat and PH domain 1	8	131064353	131455906	391554
ASAP3	ArfGAP with SH3 domain, ankyrin repeat and PH domain 3	1	23755056	23811061	56006
ASB18	ankyrin repeat and SOCS box containing 18	2	237102095	237173052	70958
ASB3	ankyrin repeat and SOCS box containing 3	2	53897430	54087297	189868
ASB5	ankyrin repeat and SOCS box containing 5	4	177134824	177198722	63899
ASCC1	activating signal cointegrator 1 complex subunit 1	10	73856278	73976892	120615
ASCC3	activating signal cointegrator 1 complex subunit 3	6	100956070	101329248	373179
ASCL1	achaete-scute family bHLH transcription factor 1	12	103351464	103354294	2831
ASH1L	ASH1 like histone lysine methyltransferase	1	155305059	155532598	227540
ASIC2	acid sensing ion channel subunit 2	17	31340105	32501983	1161879
ASIC5	acid sensing ion channel subunit family member 5	4	156750881	156787425	36545
ASNS	asparagine synthetase (glutamine-hydrolyzing)	7	97481430	97501854	20425
ASPSCR1	ASPSCR1, UBX domain containing tether for SLC2A4	17	79934683	79975282	40600
ASRGL1	asparaginase like 1	11	62104920	62160882	55963
ASTN1	astrotactin 1	1	176826438	177134109	307672
ASTN2	astrotactin 2	9	119187504	120177348	989845
ATE1	arginyltransferase 1	10	123499939	123688316	188378
ATF2	activating transcription factor 2	2	175936978	176033110	96133
ATF3	activating transcription factor 3	1	212738676	212794119	55444
ATF6	activating transcription factor 6	1	161736084	161933860	197777
ATF7	activating transcription factor 7	12	53901640	54020199	118560
ATF7IP	activating transcription factor 7 interacting protein	12	14518610	14651697	133088
ATG10	autophagy related 10	5	81267844	81572676	304833
ATG2B	autophagy related 2B	14	96747595	96830207	82613
**ATG7**	autophagy related 7	3	11313995	11599139	285145
ATL1	atlastin GTPase 1	14	50999227	51099786	100560
ATM	ATM serine/threonine kinase	11	108093211	108239829	146619
ATN1	atrophin 1	12	7033626	7051484	17859
ATP10A	ATPase phospholipid transporting 10A (putative)	15	25922420	26110317	187898
ATP10B	ATPase phospholipid transporting 10B (putative)	5	159990127	160279221	289095
ATP11A	ATPase phospholipid transporting 11A	13	113344643	113541482	196840
ATP11B	ATPase phospholipid transporting 11B (putative)	3	182511288	182639423	128136
ATP13A2	ATPase 13A2	1	17312453	17338423	25971
ATP13A5	ATPase 13A5	3	192992579	193096632	104054
ATP1A2	ATPase Na+/K+ transporting subunit alpha 2	1	160085549	160113381	27833
ATP1A3	ATPase Na+/K+ transporting subunit alpha 3	19	42470734	42501649	30916
ATP1B4	ATPase Na+/K+ transporting family member beta 4	X	119495967	119516226	20260
ATP2A2	ATPase sarcoplasmic/endoplasmic reticulum Ca2+ transporting 2	12	110718561	110788898	70338
ATP5B	ATP synthase, H+ transporting, mitochondrial F1 complex, beta polypeptide	12	57031959	57039798	7840
ATP5E	ATP synthase, H+ transporting, mitochondrial F1 complex, epsilon subunit	20	57600522	57607437	6916
ATP6AP2	ATPase H+ transporting accessory protein 2	X	40440146	40465889	25744
ATP6V0A2	ATPase H+ transporting V0 subunit a2	12	124196865	124246302	49438
ATP6V1H	ATPase H+ transporting V1 subunit H	8	54628117	54756118	128002
ATP7A	ATPase copper transporting alpha	X	77166194	77305892	139699
ATP8A1	ATPase phospholipid transporting 8A1	4	42410390	42659122	248733
ATP8A2	ATPase phospholipid transporting 8A2	13	25946209	26599989	653781
ATP8B1	ATPase phospholipid transporting 8B1	18	55313658	55470333	156676
ATP8B4	ATPase phospholipid transporting 8B4 (putative)	15	50150435	50475014	324580
ATP9A	ATPase phospholipid transporting 9A (putative)	20	50213053	50385173	172121
ATP9B	ATPase phospholipid transporting 9B (putative)	18	76829285	77138283	308999
ATPAF2	ATP synthase mitochondrial F1 complex assembly factor 2	17	17880723	17942523	61801
ATRIP	ATR interacting protein	3	48488114	48507115	19002
**ATRN**	attractin	20	3451687	3631769	180083
ATRNL1	attractin like 1	10	116853124	117708503	855380
ATRX	ATRX, chromatin remodeler	X	76760356	77041702	281347
**ATXN1**	ataxin 1	6	16299343	16761722	462380
ATXN10	ataxin 10	22	46067678	46241187	173510
**ATXN2**	ataxin 2	12	111890018	112037480	147463
ATXN3	ataxin 3	14	92524896	92572965	48070
**ATXN3L**	ataxin 3 like	X	13336770	13338518	1749
ATXN7	ataxin 7	3	63850233	63989138	138906
ATXN7L1	ataxin 7 like 1	7	105245514	105517050	271537
ATXN8OS	ATXN8 opposite strand (non-protein coding)	13	70681345	70713561	32217

AUP1	ancient ubiquitous protein 1	2	74753772	74757066	3295
AUTS2	AUTS2, activator of transcription and developmental regulator	7	69063905	70258054	1194150
AVEN	apoptosis and caspase activation inhibitor	15	34158428	34331377	172950
AVL9	AVL9 cell migration associated	7	32535038	33078516	543479
AXDND1	axonemal dynein light chain domain containing 1	1	179334855	179523870	189016
AXIN1	axin 1	16	337440	402673	65234
AZIN1	antizyme inhibitor 1	8	103838585	103906092	67508
B2M	beta-2-microglobulin	15	45003675	45011075	7401
B3GAT2	beta-1,3-glucuronyltransferase 2	6	71566382	71666741	100360
B3GNT2	UDP-GlcNAc:betaGal beta-1,3-N-acetylglucosaminyltransferase 2	2	62423248	62451866	28619
B3GNTL1	UDP-GlcNAc:betaGal beta-1,3-N-acetylglucosaminyltransferase like 1	17	80900031	81009686	109656
B4GALT4	beta-1,4-galactosyltransferase 4	3	118930579	118959950	29372
**B4GALT6**	beta-1,4-galactosyltransferase 6	18	29202210	29265799	63590
B9D1	B9 domain containing 1	17	19240867	19281495	40629
BACE1	beta-secretase 1	11	117156402	117186975	30574
BACE2	beta-site APP-cleaving enzyme 2	21	42539728	42654445	114718
BACH2	BTB domain and CNC homolog 2	6	90636248	91006627	370380
BAD	BCL2 associated agonist of cell death	11	64037302	64052176	14875
BAG6	BCL2 associated athanogene 6	6	31606805	31620482	13678
BAIAP2L1	BAI1 associated protein 2 like 1	7	97920963	98030380	109418
BANK1	B-cell scaffold protein with ankyrin repeats 1	4	102332443	102995969	663527
BARD1	BRCA1 associated RING domain 1	2	215590370	215674428	84059
BAX	BCL2 associated X, apoptosis regulator	19	49458072	49465055	6984
BAZ1B	bromodomain adjacent to zinc finger domain 1B	7	72854728	72936608	81881
BAZ2B	bromodomain adjacent to zinc finger domain 2B	2	160175490	160473203	297714
BBOX1	gamma-butyrobetaine hydroxylase 1	11	27062272	27149356	87085
BBS4	Bardet-Biedl syndrome 4	15	72978527	73030817	52291
BBS9	Bardet-Biedl syndrome 9	7	33168856	33645680	476825
BBX	BBX, HMG-box containing	3	107241783	107530171	288389
BCAR3	breast cancer anti-estrogen resistance 3	1	94027347	94312706	285360
BCAS1	breast carcinoma amplified sequence 1	20	52553316	52687304	133989
BCAS3	BCAS3, microtubule associated cell migration factor	17	58754814	59470199	715386
BCAT1	branched chain amino acid transaminase 1	12	24964295	25102393	138099
BCHE	butyrylcholinesterase	3	165490692	165555260	64569
BCKDHB	branched chain keto acid dehydrogenase E1 subunit beta	6	80816364	81055987	239624
**BCL11B**	B-cell CLL/lymphoma 11B	14	99635624	99737861	102238
BCL2	BCL2, apoptosis regulator	18	60790579	60987361	196783
BCL2L13	BCL2 like 13	22	18111621	18213388	101768
**BCL6**	B-cell CLL/lymphoma 6	3	187439165	187463515	24351
BCL7A	BCL tumor suppressor 7A	12	122457328	122499948	42621
BCR	BCR, RhoGEF and GTPase activating protein	22	23521891	23660224	138334
BCYRN1	brain cytoplasmic RNA 1	2	47558199	47571656	13458
BDNF	brain derived neurotrophic factor	11	27676440	27743605	67166
BDNF-AS	BDNF antisense RNA	11	27528385	27719721	191337
BDP1	B double prime 1, subunit of RNA polymerase III transcription initiation factor IIIB	5	70751442	70863649	112208
BEAN1	brain expressed associated with NEDD4 1	16	66461200	66527432	66233
BECN1	beclin 1	17	40962152	40985367	23216
BEND4	BEN domain containing 4	4	42112955	42154895	41941
BEND7	BEN domain containing 7	10	13480484	13570974	90491
BEST1	bestrophin 1	11	61717293	61732987	15695
BEST3	bestrophin 3	12	70037140	70093256	56117
BEX5	brain expressed X-linked 5	X	101408680	101411029	2350
BFSP2	beaded filament structural protein 2	3	133118839	133194066	75228
BHMT	betaine--homocysteine S-methyltransferase	5	78407602	78428108	20507
BICC1	BicC family RNA binding protein 1	10	60272900	60591195	318296
BICD1	BICD cargo adaptor 1	12	32259769	32536567	276799
BICD2	BICD cargo adaptor 2	9	95473645	95527094	53450
BIN1	bridging integrator 1	2	127805603	127864931	59329
BIRC2	baculoviral IAP repeat containing 2	11	102217942	102249401	31460
BIRC6	baculoviral IAP repeat containing 6	2	32582096	32843966	261871
BLCAP	bladder cancer associated protein	20	36120874	36156333	35460
BLM	Bloom syndrome RecQ like helicase	15	91260558	91358859	98302
BLMH	bleomycin hydrolase	17	28575218	28619074	43857
BLOC1S5-TXNDC5	BLOC1S5-TXNDC5 readthrough (NMD candidate)	6	7881755	8064597	182843
BLVRA	biliverdin reductase A	7	43798279	43846939	48661
BMP2K	BMP2 inducible kinase	4	79697496	79837526	140031
BMP5	bone morphogenetic protein 5	6	55618443	55740362	121920
BMP7	bone morphogenetic protein 7	20	55743804	55841685	97882
BMPER	BMP binding endothelial regulator	7	33944523	34195484	250962
BMPR1B	bone morphogenetic protein receptor type 1B	4	95679119	96079599	400481
BMPR2	bone morphogenetic protein receptor type 2	2	203241659	203432474	190816
BNC2	basonuclin 2	9	16409501	16870841	461341
BOD1L1	biorientation of chromosomes in cell division 1 like 1	4	13570362	13629347	58986
BPTF	bromodomain PHD finger transcription factor	17	65821640	65980494	158855
BRAF	B-Raf proto-oncogene, serine/threonine kinase	7	140419127	140624564	205438
BRAP	BRCA1 associated protein	12	112079950	112123790	43841

BRCA1	BRCA1, DNA repair associated	17	41196312	41277500	81189
BRD1	bromodomain containing 1	22	50166931	50221160	54230
BRD2	bromodomain containing 2	6	32936437	32949282	12846
BRD3	bromodomain containing 3	9	136895427	136933657	38231
BRF1	BRF1, RNA polymerase III transcription initiation factor subunit	14	105675623	105781926	106304
BRINP3	BMP/retinoic acid inducible neural specific 3	1	190066792	190446759	379968
BRPF3	bromodomain and PHD finger containing 3	6	36164521	36200567	36047
BRWD1	bromodomain and WD repeat domain containing 1	21	40556102	40693485	137384
BRWD3	bromodomain and WD repeat domain containing 3	X	79926353	80065187	138835
BSCL2	BSCL2, seipin lipid droplet biogenesis associated	11	62457747	62477317	19571
BSN	bassoon presynaptic cytomatrix protein	3	49591922	49708978	117057
BSPH1	binder of sperm protein homolog 1	19	48471303	48495427	24125
BST1	bone marrow stromal cell antigen 1	4	15704573	15739936	35364
BTBD11	BTB domain containing 11	12	107712190	108053419	341230
BTBD2	BTB domain containing 2	19	1985447	2034880	49434
BTBD8	BTB domain containing 8	1	92545862	92613393	67532
BTBD9	BTB domain containing 9	6	38136227	38607924	471698
BTG4	BTG anti-proliferation factor 4	11	111338251	111383079	44829
BTNL2	butyrophilin like 2	6	32361740	32374905	13166
BTRC	beta-transducin repeat containing E3 ubiquitin protein ligase	10	103113820	103317078	203259
BUB1	BUB1 mitotic checkpoint serine/threonine kinase	2	111395275	111435691	40417
BUB1B	BUB1 mitotic checkpoint serine/threonine kinase B	15	40453224	40513337	60114
**BUB3**	BUB3, mitotic checkpoint protein	10	124913793	124924886	11094
BUD13	BUD13 homolog	11	116618886	116643704	24819
BVES	blood vessel epicardial substance	6	105544697	105585049	40353
C10orf107	chromosome 10 open reading frame 107	10	63422719	63526524	103806
C10orf126	chromosome 10 open reading frame 126	10	29135337	29170827	35491
C10orf55	chromosome 10 open reading frame 55	10	75669727	75682535	12809
C10orf76	chromosome 10 open reading frame 76	10	103605356	103815950	210595
C10orf90	chromosome 10 open reading frame 90	10	128113566	128359079	245514
C11orf49	chromosome 11 open reading frame 49	11	46958240	47185936	227697
C11orf53	chromosome 11 open reading frame 53	11	111126707	111157126	30420
C11orf63	chromosome 11 open reading frame 63	11	122753391	122830506	77116
C11orf70	chromosome 11 open reading frame 70	11	101918174	101955291	37118
C12orf29	chromosome 12 open reading frame 29	12	88427623	88443937	16315
C12orf42	chromosome 12 open reading frame 42	12	103631369	103889749	258381
C12orf56	chromosome 12 open reading frame 56	12	64660217	64784972	124756
C12orf65	chromosome 12 open reading frame 65	12	123717463	123742506	25044
C14orf105	chromosome 14 open reading frame 105	14	57936019	57960585	24567
C14orf159	chromosome 14 open reading frame 159	14	91526677	91691976	165300
**C14orf177**	chromosome 14 open reading frame 177	14	99177950	99184098	6149
C14orf37	chromosome 14 open reading frame 37	14	58466453	58764857	298405
C16orf52	chromosome 16 open reading frame 52	16	22018959	22098855	79897
C16orf62	chromosome 16 open reading frame 62	16	19566562	19718115	151554
C16orf89	chromosome 16 open reading frame 89	16	5094123	5116111	21989
C17orf53	chromosome 17 open reading frame 53	17	42219274	42239844	20571
C17orf97	chromosome 17 open reading frame 97	17	260118	273510	13393
C18orf63	chromosome 18 open reading frame 63	18	71983048	72026422	43375
C19orf12	chromosome 19 open reading frame 12	19	30191721	30206364	14644
C1orf167	chromosome 1 open reading frame 167	1	11821844	11849642	27799
C1orf21	chromosome 1 open reading frame 21	1	184356192	184598154	241963
C1orf228	chromosome 1 open reading frame 228	1	45140364	45191263	50900
C1orf87	chromosome 1 open reading frame 87	1	60452941	60539442	86502
C1QTNF1	C1q and TNF related 1	17	77018896	77045870	26975
C1QTNF1-AS1	C1QTNF1 antisense RNA 1	17	77015291	77023737	8447
C1R	complement C1r	12	7187513	7245203	57691
C1RL	complement C1r subcomponent like	12	7242183	7261869	19687
C2	complement C2	6	31865562	31913449	47888
C20orf194	chromosome 20 open reading frame 194	20	3229951	3388272	158322
**C21orf58**	chromosome 21 open reading frame 58	21	47720095	47743789	23695
C22orf15	chromosome 22 open reading frame 15	22	24105208	24108048	2841
C22orf34	chromosome 22 open reading frame 34	22	49808176	50051190	243015
C2CD3	C2 calcium dependent domain containing 3	11	73723763	73882255	158493
C2CD5	C2 calcium dependent domain containing 5	12	22601517	22697480	95964
C2orf16	chromosome 2 open reading frame 16	2	27799389	27805588	6200
C2orf76	chromosome 2 open reading frame 76	2	120059801	120124404	64604
C2orf78	chromosome 2 open reading frame 78	2	74011316	74044274	32959
C2orf88	chromosome 2 open reading frame 88	2	190744335	191068210	323876
C3orf20	chromosome 3 open reading frame 20	3	14716606	14814541	97936
C3orf33	chromosome 3 open reading frame 33	3	155480401	155524140	43740
C3orf62	chromosome 3 open reading frame 62	3	49306035	49315342	9308
C3orf67	chromosome 3 open reading frame 67	3	58703092	59035810	332719
C3orf70	chromosome 3 open reading frame 70	3	184795838	184870802	74965
C3P1	complement component 3 precursor pseudogene	19	10148479	10184811	36333
C4A	complement C4A (Rodgers blood group)	6	31949801	31970458	20658
C4orf22	chromosome 4 open reading frame 22	4	81256874	81884910	628037
C5orf15	chromosome 5 open reading frame 15	5	133291201	133304478	13278

C5orf42	chromosome 5 open reading frame 42	5	37106330	37249530	143201
C5orf64	chromosome 5 open reading frame 64	5	60933535	61047590	114056
C6	complement C6	5	41142336	41261540	119205
C6orf106	chromosome 6 open reading frame 106	6	34555065	34664636	109572
C6orf132	chromosome 6 open reading frame 132	6	42068856	42110357	41502
C6orf89	chromosome 6 open reading frame 89	6	36839646	36896740	57095
C8orf34	chromosome 8 open reading frame 34	8	69242957	69731257	488301
C9orf24	chromosome 9 open reading frame 24	9	34379017	34397830	18814
C9orf3	chromosome 9 open reading frame 3	9	97488983	97849441	360459
**C9orf72**	chromosome 9 open reading frame 72	9	27546544	27573864	27321
C9orf84	chromosome 9 open reading frame 84	9	114448453	114557288	108836
CA10	carbonic anhydrase 10	17	49707674	50237377	529704
CA12	carbonic anhydrase 12	15	63613577	63674360	60784
CA13	carbonic anhydrase 13	8	86132816	86196302	63487
CA2	carbonic anhydrase 2	8	86376081	86393722	17642
CA5A	carbonic anhydrase 5A	16	87921625	87970135	48511
CA8	carbonic anhydrase 8	8	61099906	61193971	94066
CAAP1	caspase activity and apoptosis inhibitor 1	9	26840683	26892802	52120
CAB39	calcium binding protein 39	2	231577560	231685790	108231
CAB39L	calcium binding protein 39 like	13	49882786	50018262	135477
CABIN1	calcineurin binding protein 1	22	24407642	24574596	166955
CABP7	calcium binding protein 7	22	30116073	30127828	11756
CACHD1	cache domain containing 1	1	64936428	65158741	222314
CACNA1A	calcium voltage-gated channel subunit alpha1 A	19	13317256	13734804	417549
CACNA1B	calcium voltage-gated channel subunit alpha1 B	9	140772241	141019076	246836
CACNA1C	calcium voltage-gated channel subunit alpha1 C	12	2079952	2802108	722157
CACNA1D	calcium voltage-gated channel subunit alpha1 D	3	53528683	53847760	319078
CACNA1E	calcium voltage-gated channel subunit alpha1 E	1	181382238	181777219	394982
**CACNA1H**	calcium voltage-gated channel subunit alpha1 H	16	1203241	1271771	68531
**CACNA2D1**	calcium voltage-gated channel auxiliary subunit alpha2delta 1	7	81575760	82073114	497355
CACNA2D3	calcium voltage-gated channel auxiliary subunit alpha2delta 3	3	54156574	55108584	952011
CACNB2	calcium voltage-gated channel auxiliary subunit beta 2	10	18429606	18830798	401193
CACNB4	calcium voltage-gated channel auxiliary subunit beta 4	2	152689290	152955593	266304
CACUL1	CDK2 associated cullin domain 1	10	120433679	120514761	81083
CADM1	cell adhesion molecule 1	11	115039938	115375675	335738
CADM2	cell adhesion molecule 2	3	85008132	86123579	1115448
CADPS	calcium dependent secretion activator	3	62384022	62861054	477033
CADPS2	calcium dependent secretion activator 2	7	121958481	122526813	568333
CALB1	calbindin 1	8	91070836	91107703	36868
CALCA	calcitonin related polypeptide alpha	11	14988214	14993900	5687
CALCRL	calcitonin receptor like receptor	2	188207856	188313187	105332
CALD1	caldesmon 1	7	134429003	134655479	226477
CALHM1	calcium homeostasis modulator 1	10	105213144	105218645	5502
**CALHM2**	calcium homeostasis modulator 2	10	105206543	105212660	6118
**CALHM3**	calcium homeostasis modulator 3	10	105232561	105238997	6437
CALML4	calmodulin like 4	15	68483043	68498417	15375
CALN1	calneuron 1	7	71244476	71912136	667661
CAMK1D	calcium/calmodulin dependent protein kinase ID	10	12391481	12877545	486065
CAMK2D	calcium/calmodulin dependent protein kinase II delta	4	114372188	114683083	310896
CAMK2G	calcium/calmodulin dependent protein kinase II gamma	10	75572259	75634343	62085
CAMK4	calcium/calmodulin dependent protein kinase IV	5	110559351	110830584	271234
CAMKMT	calmodulin-lysine N-methyltransferase	2	44589089	44999731	410643
CAMLG	calcium modulating ligand	5	134074191	134087847	13657
CAMSAP1	calmodulin regulated spectrin associated protein 1	9	138700333	138799074	98742
CAMTA1	calmodulin binding transcription activator 1	1	6845384	7829766	984383
CAPN11	calpain 11	6	44126548	44152139	25592
CAPN13	calpain 13	2	30945637	31043408	97772
CAPN2	calpain 2	1	223889295	223963720	74426
CAPN3	calpain 3	15	42640301	42704516	64216
CAPRIN1	cell cycle associated protein 1	11	34073230	34122703	49474
CAPRIN2	caprin family member 2	12	30862486	30907885	45400
CAPZA2	capping actin protein of muscle Z-line alpha subunit 2	7	116451124	116562103	110980
CARD11	caspase recruitment domain family member 11	7	2945775	3083579	137805
CARD8	caspase recruitment domain family member 8	19	48684027	48759203	75177
CASC11	cancer susceptibility 11 (non-protein coding)	8	128698588	128746213	47626
CASC15	cancer susceptibility 15 (non-protein coding)	6	21665003	22214734	549732
CASC2	cancer susceptibility 2 (non-protein coding)	10	119805790	119969663	163874
CASC6	cancer susceptibility 6 (non-protein coding)	6	92339633	92400146	60514
CASK	calcium/calmodulin dependent serine protein kinase	X	41374187	41782716	408530
CASP2	caspase 2	7	142985308	143004789	19482
CASP3	caspase 3	4	185548850	185570663	21814
CASP4	caspase 4	11	104813593	104840163	26571
CASP6	caspase 6	4	110609785	110624739	14955
CASP8	caspase 8	2	202098166	202152434	54269
CASP9	caspase 9	1	15817327	15853029	35703
CASQ2	calsequestrin 2	1	116242628	116311402	68775
CASR	calcium sensing receptor	3	121902530	122005342	102813

CASS4	Cas scaffolding protein family member 4	20	54987168	55034396	47229
CAST	calpastatin	5	95860971	96115299	254329
CASZ1	castor zinc finger 1	1	10696661	10856707	160047
CAT	catalase	11	34460472	34493609	33138
CAV1	caveolin 1	7	116164839	116201233	36395
CAV3	caveolin 3	3	8775486	8883492	108007
CBFA2T2	CBFA2/RUNX1 translocation partner 2	20	32077881	32237842	159962
CBFA2T3	CBFA2/RUNX1 translocation partner 3	16	88941266	89043612	102347
CBFB	core-binding factor beta subunit	16	67063019	67134961	71943
CBL	Cbl proto-oncogene	11	119076752	119178859	102108
CBS	cystathionine-beta-synthase	21	44473301	44497053	23753
CBWD5	COBW domain containing 5	9	70432004	70497240	65237
CBWD6	COBW domain containing 6	9	69204538	69269662	65125
CBX5	chromobox 5	12	54624724	54673886	49163
CCBE1	collagen and calcium binding EGF domains 1	18	57098172	57364612	266441
CCDC102B	coiled-coil domain containing 102B	18	66382446	66722426	339981
CCDC117	coiled-coil domain containing 117	22	29168662	29185283	16622
CCDC12	coiled-coil domain containing 12	3	46963216	47023500	60285
CCDC120	coiled-coil domain containing 120	X	48911101	48927509	16409
CCDC129	coiled-coil domain containing 129	7	31553704	31698334	144631
CCDC13	coiled-coil domain containing 13	3	42734155	42814745	80591
CCDC14	coiled-coil domain containing 14	3	123616152	123680564	64413
CCDC141	coiled-coil domain containing 141	2	179694484	179914813	220330
CCDC144A	coiled-coil domain containing 144A	17	16592851	16707767	114917
CCDC148	coiled-coil domain containing 148	2	159027593	159313265	285673
CCDC148-AS1	CCDC148 antisense RNA 1	2	159023162	159092681	69520
CCDC149	coiled-coil domain containing 149	4	24807739	24981826	174088
CCDC152	coiled-coil domain containing 152	5	42756903	42802462	45560
CCDC158	coiled-coil domain containing 158	4	77234154	77343021	108868
CCDC167	coiled-coil domain containing 167	6	37450696	37467698	17003
CCDC169-SOHLH2	CCDC169-SOHLH2 readthrough	13	36742931	36871979	129049
CCDC171	coiled-coil domain containing 171	9	15552895	16061661	508767
CCDC178	coiled-coil domain containing 178	18	30517366	31021065	503700
CCDC18	coiled-coil domain containing 18	1	93645476	93744287	98812
CCDC25	coiled-coil domain containing 25	8	27590835	27630170	39336
CCDC26	CCDC26 long non-coding RNA	8	130363937	130692485	328549
CCDC3	coiled-coil domain containing 3	10	12938627	13141652	203026
CCDC59	coiled-coil domain containing 59	12	82617460	82752584	135125
CCDC6	coiled-coil domain containing 6	10	61548521	61666414	117894
CCDC7	coiled-coil domain containing 7	10	32735068	32863492	128425
**CCDC82**	coiled-coil domain containing 82	11	96085933	96123087	37155
CCDC85A	coiled-coil domain containing 85A	2	56411258	56613308	202051
CCDC92	coiled-coil domain containing 92	12	124403207	124457378	54172
CCK	cholecystokinin	3	42299317	42307699	8383
CCKAR	cholecystokinin A receptor	4	26483022	26492084	9063
CCKBR	cholecystokinin B receptor	11	6280966	6293357	12392
CCL2	C-C motif chemokine ligand 2	17	32582304	32584222	1919
CCL3	C-C motif chemokine ligand 3	17	34415602	34417515	1914
CCL5	C-C motif chemokine ligand 5	17	34198495	34207797	9303
CCL8	C-C motif chemokine ligand 8	17	32646055	32648421	2367
**CCNA2**	cyclin A2	4	122737599	122745087	7489
CCNB1IP1	cyclin B1 interacting protein 1	14	20779527	20801471	21945
CCNB2	cyclin B2	15	59397277	59417244	19968
CCNH	cyclin H	5	86687311	86708836	21526
CCNT2-AS1	CCNT2 antisense RNA 1	2	135493034	135676280	183247
CCNY	cyclin Y	10	35535953	35860852	324900
CCR2	C-C motif chemokine receptor 2	3	46395225	46402419	7195
CCR3	C-C motif chemokine receptor 3	3	46205096	46308197	103102
**CCS**	copper chaperone for superoxide dismutase	11	66360292	66373490	13199
CCSER1	coiled-coil serine rich protein 1	4	91048686	92523064	1474379
CCT4	chaperonin containing TCP1 subunit 4	2	62095224	62115939	20716
CCT5	chaperonin containing TCP1 subunit 5	5	10250033	10266524	16492
CD109	CD109 molecule	6	74405508	74538040	132533
CD14	CD14 molecule	5	140011313	140013286	1974
CD22	CD22 molecule	19	35810164	35838258	28095
CD24	CD24 molecule	6	107417708	107422630	4923
CD274	CD274 molecule	9	5450503	5470566	20064
CD300LB	CD300 molecule like family member b	17	72517313	72527613	10301
CD33	CD33 molecule	19	51728320	51747115	18796
CD36	CD36 molecule	7	79998891	80308593	309703
CD3G	CD3g molecule	11	118215059	118225876	10818
CD40	CD40 molecule	20	44746911	44758502	11592
CD44	CD44 molecule (Indian blood group)	11	35160417	35253949	93533
CD86	CD86 molecule	3	121774213	121839983	65771
CD96	CD96 molecule	3	111011566	111384597	373032
CDA	cytidine deaminase	1	20915441	20945401	29961
CDC14A	cell division cycle 14A	1	100810584	100985833	175250

CDC20	cell division cycle 20	1	43824626	43828874	4249
CDC25A	cell division cycle 25A	3	48198636	48229892	31257
CDC25B	cell division cycle 25B	20	3767578	3786762	19185
CDC25C	cell division cycle 25C	5	137620954	137674044	53091
CDC37L1	cell division cycle 37 like 1	9	4679559	4708398	28840
CDC42	cell division cycle 42	1	22379120	22419437	40318
CDC42BPA	CDC42 binding protein kinase alpha	1	227177566	227506175	328610
CDC42BPB	CDC42 binding protein kinase beta	14	103398716	103523799	125084
CDC42SE2	CDC42 small effector 2	5	130581186	130734140	152955
CDC6	cell division cycle 6	17	38443885	38459171	15287
**CDCA2**	cell division cycle associated 2	8	25316513	25365436	48924
CDCP1	CUB domain containing protein 1	3	45123770	45187914	64145
CDCP2	CUB domain containing protein 2	1	54598747	54619443	20697
CDH1	cadherin 1	16	68771128	68869451	98324
CDH12	cadherin 12	5	21750782	22853731	1102950
**CDH13**	cadherin 13	16	82660408	83830204	1169797
CDH17	cadherin 17	8	95139399	95229531	90133
CDH18	cadherin 18	5	19473060	20575982	1102923
CDH2	cadherin 2	18	25530930	25757410	226481
**CDH22**	cadherin 22	20	44802372	44937137	134766
CDH23	cadherin related 23	10	73156691	73575702	419012
CDH3	cadherin 3	16	68670092	68756519	86428
CDH4	cadherin 4	20	59827482	60515673	688192
CDH6	cadherin 6	5	31193857	31329253	135397
CDH8	cadherin 8	16	61681146	62070939	389794
CDH9	cadherin 9	5	26880709	27121257	240549
CDK1	cyclin dependent kinase 1	10	62538089	62554610	16522
CDK13	cyclin dependent kinase 13	7	39989636	40136733	147098
CDK14	cyclin dependent kinase 14	7	90095738	90839905	744168
CDK15	cyclin dependent kinase 15	2	202655184	202760273	105090
CDK16	cyclin dependent kinase 16	X	47077259	47089396	12138
CDK5	cyclin dependent kinase 5	7	150750899	150755617	4719
CDK5R1	cyclin dependent kinase 5 regulatory subunit 1	17	30813637	30818274	4638
CDK6	cyclin dependent kinase 6	7	92234235	92465908	231674
CDKAL1	CDK5 regulatory subunit associated protein 1 like 1	6	20534688	21232635	697948
CDKL5	cyclin dependent kinase like 5	X	18443703	18671749	228047
CDKN2A	cyclin dependent kinase inhibitor 2A	9	21967751	21995300	27550
CDKN3	cyclin dependent kinase inhibitor 3	14	54863567	54886936	23370
CDON	cell adhesion associated, oncogene regulated	11	125825691	125933230	107540
CDS1	CDP-diacylglycerol synthase 1	4	85504132	85572491	68360
CDYL	chromodomain Y like	6	4706393	4955785	249393
CEACAM21	carcinoembryonic antigen related cell adhesion molecule 21	19	42055886	42093197	37312
CEBPA-AS1	CEBPA antisense RNA 1 (head to head)	19	33793763	33795960	2198
CELF1	CUGBP Elav-like family member 1	11	47487496	47587121	99626
CELF2	CUGBP Elav-like family member 2	10	11047259	11378674	331416
CENPE	centromere protein E	4	104026963	104119566	92604
CENPF	centromere protein F	1	214776538	214837931	61394
CENPP	centromere protein P	9	95087766	95382815	295050
CEP104	centrosomal protein 104	1	3728645	3773778	45134
CEP112	centrosomal protein 112	17	63631656	64188202	556547
CEP120	centrosomal protein 120	5	122680579	122759286	78708
CEP128	centrosomal protein 128	14	80943330	81425861	482532
CEP135	centrosomal protein 135	4	56815037	56899529	84493
CEP152	centrosomal protein 152	15	49005125	49103343	98219
CEP164	centrosomal protein 164	11	117185273	117283984	98712
CEP170	centrosomal protein 170	1	243287730	243418650	130921
CEP192	centrosomal protein 192	18	12991361	13125051	133691
CEP290	centrosomal protein 290	12	88442793	88535993	93201
CEP350	centrosomal protein 350	1	179923873	180084015	160143
CEP41	centrosomal protein 41	7	130033612	130082274	48663
CEP63	centrosomal protein 63	3	134204585	134293859	89275
CEP70	centrosomal protein 70	3	138213186	138313380	100195
CEP85L	centrosomal protein 85 like	6	118781935	119031238	249304
CEP89	centrosomal protein 89	19	33369902	33462897	92996
CEPT1	choline/ethanolamine phosphotransferase 1	1	111682249	111727724	45476
CERS3	ceramide synthase 3	15	100940600	101085200	144601
CERS6	ceramide synthase 6	2	169312372	169631644	319273
CETN4P	centrin 4, pseudogene	4	123651703	123653348	1646
CETP	cholesteryl ester transfer protein	16	56995762	57017757	21996
CFB	complement factor B	6	31895475	31919861	24387
CFH	complement factor H	1	196621008	196716634	95627
CFHR4	complement factor H related 4	1	196819371	196888102	68732
CFLAR	CASP8 and FADD like apoptosis regulator	2	201980827	202041410	60584
CFTR	cystic fibrosis transmembrane conductance regulator	7	117105838	117356025	250188
CGNL1	cingulin like 1	15	57668165	57842925	174761
CH25H	cholesterol 25-hydroxylase	10	90965694	90967071	1378
CHAF1A	chromatin assembly factor 1 subunit A	19	4402659	4445015	42357

CHAT	choline O-acetyltransferase	10	50817141	50901925	84785
**CHCHD10**	coiled-coil-helix-coiled-coil-helix domain containing 10	22	24108021	24110630	2610
CHCHD3	coiled-coil-helix-coiled-coil-helix domain containing 3	7	132469629	132766848	297220
**CHCHD6**	coiled-coil-helix-coiled-coil-helix domain containing 6	3	126423063	126679249	256187
CHD2	chromodomain helicase DNA binding protein 2	15	93426526	93571237	144712
CHD5	chromodomain helicase DNA binding protein 5	1	6161853	6240183	78331
CHD6	chromodomain helicase DNA binding protein 6	20	40030741	40247133	216393
CHD7	chromodomain helicase DNA binding protein 7	8	61591337	61779465	188129
CHD8	chromodomain helicase DNA binding protein 8	14	21853353	21924285	70933
CHD9	chromodomain helicase DNA binding protein 9	16	53088945	53363062	274118
CHFR	checkpoint with forkhead and ring finger domains	12	133398773	133532890	134118
**CHGB**	chromogranin B	20	5892076	5906007	13932
CHIC2	cysteine rich hydrophobic domain 2	4	54875956	54930857	54902
CHL1	cell adhesion molecule L1 like	3	238279	451090	212812
CHM	CHM, Rab escort protein 1	X	85116185	85302566	186382
CHMP1B2P	charged multivesicular body protein 1B2, pseudogene	X	79528830	79565669	36840
**CHMP2B**	charged multivesicular body protein 2B	3	87276421	87304698	28278
CHMP4B	charged multivesicular body protein 4B	20	32399110	32442172	43063
CHN1	chimerin 1	2	175664091	175870097	206007
CHODL	chondrolectin	21	19273580	19639690	366111
CHORDC1	cysteine and histidine rich domain containing 1	11	89934328	89956532	22205
CHP1	calcineurin like EF-hand protein 1	15	41523037	41574043	51007
CHPT1	choline phosphotransferase 1	12	102090725	102137918	47194
CHRDL1	chordin like 1	X	109917084	110039286	122203
CHRDL2	chordin like 2	11	74407474	74442430	34957
CHRM1	cholinergic receptor muscarinic 1	11	62676151	62689279	13129
CHRM2	cholinergic receptor muscarinic 2	7	136553416	136705002	151587
CHRM3	cholinergic receptor muscarinic 3	1	239549865	240078750	528886
CHRNA2	cholinergic receptor nicotinic alpha 2 subunit	8	27317279	27337400	20122
CHRNA3	cholinergic receptor nicotinic alpha 3 subunit	15	78885394	78913637	28244
CHRNA4	cholinergic receptor nicotinic alpha 4 subunit	20	61975420	62009753	34334
CHRNA5	cholinergic receptor nicotinic alpha 5 subunit	15	78857862	78887611	29750
CHRNA7	cholinergic receptor nicotinic alpha 7 subunit	15	32322691	32464722	142032
CHRNB2	cholinergic receptor nicotinic beta 2 subunit	1	154540257	154552502	12246
CHST11	carbohydrate sulfotransferase 11	12	104849073	105155792	306720
CHST15	carbohydrate sulfotransferase 15	10	125767184	125853206	86023
CHST8	carbohydrate sulfotransferase 8	19	34112861	34264414	151554
CHST9	carbohydrate sulfotransferase 9	18	24495595	24765281	269687
CHSY1	chondroitin sulfate synthase 1	15	101715928	101792137	76210
CIAPIN1	cytokine induced apoptosis inhibitor 1	16	57462081	57481440	19360
CIDEA	cell death-inducing DFFA-like effector a	18	12254318	12277594	23277
CIRBP	cold inducible RNA binding protein	19	1259384	1274879	15496
CISH	cytokine inducible SH2 containing protein	3	50643921	50649262	5342
CIZ1	CDKN1A interacting zinc finger protein 1	9	130928343	130966662	38320
CKAP5	cytoskeleton associated protein 5	11	46764598	46867847	103250
CKS1B	CDC28 protein kinase regulatory subunit 1B	1	154947129	154951725	4597
CLASP1	cytoplasmic linker associated protein 1	2	122095352	122407163	311812
CLASP2	cytoplasmic linker associated protein 2	3	33537737	33759848	222112
CLCA3P	chloride channel accessory 3, pseudogene	1	87099956	87121059	21104
CLCC1	chloride channel CLIC like 1	1	109472130	109506111	33982
CLCN2	chloride voltage-gated channel 2	3	184063973	184079439	15467
CLCN6	chloride voltage-gated channel 6	1	11866207	11903201	36995
CLCNKA	chloride voltage-gated channel Ka	1	16345370	16360545	15176
CLCNKB	chloride voltage-gated channel Kb	1	16370272	16383803	13532
CLDN14	claudin 14	21	37832919	37948867	115949
CLEC5A	C-type lectin domain containing 5A	7	141627157	141646807	19651
CLIC5	chloride intracellular channel 5	6	45868045	46048132	180088
CLINT1	clathrin interactor 1	5	157212751	157286183	73433
CLIP1	CAP-Gly domain containing linker protein 1	12	122755979	122907179	151201
CLIP4	CAP-Gly domain containing linker protein family member 4	2	29320571	29412509	91939
CLK2	CDC like kinase 2	1	155232659	155248282	15624
CLMP	CXADR like membrane protein	11	122943035	123065989	122955
CLN3	CLN3, battenin	16	28477983	28506896	28914
CLN5	ceroid-lipofuscinosis, neuronal 5	13	77564795	77576652	11858
CLN6	ceroid-lipofuscinosis, neuronal 6, late infantile, variant	15	68499330	68549549	50220
CLN8	CLN8, transmembrane ER and ERGIC protein	8	1703944	1734738	30795
CLOCK	clock circadian regulator	4	56294070	56413305	119236
CLPB	ClpB homolog, mitochondrial AAA ATPase chaperonin	11	72003469	72145692	142224
CLPTM1	CLPTM1, transmembrane protein	19	45457842	45496599	38758
CLRN1-AS1	CLRN1 antisense RNA 1	3	150570271	150798513	228243
CLSTN2	calsyntenin 2	3	139654027	140296239	642213
CLTCL1	clathrin heavy chain like 1	22	19166986	19279239	112254
CLU	clusterin	8	27454434	27472548	18115
CLUL1	clusterin like 1	18	596988	650334	53347
CLVS1	clavesin 1	8	61969717	62414204	444488
CLVS2	clavesin 2	6	123317116	123394072	76957
CLYBL	citrate lyase beta like	13	100258919	100549387	290469

CMC2	C-X9-C motif containing 2	16	81009698	81053875	44178
CMIP	c-Maf inducing protein	16	81478775	81745367	266593
CMPK2	cytidine/uridine monophosphate kinase 2	2	6980701	7006766	26066
CMTM4	CKLF like MARVEL transmembrane domain containing 4	16	66648653	66730610	81958
CNBD1	cyclic nucleotide binding domain containing 1	8	87878670	88627447	748778
CNDP1	carnosine dipeptidase 1	18	72201675	72254448	52774
CNGB3	cyclic nucleotide gated channel beta 3	8	87566205	87755903	189699
CNIH1	cornichon family AMPA receptor auxiliary protein 1	14	54893654	54908149	14496
CNKSR2	connector enhancer of kinase suppressor of Ras 2	X	21392536	21672813	280278
CNNM2	cyclin and CBS domain divalent metal cation transport mediator 2	10	104678050	104849978	171929
CNOT1	CCR4-NOT transcription complex subunit 1	16	58553855	58663790	109936
CNOT10	CCR4-NOT transcription complex subunit 10	3	32726637	32815367	88731
CNOT4	CCR4-NOT transcription complex subunit 4	7	135046547	135194875	148329
CNOT6	CCR4-NOT transcription complex subunit 6	5	179921412	180005405	83994
CNOT6L	CCR4-NOT transcription complex subunit 6 like	4	78634541	78740769	106229
CNR1	cannabinoid receptor 1	6	88849583	88876078	26496
CNR2	cannabinoid receptor 2	1	24197016	24285549	88534
CNTLN	centlein	9	17134980	17503921	368942
CNTN2	contactin 2	1	205012325	205047627	35303
CNTN3	contactin 3	3	74311719	74570291	258573
**CNTN4**	contactin 4	3	2140497	3099645	959149
CNTN5	contactin 5	11	98891683	100229616	1337934
**CNTN6**	contactin 6	3	1134260	1445901	311642
CNTNAP2	contactin associated protein-like 2	7	145813453	148118090	2304638
CNTNAP3	contactin associated protein-like 3	9	39072764	39288312	215549
CNTNAP4	contactin associated protein like 4	16	76311176	76593135	281960
CNTNAP5	contactin associated protein like 5	2	124782864	125672864	890001
CNTRL	centriolin	9	123837141	123939888	102748
COBL	cordon-bleu WH2 repeat protein	7	51083909	51384515	300607
COCH	cochlin	14	31343720	31364271	20552
COG1	component of oligomeric golgi complex 1	17	71189129	71204646	15518
COG2	component of oligomeric golgi complex 2	1	230778235	230829728	51494
COG4	component of oligomeric golgi complex 4	16	70514471	70557468	42998
COG5	component of oligomeric golgi complex 5	7	106842000	107204959	362960
COG6	component of oligomeric golgi complex 6	13	40229764	40365802	136039
COG7	component of oligomeric golgi complex 7	16	23399814	23464501	64688
COG8	component of oligomeric golgi complex 8	16	69354043	69373570	19528
COL11A1	collagen type XI alpha 1 chain	1	103342023	103574052	232030
COL11A2	collagen type XI alpha 2 chain	6	33130458	33160276	29819
COL14A1	collagen type XIV alpha 1 chain	8	121072019	121384275	312257
COL16A1	collagen type XVI alpha 1 chain	1	32117848	32169920	52073
COL18A1	collagen type XVIII alpha 1 chain	21	46825052	46933634	108583
COL19A1	collagen type XIX alpha 1 chain	6	70576463	70919679	343217
COL21A1	collagen type XXI alpha 1 chain	6	55921388	56258892	337505
COL22A1	collagen type XXII alpha 1 chain	8	139600478	139926249	325772
COL23A1	collagen type XXIII alpha 1 chain	5	177664619	178017556	352938
COL24A1	collagen type XXIV alpha 1 chain	1	86194916	86622626	427711
COL25A1	collagen type XXV alpha 1 chain	4	109731877	110223813	491937
COL26A1	collagen type XXVI alpha 1 chain	7	101006101	101202304	196204
COL27A1	collagen type XXVII alpha 1 chain	9	116917840	117074791	156952
COL28A1	collagen type XXVIII alpha 1 chain	7	7395834	7575484	179651
COL4A1	collagen type IV alpha 1 chain	13	110801318	110959496	158179
COL4A2	collagen type IV alpha 2 chain	13	110958159	111165374	207216
COL4A4	collagen type IV alpha 4 chain	2	227867427	228028829	161403
COL4A6	collagen type IV alpha 6 chain	X	107386780	107682727	295948
COL6A3	collagen type VI alpha 3 chain	2	238232646	238323018	90373
COL6A5	collagen type VI alpha 5 chain	3	130064359	130203688	139330
COL6A6	collagen type VI alpha 6 chain	3	130279178	130396999	117822
COL9A1	collagen type IX alpha 1 chain	6	70924764	71012786	88023
**COLEC12**	collectin subfamily member 12	18	319361	500722	181362
COLGALT2	collagen beta(1-O)galactosyltransferase 2	1	183898796	184006863	108068
COMMD4	COMM domain containing 4	15	75628232	75634268	6037
COMT	catechol-O-methyltransferase	22	19929130	19957498	28369
COPA	coatomer protein complex subunit alpha	1	160259063	160313190	54128
COPB2	coatomer protein complex subunit beta 2	3	139074442	139108574	34133
COPS7B	COP9 signalosome subunit 7B	2	232646381	232673963	27583
COQ2	coenzyme Q2, polyprenyltransferase	4	84182689	84206067	23379
COQ6	coenzyme Q6, monooxygenase	14	74416629	74430373	13745
COQ9	coenzyme Q9	16	57481337	57495187	13851
CORIN	corin, serine peptidase	4	47596015	47840123	244109
CORO1C	coronin 1C	12	109038885	109125372	86488
COX10	COX10, heme A:farnesyltransferase cytochrome c oxidase assembly factor	17	13972813	14111994	139182
COX10-AS1	COX10 antisense RNA 1	17	13659795	13972812	313018
COX15	COX15, cytochrome c oxidase assembly homolog	10	101471601	101491857	20257
COX16	COX16, cytochrome c oxidase assembly homolog	14	70791798	70826448	34651
COX7B2	cytochrome c oxidase subunit 7B2	4	46736844	46911252	174409

CP	ceruloplasmin	3	148880197	148939842	59646
CPA6	carboxypeptidase A6	8	68334360	68658620	324261
CPAMD8	C3 and PZP like, alpha-2-macroglobulin domain containing 8	19	17003758	17137625	133868
CPD	carboxypeptidase D	17	28705923	28797007	91085
CPE	carboxypeptidase E	4	166282346	166419472	137127
CPEB1	cytoplasmic polyadenylation element binding protein 1	15	83211951	83317612	105662
CPEB3	cytoplasmic polyadenylation element binding protein 3	10	93806449	94050844	244396
CPEB4	cytoplasmic polyadenylation element binding protein 4	5	173315283	173388979	73697
CPED1	cadherin like and PC-esterase domain containing 1	7	120628731	120937498	308768
CPNE4	copine 4	3	131252399	132004254	751856
CPPED1	calcineurin like phosphoesterase domain containing 1	16	12756919	12897874	140956
CPQ	carboxypeptidase Q	8	97657455	98161882	504428
CPS1	carbamoyl-phosphate synthase 1	2	211342406	211543831	201426
CPSF6	cleavage and polyadenylation specific factor 6	12	69633317	69668138	34822
CPT2	carnitine palmitoyltransferase 2	1	53662101	53679869	17769
CPVL	carboxypeptidase, vitellogenic like	7	29034847	29235067	200221
CPXM2	carboxypeptidase X, M14 family member 2	10	125465723	125699783	234061
**CPZ**	carboxypeptidase Z	4	8594387	8621488	27102
CR1	complement C3b/C4b receptor 1 (Knops blood group)	1	207669492	207813992	144501
CR2	complement C3d receptor 2	1	207627575	207663240	35666
CRADD	CASP2 and RIPK1 domain containing adaptor with death domain	12	94071151	94288616	217466
CREB1	cAMP responsive element binding protein 1	2	208394461	208468155	73695
CREB5	cAMP responsive element binding protein 5	7	28338940	28865511	526572
CREBBP	CREB binding protein	16	3775055	3930727	155673
CRH	corticotropin releasing hormone	8	67088620	67090960	2341
**CRIM1**	cysteine rich transmembrane BMP regulator 1	2	36583069	36778278	195210
**CRISPLD1**	cysteine rich secretory protein LCCL domain containing 1	8	75896750	75946793	50044
CRLF3	cytokine receptor like factor 3	17	29096406	29151794	55389
CRMP1	collapsin response mediator protein 1	4	5749811	5894785	144975
CRP	C-reactive protein	1	159682079	159684379	2301
CRTC3	CREB regulated transcription coactivator 3	15	91073157	91188577	115421
CRY2	cryptochrome circadian clock 2	11	45868669	45904798	36130
CRYL1	crystallin lambda 1	13	20977806	21099996	122191
**CRYM**	crystallin mu	16	21250195	21314404	64210
CSF1	colony stimulating factor 1	1	110452864	110473614	20751
CSGALNACT2	chondroitin sulfate N-acetylgalactosaminyltransferase 2	10	43633934	43680756	46823
CSK	CSK, non-receptor tyrosine kinase	15	75074398	75095539	21142
**CSMD1**	CUB and Sushi multiple domains 1	8	2792875	4852494	2059620
CSMD2	CUB and Sushi multiple domains 2	1	33979609	34631443	651835
CSMD3	CUB and Sushi multiple domains 3	8	113235157	114449328	1214172
CSNK1A1	casein kinase 1 alpha 1	5	148871760	148931007	59248
CSNK1D	casein kinase 1 delta	17	80196899	80231607	34709
CSNK1G1	casein kinase 1 gamma 1	15	64457716	64648442	190727
**CSNK1G3**	casein kinase 1 gamma 3	5	122847793	122952739	104947
CSNK2A1	casein kinase 2 alpha 1	20	459116	524465	65350
CSNK2A2	casein kinase 2 alpha 2	16	58191811	58231824	40014
CSRP1	cysteine and glycine rich protein 1	1	201452658	201478584	25927
**CST3**	cystatin C	20	23608534	23619110	10577
CSTB	cystatin B	21	45192393	45196326	3934
CSTF3	cleavage stimulation factor subunit 3	11	33098734	33183917	85184
CTAGE5	CTAGE family member 5, ER export factor	14	39734488	39856156	121669
CTBP2	C-terminal binding protein 2	10	126676421	126849739	173319
CTCF	CCCTC-binding factor	16	67596310	67673086	76777
CTCFL	CCCTC-binding factor like	20	56071035	56100708	29674
CTDP1	CTD phosphatase subunit 1	18	77439801	77514510	74710
CTDSPL2	CTD small phosphatase like 2	15	44719432	44821236	101805
CTNNA1	catenin alpha 1	5	137946656	138270723	324068
CTNNA2	catenin alpha 2	2	79412357	80875905	1463549
CTNNA3	catenin alpha 3	10	67672276	69455927	1783652
CTNND2	catenin delta 2	5	10971952	11904155	932204
CTPS1	CTP synthase 1	1	41445007	41478235	33229
CTPS2	CTP synthase 2	X	16606126	16731059	124934
CTSB	cathepsin B	8	11700033	11726957	26925
CTSD	cathepsin D	11	1773982	1785222	11241
CTSF	cathepsin F	11	66330934	66336312	5379
CTSS	cathepsin S	1	150702672	150738433	35762
CTTNBP2NL	CTTNBP2 N-terminal like	1	112938803	113006078	67276
CUBN	cubilin	10	16865963	17171830	305868
CUEDC1	CUE domain containing 1	17	55938604	56032684	94081
CUL2	cullin 2	10	35297479	35379570	82092
CUL3	cullin 3	2	225334867	225450110	115244
CUL4B	cullin 4B	X	119658464	119709649	51186
CUL5	cullin 5	11	107879459	107978503	99045
CUX1	cut like homeobox 1	7	101458959	101927249	468291
CUX2	cut like homeobox 2	12	111471828	111788358	316531
CWC25	CWC25 spliceosome associated protein homolog	17	36956687	36981734	25048
CWC27	CWC27 spliceosome associated protein homolog	5	64064757	64314590	249834

CWF19L1	CWF19 like 1, cell cycle control (S. pombe)	10	101992055	102027437	35383
CWF19L2	CWF19 like 2, cell cycle control (S. pombe)	11	107197071	107328572	131502
CWH43	cell wall biogenesis 43 C-terminal homolog	4	48988264	49064098	75835
**CX3CR1**	C-X3-C motif chemokine receptor 1	3	39304985	39323226	18242
CXCL12	C-X-C motif chemokine ligand 12	10	44793038	44881941	88904
CXCL8	C-X-C motif chemokine ligand 8	4	74606223	74609433	3211
CXCL9	C-X-C motif chemokine ligand 9	4	76922428	76928641	6214
CXorf56	chromosome X open reading frame 56	X	118672112	118699397	27286
CXorf57	chromosome X open reading frame 57	X	105855160	105922672	67513
CYB561A3	cytochrome b561 family member A3	11	61116217	61129771	13555
CYB5RL	cytochrome b5 reductase like	1	54638009	54665709	27701
CYFIP2	cytoplasmic FMR1 interacting protein 2	5	156693089	156822606	129518
CYLC2	cylicin 2	9	105757593	105780770	23178
CYP17A1	cytochrome P450 family 17 subfamily A member 1	10	104590288	104597290	7003
CYP19A1	cytochrome P450 family 19 subfamily A member 1	15	51500254	51630807	130554
CYP1A1	cytochrome P450 family 1 subfamily A member 1	15	75011883	75017951	6069
CYP1A2	cytochrome P450 family 1 subfamily A member 2	15	75041185	75048543	7359
CYP1B1	cytochrome P450 family 1 subfamily B member 1	2	38294116	38337044	42929
CYP20A1	cytochrome P450 family 20 subfamily A member 1	2	204103663	204163009	59347
CYP2C19	cytochrome P450 family 2 subfamily C member 19	10	96447911	96613017	165107
**CYP2D6**	cytochrome P450 family 2 subfamily D member 6	22	42522501	42526908	4408
CYP2E1	cytochrome P450 family 2 subfamily E member 1	10	135333910	135374724	40815
CYP2R1	cytochrome P450 family 2 subfamily R member 1	11	14899553	14913798	14246
CYP2U1	cytochrome P450 family 2 subfamily U member 1	4	108852525	108874613	22089
CYP3A43	cytochrome P450 family 3 subfamily A member 43	7	99425636	99463718	38083
CYP46A1	cytochrome P450 family 46 subfamily A member 1	14	100150641	100193638	42998
CYP51A1	cytochrome P450 family 51 subfamily A member 1	7	91741465	91772266	30802
CYP7B1	cytochrome P450 family 7 subfamily B member 1	8	65500320	65711318	210999
CYSTM1	cysteine rich transmembrane module containing 1	5	139554227	139661637	107411
CYTH1	cytohesin 1	17	76670130	76778379	108250
CYTH3	cytohesin 3	7	6201407	6312275	110869
CYTIP	cytohesin 1 interacting protein	2	158271131	158345473	74343
CYYR1	cysteine and tyrosine rich 1	21	27838528	27945603	107076
DAAM1	dishevelled associated activator of morphogenesis 1	14	59655364	59838123	182760
DAAM2	dishevelled associated activator of morphogenesis 2	6	39760142	39872648	112507
DAB1	DAB1, reelin adaptor protein	1	57460451	59012406	1551956
DAB2IP	DAB2 interacting protein	9	124329336	124547809	218474
DACH1	dachshund family transcription factor 1	13	72012098	72441330	429233
DACH2	dachshund family transcription factor 2	X	85403462	86087607	684146
DAG1	dystroglycan 1	3	49506146	49573048	66903
**DAO**	D-amino acid oxidase	12	109252708	109294819	42112
DAPK1	death associated protein kinase 1	9	90112143	90323548	211406
DAPK2	death associated protein kinase 2	15	64199235	64364232	164998
DARS	aspartyl-tRNA synthetase	2	136664247	136743670	79424
DARS2	aspartyl-tRNA synthetase 2, mitochondrial	1	173793641	173827684	34044
DAW1	dynein assembly factor with WD repeats 1	2	228735770	228789060	53291
DAZ1	deleted in azoospermia 1	Y	25275502	25345241	69740
**DBH**	dopamine beta-hydroxylase	9	136501482	136524466	22985
DCAF12	DDB1 and CUL4 associated factor 12	9	34086385	34127397	41013
DCAF4	DDB1 and CUL4 associated factor 4	14	73393040	73426411	33372
DCC	DCC netrin 1 receptor	18	49866542	51057784	1191243
DCDC2	doublecortin domain containing 2	6	24171984	24358280	186297
DCHS2	dachsous cadherin-related 2	4	155153399	155412930	259532
DCN	decorin	12	91539025	91576900	37876
DCP1A	decapping mRNA 1A	3	53317447	53381654	64208
DCT	dopachrome tautomerase	13	95089558	95131936	42379
**DCTN1**	dynactin subunit 1	2	74588281	74619214	30934
DCUN1D1	defective in cullin neddylation 1 domain containing 1	3	182655862	182703741	47880
DCUN1D3	defective in cullin neddylation 1 domain containing 3	16	20866247	20911706	45460
DCX	doublecortin	X	110537007	110655603	118597
DDAH1	dimethylarginine dimethylaminohydrolase 1	1	85784164	86043933	259770
DDC	dopa decarboxylase	7	50526134	50633154	107021
DDHD1	DDHD domain containing 1	14	53510686	53620000	109315
DDHD2	DDHD domain containing 2	8	38082736	38133076	50341
DDO	D-aspartate oxidase	6	110712974	110736765	23792
DDOST	dolichyl-diphosphooligosaccharide--protein glycosyltransferase non-catalytic subunit	1	20978270	20988000	9731
DDX10	DEAD-box helicase 10	11	108535752	108811657	275906
DDX21	DExD-box helicase 21	10	70715884	70744829	28946
**DDX31**	DEAD-box helicase 31	9	135468384	135545788	77405
DDX39B	DExD-box helicase 39B	6	31497996	31510225	12230
DDX3X	DEAD-box helicase 3, X-linked	X	41192651	41223725	31075
DDX5	DEAD-box helicase 5	17	62495734	62504317	8584
DDX6	DEAD-box helicase 6	11	118620034	118661858	41825
DDX60	DExD/H-box helicase 60	4	169137444	169239958	102515
DDX60L	DEAD-box helicase 60 like	4	169277886	169458937	181052
**DEAF1**	DEAF1, transcription factor	11	644233	706715	62483

DECR1	2,4-dienoyl-CoA reductase 1	8	91013633	91064320	50688
DEF6	DEF6, guanine nucleotide exchange factor	6	35265595	35289548	23954
DEFB132	defensin beta 132	20	238377	241737	3361
DENND1A	DENN domain containing 1A	9	126141933	126692431	550499
DENND1B	DENN domain containing 1B	1	197473878	197744826	270949
DENND2A	DENN domain containing 2A	7	140218220	140373793	155574
DENND2C	DENN domain containing 2C	1	115125469	115213043	87575
DENND3	DENN domain containing 3	8	142127377	142205907	78531
DENND4A	DENN domain containing 4A	15	65950384	66084631	134248
DENND4C	DENN domain containing 4C	9	19230433	19374139	143707
DENND5A	DENN domain containing 5A	11	9160372	9286937	126566
DENND5B-AS1	DENND5B antisense RNA 1	12	31742857	31768600	25744
DENND6A	DENN domain containing 6A	3	57611184	57678816	67633
**DEPDC1B**	DEP domain containing 1B	5	59892739	59996017	103279
DEPDC5	DEP domain containing 5	22	32149944	32303012	153069
**DERA**	deoxyribose-phosphate aldolase	12	16064106	16190220	126115
DERL1	derlin 1	8	124025404	124054663	29260
DES	desmin	2	220283099	220291461	8363
DET1	de-etiolated homolog 1 (Arabidopsis)	15	89054790	89089906	35117
DGCR8	DGCR8, microprocessor complex subunit	22	20067755	20099400	31646
DGKB	diacylglycerol kinase beta	7	14184674	15014402	829729
DGKG	diacylglycerol kinase gamma	3	185823457	186080026	256570
**DGKI**	diacylglycerol kinase iota	7	137065783	137531838	466056
DGKQ	diacylglycerol kinase theta	4	952675	980683	28009
DHCR24	24-dehydrocholesterol reductase	1	55315306	55352891	37586
DHDDS	dehydrodolichyl diphosphate synthase subunit	1	26758773	26797785	39013
DHRS11	dehydrogenase/reductase 11	17	34948228	34957235	9008
DHRS3	dehydrogenase/reductase 3	1	12627939	12677737	49799
DHRS7	dehydrogenase/reductase 7	14	60610838	60636574	25737
DHX30	DExH-box helicase 30	3	47844399	47891685	47287
**DHX35**	DEAH-box helicase 35	20	37590942	37668366	77425
DHX36	DEAH-box helicase 36	3	153990335	154042286	51952
**DHX57**	DExH-box helicase 57	2	39024871	39103075	78205
DHX58	DExH-box helicase 58	17	40253422	40264751	11330
DIAPH1	diaphanous related formin 1	5	140894583	140998622	104040
DIAPH2	diaphanous related formin 2	X	95939662	96859996	920335
**DIAPH3**	diaphanous related formin 3	13	60239717	60738121	498405
DICER1	dicer 1, ribonuclease III	14	95552565	95624347	71783
DIDO1	death inducer-obliterator 1	20	61509090	61569304	60215
DIO2-AS1	DIO2 antisense RNA 1	14	80677762	80921812	244051
DIP2B	disco interacting protein 2 homolog B	12	50898768	51142450	243683
DIP2C	disco interacting protein 2 homolog C	10	320130	735683	415554
DIRC1	disrupted in renal carcinoma 1	2	189598882	189654831	55950
DIRC2	disrupted in renal carcinoma 2	3	122513642	122599986	86345
DIRC3	disrupted in renal carcinoma 3	2	218148742	218621316	472575
DIS3L2	DIS3 like 3'-5' exoribonuclease 2	2	232825955	233209060	383106
DISC1FP1	DISC1 fusion partner 1 (non-protein coding)	11	89984400	90648220	663821
DISP1	dispatched RND transporter family member 1	1	222988406	223179337	190932
DIXDC1	DIX domain containing 1	11	111797868	111893308	95441
DKK1	dickkopf WNT signaling pathway inhibitor 1	10	54074056	54077802	3747
DKK2	dickkopf WNT signaling pathway inhibitor 2	4	107842959	108204963	362005
DLC1	DLC1 Rho GTPase activating protein	8	12940870	13373167	432298
DLD	dihydrolipoamide dehydrogenase	7	107531415	107572175	40761
DLEC1	deleted in lung and esophageal cancer 1	3	38080696	38165516	84821
DLEU1	deleted in lymphocytic leukemia 1	13	50656307	51297372	641066
DLEU2	deleted in lymphocytic leukemia 2 (non-protein coding)	13	50601269	50699856	98588
DLG1	discs large MAGUK scaffold protein 1	3	196769431	197026171	256741
DLG2	discs large MAGUK scaffold protein 2	11	83166055	85338966	2172912
DLGAP1	DLG associated protein 1	18	3496030	4455335	959306
DLGAP2	DLG associated protein 2	8	1449532	1656642	207111
DLGAP4	DLG associated protein 4	20	34894258	35157040	262783
DLGAP5	DLG associated protein 5	14	55614830	55658396	43567
**DLL3**	delta like canonical Notch ligand 3	19	39989535	39999121	9587
DLST	dihydrolipoamide S-succinyltransferase	14	75348594	75370448	21855
DMC1	DNA meiotic recombinase 1	22	38914954	38966291	51338
DMD	dystrophin	X	31115794	33357558	2241765
DNAH11	dynein axonemal heavy chain 11	7	21582833	21941457	358625
DNAH12	dynein axonemal heavy chain 12	3	57327727	57530071	202345
DNAH17	dynein axonemal heavy chain 17	17	76419778	76573476	153699
DNAH2	dynein axonemal heavy chain 2	17	7620672	7737062	116391
DNAH6	dynein axonemal heavy chain 6	2	84743579	85046713	303135
DNAH7	dynein axonemal heavy chain 7	2	196602427	196933536	331110
**DNAH8**	dynein axonemal heavy chain 8	6	38683117	38998301	315185
DNAH9	dynein axonemal heavy chain 9	17	11501748	11873065	371318
DNAJA2	DnaJ heat shock protein family (Hsp40) member A2	16	46989299	47007699	18401
DNAJB2	DnaJ heat shock protein family (Hsp40) member B2	2	220143989	220151622	7634
DNAJB6	DnaJ heat shock protein family (Hsp40) member B6	7	157128075	157210133	82059

DNAJC1	DnaJ heat shock protein family (Hsp40) member C1	10	22045466	22292698	247233
DNAJC12	DnaJ heat shock protein family (Hsp40) member C12	10	69556427	69597924	41498
DNAJC2	DnaJ heat shock protein family (Hsp40) member C2	7	102952921	102985320	32400
DNAJC27	DnaJ heat shock protein family (Hsp40) member C27	2	25166505	25194963	28459
DNAJC30	DnaJ heat shock protein family (Hsp40) member C30	7	73095299	73097783	2485
DNAJC6	DnaJ heat shock protein family (Hsp40) member C6	1	65713902	65881552	167651
DNAJC8	DnaJ heat shock protein family (Hsp40) member C8	1	28525967	28559536	33570
DNASE1L3	deoxyribonuclease 1 like 3	3	58177984	58200424	22441
DNER	delta/notch like EGF repeat containing	2	230222345	230579274	356930
DNM1	dynamin 1	9	130965658	131017527	51870
DNM2	dynamin 2	19	10828755	10944164	115410
DNM3	dynamin 3	1	171810621	172387606	576986
DNMBP	dynamin binding protein	10	101635334	101769676	134343
DNMT1	DNA methyltransferase 1	19	10244021	10341962	97942
**DOC2B**	double C2 domain beta	17	5810	31427	25618
DOCK1	dedicator of cytokinesis 1	10	128593978	129250781	656804
DOCK10	dedicator of cytokinesis 10	2	225629807	225907162	277356
DOCK11	dedicator of cytokinesis 11	X	117629861	117820126	190266
DOCK2	dedicator of cytokinesis 2	5	169064251	169510386	446136
DOCK3	dedicator of cytokinesis 3	3	50712672	51421629	708958
DOCK4	dedicator of cytokinesis 4	7	111366166	111846466	480301
DOCK5	dedicator of cytokinesis 5	8	25042238	25275598	233361
DOCK7	dedicator of cytokinesis 7	1	62920399	63153969	233571
DOCK8	dedicator of cytokinesis 8	9	214854	465259	250406
DOCK9	dedicator of cytokinesis 9	13	99445741	99738879	293139
DOK5	docking protein 5	20	53092136	53267710	175575
DOK6	docking protein 6	18	67068291	67516323	448033
DPF3	double PHD fingers 3	14	73086004	73360809	274806
DPH5	diphthamide biosynthesis 5	1	101455179	101491644	36466
DPH6	diphthamine biosynthesis 6	15	35509546	35838394	328849
DPH6-AS1	DPH6 antisense RNA 1 (head to head)	15	35838396	36151202	312807
DPH7	diphthamide biosynthesis 7	9	140449356	140473387	24032
DPM2	dolichyl-phosphate mannosyltransferase subunit 2, regulatory	9	130697378	130700763	3386
DPP10	dipeptidyl peptidase like 10	2	115199876	116603328	1403453
**DPP6**	dipeptidyl peptidase like 6	7	153584182	154685995	1101814
DPY19L1	dpy-19 like 1	7	34968488	35077883	109396
DPY19L4	dpy-19 like 4 (C. elegans)	8	95731931	95806064	74134
DPY30	dpy-30, histone methyltransferase complex regulatory subunit	2	32092878	32264881	172004
DPYD	dihydropyrimidine dehydrogenase	1	97543299	98386605	843307
DPYS	dihydropyrimidinase	8	105342552	105479281	136730
DPYSL5	dihydropyrimidinase like 5	2	27070615	27173219	102605
DRD1	dopamine receptor D1	5	174867042	174871211	4170
DRD2	dopamine receptor D2	11	113280318	113346413	66096
DRD3	dopamine receptor D3	3	113847499	113918254	70756
**DRD4**	dopamine receptor D4	11	637293	640706	3414
DRD5	dopamine receptor D5	4	9783258	9785632	2375
DROSHA	drosha ribonuclease III	5	31400604	31532303	131700
DSC3	desmocollin 3	18	28569974	28622781	52808
DSCAM	DS cell adhesion molecule	21	41382926	42219065	836140
DSCAML1	DS cell adhesion molecule like 1	11	117298489	117688240	389752
DSCC1	DNA replication and sister chromatid cohesion 1	8	120846216	120868250	22035
DSCR3	DSCR3 arrestin fold containing	21	38595721	38640262	44542
DSCR4	Down syndrome critical region 4	21	39323728	39493454	169727
DSE	dermatan sulfate epimerase	6	116575336	116762424	187089
DST	dystonin	6	56322785	56819426	496642
DSTN	destrin, actin depolymerizing factor	20	17550508	17590564	40057
DSTYK	dual serine/threonine and tyrosine protein kinase	1	205111632	205180727	69096
DTD1	D-tyrosyl-tRNA deacylase 1	20	18568537	18744561	176025
DTNA	dystrobrevin alpha	18	32073254	32471808	398555
DTNBP1	dystrobrevin binding protein 1	6	15523032	15663289	140258
DUSP10	dual specificity phosphatase 10	1	221874766	221915518	40753
DUSP18	dual specificity phosphatase 18	22	31048038	31063877	15840
DUSP22	dual specificity phosphatase 22	6	291630	351355	59726
DYM	dymeclin	18	46570039	46987717	417679
**DYNC1H1**	dynein cytoplasmic 1 heavy chain 1	14	102430865	102517129	86265
DYNC1I1	dynein cytoplasmic 1 intermediate chain 1	7	95401866	95739634	337769
DYNC2H1	dynein cytoplasmic 2 heavy chain 1	11	102980160	103350591	370432
DYNC2LI1	dynein cytoplasmic 2 light intermediate chain 1	2	44001178	44037149	35972
DYRK1A	dual specificity tyrosine phosphorylation regulated kinase 1A	21	38738092	38889753	151662
DYSF	dysferlin	2	71680852	71913898	233047
DZANK1	double zinc ribbon and ankyrin repeat domains 1	20	18364011	18447925	83915
DZIP1	DAZ interacting zinc finger protein 1	13	96230457	96296957	66501
E2F2	E2F transcription factor 2	1	23832922	23857712	24791
EBF1	early B-cell factor 1	5	158122928	158526769	403842
EBF2	early B-cell factor 2	8	25699246	25902913	203668
EBF3	early B-cell factor 3	10	131633547	131762105	128559
EBF4	early B-cell factor 4	20	2673524	2740753	67230

EBNA1BP2	EBNA1 binding protein 2	1	43629846	43736607	106762
ECE1	endothelin converting enzyme 1	1	21543740	21671997	128258
ECT2	epithelial cell transforming 2	3	172468472	172539264	70793
EDA	ectodysplasin A	X	68835911	69259319	423409
**EDA2R**	ectodysplasin A2 receptor	X	65815479	65859108	43630
EDF1	endothelial differentiation related factor 1	9	139756571	139760738	4168
EDIL3	EGF like repeats and discoidin domains 3	5	83236373	83680611	444239
EDN1	endothelin 1	6	12290596	12297427	6832
EDNRB-AS1	EDNRB antisense RNA 1	13	78402357	78408085	5729
EED	embryonic ectoderm development	11	85955586	85989855	34270
**EEF1D**	eukaryotic translation elongation factor 1 delta	8	144661867	144681711	19845
EEFSEC	eukaryotic elongation factor, selenocysteine-tRNA specific	3	127872297	128127485	255189
EFCAB1	EF-hand calcium binding domain 1	8	49623348	49647870	24523
EFCAB12	EF-hand calcium binding domain 12	3	129120164	129147494	27331
EFCAB13	EF-hand calcium binding domain 13	17	45400656	45518678	118023
EFCAB2	EF-hand calcium binding domain 2	1	245133007	245290466	157460
EFCAB6	EF-hand calcium binding domain 6	22	43924624	44208217	283594
EFCAB7	EF-hand calcium binding domain 7	1	63989043	64038364	49322
**EFEMP1**	EGF containing fibulin like extracellular matrix protein 1	2	56093102	56151274	58173
EFHC1	EF-hand domain containing 1	6	52285106	52387892	102787
EFNA5	ephrin A5	5	106712590	107006596	294007
EFNB1	ephrin B1	X	68048840	68061990	13151
EFTUD2	elongation factor Tu GTP binding domain containing 2	17	42927311	42977030	49720
EGFEM1P	EGF like and EMI domain containing 1, pseudogene	3	167967310	168548387	581078
EGFLAM	EGF like, fibronectin type III and laminin G domains	5	38258511	38465123	206613
EGFR	epidermal growth factor receptor	7	55086714	55324313	237600
EGR2	early growth response 2	10	64571756	64679660	107905
EHBP1	EH domain binding protein 1	2	62900986	63273622	372637
EHMT2	euchromatic histone lysine methyltransferase 2	6	31847536	31865464	17929
EIF2AK2	eukaryotic translation initiation factor 2 alpha kinase 2	2	37326353	37384208	57856
EIF2B1	eukaryotic translation initiation factor 2B subunit alpha	12	124104953	124118313	13361
EIF2B2	eukaryotic translation initiation factor 2B subunit beta	14	75469614	75476292	6679
EIF2B3	eukaryotic translation initiation factor 2B subunit gamma	1	45316450	45452282	135833
EIF2B4	eukaryotic translation initiation factor 2B subunit delta	2	27587219	27593353	6135
EIF2B5	eukaryotic translation initiation factor 2B subunit epsilon	3	183852826	184402546	549721
EIF3A	eukaryotic translation initiation factor 3 subunit A	10	120794356	120840316	45961
EIF3D	eukaryotic translation initiation factor 3 subunit D	22	36906897	36925483	18587
EIF3E	eukaryotic translation initiation factor 3 subunit E	8	109213445	109447562	234118
EIF3J	eukaryotic translation initiation factor 3 subunit J	15	44829255	44855227	25973
EIF4B	eukaryotic translation initiation factor 4B	12	53399942	53435993	36052
EIF4E2	eukaryotic translation initiation factor 4E family member 2	2	233414762	233448354	33593
EIF4ENIF1	eukaryotic translation initiation factor 4E nuclear import factor 1	22	31832963	31892094	59132
EIF4G1	eukaryotic translation initiation factor 4 gamma 1	3	184032283	184053146	20864
EIF4G2	eukaryotic translation initiation factor 4 gamma 2	11	10818597	10830657	12061
EIF4G3	eukaryotic translation initiation factor 4 gamma 3	1	21132963	21503377	370415
EIF4H	eukaryotic translation initiation factor 4H	7	73588575	73611431	22857
EIF5B	eukaryotic translation initiation factor 5B	2	99953816	100017789	63974
ELAVL1	ELAV like RNA binding protein 1	19	8023463	8070543	47081
ELAVL3	ELAV like RNA binding protein 3	19	11562141	11591861	29721
ELAVL4	ELAV like RNA binding protein 4	1	50513686	50669458	155773
ELFN2	extracellular leucine rich repeat and fibronectin type III domain containing 2	22	37764000	37823505	59506
ELK1	ELK1, ETS transcription factor	X	47494920	47510003	15084
ELK3	ELK3, ETS transcription factor	12	96588160	96663613	75454
ELK4	ELK4, ETS transcription factor	1	205577071	205601090	24020
ELL	elongation factor for RNA polymerase II	19	18553473	18632937	79465
ELMO1	engulfment and cell motility 1	7	36893961	37488852	594892
ELOVL5	ELOVL fatty acid elongase 5	6	53132196	53213947	81752
ELOVL6	ELOVL fatty acid elongase 6	4	110967002	111120355	153354
**ELP3**	elongator acetyltransferase complex subunit 3	8	27947190	28048673	101484
ELP4	elongator acetyltransferase complex subunit 4	11	31531297	31805546	274250
EMC3	ER membrane protein complex subunit 3	3	10004221	10052800	48580
EML1	echinoderm microtubule associated protein like 1	14	100204030	100408397	204368
EML4	echinoderm microtubule associated protein like 4	2	42396490	42559688	163199
EML5	echinoderm microtubule associated protein like 5	14	89078775	89259096	180322
EML6	echinoderm microtubule associated protein like 6	2	54950636	55199157	248522
EN1	engrailed homeobox 1	2	119599747	119605254	5508
EN2	engrailed homeobox 2	7	155250824	155257526	6703
ENAH	enabled homolog (Drosophila)	1	225674537	225840844	166308
ENO4	enolase family member 4	10	118609023	118671299	62277
ENOX1	ecto-NOX disulfide-thiol exchanger 1	13	43787654	44361044	573391
ENOX2	ecto-NOX disulfide-thiol exchanger 2	X	129757350	130037208	279859
ENPEP	glutamyl aminopeptidase	4	111286889	111486441	199553
**ENPP1**	ectonucleotide pyrophosphatase/phosphodiesterase 1	6	132129156	132216295	87140
ENPP6	ectonucleotide pyrophosphatase/phosphodiesterase 6	4	185009859	185142383	132525
ENTHD1	ENTH domain containing 1	22	40139049	40289868	150820
ENTPD1-AS1	ENTPD1 antisense RNA 1	10	97512963	97849995	337033

ENTPD4	ectonucleoside triphosphate diphosphohydrolase 4	8	23243296	23315208	71913
ENTPD7	ectonucleoside triphosphate diphosphohydrolase 7	10	101419263	101465997	46735
ENY2	ENY2, transcription and export complex 2 subunit	8	110346553	110358182	11630
EOMES	eomesodermin	3	27757440	27764206	6767
EP400NL	EP400 N-terminal like	12	132568828	132613029	44202
EPB41L1	erythrocyte membrane protein band 4.1 like 1	20	34679426	34820721	141296
EPB41L2	erythrocyte membrane protein band 4.1 like 2	6	131160487	131384462	223976
EPB41L3	erythrocyte membrane protein band 4.1 like 3	18	5392383	5630699	238317
EPB41L4A	erythrocyte membrane protein band 4.1 like 4A	5	111478138	111755013	276876
EPB41L4B	erythrocyte membrane protein band 4.1 like 4B	9	111934255	112083244	148990
EPB41L5	erythrocyte membrane protein band 4.1 like 5	2	120770581	120936695	166115
EPC1	enhancer of polycomb homolog 1	10	32556679	32667726	111048
EPC2	enhancer of polycomb homolog 2	2	149402009	149545130	143122
**EPHA3**	EPH receptor A3	3	89156674	89531284	374611
**EPHA4**	EPH receptor A4	2	222282747	222438922	156176
EPHA5	EPH receptor A5	4	66185281	66536213	350933
EPHA6	EPH receptor A6	3	96533425	97471304	937880
EPHA7	EPH receptor A7	6	93949738	94129265	179528
EPHB1	EPH receptor B1	3	134316643	134979309	662667
EPHB2	EPH receptor B2	1	23037332	23241818	204487
EPHX1	epoxide hydrolase 1	1	225997794	226033260	35467
EPHX2	epoxide hydrolase 2	8	27348296	27403081	54786
EPM2A	EPM2A, laforin glucan phosphatase	6	145822719	146057160	234442
EPN2	epsin 2	17	19118928	19240028	121101
EPS15	epidermal growth factor receptor pathway substrate 15	1	51819935	51985000	165066
**EPS8**	epidermal growth factor receptor pathway substrate 8	12	15773092	16035263	262172
EPSTI1	epithelial stromal interaction 1	13	43460524	43566407	105884
ERAP1	endoplasmic reticulum aminopeptidase 1	5	96096521	96143803	47283
ERBB2	erb-b2 receptor tyrosine kinase 2	17	37844167	37886679	42513
**ERBB4**	erb-b2 receptor tyrosine kinase 4	2	212240446	213403565	1163120
ERC1	ELKS/RAB6-interacting/CAST family member 1	12	1099675	1605099	505425
ERC2	ELKS/RAB6-interacting/CAST family member 2	3	55542336	56502391	960056
ERCC4	ERCC excision repair 4, endonuclease catalytic subunit	16	14014014	14046202	32189
ERCC6	ERCC excision repair 6, chromatin remodeling factor	10	50663414	50747584	84171
ERCC6L2	ERCC excision repair 6 like 2	9	98637983	98776842	138860
**ERCC8**	ERCC excision repair 8, CSA ubiquitin ligase complex subunit	5	60169658	60240900	71243
ERG	ERG, ETS transcription factor	21	39751949	40033704	281756
ERI3	ERI1 exoribonuclease family member 3	1	44686742	44820932	134191
ERLIN1	ER lipid raft associated 1	10	101909851	101948091	38241
ERLIN2	ER lipid raft associated 2	8	37594117	37616619	22503
ESR1	estrogen receptor 1	6	151977826	152450754	472929
ESRRG	estrogen related receptor gamma	1	216676588	217311097	634510
ETF1	eukaryotic translation termination factor 1	5	137841784	137878989	37206
ETNK1	ethanolamine kinase 1	12	22778009	22843599	65591
ETS2	ETS proto-oncogene 2, transcription factor	21	40177231	40196879	19649
ETV1	ETS variant 1	7	13930853	14031050	100198
ETV4	ETS variant 4	17	41605212	41656988	51777
ETV6	ETS variant 6	12	11802788	12048336	245549
EVL	Enah/Vasp-like	14	100437786	100610573	172788
**EWSR1**	EWS RNA binding protein 1	22	29663998	29696515	32518
EXD2	exonuclease 3'-5' domain containing 2	14	69658228	69709075	50848
**EXO1**	exonuclease 1	1	242011269	242058450	47182
EXOC1	exocyst complex component 1	4	56719782	56771200	51419
EXOC2	exocyst complex component 2	6	485133	693117	207985
EXOC3L2	exocyst complex component 3 like 2	19	45715879	45737469	21591
**EXOC4**	exocyst complex component 4	7	132937829	133751342	813514
EXOC6B	exocyst complex component 6B	2	72403113	73053170	650058
EXOSC3	exosome component 3	9	37766975	37801434	34460
EXPH5	exophilin 5	11	108376158	108464465	88308
EXT1	exostosin glycosyltransferase 1	8	118806729	119124092	317364
EXT2	exostosin glycosyltransferase 2	11	44117099	44266979	149881
EYA1	EYA transcriptional coactivator and phosphatase 1	8	72109668	72274467	164800
EYA2	EYA transcriptional coactivator and phosphatase 2	20	45523263	45817492	294230
EYS	eyes shut homolog (Drosophila)	6	64429876	66417118	1987243
EZH2	enhancer of zeste 2 polycomb repressive complex 2 subunit	7	148504475	148581413	76939
EZR	ezrin	6	159186773	159240444	53672
F13A1	coagulation factor XIII A chain	6	6144318	6321246	176929
F2	coagulation factor II, thrombin	11	46740730	46761056	20327
F5	coagulation factor V	1	169483404	169555826	72423
F8	coagulation factor VIII	X	154064063	154255215	191153
FA2H	fatty acid 2-hydroxylase	16	74746853	74808729	61877
FAAH2	fatty acid amide hydrolase 2	X	57313139	57515629	202491
FABP1	fatty acid binding protein 1	2	88422510	88427635	5126
FAF1	Fas associated factor 1	1	50905150	51425935	520786
FAHD2CP	fumarylacetoacetate hydrolase domain containing 2C, pseudogene	2	96676299	96689128	12830
FAM102B	family with sequence similarity 102 member B	1	109102711	109187522	84812
FAM104A	family with sequence similarity 104 member A	17	71203492	71232892	29401

FAM107B	family with sequence similarity 107 member B	10	14560556	14816896	256341
FAM110B	family with sequence similarity 110 member B	8	58907068	59116838	209771
**FAM114A1**	family with sequence similarity 114 member A1	4	38869298	38947360	78063
FAM114A2	family with sequence similarity 114 member A2	5	153369688	153418496	48809
FAM118B	family with sequence similarity 118 member B	11	126081309	126132881	51573
FAM120A	family with sequence similarity 120A	9	96214004	96328397	114394
FAM120B	family with sequence similarity 120B	6	170599791	170716153	116363
FAM120C	family with sequence similarity 120C	X	54094757	54209714	114958
FAM126A	family with sequence similarity 126 member A	7	22980878	23053749	72872
FAM126B	family with sequence similarity 126 member B	2	201838441	201936394	97954
FAM129A	family with sequence similarity 129 member A	1	184759858	184943682	183825
FAM131A	family with sequence similarity 131 member A	3	184053714	184064063	10350
FAM135A	family with sequence similarity 135 member A	6	71122644	71270877	148234
**FAM135B**	family with sequence similarity 135 member B	8	139142266	139509065	366800
FAM13A	family with sequence similarity 13 member A	4	89647106	90032549	385444
FAM13C	family with sequence similarity 13 member C	10	61005890	61122939	117050
FAM155A	family with sequence similarity 155 member A	13	107820883	108519083	698201
FAM157C	family with sequence similarity 157 member C (non-protein coding)	16	90168679	90244752	76074
FAM159A	family with sequence similarity 159 member A	1	53099016	53135355	36340
FAM160A1	family with sequence similarity 160 member A1	4	152330368	152584784	254417
FAM160B1	family with sequence similarity 160 member B1	10	116581503	116659591	78089
FAM161B	family with sequence similarity 161 member B	14	74398204	74417117	18914
FAM168A	family with sequence similarity 168 member A	11	73111532	73309234	197703
FAM172A	family with sequence similarity 172 member A	5	92953775	93447404	493630
FAM172BP	family with sequence similarity 172 member B, pseudogene	3	101237711	101242731	5021
FAM175A	family with sequence similarity 175 member A	4	84382092	84444501	62410
FAM189A1	family with sequence similarity 189 member A1	15	29412457	29862927	450471
FAM192A	family with sequence similarity 192 member A	16	57186378	57220028	33651
FAM193A	family with sequence similarity 193 member A	4	2626988	2734292	107305
FAM193B	family with sequence similarity 193 member B	5	176946789	176981542	34754
FAM199X	family with sequence similarity 199, X-linked	X	103411301	103440583	29283
FAM19A1	family with sequence similarity 19 member A1, C-C motif chemokine like	3	68053359	68594776	541418
FAM19A2	family with sequence similarity 19 member A2, C-C motif chemokine like	12	62102040	62672931	570892
FAM19A5	family with sequence similarity 19 member A5, C-C motif chemokine like	22	48885272	49246724	361453
FAM204A	family with sequence similarity 204 member A	10	120065401	120101840	36440
FAM208A	family with sequence similarity 208 member A	3	56654161	56717265	63105
FAM212B	family with sequence similarity 212 member B	1	112223252	112298446	75195
FAM214A	family with sequence similarity 214 member A	15	52873514	53002014	128501
FAM222B	family with sequence similarity 222 member B	17	27082996	27182250	99255
FAM227B	family with sequence similarity 227 member B	15	49619159	49913128	293970
FAM49A	family with sequence similarity 49 member A	2	16730727	16847599	116873
**FAM49B**	family with sequence similarity 49 member B	8	130851839	131029375	177537
FAM50A	family with sequence similarity 50 member A	X	153672473	153679002	6530
FAM53B	family with sequence similarity 53 member B	10	126307861	126432838	124978
FAM60A	family with sequence similarity 60 member A	12	31433518	31479992	46475
FAM66E	family with sequence similarity 66 member E	8	7812536	7866277	53742
FAM69A	family with sequence similarity 69 member A	1	93307724	93427057	119334
FAM71D	family with sequence similarity 71 member D	14	67656110	67695267	39158
FAM71F2	family with sequence similarity 71 member F2	7	128312342	128326929	14588
FAM91A1	family with sequence similarity 91 member A1	8	124780696	124827692	46997
FANCI	Fanconi anemia complementation group I	15	89787180	89860492	73313
FANCL	Fanconi anemia complementation group L	2	58386378	58468507	82130
FANCM	Fanconi anemia complementation group M	14	45605143	45670093	64951
FANK1	fibronectin type III and ankyrin repeat domains 1	10	127585108	127698161	113054
FAP	fibroblast activation protein alpha	2	163027194	163101661	74468
FAR2	fatty acyl-CoA reductase 2	12	29302036	29493913	191878
FARP1	FERM, ARH/RhoGEF and pleckstrin domain protein 1	13	98794816	99102027	307212
FARP2	FERM, ARH/RhoGEF and pleckstrin domain protein 2	2	242295658	242434256	138599
FARS2	phenylalanyl-tRNA synthetase 2, mitochondrial	6	5261277	5771813	510537
FARSB	phenylalanyl-tRNA synthetase beta subunit	2	223435255	223521056	85802
FAS	Fas cell surface death receptor	10	90750414	90775542	25129
FAT1	FAT atypical cadherin 1	4	187508937	187647876	138940
FAT3	FAT atypical cadherin 3	11	92085262	92629618	544357
FAT4	FAT atypical cadherin 4	4	126237554	126414087	176534
FBLN5	fibulin 5	14	92335756	92414331	78576
FBLN7	fibulin 7	2	112895962	112945791	49830
FBN1	fibrillin 1	15	48700503	48938046	237544
FBN2	fibrillin 2	5	127593601	127994878	401278
FBRSL1	fibrosin like 1	12	133066137	133161774	95638
FBXL12	F-box and leucine rich repeat protein 12	19	9920943	9938492	17550
FBXL13	F-box and leucine rich repeat protein 13	7	102453308	102715286	261979
FBXL17	F-box and leucine rich repeat protein 17	5	107194736	107717799	523064
FBXL18	F-box and leucine rich repeat protein 18	7	5470966	5553429	82464
FBXL20	F-box and leucine rich repeat protein 20	17	37415384	37558776	143393

FBXL3	F-box and leucine rich repeat protein 3	13	77566740	77601330	34591
FBXL7	F-box and leucine rich repeat protein 7	5	15500305	15939900	439596
FBXO15	F-box protein 15	18	71740588	71815100	74513
FBXO3	F-box protein 3	11	33762485	33796089	33605
FBXO31	F-box protein 31	16	87360593	87425748	65156
FBXO32	F-box protein 32	8	124510129	124553446	43318
FBXO38	F-box protein 38	5	147763498	147822399	58902
**FBXO42**	F-box protein 42	1	16573334	16678949	105616
FBXO46	F-box protein 46	19	46213887	46234162	20276
FBXO5	F-box protein 5	6	153291664	153304714	13051
FBXO7	F-box protein 7	22	32870663	32894818	24156
FBXW11	F-box and WD repeat domain containing 11	5	171288553	171433877	145325
FBXW4	F-box and WD repeat domain containing 4	10	103370423	103455052	84630
FBXW7	F-box and WD repeat domain containing 7	4	153242410	153457253	214844
FCER2	Fc fragment of IgE receptor II	19	7753644	7767032	13389
FCHO2	FCH domain only 2	5	72251808	72386349	134542
FCHSD2	FCH and double SH3 domains 2	11	72547790	72853306	305517
FCRL3	Fc receptor like 3	1	157644111	157670647	26537
FDPS	farnesyl diphosphate synthase	1	155278539	155290457	11919
FEM1C	fem-1 homolog C	5	114856608	114880591	23984
FER	FER tyrosine kinase	5	108083523	108532542	449020
FER1L6-AS2	FER1L6 antisense RNA 2	8	125058314	125183763	125450
FERMT3	fermitin family member 3	11	63974150	63991354	17205
**FEZF2**	FEZ family zinc finger 2	3	62355356	62359999	4644
FGD3	FYVE, RhoGEF and PH domain containing 3	9	95709733	95798518	88786
FGD4	FYVE, RhoGEF and PH domain containing 4	12	32552463	32798984	246522
FGD6	FYVE, RhoGEF and PH domain containing 6	12	95470525	95611258	140734
FGF1	fibroblast growth factor 1	5	141971743	142077617	105875
FGF12	fibroblast growth factor 12	3	191857184	192485553	628370
FGF13	fibroblast growth factor 13	X	137713735	138304939	591205
FGF14	fibroblast growth factor 14	13	102372134	103054124	681991
FGF20	fibroblast growth factor 20	8	16849678	16859690	10013
FGF3	fibroblast growth factor 3	11	69624992	69633792	8801
FGFBP2	fibroblast growth factor binding protein 2	4	15961866	15970932	9067
FGFR1	fibroblast growth factor receptor 1	8	38268656	38326352	57697
FGFR1OP2	FGFR1 oncogene partner 2	12	27091316	27119583	28268
FGFR2	fibroblast growth factor receptor 2	10	123237848	123357972	120125
FGFR3	fibroblast growth factor receptor 3	4	1795034	1810599	15566
**FGGY**	FGGY carbohydrate kinase domain containing	1	59762310	60233347	471038
FHAD1	forkhead associated phosphopeptide binding domain 1	1	15573768	15726779	153012
FHDC1	FH2 domain containing 1	4	153857504	153900848	43345
FHIT	fragile histidine triad	3	59735036	61237133	1502098
FHOD3	formin homology 2 domain containing 3	18	33877677	34360018	482342
**FIG4**	FIG4 phosphoinositide 5-phosphatase	6	110012499	110146631	134133
FIGNL1	fidgetin like 1	7	50511831	50518088	6258
FILIP1	filamin A interacting protein 1	6	76001575	76203454	201880
FKBP14	FK506 binding protein 14	7	30050203	30066300	16098
FKBP5	FK506 binding protein 5	6	35541362	35696360	154999
FKRP	fukutin related protein	19	47249303	47280245	30943
FKTN	fukutin	9	108320411	108403399	82989
FLG	filaggrin	1	152274651	152297679	23029
FLI1	Fli-1 proto-oncogene, ETS transcription factor	11	128556430	128683162	126733
FLNA	filamin A	X	153576892	153603006	26115
FLOT1	flotillin 1	6	30695486	30710510	15025
FLT1	fms related tyrosine kinase 1	13	28874489	29069265	194777
FLT3	fms related tyrosine kinase 3	13	28577411	28674729	97319
FMN1	formin 1	15	33057747	33486897	429151
**FMN2**	formin 2	1	240177648	240638489	460842
**FMNL2**	formin like 2	2	153191751	153506348	314598
FMO6P	flavin containing monooxygenase 6 pseudogene	1	171106879	171130707	23829
FMOD	fibromodulin	1	203309756	203320617	10862
FMR1	fragile X mental retardation 1	X	146993469	147032645	39177
FMR1-AS1	FMR1 antisense RNA 1	X	146990949	146993335	2387
FN1	fibronectin 1	2	216225163	216300895	75733
FN3KRP	fructosamine 3 kinase related protein	17	80674559	80688204	13646
FNBP1	formin binding protein 1	9	132649466	132805473	156008
FNDC3A	fibronectin type III domain containing 3A	13	49550048	49783915	233868
**FNDC3B**	fibronectin type III domain containing 3B	3	171757418	172119455	362038
FNIP1	folliculin interacting protein 1	5	130977407	131132710	155304
FOCAD	focadhesin	9	20658308	20995954	337647
FOLR1	folate receptor 1	11	71900602	71907345	6744
FOPNL	FGFR1OP N-terminal like	16	15959577	15982482	22906
FOS	Fos proto-oncogene, AP-1 transcription factor subunit	14	75745477	75748933	3457
FOXA1	forkhead box A1	14	38059189	38069245	10057
FOXA3	forkhead box A3	19	46367247	46377055	9809
FOXG1	forkhead box G1	14	29235050	29238870	3821
FOXJ3	forkhead box J3	1	42642210	42801548	159339

FOXN3	forkhead box N3	14	89591215	90085493	494279
FOXN4	forkhead box N4	12	109715784	109747025	31242
FOXO1	forkhead box O1	13	41129804	41240734	110931
FOXO3	forkhead box O3	6	108881038	109005977	124940
FOXO6	forkhead box O6	1	41827594	41849262	21669
FOXP1	forkhead box P1	3	71003844	71633140	629297
FOXP2	forkhead box P2	7	113726382	114333827	607446
FRAS1	Fraser extracellular matrix complex subunit 1	4	78978724	79465423	486700
FREM1	FRAS1 related extracellular matrix 1	9	14734664	14910993	176330
FREM2	FRAS1 related extracellular matrix protein 2	13	39261266	39460074	198809
FREM3	FRAS1 related extracellular matrix 3	4	144498455	144621828	123374
FRMD3	FERM domain containing 3	9	85857905	86153461	295557
FRMD4A	FERM domain containing 4A	10	13685706	14504141	818436
FRMD5	FERM domain containing 5	15	44162962	44487450	324489
FRMD6	FERM domain containing 6	14	51955818	52197445	241628
FRMPD2	FERM and PDZ domain containing 2	10	49364601	49482941	118341
FRMPD4	FERM and PDZ domain containing 4	X	12156585	12742642	586058
FRY	FRY microtubule binding protein	13	32605437	32870794	265358
FRYL	FRY like transcription coactivator	4	48499378	48782339	282962
FRZB	frizzled-related protein	2	183698002	183731890	33889
FSD1L	fibronectin type III and SPRY domain containing 1 like	9	108210077	108314714	104638
FSHR	follicle stimulating hormone receptor	2	49189296	49381676	192381
FSIP1	fibrous sheath interacting protein 1	15	39892232	40075031	182800
FSTL1	follistatin like 1	3	120111140	120170100	58961
FSTL4	follistatin like 4	5	132532147	132948255	416109
FSTL5	follistatin like 5	4	162305049	163085187	780139
FTCDNL1	formiminotransferase cyclodeaminase N-terminal like	2	200625267	200715896	90630
FTH1	ferritin heavy chain 1	11	61727190	61735132	7943
FTL	ferritin light chain	19	49468558	49470135	1578
FTO	FTO, alpha-ketoglutarate dependent dioxygenase	16	53737875	54155853	417979
FTX	FTX transcript, XIST regulator (non-protein coding)	X	73183790	73513409	329620
**FUS**	FUS RNA binding protein	16	31191431	31203127	11697
FUT8	fucosyltransferase 8	14	65877310	66210839	333530
FUT9	fucosyltransferase 9	6	96463860	96663488	199629
FXN	frataxin	9	71650175	71715094	64920
FYB	FYN binding protein	5	39105338	39274630	169293
FYCO1	FYVE and coiled-coil domain containing 1	3	45959396	46037316	77921
FYN	FYN proto-oncogene, Src family tyrosine kinase	6	111981535	112194655	213121
FYTTD1	forty-two-three domain containing 1	3	197464050	197514467	50418
FZD3	frizzled class receptor 3	8	28351729	28431775	80047
GAA	glucosidase alpha, acid	17	78075355	78093678	18324
GAB2	GRB2 associated binding protein 2	11	77926343	78129394	203052
GAB3	GRB2 associated binding protein 3	X	153903529	153979858	76330
GABBR2	gamma-aminobutyric acid type B receptor subunit 2	9	101050391	101471479	421089
GABPB1	GA binding protein transcription factor beta subunit 1	15	50569389	50647605	78217
GABRA1	gamma-aminobutyric acid type A receptor alpha1 subunit	5	161274197	161326975	52779
GABRA2	gamma-aminobutyric acid type A receptor alpha2 subunit	4	46250444	46477247	226804
GABRA3	gamma-aminobutyric acid type A receptor alpha3 subunit	X	151334706	151619830	285125
GABRB1	gamma-aminobutyric acid type A receptor beta1 subunit	4	46995740	47428461	432722
GABRB2	gamma-aminobutyric acid type A receptor beta2 subunit	5	160715436	160976050	260615
GABRB3	gamma-aminobutyric acid type A receptor beta3 subunit	15	26788693	27184686	395994
GABRD	gamma-aminobutyric acid type A receptor delta subunit	1	1950780	1962192	11413
GABRG2	gamma-aminobutyric acid type A receptor gamma2 subunit	5	161494546	161582542	87997
GABRG3	gamma-aminobutyric acid type A receptor gamma3 subunit	15	27216429	27778373	561945
GABRR1	gamma-aminobutyric acid type A receptor rho1 subunit	6	89887220	89940997	53778
GAD1	glutamate decarboxylase 1	2	171669723	171717661	47939
GADL1	glutamate decarboxylase like 1	3	30767692	30936257	168566
GAK	cyclin G associated kinase	4	843064	926161	83098
GALC	galactosylceramidase	14	88304164	88460009	155846
GALNT1	polypeptide N-acetylgalactosaminyltransferase 1	18	33161081	33291798	130718
GALNT10	polypeptide N-acetylgalactosaminyltransferase 10	5	153570290	153800544	230255
GALNT13	polypeptide N-acetylgalactosaminyltransferase 13	2	154728426	155310361	581936
GALNT14	polypeptide N-acetylgalactosaminyltransferase 14	2	31133333	31378068	244736
GALNT2	polypeptide N-acetylgalactosaminyltransferase 2	1	230193536	230417870	224335
GALNT7	polypeptide N-acetylgalactosaminyltransferase 7	4	174089904	174245118	155215
GALNT9	polypeptide N-acetylgalactosaminyltransferase 9	12	132680924	132905935	225012
GALNTL5	polypeptide N-acetylgalactosaminyltransferase-like 5	7	151653464	151717019	63556
GALNTL6	polypeptide N-acetylgalactosaminyltransferase-like 6	4	172733405	173962710	1229306
GALP	galanin like peptide	19	56687389	56697144	9756
GAMT	guanidinoacetate N-methyltransferase	19	1397091	1401569	4479
GAN	gigaxonin	16	81348557	81424489	75933
GAP43	growth associated protein 43	3	115342171	115440337	98167
GAPDH	glyceraldehyde-3-phosphate dehydrogenase	12	6643093	6647537	4445
GAPDHS	glyceraldehyde-3-phosphate dehydrogenase, spermatogenic	19	36024314	36036218	11905
**GARS**	glycyl-tRNA synthetase	7	30634297	30673649	39353

GART	phosphoribosylglycinamide formyltransferase, phosphoribosylglycinamide synthetase, phosphoribosylaminoimidazole synthetase	21	34876238	34915797	39560
GAS2	growth arrest specific 2	11	22647188	22834601	187414
GAS6-AS2	GAS6 antisense RNA 2 (head to head)	13	114567141	114569806	2666
GATA2	GATA binding protein 2	3	128198270	128212028	13759
GATA2-AS1	GATA2 antisense RNA 1	3	128208036	128216768	8733
**GATA4**	GATA binding protein 4	8	11534468	11617511	83044
GATAD1	GATA zinc finger domain containing 1	7	92076767	92088150	11384
GATAD2A	GATA zinc finger domain containing 2A	19	19496635	19619740	123106
GATAD2B	GATA zinc finger domain containing 2B	1	153777201	153895451	118251
GBA	glucosylceramidase beta	1	155204243	155214490	10248
GBE1	1,4-alpha-glucan branching enzyme 1	3	81538850	81811312	272463
GBF1	golgi brefeldin A resistant guanine nucleotide exchange factor 1	10	104005289	104142656	137368
GBP7	guanylate binding protein 7	1	89597434	89641723	44290
GBX1	gastrulation brain homeobox 1	7	150845676	150871832	26157
GCH1	GTP cyclohydrolase 1	14	55308726	55369570	60845
GCK	glucokinase	7	44183872	44237769	53898
GCLC	glutamate-cysteine ligase catalytic subunit	6	53362139	53481768	119630
GCSH	glycine cleavage system protein H	16	81115566	81130008	14443
GDAP1	ganglioside induced differentiation associated protein 1	8	75233365	75401107	167743
GDAP2	ganglioside induced differentiation associated protein 2	1	118406107	118472253	66147
GDI2	GDP dissociation inhibitor 2	10	5807186	5884095	76910
GDNF	glial cell derived neurotrophic factor	5	37812779	37839788	27010
GDPD5	glycerophosphodiester phosphodiesterase domain containing 5	11	75145685	75236948	91264
**GEMIN6**	gem nuclear organelle associated protein 6	2	38978676	39012142	33467
GET4	golgi to ER traffic protein 4	7	916189	936073	19885
GFAP	glial fibrillary acidic protein	17	42982376	42994305	11930
GFM2	G elongation factor mitochondrial 2	5	74017029	74063196	46168
GGA2	golgi associated, gamma adaptin ear containing, ARF binding protein 2	16	23474863	23533316	58454
GGACT	gamma-glutamylamine cyclotransferase	13	101183810	101241782	57973
GGH	gamma-glutamyl hydrolase	8	63927638	63951730	24093
GHR	growth hormone receptor	5	42423879	42721979	298101
GIF	gastric intrinsic factor	11	59596741	59612974	16234
GIGYF2	GRB10 interacting GYF protein 2	2	233562009	233725285	163277
GIN1	gypsy retrotransposon integrase 1	5	102421704	102455855	34152
GJC1	gap junction protein gamma 1	17	42875816	42908184	32369
GJC2	gap junction protein gamma 2	1	228337553	228347527	9975
GK5	glycerol kinase 5 (putative)	3	141882414	141944449	62036
GLB1	galactosidase beta 1	3	33038100	33138722	100623
GLDC	glycine decarboxylase	9	6532464	6645650	113187
GLDN	gliomedin	15	51633826	51700210	66385
GLG1	golgi glycoprotein 1	16	74485856	74641012	155157
GLI2	GLI family zinc finger 2	2	121493199	121750229	257031
GLI3	GLI family zinc finger 3	7	42000548	42277469	276922
GLIS1	GLIS family zinc finger 1	1	53971910	54199877	227968
GLIS3	GLIS family zinc finger 3	9	3824127	4348392	524266
GLO1	glyoxalase I	6	38643701	38670917	27217
GLP2R	glucagon like peptide 2 receptor	17	9725523	9795419	69897
GLRA1	glycine receptor alpha 1	5	151202074	151304403	102330
GLRB	glycine receptor beta	4	157997209	158093242	96034
GLT1D1	glycosyltransferase 1 domain containing 1	12	129337972	129469509	131538
GLUD1	glutamate dehydrogenase 1	10	88810243	88854623	44381
GLUD2	glutamate dehydrogenase 2	X	120181462	120183794	2333
GLYR1	glyoxylate reductase 1 homolog	16	4853204	4897343	44140
GMDS	GDP-mannose 4,6-dehydratase	6	1624041	2245926	621886
GML	glycosylphosphatidylinositol anchored molecule like	8	143915663	143997922	82260
GMPPB	GDP-mannose pyrophosphorylase B	3	49754277	49761384	7108
GMPR	guanosine monophosphate reductase	6	16238811	16295780	56970
GNA12	G protein subunit alpha 12	7	2767746	2883958	116213
GNAI1	G protein subunit alpha i1	7	79763271	79848718	85448
GNAI3	G protein subunit alpha i3	1	110091233	110136975	45743
GNAL	G protein subunit alpha L	18	11688955	11885684	196730
GNAO1	G protein subunit alpha o1	16	56225302	56391356	166055
**GNAQ**	G protein subunit alpha q	9	80331003	80646374	315372
GNB1	G protein subunit beta 1	1	1716729	1822495	105767
GNB3	G protein subunit beta 3	12	6949118	6956557	7440
GNG7	G protein subunit gamma 7	19	2511217	2702707	191491
GNGT1	G protein subunit gamma transducin 1	7	93220885	93540577	319693
GNL2	G protein nucleolar 2	1	38032417	38061536	29120
GNPAT	glyceronephosphate O-acyltransferase	1	231376953	231413719	36767
GOLM1	golgi membrane protein 1	9	88641061	88715088	74028
GON4L	gon-4 like	1	155719508	155829191	109684
GOPC	golgi associated PDZ and coiled-coil motif containing	6	117639374	117923691	284318
GORAB	golgin, RAB6 interacting	1	170501270	170522587	21318
GORASP1	golgi reassembly stacking protein 1	3	39138150	39149854	11705
GOSR2	golgi SNAP receptor complex member 2	17	45000483	45105003	104521

GPATCH2L	G-patch domain containing 2 like	14	76618259	76720685	102427
GPATCH3	G-patch domain containing 3	1	27216979	27226957	9979
GPATCH8	G-patch domain containing 8	17	42472652	42580798	108147
GPC3	glypican 3	X	132669773	133119922	450150
GPC5	glypican 5	13	92050929	93519490	1468562
GPC6	glypican 6	13	93879095	95059655	1180561
GPCPD1	glycerophosphocholine phosphodiesterase 1	20	5525085	5591672	66588
GPD1L	glycerol-3-phosphate dehydrogenase 1-like	3	32147181	32210205	63025
GPHN	gephyrin	14	66974125	67648520	674396
GPM6A	glycoprotein M6A	4	176554085	176923815	369731
GPM6B	glycoprotein M6B	X	13789150	13956757	167608
GPR132	G protein-coupled receptor 132	14	105515728	105531782	16055
GPR137C	G protein-coupled receptor 137C	14	53019866	53104431	84566
GPR157	G protein-coupled receptor 157	1	9160364	9189229	28866
GPR158	G protein-coupled receptor 158	10	25463991	25891155	427165
GPR180	G protein-coupled receptor 180	13	95254157	95286899	32743
**GPR37**	G protein-coupled receptor 37	7	124386051	124405681	19631
GPR39	G protein-coupled receptor 39	2	133174147	133404132	229986
GPRC5B	G protein-coupled receptor class C group 5 member B	16	19868616	19897489	28874
GPRIN3	GPRIN family member 3	4	90157537	90229161	71625
GPSM2	G protein signaling modulator 2	1	109417972	109477167	59196
GPSM3	G protein signaling modulator 3	6	32158543	32163300	4758
GRB10	growth factor receptor bound protein 10	7	50657760	50861159	203400
**GRB14**	growth factor receptor bound protein 14	2	165349322	165478358	129037
GRB2	growth factor receptor bound protein 2	17	73314157	73401790	87634
GREB1	growth regulation by estrogen in breast cancer 1	2	11674242	11782914	108673
GREB1L	growth regulation by estrogen in breast cancer 1 like	18	18822203	19105378	283176
GRHL2	grainyhead like transcription factor 2	8	102504660	102681954	177295
GRIA1	glutamate ionotropic receptor AMPA type subunit 1	5	152869175	153193429	324255
GRIA3	glutamate ionotropic receptor AMPA type subunit 3	X	122318006	122624766	306761
GRIA4	glutamate ionotropic receptor AMPA type subunit 4	11	105480721	105852819	372099
GRID1	glutamate ionotropic receptor delta type subunit 1	10	87359312	88126250	766939
GRID2	glutamate ionotropic receptor delta type subunit 2	4	93225550	94695707	1470158
**GRIK1**	glutamate ionotropic receptor kainate type subunit 1	21	30909254	31312351	403098
**GRIK2**	glutamate ionotropic receptor kainate type subunit 2	6	101846664	102517958	671295
GRIN1	glutamate ionotropic receptor NMDA type subunit 1	9	140032842	140063207	30366
GRIN2A	glutamate ionotropic receptor NMDA type subunit 2A	16	9852376	10276611	424236
GRIN2B	glutamate ionotropic receptor NMDA type subunit 2B	12	13693165	14133053	439889
GRIN3A	glutamate ionotropic receptor NMDA type subunit 3A	9	104331635	104500862	169228
GRIN3B	glutamate ionotropic receptor NMDA type subunit 3B	19	1000418	1009731	9314
GRIP1	glutamate receptor interacting protein 1	12	66741211	67197966	456756
GRK5	G protein-coupled receptor kinase 5	10	120967101	121215131	248031
GRK6	G protein-coupled receptor kinase 6	5	176830205	176869902	39698
GRM1	glutamate metabotropic receptor 1	6	146348782	146758734	409953
GRM3	glutamate metabotropic receptor 3	7	86273230	86494200	220971
GRM5	glutamate metabotropic receptor 5	11	88237744	88799113	561370
GRM7	glutamate metabotropic receptor 7	3	6811688	7783215	971528
GRM8	glutamate metabotropic receptor 8	7	126078652	126893348	814697
**GRN**	granulin precursor	17	42422614	42430470	7857
GRXCR1	glutaredoxin and cysteine rich domain containing 1	4	42895284	43032675	137392
GSAP	gamma-secretase activating protein	7	76940068	77045717	105650
**GSDMD**	gasdermin D	8	144635377	144645232	9856
GSG1	germ cell associated 1	12	13236494	13256619	20126
GSG1L	GSG1 like	16	27798850	28074830	275981
GSK3B	glycogen synthase kinase 3 beta	3	119540170	119813264	273095
GSPT2	G1 to S phase transition 2	X	51486481	51489324	2844
GSTA4	glutathione S-transferase alpha 4	6	52842751	52860176	17426
GSTCD	glutathione S-transferase C-terminal domain containing	4	106629935	106768885	138951
GSTM1	glutathione S-transferase mu 1	1	110230436	110251661	21226
GSTM3	glutathione S-transferase mu 3	1	110276554	110284384	7831
GSTO1	glutathione S-transferase omega 1	10	105995114	106027217	32104
GSTO2	glutathione S-transferase omega 2	10	106028631	106064703	36073
GSTP1	glutathione S-transferase pi 1	11	67351066	67354131	3066
GSTT1	glutathione S-transferase theta 1	22	24376133	24384680	8548
GSTZ1	glutathione S-transferase zeta 1	14	77787227	77797940	10714
GTDC1	glycosyltransferase like domain containing 1	2	144695635	145090135	394501
GTF2E2	general transcription factor IIE subunit 2	8	30435835	30515768	79934
GTF2H3	general transcription factor IIH subunit 3	12	124118375	124147153	28779
GTF2I	general transcription factor IIi	7	74071994	74175026	103033
GTF3A	general transcription factor IIIA	13	27998681	28009958	11278
GTF3C1	general transcription factor IIIC subunit 1	16	27470876	27561234	90359
GUCY1A2	guanylate cyclase 1 soluble subunit alpha 2	11	106544738	106889250	344513
GUCY1A3	guanylate cyclase 1 soluble subunit alpha	4	156587863	156653501	65639
GUCY2EP	guanylate cyclase 2E, pseudogene	11	76405087	76418685	13599
GUCY2F	guanylate cyclase 2F, retinal	X	108616135	108725301	109167
GULP1	GULP, engulfment adaptor PTB domain containing 1	2	189156396	189460653	304258
GUSBP11	glucuronidase, beta pseudogene 11	22	23980674	24059543	78870

GUSBP3	glucuronidase, beta pseudogene 3	5	68790040	69006341	216302
GYS2	glycogen synthase 2	12	21689123	21757781	68659
H2AFY	H2A histone family member Y	5	134669590	134735604	66015
HABP4	hyaluronan binding protein 4	9	99212483	99253618	41136
HADHB	hydroxyacyl-CoA dehydrogenase/3-ketoacyl-CoA thiolase/enoyl-CoA hydratase (trifunctional protein), beta subunit	2	26466038	26513336	47299
HAND1	heart and neural crest derivatives expressed 1	5	153854532	153857824	3293
HAO1	hydroxyacid oxidase 1	20	7863628	7921121	57494
HAO2	hydroxyacid oxidase 2	1	119911402	119936753	25352
HARS	histidyl-tRNA synthetase	5	140052758	140071609	18852
HAX1	HCLS1 associated protein X-1	1	154244987	154248351	3365
HBB	hemoglobin subunit beta	11	5246694	5250625	3932
HBP1	HMG-box transcription factor 1	7	106809406	106842974	33569
HBS1L	HBS1 like translational GTPase	6	135281516	135424194	142679
HCK	HCK proto-oncogene, Src family tyrosine kinase	20	30639991	30689659	49669
HCN1	hyperpolarization activated cyclic nucleotide gated potassium channel 1	5	45259349	45696253	436905
HCRTR2	hypocretin receptor 2	6	55039050	55147418	108369
HDAC2	histone deacetylase 2	6	114254192	114332472	78281
HDAC4	histone deacetylase 4	2	239969864	240323348	353485
HDAC6	histone deacetylase 6	X	48659784	48683392	23609
HDAC8	histone deacetylase 8	X	71549366	71792953	243588
HDAC9	histone deacetylase 9	7	18126572	19042039	915468
HDLBP	high density lipoprotein binding protein	2	242166679	242256476	89798
HDX	highly divergent homeobox	X	83572882	83757487	184606
HEATR6	HEAT repeat containing 6	17	58120555	58156292	35738
HECTD1	HECT domain E3 ubiquitin protein ligase 1	14	31569318	31677010	107693
HECW1	HECT, C2 and WW domain containing E3 ubiquitin protein ligase 1	7	43152198	43605600	453403
HECW2	HECT, C2 and WW domain containing E3 ubiquitin protein ligase 2	2	197059094	197458416	399323
HELZ	helicase with zinc finger	17	65066554	65242105	175552
HERC1	HECT and RLD domain containing E3 ubiquitin protein ligase family member 1	15	63900817	64126141	225325
HERC2	HECT and RLD domain containing E3 ubiquitin protein ligase 2	15	28356186	28567298	211113
HERC3	HECT and RLD domain containing E3 ubiquitin protein ligase 3	4	89442199	89629693	187495
HERC4	HECT and RLD domain containing E3 ubiquitin protein ligase 4	10	69681665	69835105	153441
**HEXA**	hexosaminidase subunit alpha	15	72635775	72668817	33043
HEXA-AS1	HEXA antisense RNA 1	15	72668454	72671129	2676
**HFE**	hemochromatosis	6	26087509	26098571	11063
HFM1	HFM1, ATP dependent DNA helicase homolog	1	91726323	91870426	144104
HGF	hepatocyte growth factor	7	81328322	81399754	71433
HHAT	hedgehog acyltransferase	1	210501596	210849638	348043
HHEX	hematopoietically expressed homeobox	10	94447945	94455403	7459
HIBADH	3-hydroxyisobutyrate dehydrogenase	7	27565061	27702614	137554
HIF1A	hypoxia inducible factor 1 alpha subunit	14	62162231	62214976	52746
HINT1	histidine triad nucleotide binding protein 1	5	130494720	130507428	12709
HIP1	huntingtin interacting protein 1	7	75162621	75368280	205660
HIPK2	homeodomain interacting protein kinase 2	7	139246316	139477577	231262
HIRA	histone cell cycle regulator	22	19318221	19435224	117004
HIST1H1C	histone cluster 1 H1 family member c	6	26055968	26056699	732
HIST1H2BD	histone cluster 1 H2B family member d	6	26158349	26171577	13229
HIVEP2	human immunodeficiency virus type I enhancer binding protein 2	6	143072604	143266338	193735
HIVEP3	human immunodeficiency virus type I enhancer binding protein 3	1	41972036	42501596	529561
HJURP	Holliday junction recognition protein	2	234742062	234763212	21151
HK1	hexokinase 1	10	71029740	71161638	131899
HLA-A	major histocompatibility complex, class I, A	6	29909037	29913661	4625
HLA-DRA	major histocompatibility complex, class II, DR alpha	6	32407619	32412823	5205
HLA-DRB5	major histocompatibility complex, class II, DR beta 5	6	32485120	32498064	12945
HLCS	holocarboxylase synthetase	21	38123189	38362536	239348
HLF	HLF, PAR bZIP transcription factor	17	53342373	53402426	60054
HMBOX1	homeobox containing 1	8	28747911	28922281	174371
HMCN1	hemicentin 1	1	185703683	186160085	456403
HMCN2	hemicentin 2	9	133046882	133309510	262629
HMG20A	high mobility group 20A	15	77712754	77777949	65196
HMGA2	high mobility group AT-hook 2	12	66217911	66360075	142165
HMGCR	3-hydroxy-3-methylglutaryl-CoA reductase	5	74632154	74657929	25776
HMGCS1	3-hydroxy-3-methylglutaryl-CoA synthase 1	5	43289497	43313614	24118
HMGCS2	3-hydroxy-3-methylglutaryl-CoA synthase 2	1	120290619	120311528	20910
HMGN2P46	high mobility group nucleosomal binding domain 2 pseudogene 46	15	45803334	45878488	75155
HMGN3	high mobility group nucleosomal binding domain 3	6	79910962	79944406	33445
HMGN5	high mobility group nucleosome binding domain 5	X	80369200	80457441	88242
HMGXB4	HMG-box containing 4	22	35653445	35691800	38356
HMMR	hyaluronan mediated motility receptor	5	162887209	162918947	31739
HMOX1	heme oxygenase 1	22	35776354	35790207	13854
HMOX2	heme oxygenase 2	16	4524691	4560348	35658
HNF4A	hepatocyte nuclear factor 4 alpha	20	42984340	43061485	77146
HNMT	histamine N-methyltransferase	2	138721590	138773930	52341
**HNRNPA1**	heterogeneous nuclear ribonucleoprotein A1	12	54673977	54680872	6896

**HNRNPA2B1**	heterogeneous nuclear ribonucleoprotein A2/B1	7	26229547	26241149	11603
HNRNPD	heterogeneous nuclear ribonucleoprotein D	4	83273651	83295656	22006
HNRNPH1	heterogeneous nuclear ribonucleoprotein H1	5	179041179	179061785	20607
HNRNPLL	heterogeneous nuclear ribonucleoprotein L like	2	38789120	38830728	41609
HNRNPU	heterogeneous nuclear ribonucleoprotein U	1	245014468	245027844	13377
HNRNPUL1	heterogeneous nuclear ribonucleoprotein U like 1	19	41768401	41813503	45103
HOMER1	homer scaffolding protein 1	5	78668459	78810040	141582
HOMER2	homer scaffolding protein 2	15	83509838	83654661	144824
HORMAD2	HORMA domain containing 2	22	30476163	30573064	96902
HOXC4	homeobox C4	12	54410715	54449813	39099
HP	haptoglobin	16	72088491	72094954	6464
HPD	4-hydroxyphenylpyruvate dioxygenase	12	122277433	122301502	24070
HPS3	HPS3, biogenesis of lysosomal organelles complex 2 subunit 1	3	148847371	148891519	44149
HPS5	HPS5, biogenesis of lysosomal organelles complex 2 subunit 2	11	18300223	18343745	43523
HPSE2	heparanase 2 (inactive)	10	100218875	100995619	776745
HS1BP3	HCLS1 binding protein 3	2	20760208	20850849	90642
HS2ST1	heparan sulfate 2-O-sulfotransferase 1	1	87380331	87602334	222004
HS3ST3A1	heparan sulfate-glucosamine 3-sulfotransferase 3A1	17	13399006	13505244	106239
HS3ST4	heparan sulfate-glucosamine 3-sulfotransferase 4	16	25703347	26149009	445663
HS6ST1	heparan sulfate 6-O-sulfotransferase 1	2	128994290	129076151	81862
HS6ST3	heparan sulfate 6-O-sulfotransferase 3	13	96743093	97485671	742579
HSD11B1	hydroxysteroid 11-beta dehydrogenase 1	1	209859510	209908295	48786
HSD17B13	hydroxysteroid 17-beta dehydrogenase 13	4	88224941	88244058	19118
HSD17B7P2	hydroxysteroid 17-beta dehydrogenase 7 pseudogene 2	10	38645305	38667433	22129
HSDL1	hydroxysteroid dehydrogenase like 1	16	84155886	84178797	22912
HSF2	heat shock transcription factor 2	6	122720691	122754264	33574
HSF2BP	heat shock transcription factor 2 binding protein	21	44949072	45079374	130303
HSP90AA1	heat shock protein 90 alpha family class A member 1	14	102547075	102606036	58962
**HSPA12B**	heat shock protein family A (Hsp70) member 12B	20	3713314	3733758	20445
HSPA14	heat shock protein family A (Hsp70) member 14	10	14880163	14913740	33578
HSPA1A	heat shock protein family A (Hsp70) member 1A	6	31783291	31785723	2433
HSPA1B	heat shock protein family A (Hsp70) member 1B	6	31795512	31798031	2520
HSPA1L	heat shock protein family A (Hsp70) member 1 like	6	31777396	31783437	6042
HSPA5	heat shock protein family A (Hsp70) member 5	9	127997132	128003609	6478
HSPA8	heat shock protein family A (Hsp70) member 8	11	122928197	122933938	5742
HSPA9	heat shock protein family A (Hsp70) member 9	5	137890571	137911133	20563
HSPB1	heat shock protein family B (small) member 1	7	75931861	75933612	1752
HSPB3	heat shock protein family B (small) member 3	5	53751445	53752207	763
HSPB8	heat shock protein family B (small) member 8	12	119616447	119658936	42490
HSPBAP1	HSPB1 associated protein 1	3	122458846	122512671	53826
HSPD1	heat shock protein family D (Hsp60) member 1	2	198351305	198381461	30157
HSPG2	heparan sulfate proteoglycan 2	1	22148738	22263790	115053
HTR1E	5-hydroxytryptamine receptor 1E	6	87647024	87726349	79326
HTR2A	5-hydroxytryptamine receptor 2A	13	47405685	47471169	65485
HTR2C	5-hydroxytryptamine receptor 2C	X	113818551	114144624	326074
HTR4	5-hydroxytryptamine receptor 4	5	147830595	148056798	226204
HTR6	5-hydroxytryptamine receptor 6	1	19991780	20006055	14276
HTR7	5-hydroxytryptamine receptor 7	10	92500578	92617671	117094
HTRA1	HtrA serine peptidase 1	10	124221041	124274424	53384
HTRA2	HtrA serine peptidase 2	2	74756504	74760472	3969
HTT	huntingtin	4	3076408	3245676	169269
HYAL2	hyaluronoglucosaminidase 2	3	50355221	50360337	5117
HYAL4	hyaluronoglucosaminidase 4	7	123469037	123517532	48496
HYDIN	HYDIN, axonemal central pair apparatus protein	16	70841281	71264625	423345
HYKK	hydroxylysine kinase	15	78799906	78829714	29809
ICA1	islet cell autoantigen 1	7	8152814	8302317	149504
ICA1L	islet cell autoantigen 1 like	2	203640690	203736708	96019
ICAM1	intercellular adhesion molecule 1	19	10381511	10397291	15781
ICK	intestinal cell kinase	6	52866077	52926600	60524
IDE	insulin degrading enzyme	10	94211441	94333833	122393
IDH1	isocitrate dehydrogenase (NADP(+)) 1, cytosolic	2	209100951	209130798	29848
IDH3B	isocitrate dehydrogenase 3 (NAD(+)) beta	20	2639041	2644865	5825
IER3IP1	immediate early response 3 interacting protein 1	18	44661027	44702652	41626
IFI16	interferon gamma inducible protein 16	1	158969758	159024945	55188
IFNG	interferon gamma	12	68548548	68553527	4980
IFT57	intraflagellar transport 57	3	107879659	107941417	61759
IFT88	intraflagellar transport 88	13	21140585	21265503	124919
IGF1	insulin like growth factor 1	12	102789645	102874423	84779
IGF1R	insulin like growth factor 1 receptor	15	99192200	99507759	315560
IGF2	insulin like growth factor 2	11	2150342	2170833	20492
IGF2BP2	insulin like growth factor 2 mRNA binding protein 2	3	185361527	185542844	181318
IGF2BP3	insulin like growth factor 2 mRNA binding protein 3	7	23349828	23510086	160259
IGFBP5	insulin like growth factor binding protein 5	2	217536828	217560248	23421
IGFBP7	insulin like growth factor binding protein 7	4	57896939	57976551	79613
IGHMBP2	immunoglobulin mu binding protein 2	11	68671310	68708070	36761
IGSF11	immunoglobulin superfamily member 11	3	118619404	118864915	245512
IGSF21	immunoglobin superfamily member 21	1	18434240	18704977	270738

IKBKAP	inhibitor of kappa light polypeptide gene enhancer in B-cells, kinase complex-associated protein	9	111629797	111696396	66600
IKZF2	IKAROS family zinc finger 2	2	213864429	214017151	152723
IKZF3	IKAROS family zinc finger 3	17	37921198	38020441	99244
IL10	interleukin 10	1	206940947	206945839	4893
IL12A-AS1	IL12A antisense RNA 1	3	159631189	159943086	311898
IL17A	interleukin 17A	6	52051185	52055436	4252
IL18	interleukin 18	11	112013974	112034840	20867
IL1A	interleukin 1 alpha	2	113531492	113542167	10676
IL1B	interleukin 1 beta	2	113587328	113594480	7153
IL1RAP	interleukin 1 receptor accessory protein	3	190231840	190375843	144004
IL1RAPL1	interleukin 1 receptor accessory protein like 1	X	28605516	29974840	1369325
IL1RAPL2	interleukin 1 receptor accessory protein like 2	X	103810996	105011822	1200827
IL1RL2	interleukin 1 receptor like 2	2	102803433	102856462	53030
IL1RN	interleukin 1 receptor antagonist	2	113864791	113891593	26803
IL2	interleukin 2	4	123372625	123377880	5256
IL20RA	interleukin 20 receptor subunit alpha	6	137321108	137366298	45191
IL24	interleukin 24	1	207070788	207077484	6697
IL33	interleukin 33	9	6215805	6257983	42179
IL4	interleukin 4	5	132009678	132018368	8691
IL6	interleukin 6	7	22765503	22771621	6119
IL6ST	interleukin 6 signal transducer	5	55230923	55290821	59899
ILDR2	immunoglobulin like domain containing receptor 2	1	166882443	166944719	62277
ILKAP	ILK associated serine/threonine phosphatase	2	239079042	239112370	33329
IMMP1L	inner mitochondrial membrane peptidase subunit 1	11	31453948	31531192	77245
IMMP2L	inner mitochondrial membrane peptidase subunit 2	7	110303110	111202573	899464
IMPA1	inositol monophosphatase 1	8	82570196	82598928	28733
IMPDH1	inosine monophosphate dehydrogenase 1	7	128032331	128050306	17976
INHBA	inhibin beta A subunit	7	41724712	41742706	17995
INO80	INO80 complex subunit	15	41271078	41408552	137475
INO80D	INO80 complex subunit D	2	206858445	206951027	92583
INPP4B	inositol polyphosphate-4-phosphatase type II B	4	142944313	143768585	824273
INPP5A	inositol polyphosphate-5-phosphatase A	10	134351324	134596979	245656
INPP5D	inositol polyphosphate-5-phosphatase D	2	233924677	234116549	191873
INPP5F	inositol polyphosphate-5-phosphatase F	10	121485609	121588652	103044
INS-IGF2	INS-IGF2 readthrough	11	2153768	2182439	28672
INSR	insulin receptor	19	7112266	7294045	181780
INTS7	integrator complex subunit 7	1	212113741	212208884	95144
INTS9	integrator complex subunit 9	8	28625178	28747759	122582
INVS	inversin	9	102861538	103063282	201745
**IP6K2**	inositol hexakisphosphate kinase 2	3	48725436	48777786	52351
IPCEF1	interaction protein for cytohesin exchange factors 1	6	154475631	154677926	202296
IPMK	inositol polyphosphate multikinase	10	59951278	60027694	76417
IPO11	importin 11	5	61699799	61924409	224611
IPO5	importin 5	13	98605912	98676551	70640
IPP	intracisternal A particle-promoted polypeptide	1	46159996	46216322	56327
IQCG	IQ motif containing G	3	197615946	197687013	71068
IQCH	IQ motif containing H	15	67547138	67794598	247461
IQCJ	IQ motif containing J	3	158680717	158984096	303380
IQCJ-SCHIP1	IQCJ-SCHIP1 readthrough	3	158680024	159615155	935132
IQGAP2	IQ motif containing GTPase activating protein 2	5	75699074	76003957	304884
IQSEC1	IQ motif and Sec7 domain 1	3	12938719	13114617	175899
IQSEC2	IQ motif and Sec7 domain 2	X	53262058	53350522	88465
IREB2	iron responsive element binding protein 2	15	78729773	78793798	64026
IRF1	interferon regulatory factor 1	5	131817301	131826490	9190
IRF2	interferon regulatory factor 2	4	185308867	185395734	86868
IRF2BP2	interferon regulatory factor 2 binding protein 2	1	234740015	234745271	5257
IRS1	insulin receptor substrate 1	2	227596033	227664475	68443
ISL1	ISL LIM homeobox 1	5	50678921	50690564	11644
ISPD	isoprenoid synthase domain containing	7	16130817	16460947	330131
ISY1-RAB43	ISY1-RAB43 readthrough	3	128806418	128879879	73462
ITFG1	integrin alpha FG-GAP repeat containing 1	16	47188298	47498060	309763
ITGA11	integrin subunit alpha 11	15	68594050	68724501	130452
ITGA2	integrin subunit alpha 2	5	52285156	52390609	105454
ITGA4	integrin subunit alpha 4	2	182321929	182400914	78986
ITGA6	integrin subunit alpha 6	2	173292082	173371181	79100
ITGA8	integrin subunit alpha 8	10	15555948	15762124	206177
ITGA9	integrin subunit alpha 9	3	37493606	37865005	371400
ITGAE	integrin subunit alpha E	17	3617922	3704537	86616
ITGB2	integrin subunit beta 2	21	46305868	46351904	46037
ITGB5	integrin subunit beta 5	3	124480795	124620265	139471
**ITGB6**	integrin subunit beta 6	2	160956177	161128399	172223
ITIH4	inter-alpha-trypsin inhibitor heavy chain family member 4	3	52846991	52865495	18505
ITM2A	integral membrane protein 2A	X	78615881	78623164	7284
ITPK1	inositol-tetrakisphosphate 1-kinase	14	93403259	93582665	179407
ITPKB	inositol-trisphosphate 3-kinase B	1	226819391	226927024	107634
ITPR1	inositol 1,4,5-trisphosphate receptor type 1	3	4535032	4889524	354493

**ITPR2**	inositol 1,4,5-trisphosphate receptor type 2	12	26490342	26986131	495790
ITSN2	intersectin 2	2	24425733	24583583	157851
IVD	isovaleryl-CoA dehydrogenase	15	40697686	40728146	30461
IZUMO2	IZUMO family member 2	19	50655805	50666452	10648
JAK1	Janus kinase 1	1	65298912	65432187	133276
JAKMIP1	janus kinase and microtubule interacting protein 1	4	6027926	6202318	174393
JAKMIP3	Janus kinase and microtubule interacting protein 3	10	133918175	133998313	80139
JARID2	jumonji and AT-rich interaction domain containing 2	6	15246527	15522252	275726
JAZF1	JAZF zinc finger 1	7	27870192	28220362	350171
JAZF1-AS1	JAZF1 antisense RNA 1	7	28219941	28283536	63596
JMJD1C	jumonji domain containing 1C	10	64926981	65225722	298742
JPX	JPX transcript, XIST activator (non-protein coding)	X	73164159	73290243	126085
KALRN	kalirin, RhoGEF kinase	3	123798870	124445172	646303
KANK2	KN motif and ankyrin repeat domains 2	19	11274943	11308467	33525
KANSL1	KAT8 regulatory NSL complex subunit 1	17	44107282	44302733	195452
KANSL3	KAT8 regulatory NSL complex subunit 3	2	97258907	97308524	49618
KARS	lysyl-tRNA synthetase	16	75661622	75682541	20920
KAT2B	lysine acetyltransferase 2B	3	20081515	20195896	114382
KAT5	lysine acetyltransferase 5	11	65479467	65487075	7609
KAT6A	lysine acetyltransferase 6A	8	41786997	41909508	122512
KAT6B	lysine acetyltransferase 6B	10	76585340	76792380	207041
KATNAL1	katanin catalytic subunit A1 like 1	13	30776767	30881621	104855
KAZN	kazrin, periplakin interacting protein	1	14925200	15444539	519340
KBTBD2	kelch repeat and BTB domain containing 2	7	32907784	32933743	25960
KBTBD3	kelch repeat and BTB domain containing 3	11	105921825	105948492	26668
KCNA1	potassium voltage-gated channel subfamily A member 1	12	5019071	5040527	21457
KCNAB1	potassium voltage-gated channel subfamily A member regulatory beta subunit 1	3	155755490	156256545	501056
KCNB1	potassium voltage-gated channel subfamily B member 1	20	47980414	48099184	118771
**KCNB2**	potassium voltage-gated channel subfamily B member 2	8	73449626	73850584	400959
KCNC2	potassium voltage-gated channel subfamily C member 2	12	75433857	75603648	169792
KCNC3	potassium voltage-gated channel subfamily C member 3	19	50815194	50836772	21579
KCND2	potassium voltage-gated channel subfamily D member 2	7	119913722	120390385	476664
KCND3	potassium voltage-gated channel subfamily D member 3	1	112313284	112531777	218494
KCNH1	potassium voltage-gated channel subfamily H member 1	1	210856555	211307457	450903
KCNH2	potassium voltage-gated channel subfamily H member 2	7	150642049	150675403	33355
KCNH5	potassium voltage-gated channel subfamily H member 5	14	63173287	63568755	395469
KCNH7	potassium voltage-gated channel subfamily H member 7	2	163227917	163695240	467324
KCNH8	potassium voltage-gated channel subfamily H member 8	3	19189946	19577138	387193
KCNIP1	potassium voltage-gated channel interacting protein 1	5	169780491	170163636	383146
KCNIP3	potassium voltage-gated channel interacting protein 3	2	95963052	96051825	88774
**KCNIP4**	potassium voltage-gated channel interacting protein 4	4	20730239	21950422	1220184
KCNJ10	potassium voltage-gated channel subfamily J member 10	1	160007257	160040038	32782
KCNJ11	potassium voltage-gated channel subfamily J member 11	11	17407406	17410878	3473
KCNJ12	potassium voltage-gated channel subfamily J member 12	17	21279509	21323179	43671
KCNJ5	potassium voltage-gated channel subfamily J member 5	11	128761251	128790930	29680
KCNJ6	potassium voltage-gated channel subfamily J member 6	21	38979678	39288749	309072
KCNJ8	potassium voltage-gated channel subfamily J member 8	12	21917889	21928515	10627
KCNK10	potassium two pore domain channel subfamily K member 10	14	88649113	88793251	144139
KCNK12	potassium two pore domain channel subfamily K member 12	2	47743720	47798078	54359
KCNK9	potassium two pore domain channel subfamily K member 9	8	140613081	140715299	102219
KCNMA1	potassium calcium-activated channel subfamily M alpha 1	10	78629359	79398353	768995
KCNN1	potassium calcium-activated channel subfamily N member 1	19	18062102	18110889	48788
KCNN2	potassium calcium-activated channel subfamily N member 2	5	113696642	113832337	135696
KCNN3	potassium calcium-activated channel subfamily N member 3	1	154669931	154842756	172826
KCNQ1	potassium voltage-gated channel subfamily Q member 1	11	2465914	2870339	404426
KCNQ2	potassium voltage-gated channel subfamily Q member 2	20	62037542	62103993	66452
**KCNQ3**	potassium voltage-gated channel subfamily Q member 3	8	133133108	133493200	360093
KCNQ4	potassium voltage-gated channel subfamily Q member 4	1	41249684	41306124	56441
**KCNQ5**	potassium voltage-gated channel subfamily Q member 5	6	73331520	73908574	577055
KCNT1	potassium sodium-activated channel subfamily T member 1	9	138594031	138684992	90962
KCNT2	potassium sodium-activated channel subfamily T member 2	1	196194909	196578355	383447
KCNU1	potassium calcium-activated channel subfamily U member 1	8	36641842	36793646	151805
KCTD1	potassium channel tetramerization domain containing 1	18	24034874	24237365	202492
KCTD16	potassium channel tetramerization domain containing 16	5	143550396	143865249	314854
KCTD7	potassium channel tetramerization domain containing 7	7	66093868	66276446	182579
KCTD8	potassium channel tetramerization domain containing 8	4	44175926	44450824	274899
KDELR2	KDEL endoplasmic reticulum protein retention receptor 2	7	6485584	6523873	38290
KDM1B	lysine demethylase 1B	6	18155560	18224084	68525
KDM2A	lysine demethylase 2A	11	66886740	67025558	138819
KDM4C	lysine demethylase 4C	9	6720863	7175648	454786
KDM5B	lysine demethylase 5B	1	202696526	202778598	82073
KDM5C	lysine demethylase 5C	X	53220503	53254604	34102
KDM5D	lysine demethylase 5D	Y	21865751	21906825	41075
KDM6A	lysine demethylase 6A	X	44732757	44971847	239091
**KDR**	kinase insert domain receptor	4	55944644	55991756	47113
KHDRBS2	KH RNA binding domain containing, signal transduction associated 2	6	62389865	62996132	606268

KHDRBS3	KH RNA binding domain containing, signal transduction associated 3	8	136469700	136668965	199266
KIAA0232	KIAA0232	4	6783102	6885897	102796
KIAA0319	KIAA0319	6	24544332	24646383	102052
KIAA0319L	KIAA0319 like	1	35899091	36023551	124461
KIAA0355	KIAA0355	19	34745442	34846491	101050
KIAA0368	KIAA0368	9	114122972	114247025	124054
KIAA0408	KIAA0408	6	127761488	127780536	19049
KIAA0586	KIAA0586	14	58894103	59015216	121114
KIAA0753	KIAA0753	17	6481468	6544247	62780
KIAA0825	KIAA0825	5	93488671	93954309	465639
KIAA0922	KIAA0922	4	154387498	154557863	170366
KIAA1024	KIAA1024	15	79724858	79764632	39775
KIAA1147	KIAA1147	7	141356528	141401953	45426
KIAA1191	KIAA1191	5	175773064	175788971	15908
KIAA1211	KIAA1211	4	57036361	57194791	158431
KIAA1211L	KIAA1211 like	2	99410309	99552722	142414
KIAA1217	KIAA1217	10	23983675	24836772	853098
KIAA1324L	KIAA1324 like	7	86506222	86689015	182794
KIAA1328	KIAA1328	18	34409069	34812135	403067
KIAA1429	KIAA1429	8	95499921	95565757	65837
KIAA1462	KIAA1462	10	30301729	30404423	102695
KIAA1468	KIAA1468	18	59854491	59974355	119865
KIAA1549	KIAA1549	7	138516126	138666064	149939
KIAA1671	KIAA1671	22	25348697	25593415	244719
KIAA1958	KIAA1958	9	115249127	115431677	182551
KIAA2026	KIAA2026	9	5881596	6007901	126306
KIF11	kinesin family member 11	10	94353043	94415150	62108
KIF13A	kinesin family member 13A	6	17759414	17987854	228441
KIF13B	kinesin family member 13B	8	28924796	29120641	195846
KIF16B	kinesin family member 16B	20	16252749	16554078	301330
KIF1A	kinesin family member 1A	2	241653181	241759725	106545
KIF1B	kinesin family member 1B	1	10270863	10441661	170799
KIF1C	kinesin family member 1C	17	4901243	4931696	30454
KIF21B	kinesin family member 21B	1	200938520	200992828	54309
KIF25	kinesin family member 25	6	168396921	168445769	48849
KIF26B	kinesin family member 26B	1	245318287	245872733	554447
KIF27	kinesin family member 27	9	86451613	86536342	84730
KIF2A	kinesin family member 2A	5	61601989	61833076	231088
KIF3A	kinesin family member 3A	5	132028320	132073330	45011
KIF4A	kinesin family member 4A	X	69509879	69640682	130804
KIF5A	kinesin family member 5A	12	57943781	57980415	36635
KIF5C	kinesin family member 5C	2	149632819	149883273	250455
KIF6	kinesin family member 6	6	39297766	39693181	395416
KIF7	kinesin family member 7	15	90152020	90198682	46663
**KIFAP3**	kinesin associated protein 3	1	169890467	170054349	163883
KIN	Kin17 DNA and RNA binding protein	10	7792925	7829990	37066
KIRREL3	kin of IRRE like 3 (Drosophila)	11	126293254	126873355	580102
KLC1	kinesin light chain 1	14	104028233	104167888	139656
KLF12	Kruppel like factor 12	13	74260226	74708394	448169
KLF5	Kruppel like factor 5	13	73629114	73651676	22563
KLF7	Kruppel like factor 7	2	207938861	208031991	93131
KLHL1	kelch like family member 1	13	70274726	70682591	407866
KLHL13	kelch like family member 13	X	117031776	117251303	219528
KLHL14	kelch like family member 14	18	30252634	30353025	100392
KLHL24	kelch like family member 24	3	183353356	183402265	48910
KLHL28	kelch like family member 28	14	45393522	45511525	118004
KLHL29	kelch like family member 29	2	23608088	23931481	323394
KLHL32	kelch like family member 32	6	97372605	97588630	216026
KLHL4	kelch like family member 4	X	86772752	86925050	152299
KLHL41	kelch like family member 41	2	170366212	170382772	16561
KLHL9	kelch like family member 9	9	21329670	21335379	5710
KLKB1	kallikrein B1	4	187130133	187179625	49493
KMT2C	lysine methyltransferase 2C	7	151832010	152133090	301081
KMT2D	lysine methyltransferase 2D	12	49412758	49453557	40800
KNSTRN	kinetochore localized astrin/SPAG5 binding protein	15	40674922	40686447	11526
KPNA3	karyopherin subunit alpha 3	13	50273447	50367057	93611
KRBOX4	KRAB box domain containing 4	X	46306292	46356857	50566
KRT23	keratin 23	17	39078948	39093886	14939
KRT4	keratin 4	12	53200333	53208335	8003
KRT75	keratin 75	12	52817854	52828309	10456
KRTAP5-AS1	KRTAP5-1/KRTAP5-2 antisense RNA 1	11	1592583	1620414	27832
KSR1	kinase suppressor of ras 1	17	25783670	25953461	169792
KSR2	kinase suppressor of ras 2	12	117890817	118406788	515972
KTN1	kinectin 1	14	56025790	56168244	142455
KY	kyphoscoliosis peptidase	3	134321980	134370478	48499
KYNU	kynureninase	2	143635067	143799890	164824
L1CAM	L1 cell adhesion molecule	X	153126969	153174677	47709

L2HGDH	L-2-hydroxyglutarate dehydrogenase	14	50704281	50779266	74986
L3MBTL4	l(3)mbt-like 4 (Drosophila)	18	5954705	6415236	460532
LAMA2	laminin subunit alpha 2	6	129204342	129837714	633373
**LAMA3**	laminin subunit alpha 3	18	21269407	21535030	265624
LAMA4	laminin subunit alpha 4	6	112429963	112576141	146179
LAMB4	laminin subunit beta 4	7	107663993	107770801	106809
LAMC3	laminin subunit gamma 3	9	133884469	133969860	85392
LANCL3	LanC like 3	X	37430822	37543716	112895
LARP1B	La ribonucleoprotein domain family member 1B	4	128982423	129144086	161664
LARP4B	La ribonucleoprotein domain family member 4B	10	855484	977564	122081
LARS2	leucyl-tRNA synthetase 2, mitochondrial	3	45429998	45590913	160916
LAS1L	LAS1 like, ribosome biogenesis factor	X	64732462	64754655	22194
LCA5L	LCA5L, lebercilin like	21	40777770	40817731	39962
LCOR	ligand dependent nuclear receptor corepressor	10	98592017	98740800	148784
LCORL	ligand dependent nuclear receptor corepressor like	4	17842822	18023499	180678
LDB2	LIM domain binding 2	4	16503164	16900432	397269
LDLR	low density lipoprotein receptor	19	11200038	11244492	44455
LDLRAD2	low density lipoprotein receptor class A domain containing 2	1	22138758	22151714	12957
LDLRAD3	low density lipoprotein receptor class A domain containing 3	11	35965531	36253686	288156
LDLRAD4	low density lipoprotein receptor class A domain containing 4	18	13217497	13652754	435258
LDLRAP1	low density lipoprotein receptor adaptor protein 1	1	25870071	25895377	25307
LEF1	lymphoid enhancer binding factor 1	4	108968701	109090112	121412
LEKR1	leucine, glutamate and lysine rich 1	3	156543270	156763918	220649
LEPR	leptin receptor	1	65886248	66107242	220995
LETMD1	LETM1 domain containing 1	12	51441745	51454207	12463
LEUTX	leucine twenty homeobox	19	40267235	40276775	9541
LGALS3	galectin 3	14	55590828	55612126	21299
LGALSL	galectin like	2	64681103	64688515	7413
LGMN	legumain	14	93170152	93215047	44896
LGR4	leucine rich repeat containing G protein-coupled receptor 4	11	27387508	27494322	106815
LGR6	leucine rich repeat containing G protein-coupled receptor 6	1	202163029	202288909	125881
LGSN	lengsin, lens protein with glutamine synthetase domain	6	63985856	64029882	44027
**LHFP**	lipoma HMGIC fusion partner	13	39917029	40177665	260637
LHFPL2	lipoma HMGIC fusion partner-like 2	5	77781038	78065844	284807
LHFPL3	lipoma HMGIC fusion partner-like 3	7	103969104	104549001	579898
LHPP	phospholysine phosphohistidine inorganic pyrophosphate phosphatase	10	126150403	126306457	156055
**LIF**	LIF, interleukin 6 family cytokine	22	30636436	30642840	6405
LIG4	DNA ligase 4	13	108859787	108870716	10930
LIMCH1	LIM and calponin homology domains 1	4	41361624	41702061	340438
LIMS1	LIM zinc finger domain containing 1	2	109150857	109303702	152846
LIMS2	LIM zinc finger domain containing 2	2	128395956	128439360	43405
LIN52	lin-52 DREAM MuvB core complex component	14	74551499	74667936	116438
LIN54	lin-54 DREAM MuvB core complex component	4	83831126	83934079	102954
LIN7A	lin-7 homolog A, crumbs cell polarity complex component	12	81186299	81331704	145406
LIN9	lin-9 DREAM MuvB core complex component	1	226418850	226497570	78721
LINC00158	long intergenic non-protein coding RNA 158	21	26758133	26804013	45881
LINC00189	long intergenic non-protein coding RNA 189	21	30565801	30660526	94726
LINC00210	long intergenic non-protein coding RNA 210	1	218066242	218094146	27905
LINC00271	long intergenic non-protein coding RNA 271	6	135818489	136037193	218705
LINC00301	long intergenic non-protein coding RNA 301	11	60383209	60454622	71414
LINC00305	long intergenic non-protein coding RNA 305	18	61747243	61816264	69022
LINC00333	long intergenic non-protein coding RNA 333	13	85136499	85137371	873
LINC00375	long intergenic non-protein coding RNA 375	13	85639222	85653055	13834
LINC00376	long intergenic non-protein coding RNA 376	13	63757234	63801189	43956
LINC00379	long intergenic non-protein coding RNA 379	13	91779867	91783661	3795
LINC00381	long intergenic non-protein coding RNA 381	13	74993310	75009296	15987
LINC00382	long intergenic non-protein coding RNA 382	13	80446721	80492079	45359
LINC00395	long intergenic non-protein coding RNA 395	13	64241814	64312151	70338
LINC00400	long intergenic non-protein coding RNA 400	13	43732767	43733602	836
LINC00403	long intergenic non-protein coding RNA 403	13	112761433	112762329	897
LINC00448	long intergenic non-protein coding RNA 448	13	63246418	63306492	60075
LINC00457	long intergenic non-protein coding RNA 457	13	35009587	35214822	205236
LINC00466	long intergenic non-protein coding RNA 466	1	63655660	63782902	127243
LINC00494	long intergenic non-protein coding RNA 494	20	46988693	46999372	10680
LINC00499	long intergenic non-protein coding RNA 499	4	139230865	139345498	114634
LINC00504	long intergenic non-protein coding RNA 504	4	14472089	14889793	417705
LINC00508	long intergenic non-protein coding RNA 508	12	128425140	128434705	9566
LINC00535	long intergenic non-protein coding RNA 535	8	94225531	94712661	487131
LINC00536	long intergenic non-protein coding RNA 536	8	116962736	117337297	374562
LINC00598	long intergenic non-protein coding RNA 598	13	41025131	41055143	30013
LINC00607	long intergenic non-protein coding RNA 607	2	216476286	216708445	232160
LINC00616	long intergenic non-protein coding RNA 616	4	138948576	139051863	103288
LINC00620	long intergenic non-protein coding RNA 620	3	13692196	13788132	95937
LINC00630	long intergenic non-protein coding RNA 630	X	102024089	102140334	116246
LINC00639	long intergenic non-protein coding RNA 639	14	39218545	39417477	198933
LINC00648	long intergenic non-protein coding RNA 648	14	48234157	48264295	30139

LINC00866	long intergenic non-protein coding RNA 866	10	99588235	99609555	21321
LINC00871	long intergenic non-protein coding RNA 871	14	46410146	46971026	560881
LINC00877	long intergenic non-protein coding RNA 877	3	72084451	72291716	207266
LINC00882	long intergenic non-protein coding RNA 882	3	106555658	106959488	403831
LINC00894	long intergenic non-protein coding RNA 894	X	149106846	149392815	285970
LINC00907	long intergenic non-protein coding RNA 907	18	39739247	40271387	532141
LINC00910	long intergenic non-protein coding RNA 910	17	41447213	41466567	19355
LINC00922	long intergenic non-protein coding RNA 922	16	65318402	65610203	291802
LINC00955	long intergenic non-protein coding RNA 955	4	3578596	3592438	13843
LINC00959	long intergenic non-protein coding RNA 959	10	131864638	131909081	44444
LINC00971	long intergenic non-protein coding RNA 971	3	84687557	84930830	243274
LINC00989	long intergenic non-protein coding RNA 989	4	80413570	80497614	84045
LINC00992	long intergenic non-protein coding RNA 992	5	116751205	116881993	130789
LINC00993	long intergenic non-protein coding RNA 993	10	37598113	37635959	37847
LINC01016	long intergenic non-protein coding RNA 1016	6	33835283	33864691	29409
LINC01021	long intergenic non-protein coding RNA 1021	5	27472399	27496508	24110
LINC01036	long intergenic non-protein coding RNA 1036	1	187061974	187329455	267482
LINC01060	long intergenic non-protein coding RNA 1060	4	189321890	189602205	280316
LINC01087	long intergenic non-protein coding RNA 1087	2	132394598	132407188	12591
LINC01088	long intergenic non-protein coding RNA 1088	4	79892902	80229952	337051
LINC01091	long intergenic non-protein coding RNA 1091	4	124571422	124851561	280140
LINC01098	long intergenic non-protein coding RNA 1098	4	178649911	178911904	261994
LINC01122	long intergenic non-protein coding RNA 1122	2	58654934	59290901	635968
LINC01135	long intergenic non-protein coding RNA 1135	1	59250823	59365384	114562
LINC01170	long intergenic non-protein coding RNA 1170	5	123641923	123740772	98850
LINC01284	long intergenic non-protein coding RNA 1284	X	50844121	50844465	345
LINC01411	long intergenic non-protein coding RNA 1411	5	173953231	173954142	912
LINC01568	long intergenic non-protein coding RNA 1568	16	73420704	73455295	34592
LINC01572	long intergenic non-protein coding RNA 1572	16	72302913	72324506	21594
LINC01605	long intergenic non-protein coding RNA 1605	8	37263982	37279087	15106
LINC-PINT	long intergenic non-protein coding RNA, p53 induced transcript	7	130626519	130794935	168417
LINGO1	leucine rich repeat and Ig domain containing 1	15	77905369	78113242	207874
LINGO2	leucine rich repeat and Ig domain containing 2	9	27948076	28670283	722208
LIPA	lipase A, lysosomal acid type	10	90973326	91174314	200989
**LIPC**	lipase C, hepatic type	15	58702768	58861151	158384
LIPE-AS1	LIPE antisense RNA 1	19	42901280	43156507	255228
LIPJ	lipase family member J	10	90346510	90366733	20224
LIPM	lipase family member M	10	90562487	90580303	17817
LITAF	lipopolysaccharide induced TNF factor	16	11641853	11730237	88385
LMBRD2	LMBR1 domain containing 2	5	36098514	36152063	53550
LMCD1	LIM and cysteine rich domains 1	3	8543393	8609805	66413
LMCD1-AS1	LMCD1 antisense RNA 1 (head to head)	3	7994492	8653610	659119
LMF1	lipase maturation factor 1	16	903634	1031318	127685
LMNA	lamin A/C	1	156052364	156109880	57517
**LMNB1**	lamin B1	5	126112315	126172712	60398
LMO2	LIM domain only 2	11	33880122	33913836	33715
LMO7	LIM domain 7	13	76194570	76434004	239435
LMTK2	lemur tyrosine kinase 2	7	97736197	97838945	102749
LMX1A	LIM homeobox transcription factor 1 alpha	1	165171104	165325952	154849
LMX1B	LIM homeobox transcription factor 1 beta	9	129376722	129463311	86590
LNX1-AS2	LNX1 antisense RNA 2	4	54459123	54470214	11092
LNX2	ligand of numb-protein X 2	13	28120050	28194541	74492
LONRF2	LON peptidase N-terminal domain and ring finger 2	2	100889753	100939195	49443
**LOX**	lysyl oxidase	5	121398890	121413980	15091
LOXHD1	lipoxygenase homology domains 1	18	44056935	44236996	180062
LPA	lipoprotein(a)	6	160952515	161087407	134893
LPAR5	lysophosphatidic acid receptor 5	12	6728001	6745613	17613
LPCAT4	lysophosphatidylcholine acyltransferase 4	15	34651106	34659479	8374
LPIN2	lipin 2	18	2916992	3013313	96322
LPL	lipoprotein lipase	8	19759228	19824769	65542
LPP	LIM domain containing preferred translocation partner in lipoma	3	187871072	188608460	737389
LPXN	leupaxin	11	58294344	58345693	51350
LRAT	lecithin retinol acyltransferase (phosphatidylcholine--retinol O-acyltransferase)	4	155548097	155674270	126174
LRBA	LPS responsive beige-like anchor protein	4	151185594	151936879	751286
LRCH1	leucine rich repeats and calponin homology domain containing 1	13	47127303	47327175	199873
LRCH2	leucine rich repeats and calponin homology domain containing 2	X	114345185	114468635	123451
LRFN5	leucine rich repeat and fibronectin type III domain containing 5	14	42076773	42373752	296980
LRIG1	leucine rich repeats and immunoglobulin like domains 1	3	66429221	66551687	122467
LRMP	lymphoid restricted membrane protein	12	25173936	25261268	87333
LRP1	LDL receptor related protein 1	12	57522276	57607134	84859
LRP12	LDL receptor related protein 12	8	105501459	105601252	99794
LRP1B	LDL receptor related protein 1B	2	140988992	142889270	1900279
LRP2	LDL receptor related protein 2	2	169983619	170219195	235577
LRP6	LDL receptor related protein 6	12	12268959	12419946	150988
LRP8	LDL receptor related protein 8	1	53711217	53793742	82526
LRPAP1	LDL receptor related protein associated protein 1	4	3508103	3534286	26184

LRRC1	leucine rich repeat containing 1	6	53659295	53788919	129625
LRRC37B	leucine rich repeat containing 37B	17	30334891	30380523	45633
LRRC37BP1	leucine rich repeat containing 37B pseudogene 1	17	28956698	28964482	7785
LRRC4C	leucine rich repeat containing 4C	11	40135753	41481323	1345571
LRRC69	leucine rich repeat containing 69	8	92114060	92231464	117405
LRRC7	leucine rich repeat containing 7	1	70034081	70617628	583548
LRRFIP1	LRR binding FLII interacting protein 1	2	238536219	238722325	186107
LRRFIP2	LRR binding FLII interacting protein 2	3	37094117	37225180	131064
LRRIQ1	leucine rich repeats and IQ motif containing 1	12	85430092	85657002	226911
LRRIQ3	leucine rich repeats and IQ motif containing 3	1	74491699	74663871	172173
LRRK1	leucine rich repeat kinase 1	15	101459420	101610317	150898
LRRK2	leucine rich repeat kinase 2	12	40590546	40763087	172542
LRRTM4	leucine rich repeat transmembrane neuronal 4	2	76974845	77820445	845601
LRSAM1	leucine rich repeat and sterile alpha motif containing 1	9	130213765	130265780	52016
LRTOMT	leucine rich transmembrane and O-methyltransferase domain containing	11	71791382	71821828	30447
LRWD1	leucine rich repeats and WD repeat domain containing 1	7	102105376	102113615	8240
LSAMP	limbic system-associated membrane protein	3	115521235	117716095	2194861
LSM10	LSM10, U7 small nuclear RNA associated	1	36856839	36863493	6655
LSM14B	LSM family member 14B	20	60697517	60710434	12918
LTA	lymphotoxin alpha	6	31539831	31542101	2271
LTA4H	leukotriene A4 hydrolase	12	96394606	96437298	42693
LTBP1	latent transforming growth factor beta binding protein 1	2	33172039	33624576	452538
LTBR	lymphotoxin beta receptor	12	6484211	6500733	16523
LUC7L2	LUC7 like 2, pre-mRNA splicing factor	7	139026106	139107345	81240
**LUM**	lumican	12	91496406	91505608	9203
LUZP2	leucine zipper protein 2	11	24518516	25104150	585635
LY86	lymphocyte antigen 86	6	6588341	6655216	66876
LYAR	Ly1 antibody reactive	4	4269428	4291896	22469
LYN	LYN proto-oncogene, Src family tyrosine kinase	8	56792372	56923940	131569
LYPD5	LY6/PLAUR domain containing 5	19	44300081	44331358	31278
LYPD6B	LY6/PLAUR domain containing 6B	2	149894621	150071776	177156
LYST	lysosomal trafficking regulator	1	235824341	236046940	222600
M6PR	mannose-6-phosphate receptor, cation dependent	12	9092959	9102551	9593
MACF1	microtubule-actin crosslinking factor 1	1	39546988	39952849	405862
MACROD2	MACRO domain containing 2	20	13976015	16033842	2057828
MAD1L1	MAD1 mitotic arrest deficient like 1	7	1855429	2272878	417450
MAGEA11	MAGE family member A11	X	148769894	148798926	29033
MAGI1	membrane associated guanylate kinase, WW and PDZ domain containing 1	3	65339200	66024509	685310
MAGI2	membrane associated guanylate kinase, WW and PDZ domain containing 2	7	77646393	79082890	1436498
MAGI3	membrane associated guanylate kinase, WW and PDZ domain containing 3	1	113933371	114228545	295175
MAGT1	magnesium transporter 1	X	77081861	77151090	69230
MAK	male germ cell associated kinase	6	10762956	10838764	75809
MALRD1	MAM and LDL receptor class A domain containing 1	10	19492779	20079330	586552
MAMDC2	MAM domain containing 2	9	72658497	72841886	183390
MAML2	mastermind like transcriptional coactivator 2	11	95709762	96076344	366583
MAML3	mastermind like transcriptional coactivator 3	4	140637907	141075338	437432
MAN1A1	mannosidase alpha class 1A member 1	6	119498374	119670926	172553
MAN1A2	mannosidase alpha class 1A member 2	1	117910071	118071494	161424
MAN1B1	mannosidase alpha class 1B member 1	9	139981379	140003635	22257
MAN1C1	mannosidase alpha class 1C member 1	1	25943959	26112698	168740
MAOA	monoamine oxidase A	X	43515467	43606068	90602
**MAOB**	monoamine oxidase B	X	43625858	43741693	115836
MAP1LC3B2	microtubule associated protein 1 light chain 3 beta 2	12	116997186	117014425	17240
MAP2	microtubule associated protein 2	2	210288782	210598842	310061
MAP2K1	mitogen-activated protein kinase kinase 1	15	66679155	66784650	105496
MAP2K5	mitogen-activated protein kinase kinase 5	15	67835047	68099461	264415
MAP2K6	mitogen-activated protein kinase kinase 6	17	67410839	67539472	128634
MAP2K7	mitogen-activated protein kinase kinase 7	19	7968728	7979363	10636
MAP3K13	mitogen-activated protein kinase kinase kinase 13	3	185000729	185206885	206157
MAP3K14	mitogen-activated protein kinase kinase kinase 14	17	43340488	43394414	53927
MAP3K19	mitogen-activated protein kinase kinase kinase 19	2	135722061	135805038	82978
MAP3K5	mitogen-activated protein kinase kinase kinase 5	6	136878185	137113656	235472
MAP3K7	mitogen-activated protein kinase kinase kinase 7	6	91223292	91296764	73473
MAP3K9	mitogen-activated protein kinase kinase kinase 9	14	71189243	71276251	87009
MAP4K3	mitogen-activated protein kinase kinase kinase kinase 3	2	39476407	39664453	188047
MAP4K4	mitogen-activated protein kinase kinase kinase kinase 4	2	102313312	102511149	197838
MAP4K5	mitogen-activated protein kinase kinase kinase kinase 5	14	50885219	51027844	142626
MAP6	microtubule associated protein 6	11	75297963	75380165	82203
MAP7	microtubule associated protein 7	6	136663875	136871957	208083
MAP7D2	MAP7 domain containing 2	X	20024831	20135035	110205
MAPK1	mitogen-activated protein kinase 1	22	22108789	22221970	113182
MAPK10	mitogen-activated protein kinase 10	4	86936276	87515284	579009
MAPK4	mitogen-activated protein kinase 4	18	48086448	48258194	171747

MAPK8IP1	mitogen-activated protein kinase 8 interacting protein 1	11	45907202	45928016	20815
MAPK9	mitogen-activated protein kinase 9	5	179660143	179719099	58957
MAPKAP1	mitogen-activated protein kinase associated protein 1	9	128199672	128469513	269842
MAPKAPK5	mitogen-activated protein kinase-activated protein kinase 5	12	112279782	112334343	54562
MAPRE1	microtubule associated protein RP/EB family member 1	20	31407699	31438211	30513
MAPRE2	microtubule associated protein RP/EB family member 2	18	32556892	32723434	166543
**MAPT**	microtubule associated protein tau	17	43971748	44105700	133953
MAPT-AS1	MAPT antisense RNA 1	17	43921017	43972966	51950
MARK1	microtubule affinity regulating kinase 1	1	220701568	220837803	136236
MARK2	microtubule affinity regulating kinase 2	11	63606400	63678491	72092
MARK3	microtubule affinity regulating kinase 3	14	103851729	103970168	118440
MARS2	methionyl-tRNA synthetase 2, mitochondrial	2	198570087	198573113	3027
MARVELD2	MARVEL domain containing 2	5	68710939	68740157	29219
MAST2	microtubule associated serine/threonine kinase 2	1	46252659	46501796	249138
MAST4	microtubule associated serine/threonine kinase family member 4	5	65892176	66465423	573248
MAT2A	methionine adenosyltransferase 2A	2	85766288	85772403	6116
**MATR3**	matrin 3	5	138609441	138667360	57920
MAX	MYC associated factor X	14	65472892	65569413	96522
MB21D2	Mab-21 domain containing 2	3	192514604	192635950	121347
MBD5	methyl-CpG binding domain protein 5	2	148778580	149275805	497226
MBL2	mannose binding lectin 2	10	54525140	54531460	6321
MBNL1	muscleblind like splicing regulator 1	3	151961617	152183569	221953
MBNL2	muscleblind like splicing regulator 2	13	97873688	98046374	172687
MBNL3	muscleblind like splicing regulator 3	X	131503345	131623996	120652
MBOAT2	membrane bound O-acyltransferase domain containing 2	2	8992820	9143942	151123
MBTPS2	membrane bound transcription factor peptidase, site 2	X	21857754	21903542	45789
MC1R	melanocortin 1 receptor	16	89978527	89987385	8859
MCC	mutated in colorectal cancers	5	112357796	112824527	466732
MCCC1	methylcrotonoyl-CoA carboxylase 1	3	182733006	182833863	100858
MCF2	MCF.2 cell line derived transforming sequence	X	138663929	138790386	126458
MCF2L2	MCF.2 cell line derived transforming sequence-like 2	3	182895831	183146566	250736
MCFD2	multiple coagulation factor deficiency 2	2	47129009	47168994	39986
MCHR2	melanin concentrating hormone receptor 2	6	100367786	100442123	74338
MCL1	MCL1, BCL2 family apoptosis regulator	1	150547032	150552066	5035
MCM2	minichromosome maintenance complex component 2	3	127317066	127341276	24211
MCM4	minichromosome maintenance complex component 4	8	48872745	48890720	17976
MCM5	minichromosome maintenance complex component 5	22	35796056	35821423	25368
MCM6	minichromosome maintenance complex component 6	2	136597196	136633996	36801
MCM7	minichromosome maintenance complex component 7	7	99690351	99699563	9213
MCM8	minichromosome maintenance 8 homologous recombination repair factor	20	5931298	5975852	44555
MCM9	minichromosome maintenance 9 homologous recombination repair factor	6	119134605	119256327	121723
MCOLN2	mucolipin 2	1	85391268	85462796	71529
MCTP1	multiple C2 and transmembrane domain containing 1	5	94039446	94620279	580834
MCTP2	multiple C2 and transmembrane domain containing 2	15	94774767	95023632	248866
MDGA2	MAM domain containing glycosylphosphatidylinositol anchor 2	14	47308826	48144157	835332
ME1	malic enzyme 1	6	83920108	84140797	220690
ME3	malic enzyme 3	11	86152150	86383678	231529
MECOM	MDS1 and EVI1 complex locus	3	168801287	169381406	580120
MECP2	methyl-CpG binding protein 2	X	153287024	153363212	76189
MED12L	mediator complex subunit 12 like	3	150803484	151154860	351377
MED13	mediator complex subunit 13	17	60019966	60142643	122678
**MED13L**	mediator complex subunit 13 like	12	116395711	116715143	319433
MED14	mediator complex subunit 14	X	40507558	40595110	87553
MED26	mediator complex subunit 26	19	16685718	16739873	54156
MED27	mediator complex subunit 27	9	134735494	134955295	219802
MED30	mediator complex subunit 30	8	118532952	118552501	19550
MED8	mediator complex subunit 8	1	43849588	43855479	5892
MEF2A	myocyte enhancer factor 2A	15	100017370	100256671	239302
MEF2C	myocyte enhancer factor 2C	5	88013975	88199922	185948
MEFV	MEFV, pyrin innate immunity regulator	16	3292028	3306627	14600
MEGF10	multiple EGF like domains 10	5	126626523	126801429	174907
MEGF11	multiple EGF like domains 11	15	66187417	66546085	358669
MEGF9	multiple EGF like domains 9	9	123363091	123476748	113658
MEI4	meiotic double-stranded break formation protein 4	6	78400375	78636691	236317
MEMO1	mediator of cell motility 1	2	32090129	32236299	146171
MERTK	MER proto-oncogene, tyrosine kinase	2	112656056	112787138	131083
MESDC2	mesoderm development candidate 2	15	81239667	81282219	42553
MEST	mesoderm specific transcript	7	130126012	130146133	20122
METAP1D	methionyl aminopeptidase type 1D, mitochondrial	2	172864490	172947158	82669
METTL15	methyltransferase like 15	11	28129795	28355054	225260
METTL16	methyltransferase like 16	17	2308856	2415185	106330
METTL21EP	methyltransferase like 21E, pseudogene	13	103532449	103548383	15935
METTL9	methyltransferase like 9	16	21608539	21668794	60256
MFAP3L	microfibrillar associated protein 3 like	4	170907748	170954182	46435
MFN2	mitofusin 2	1	12040238	12073571	33334

MFSD11	major facilitator superfamily domain containing 11	17	74731947	74777531	45585
MGA	MGA, MAX dimerization protein	15	41913422	42062141	148720
MGAT1	mannosyl (alpha-1,3-)-glycoprotein beta-1,2-N-acetylglucosaminyltransferase	5	180217541	180242652	25112
MGAT4A	mannosyl (alpha-1,3-)-glycoprotein beta-1,4-N-acetylglucosaminyltransferase, isozyme A	2	99235569	99347589	112021
MGAT4B	mannosyl (alpha-1,3-)-glycoprotein beta-1,4-N-acetylglucosaminyltransferase, isozyme B	5	179224597	179233952	9356
MGAT4C	MGAT4 family member C	12	86372516	87232681	860166
MGLL	monoglyceride lipase	3	127407909	127542051	134143
MGME1	mitochondrial genome maintenance exonuclease 1	20	17949556	17971765	22210
MGMT	O-6-methylguanine-DNA methyltransferase	10	131265448	131566271	300824
MGST2	microsomal glutathione S-transferase 2	4	140586922	140661899	74978
MIA3	MIA family member 3, ER export factor	1	222791428	222841354	49927
MIB1	mindbomb E3 ubiquitin protein ligase 1	18	19284918	19450918	166001
MICB	MHC class I polypeptide-related sequence B	6	31462658	31478901	16244
MICU1	mitochondrial calcium uptake 1	10	74127098	74385899	258802
MICU2	mitochondrial calcium uptake 2	13	22066836	22178353	111518
MICU3	mitochondrial calcium uptake family member 3	8	16884747	16980153	95407
MID1	midline 1	X	10413350	10851773	438424
MIEF1	mitochondrial elongation factor 1	22	39895437	39914137	18701
MIEN1	migration and invasion enhancer 1	17	37884749	37887040	2292
MIER3	MIER family member 3	5	56215429	56267502	52074
MIPEP	mitochondrial intermediate peptidase	13	24304328	24463558	159231
MIPOL1	mirror-image polydactyly 1	14	37667118	38021566	354449
MIR1-1HG	MIR1-1 host gene	20	61147660	61167971	20312
MIR1268A	microRNA 1268a	15	22513229	22513280	52
MIR137HG	MIR137 host gene	1	98453556	98515419	61864
MIR181A1HG	MIR181A1 host gene	1	198776622	198906558	129937
MIR3134	microRNA 3134	3	15738805	15738878	74
MIR31HG	MIR31 host gene	9	21455641	21559668	104028
MIR5009	microRNA 5009	15	90427163	90427262	100
MIR5096	microRNA 5096	4	79741906	79741975	70
MIR548AE2	microRNA 548ae-2	5	57825870	57825936	67
MIR548AI	microRNA 548ai	6	99572485	99572572	88
MIR548AJ2	microRNA 548aj-2	X	37883148	37883239	92
MIR548F1	microRNA 548f-1	10	56367634	56367717	84
MIR548F3	microRNA 548f-3	5	109849530	109849616	87
MIR548F5	microRNA 548f-5	X	32659591	32659676	86
MIR548G	microRNA 548g	4	148265781	148265869	89
MIR548H3	microRNA 548h-3	17	13446846	13446963	118
MIR548H4	microRNA 548h-4	8	26906370	26906480	111
MIR548N	microRNA 548n	7	34980372	34980446	75
MIR548W	microRNA 548w	16	26036558	26036631	74
MIR5684	microRNA 5684	19	12897942	12898006	65
MIR5694	microRNA 5694	14	67908572	67908647	76
MITF	melanogenesis associated transcription factor	3	69788586	70017488	228903
MKL1	megakaryoblastic leukemia (translocation) 1	22	40806285	41032706	226422
MKLN1	muskelin 1	7	130794855	131181395	386541
MKNK1	MAP kinase interacting serine/threonine kinase 1	1	47023090	47082515	59426
MLC1	megalencephalic leukoencephalopathy with subcortical cysts 1	22	50497820	50524331	26512
MLIP	muscular LMNA interacting protein	6	53794780	54131078	336299
MLLT3	MLLT3, super elongation complex subunit	9	20341663	20622542	280880
MLLT6	MLLT6, PHD finger domain containing	17	36861795	36886056	24262
MME	membrane metalloendopeptidase	3	154741913	154901497	159585
MMEL1	membrane metalloendopeptidase like 1	1	2522078	2564481	42404
MMP16	matrix metallopeptidase 16	8	89044237	89340254	296018
MMP2	matrix metallopeptidase 2	16	55423612	55540603	116992
MMP20	matrix metallopeptidase 20	11	102447566	102496063	48498
MMP24	matrix metallopeptidase 24	20	33814457	33864801	50345
MMP3	matrix metallopeptidase 3	11	102706532	102714534	8003
MMP9	matrix metallopeptidase 9	20	44637547	44645200	7654
MMRN1	multimerin 1	4	90800683	90875780	75098
MOB3B	MOB kinase activator 3B	9	27325207	27529779	204573
MOBP	myelin-associated oligodendrocyte basic protein	3	39508689	39570970	62282
MOCS1	molybdenum cofactor synthesis 1	6	39867354	39902290	34937
MOCS2	molybdenum cofactor synthesis 2	5	52391509	52405893	14385
MOG	myelin oligodendrocyte glycoprotein	6	29624758	29640149	15392
MON2	MON2 homolog, regulator of endosome-to-Golgi trafficking	12	62860597	62991363	130767
MORC1	MORC family CW-type zinc finger 1	3	108677086	108836989	159904
MORC2	MORC family CW-type zinc finger 2	22	31321117	31364284	43168
MORN1	MORN repeat containing 1	1	2252692	2323146	70455
MOV10L1	Mov10 RISC complex RNA helicase like 1	22	50528308	50600119	71812
MOXD1	monooxygenase DBH like 1	6	132617194	132722684	105491
MPDZ	multiple PDZ domain crumbs cell polarity complex component	9	13105703	13279589	173887
MPO	myeloperoxidase	17	56347217	56358296	11080
MPP4	membrane palmitoylated protein 4	2	202509593	202563417	53825

MPRIP	myosin phosphatase Rho interacting protein	17	16945859	17120993	175135
MPZ	myelin protein zero	1	161274525	161279762	5238
MRAP2	melanocortin 2 receptor accessory protein 2	6	84743475	84800600	57126
MRAS	muscle RAS oncogene homolog	3	138066539	138124375	57837
MRC1	mannose receptor C-type 1	10	18098352	18200091	101740
MROH1	maestro heat like repeat family member 1	8	145202919	145316843	113925
MROH5	maestro heat like repeat family member 5	8	142443929	142517330	73402
MROH7-TTC4	MROH7-TTC4 readthrough (NMD candidate)	1	55107459	55207981	100523
MRPL3	mitochondrial ribosomal protein L3	3	131181056	131221827	40772
MRPL48	mitochondrial ribosomal protein L48	11	73498361	73576178	77818
MRPS12	mitochondrial ribosomal protein S12	19	39421188	39423802	2615
MRPS25	mitochondrial ribosomal protein S25	3	15083967	15106842	22876
MRPS27	mitochondrial ribosomal protein S27	5	71515236	71616473	101238
MRPS5	mitochondrial ribosomal protein S5	2	95752952	95815179	62228
MRVI1-AS1	MRVI1 antisense RNA 1	11	10562819	10621479	58661
MSANTD3-TMEFF1	MSANTD3-TMEFF1 readthrough	9	103204553	103339918	135366
MSH3	mutS homolog 3	5	79950467	80172279	221813
MSH6	mutS homolog 6	2	47922669	48037240	114572
MSI2	musashi RNA binding protein 2	17	55333212	55762046	428835
MSR1	macrophage scavenger receptor 1	8	15965387	16424999	459613
MSRA	methionine sulfoxide reductase A	8	9911778	10286401	374624
MSRB3	methionine sulfoxide reductase B3	12	65672423	65882024	209602
MTA3	metastasis associated 1 family member 3	2	42721709	42984087	262379
MTAP	methylthioadenosine phosphorylase	9	21802542	21931646	129105
MTBP	MDM2 binding protein	8	121457640	121554373	96734
MTDH	metadherin	8	98656407	98740998	84592
MTF1	metal regulatory transcription factor 1	1	38275239	38325292	50054
MTHFD1L	methylenetetrahydrofolate dehydrogenase (NADP+ dependent) 1-like	6	151186685	151423023	236339
MTHFD2L	methylenetetrahydrofolate dehydrogenase (NADP+ dependent) 2-like	4	74979891	75168816	188926
MTHFR	methylenetetrahydrofolate reductase	1	11845780	11866977	21198
MTHFS	methenyltetrahydrofolate synthetase	15	80125927	80189721	63795
MTIF3	mitochondrial translational initiation factor 3	13	28009776	28024739	14964
MTM1	myotubularin 1	X	149737069	149841795	104727
MTMR12	myotubularin related protein 12	5	32227100	32313115	86016
MTMR2	myotubularin related protein 2	11	95566046	95658479	92434
MTMR6	myotubularin related protein 6	13	25802307	25862147	59841
**MTMR7**	myotubularin related protein 7	8	17155539	17271037	115499
MTOR	mechanistic target of rapamycin	1	11166592	11322564	155973
MTR	5-methyltetrahydrofolate-homocysteine methyltransferase	1	236958610	237067281	108672
MTURN	maturin, neural progenitor differentiation regulator homolog	7	30174426	30202378	27953
MTUS1	microtubule associated scaffold protein 1	8	17501304	17658426	157123
MTUS2	microtubule associated scaffold protein 2	13	29598748	30077892	479145
MTX2	metaxin 2	2	177134123	177202753	68631
MUC12	mucin 12, cell surface associated	7	100612904	100662230	49327
MUC19	mucin 19, oligomeric	12	40787197	40964632	177436
MUC4	mucin 4, cell surface associated	3	195473636	195539148	65513
MUSK	muscle associated receptor tyrosine kinase	9	113431051	113563859	132809
MVB12B	multivesicular body subunit 12B	9	129089128	129269320	180193
MXD1	MAX dimerization protein 1	2	70124820	70170077	45258
MYCBP2	MYC binding protein 2, E3 ubiquitin protein ligase	13	77618792	77901185	282394
MYH10	myosin heavy chain 10	17	8377523	8534079	156557
MYH13	myosin heavy chain 13	17	10201401	10276447	75047
MYH14	myosin heavy chain 14	19	50691443	50813802	122360
MYH15	myosin heavy chain 15	3	108099216	108248169	148954
MYH7B	myosin heavy chain 7B	20	33563206	33590240	27035
MYLK	myosin light chain kinase	3	123328896	123603178	274283
MYLK3	myosin light chain kinase 3	16	46740891	46824319	83429
MYO10	myosin X	5	16665395	16936372	270978
MYO16	myosin XVI	13	109248500	109860355	611856
MYO18B	myosin XVIIIB	22	26138111	26427007	288897
MYO1B	myosin IB	2	192109911	192290115	180205
MYO1D	myosin ID	17	30819540	31204195	384656
MYO3A	myosin IIIA	10	26223196	26501456	278261
MYO3B	myosin IIIB	2	171034655	171511681	477027
MYO5B	myosin VB	18	47349183	47721463	372281
MYO6	myosin VI	6	76458909	76629254	170346
MYO9A	myosin IXA	15	72114632	72410918	296287
MYOCD	myocardin	17	12569207	12672266	103060
MYOM1	myomesin 1	18	3066805	3220106	153302
MYOT	myotilin	5	137203480	137223540	20061
MYOZ3	myozenin 3	5	150040436	150058927	18492
MYRIP	myosin VIIA and Rab interacting protein	3	39850405	40301812	451408
MYT1L	myelin transcription factor 1 like	2	1792885	2335032	542148
**N4BP2L1**	NEDD4 binding protein 2 like 1	13	32974861	33002315	27455
NAA15	N(alpha)-acetyltransferase 15, NatA auxiliary subunit	4	140222609	140341187	118579
NAALADL2	N-acetylated alpha-linked acidic dipeptidase like 2	3	174156363	175523428	1367066
NADK2	NAD kinase 2, mitochondrial	5	36192694	36242381	49688

NAE1	NEDD8 activating enzyme E1 subunit 1	16	66836778	66907159	70382
NAF1	nuclear assembly factor 1 ribonucleoprotein	4	164031225	164088073	56849
**NAIP**	NLR family apoptosis inhibitory protein	5	70264310	70320941	56632
NALCN	sodium leak channel, non-selective	13	101706130	102068843	362714
NALCN-AS1	NALCN antisense RNA 1	13	101360579	101711638	351060
NAP1L4	nucleosome assembly protein 1 like 4	11	2965667	3013607	47941
NAP1L6	nucleosome assembly protein 1 like 6	X	72345876	72347919	2044
NARF	nuclear prelamin A recognition factor	17	80416056	80448413	32358
NARS2	asparaginyl-tRNA synthetase 2, mitochondrial (putative)	11	78147007	78285919	138913
NAT1	N-acetyltransferase 1	8	18027986	18081198	53213
**NAT2**	N-acetyltransferase 2	8	18248755	18258728	9974
NAV1	neuron navigator 1	1	201592411	201796102	203692
NAV2	neuron navigator 2	11	19372271	20143144	770874
NAV3	neuron navigator 3	12	78224685	78606790	382106
NBAS	neuroblastoma amplified sequence	2	15307032	15701454	394423
NBEA	neurobeachin	13	35516424	36247159	730736
NBN	nibrin	8	90945564	91015456	69893
NBPF20	NBPF member 20	1	148250249	148347506	97258
NBPF8	NBPF member 8	1	144146808	144224481	77674
NCALD	neurocalcin delta	8	102698771	103137135	438365
NCAM1	neural cell adhesion molecule 1	11	112831997	113149158	317162
NCAM2	neural cell adhesion molecule 2	21	22370633	22915650	545018
NCAPD2	non-SMC condensin I complex subunit D2	12	6602522	6641121	38600
NCAPD3	non-SMC condensin II complex subunit D3	11	134020014	134095348	75335
NCAPG2	non-SMC condensin II complex subunit G2	7	158424003	158497520	73518
NCEH1	neutral cholesterol ester hydrolase 1	3	172348039	172429008	80970
NCK2	NCK adaptor protein 2	2	106361354	106510730	149377
NCKAP1L	NCK associated protein 1 like	12	54891495	54937726	46232
NCKAP5	NCK associated protein 5	2	133429374	134326034	896661
NCOA1	nuclear receptor coactivator 1	2	24714783	24993571	278789
NCOA2	nuclear receptor coactivator 2	8	71021997	71316040	294044
NCOA3	nuclear receptor coactivator 3	20	46130601	46285621	155021
**NCOA7**	nuclear receptor coactivator 7	6	126102307	126252266	149960
NCOR2	nuclear receptor corepressor 2	12	124808961	125052135	243175
NCSTN	nicastrin	1	160313062	160328742	15681
NDC1	NDC1 transmembrane nucleoporin	1	54231133	54304533	73401
NDRG1	N-myc downstream regulated 1	8	134249414	134314265	64852
NDRG3	NDRG family member 3	20	35280169	35374481	94313
NDST3	N-deacetylase and N-sulfotransferase 3	4	118954773	119179803	225031
NDST4	N-deacetylase and N-sulfotransferase 4	4	115748919	116035032	286114
NDUFA1	NADH:ubiquinone oxidoreductase subunit A1	X	119005450	119010625	5176
NDUFA10	NADH:ubiquinone oxidoreductase subunit A10	2	240831867	240964819	132953
NDUFA5	NADH:ubiquinone oxidoreductase subunit A5	7	123177051	123198309	21259
NDUFA6	NADH:ubiquinone oxidoreductase subunit A6	22	42481529	42486959	5431
NDUFA8	NADH:ubiquinone oxidoreductase subunit A8	9	124894745	124922098	27354
NDUFAF1	NADH:ubiquinone oxidoreductase complex assembly factor 1	15	41679551	41694717	15167
NDUFB5	NADH:ubiquinone oxidoreductase subunit B5	3	179322478	179345435	22958
NDUFB7	NADH:ubiquinone oxidoreductase subunit B7	19	14676890	14682874	5985
NDUFB8	NADH:ubiquinone oxidoreductase subunit B8	10	102265385	102289638	24254
NDUFC2-KCTD14	NDUFC2-KCTD14 readthrough	11	77728017	77790911	62895
NDUFS1	NADH:ubiquinone oxidoreductase core subunit S1	2	206979541	207024327	44787
NDUFS4	NADH:ubiquinone oxidoreductase subunit S4	5	52856463	52979168	122706
NDUFS7	NADH:ubiquinone oxidoreductase core subunit S7	19	1383526	1395583	12058
NDUFS8	NADH:ubiquinone oxidoreductase core subunit S8	11	67798084	67804111	6028
NDUFV2	NADH:ubiquinone oxidoreductase core subunit V2	18	9102628	9134343	31716
NEB	nebulin	2	152341850	152591001	249152
NEBL	nebulette	10	21068902	21463116	394215
NEDD4	neural precursor cell expressed, developmentally down-regulated 4, E3 ubiquitin protein ligase	15	56119120	56285944	166825
NEDD4L	neural precursor cell expressed, developmentally down-regulated 4-like, E3 ubiquitin protein ligase	18	55711599	56068772	357174
NEDD9	neural precursor cell expressed, developmentally down-regulated 9	6	11183531	11382581	199051
**NEFH**	neurofilament heavy	22	29876219	29887379	11161
NEFL	neurofilament light	8	24808468	24814624	6157
NEGR1	neuronal growth regulator 1	1	71861623	72748417	886795
NEIL3	nei like DNA glycosylase 3	4	178230990	178284097	53108
**NEK1**	NIMA related kinase 1	4	170314426	170533780	219355
**NEK11**	NIMA related kinase 11	3	130745694	131069309	323616
NEK3	NIMA related kinase 3	13	52706775	52733996	27222
NEK5	NIMA related kinase 5	13	52611093	52703214	92122
NEK7	NIMA related kinase 7	1	198126093	198291550	165458
NELFE	negative elongation factor complex member E	6	31919864	31926887	7024
NELL1	neural EGFL like 1	11	20691117	21597227	906111
NELL2	neural EGFL like 2	12	44902058	45315631	413574
NEO1	neogenin 1	15	73344051	73597547	253497
**NETO1**	neuropilin and tolloid like 1	18	70409549	70535381	125833
NF1	neurofibromin 1	17	29421945	29709134	287190

NF2	neurofibromin 2	22	29999545	30094587	95043
NFAT5	nuclear factor of activated T-cells 5	16	69598997	69738569	139573
NFATC3	nuclear factor of activated T-cells 3	16	68118654	68263162	144509
NFE2L1	nuclear factor, erythroid 2 like 1	17	46125691	46138849	13159
NFE2L2	nuclear factor, erythroid 2 like 2	2	178092323	178257425	165103
NFIA	nuclear factor I A	1	61330931	61928465	597535
NFIB	nuclear factor I B	9	14081842	14398982	317141
NFIC	nuclear factor I C	19	3359561	3469215	109655
NFKB1	nuclear factor kappa B subunit 1	4	103422486	103538459	115974
NFS1	NFS1, cysteine desulfurase	20	34255977	34287281	31305
NGB	neuroglobin	14	77731826	77737655	5830
NGEF	neuronal guanine nucleotide exchange factor	2	233743396	233877982	134587
NGF	nerve growth factor	1	115828539	115880857	52319
NGFR	nerve growth factor receptor	17	47572655	47592379	19725
NHEJ1	non-homologous end joining factor 1	2	219940039	220025587	85549
NHLRC1	NHL repeat containing E3 ubiquitin protein ligase 1	6	18120718	18122851	2134
NHS	NHS actin remodeling regulator	X	17393543	17754114	360572
NHSL1	NHS like 1	6	138743180	139013708	270529
NHSL2	NHS like 2	X	71130938	71363424	232487
NID2	nidogen 2	14	52471521	52535712	64192
NIN	ninein	14	51186481	51297839	111359
NINJ2	ninjurin 2	12	673462	772945	99484
NINL	ninein like	20	25433341	25566153	132813
NIP7	NIP7, nucleolar pre-rRNA processing protein	16	69373333	69377014	3682
**NIPA1**	non imprinted in Prader-Willi/Angelman syndrome 1	15	23043277	23100005	56729
NIPA2	non imprinted in Prader-Willi/Angelman syndrome 2	15	23004684	23034427	29744
NIPSNAP1	nipsnap homolog 1	22	29950797	29977326	26530
NIT1	nitrilase 1	1	161087876	161095235	7360
NKAIN2	sodium/potassium transporting ATPase interacting 2	6	124125286	125146803	1021518
NKAIN3	sodium/potassium transporting ATPase interacting 3	8	63161150	63912211	751062
NKAPP1	NFKB activating protein pseudogene 1	X	119254863	119379122	124260
NKD1	naked cuticle homolog 1	16	50582241	50670647	88407
NKIRAS1	NFKB inhibitor interacting Ras like 1	3	23933151	23988082	54932
NLGN1	neuroligin 1	3	173114074	174004434	890361
NLGN4Y	neuroligin 4, Y-linked	Y	16634518	16957530	323013
NLRP11	NLR family pyrin domain containing 11	19	56296770	56348166	51397
NLRP2	NLR family pyrin domain containing 2	19	55464498	55512510	48013
**NLRP4**	NLR family pyrin domain containing 4	19	56347944	56393220	45277
NME7	NME/NM23 family member 7	1	169101769	169337205	235437
NMNAT2	nicotinamide nucleotide adenylyltransferase 2	1	183217372	183387737	170366
NMRAL1	NmrA like redox sensor 1	16	4511681	4545764	34084
NMRK1	nicotinamide riboside kinase 1	9	77675489	77703133	27645
NMT1	N-myristoyltransferase 1	17	43128978	43186384	57407
NMU	neuromedin U	4	56461396	56502865	41470
NOC2L	NOC2 like nucleolar associated transcriptional repressor	1	879584	894689	15106
NOD2	nucleotide binding oligomerization domain containing 2	16	50727514	50766988	39475
NOL10	nucleolar protein 10	2	10710892	10830101	119210
NOL4	nucleolar protein 4	18	31431064	31804916	373853
NOL9	nucleolar protein 9	1	6581407	6614595	33189
NOP14	NOP14 nucleolar protein	4	2939660	2965112	25453
NOP56	NOP56 ribonucleoprotein	20	2632791	2639039	6249
NOS1	nitric oxide synthase 1	12	117645947	117889975	244029
NOS1AP	nitric oxide synthase 1 adaptor protein	1	162039564	162353321	313758
NOS2	nitric oxide synthase 2	17	26083792	26127525	43734
NOS3	nitric oxide synthase 3	7	150688083	150711676	23594
NOTCH2	notch 2	1	120454176	120612240	158065
NOTCH3	notch 3	19	15270444	15311792	41349
NOTCH4	notch 4	6	32162620	32191844	29225
NOVA1	NOVA alternative splicing regulator 1	14	26912299	27066960	154662
NOX4	NADPH oxidase 4	11	89057524	89322779	265256
NOXRED1	NADP dependent oxidoreductase domain containing 1	14	77860364	77889860	29497
NPAS2	neuronal PAS domain protein 2	2	101436614	101613291	176678
NPAS3	neuronal PAS domain protein 3	14	33404139	34273382	869244
NPC2	NPC intracellular cholesterol transporter 2	14	74942895	74960880	17986
NPHP1	nephrocystin 1	2	110879888	110962643	82756
NPHP4	nephrocystin 4	1	5922871	6052533	129663
NPLOC4	NPL4 homolog, ubiquitin recognition factor	17	79523913	79615495	91583
NPRL3	NPR3 like, GATOR1 complex subunit	16	134273	188859	54587
NPSR1-AS1	NPSR1 antisense RNA 1	7	34386124	34911194	525071
NPY	neuropeptide Y	7	24323782	24331484	7703
NQO1	NAD(P)H quinone dehydrogenase 1	16	69740899	69760854	19956
NQO2	N-ribosyldihydronicotinamide:quinone reductase 2	6	2988221	3019996	31776
NR1H2	nuclear receptor subfamily 1 group H member 2	19	50832910	50886239	53330
NR1H4	nuclear receptor subfamily 1 group H member 4	12	100867486	100958191	90706
NR2F1-AS1	NR2F1 antisense RNA 1	5	92745065	92921354	176290
NR2F2-AS1	NR2F2 antisense RNA 1	15	96670598	96870590	199993
NR3C1	nuclear receptor subfamily 3 group C member 1	5	142657496	142815077	157582

NR3C2	nuclear receptor subfamily 3 group C member 2	4	148999913	149365850	365938
NR4A2	nuclear receptor subfamily 4 group A member 2	2	157180944	157198860	17917
NR4A3	nuclear receptor subfamily 4 group A member 3	9	102584137	102629173	45037
NR5A2	nuclear receptor subfamily 5 group A member 2	1	199996730	200146552	149823
NR6A1	nuclear receptor subfamily 6 group A member 1	9	127279554	127533589	254036
NRCAM	neuronal cell adhesion molecule	7	107788068	108097161	309094
NRG1	neuregulin 1	8	31496902	32622548	1125647
NRG2	neuregulin 2	5	139226364	139422884	196521
NRG3	neuregulin 3	10	83635070	84746935	1111866
NRP2	neuropilin 2	2	206546714	206662857	116144
NRXN1	neurexin 1	2	50145643	51259674	1114032
NRXN3	neurexin 3	14	78708734	80330762	1622029
NSD1	nuclear receptor binding SET domain protein 1	5	176560026	176727216	167191
NSF	N-ethylmaleimide sensitive factor, vesicle fusing ATPase	17	44668035	44834830	166796
NSL1	NSL1, MIS12 kinetochore complex component	1	212899495	212965124	65630
NSMCE2	NSE2/MMS21 homolog, SMC5-SMC6 complex SUMO ligase	8	126103921	126379362	275442
NSUN4	NOP2/Sun RNA methyltransferase family member 4	1	46805849	46830824	24976
**NT5C1A**	5'-nucleotidase, cytosolic IA	1	40124793	40137710	12918
NT5C2	5'-nucleotidase, cytosolic II	10	104845940	104953056	107117
NT5C3B	5'-nucleotidase, cytosolic IIIB	17	39981335	39992523	11189
NT5DC1	5'-nucleotidase domain containing 1	6	116422012	116570660	148649
NT5E	5'-nucleotidase ecto	6	86159809	86205500	45692
NT5M	5',3'-nucleotidase, mitochondrial	17	17206649	17250977	44329
NTF3	neurotrophin 3	12	5541278	5630702	89425
NTM	neurotrimin	11	131240373	132206716	966344
NTN1	netrin 1	17	8924859	9147317	222459
NTN4	netrin 4	12	96051583	96184930	133348
**NTNG1**	netrin G1	1	107682629	108026080	343452
NTNG2	netrin G2	9	135037334	135119921	82588
NTRK1	neurotrophic receptor tyrosine kinase 1	1	156785432	156851642	66211
NTRK2	neurotrophic receptor tyrosine kinase 2	9	87283466	87638505	355040
NTRK3	neurotrophic receptor tyrosine kinase 3	15	88418230	88799999	381770
NTS	neurotensin	12	86268073	86276770	8698
NUAK1	NUAK family kinase 1	12	106457118	106533811	76694
NUBPL	nucleotide binding protein like	14	31959162	32330430	371269
NUCKS1	nuclear casein kinase and cyclin dependent kinase substrate 1	1	205681947	205719404	37458
NUDCD3	NudC domain containing 3	7	44418720	44530479	111760
NUDT1	nudix hydrolase 1	7	2281857	2290781	8925
NUFIP1	NUFIP1, FMR1 interacting protein 1	13	45513384	45563618	50235
NUMB	NUMB, endocytic adaptor protein	14	73741815	73930348	188534
NUP153	nucleoporin 153	6	17615269	17706656	91388
NUP188	nucleoporin 188	9	131709978	131769375	59398
NUP210P1	nucleoporin 210 pseudogene 1	3	126379452	126390782	11331
NUP37	nucleoporin 37	12	102467967	102513902	45936
NUP62CL	nucleoporin 62 C-terminal like	X	106366657	106449670	83014
NUP93	nucleoporin 93	16	56764017	56878797	114781
NUSAP1	nucleolar and spindle associated protein 1	15	41624892	41673248	48357
NUTF2	nuclear transport factor 2	16	67880635	67906470	25836
NXN	nucleoredoxin	17	702553	883010	180458
NXPE1	neurexophilin and PC-esterase domain family member 1	11	114392437	114430617	38181
NXPH1	neurexophilin 1	7	8473585	8792593	319009
NXPH2	neurexophilin 2	2	139428342	139537918	109577
NYAP2	neuronal tyrosine-phosphorylated phosphoinositide-3-kinase adaptor 2	2	226265364	226518734	253371
OARD1	O-acyl-ADP-ribose deacylase 1	6	41001366	41065526	64161
OAZ3	ornithine decarboxylase antizyme 3	1	151735445	151743808	8364
OBSCN	obscurin, cytoskeletal calmodulin and titin-interacting RhoGEF	1	228395831	228566577	170747
OCA2	OCA2 melanosomal transmembrane protein	15	28000021	28344504	344484
ODC1	ornithine decarboxylase 1	2	10580094	10588630	8537
**OGG1**	8-oxoguanine DNA glycosylase	3	9791628	9829903	38276
OLA1	Obg like ATPase 1	2	174937175	175113426	176252
OLAH	oleoyl-ACP hydrolase	10	15074226	15115851	41626
OLFM3	olfactomedin 3	1	102268130	102462586	194457
OLFM4	olfactomedin 4	13	53602894	53626192	23299
OLFML1	olfactomedin like 1	11	7506619	7532608	25990
OLIG2	oligodendrocyte transcription factor 2	21	34398153	34401504	3352
OLR1	oxidized low density lipoprotein receptor 1	12	10310902	10324737	13836
**OMA1**	OMA1 zinc metallopeptidase	1	58881056	59012474	131419
OPCML	opioid binding protein/cell adhesion molecule like	11	132284871	133402414	1117544
**OPTN**	optineurin	10	13141449	13180291	38843
OR2L13	olfactory receptor family 2 subfamily L member 13	1	248100493	248264224	163732
**OR6P1**	olfactory receptor family 6 subfamily P member 1	1	158532441	158533394	954
OR9Q1	olfactory receptor family 9 subfamily Q member 1	11	57791353	57949088	157736
ORAOV1	oral cancer overexpressed 1	11	69467844	69490184	22341
ORC4	origin recognition complex subunit 4	2	148687968	148779147	91180
ORC6	origin recognition complex subunit 6	16	46723555	46732306	8752
OSBP2	oxysterol binding protein 2	22	31089769	31303811	214043

OSBPL10	oxysterol binding protein like 10	3	31699382	32119072	419691
OSBPL11	oxysterol binding protein like 11	3	125247702	125313934	66233
OSBPL1A	oxysterol binding protein like 1A	18	21742008	21977844	235837
**OSBPL3**	oxysterol binding protein like 3	7	24836158	25021253	185096
OSBPL6	oxysterol binding protein like 6	2	179059208	179264160	204953
OTC	ornithine carbamoyltransferase	X	38211798	38280703	68906
OTUD7B	OTU deubiquitinase 7B	1	149909705	149982625	72921
OTX2	orthodenticle homeobox 2	14	57267425	57277197	9773
OVCH1-AS1	OVCH1 antisense RNA 1	12	29542227	29640421	98195
OXCT1	3-oxoacid CoA-transferase 1	5	41730167	41870621	140455
OXR1	oxidation resistance 1	8	107282473	107764922	482450
OXSR1	oxidative stress responsive 1	3	38206580	38296979	90400
**P2RX7**	purinergic receptor P2X 7	12	121570622	121623876	53255
P4HA1	prolyl 4-hydroxylase subunit alpha 1	10	74766975	74856732	89758
PABPC4	poly(A) binding protein cytoplasmic 4	1	40026488	40042462	15975
PACRG	parkin coregulated	6	163148164	163736524	588361
PACS1	phosphofurin acidic cluster sorting protein 1	11	65837834	66012218	174385
PACSIN2	protein kinase C and casein kinase substrate in neurons 2	22	43231418	43411151	179734
PAFAH1B1	platelet activating factor acetylhydrolase 1b regulatory subunit 1	17	2496504	2588909	92406
PAFAH1B3	platelet activating factor acetylhydrolase 1b catalytic subunit 3	19	42801185	42807698	6514
PAG1	phosphoprotein membrane anchor with glycosphingolipid microdomains 1	8	81880045	82024303	144259
PAK1	p21 (RAC1) activated kinase 1	11	77032752	77185680	152929
PAK3	p21 (RAC1) activated kinase 3	X	110187513	110470589	283077
PAK4	p21 (RAC1) activated kinase 4	19	39616410	39673456	57047
PALLD	palladin, cytoskeletal associated protein	4	169418217	169849608	431392
**PALM2**	paralemmin 2	9	112403068	112713755	310688
PAN3	PAN3 poly(A) specific ribonuclease subunit	13	28712643	28869475	156833
PANK2	pantothenate kinase 2	20	3869486	3907605	38120
PANX1	pannexin 1	11	93862094	93915138	53045
PAPD5	poly(A) RNA polymerase D5, non-canonical	16	50186829	50269221	82393
**PAPPA**	pappalysin 1	9	118916083	119164601	248519
PAPPA2	pappalysin 2	1	176432307	176814735	382429
PAPSS2	3'-phosphoadenosine 5'-phosphosulfate synthase 2	10	89419370	89507462	88093
PAQR5	progestin and adipoQ receptor family member 5	15	69591286	69700119	108834
PARD3	par-3 family cell polarity regulator	10	34398488	35104253	705766
PARD3B	par-3 family cell polarity regulator beta	2	205410516	206484886	1074371
PARG	poly(ADP-ribose) glycohydrolase	10	51026325	51130715	104391
**PARK7**	Parkinsonism associated deglycase	1	8014351	8045565	31215
PARN	poly(A)-specific ribonuclease	16	14529558	14726585	197028
PARP1	poly(ADP-ribose) polymerase 1	1	226548392	226595780	47389
PARP12	poly(ADP-ribose) polymerase family member 12	7	139723544	139763521	39978
PARP4	poly(ADP-ribose) polymerase family member 4	13	24995064	25086948	91885
PARVB	parvin beta	22	44395091	44568829	173739
PASD1	PAS domain containing repressor 1	X	150732094	150845211	113118
PATL2	PAT1 homolog 2	15	44957930	45003514	45585
PAWR	pro-apoptotic WT1 regulator	12	79968759	80084877	116119
PAX2	paired box 2	10	102495360	102589698	94339
PAX3	paired box 3	2	223064607	223163715	99109
PAX6	paired box 6	11	31806340	31839509	33170
PBK	PDZ binding kinase	8	27667137	27695612	28476
PBRM1	polybromo 1	3	52579368	52719933	140566
PBX1	PBX homeobox 1	1	164524821	164868533	343713
PBX3	PBX homeobox 3	9	128509624	128729656	220033
PCAT4	prostate cancer associated transcript 4 (non-protein coding)	4	80748625	80798924	50300
PCCA	propionyl-CoA carboxylase alpha subunit	13	100741269	101182686	441418
PCDH11X	protocadherin 11 X-linked	X	91034260	91878229	843970
PCDH11Y	protocadherin 11 Y-linked	Y	4868267	5610265	741999
PCDH15	protocadherin related 15	10	55562531	57387702	1825172
PCDH19	protocadherin 19	X	99546642	99665271	118630
PCDH7	protocadherin 7	4	30722037	31148422	426386
PCDH9	protocadherin 9	13	66876967	67804468	927502
PCED1B	PC-esterase domain containing 1B	12	47473386	47630443	157058
PCGF3	polycomb group ring finger 3	4	699537	764428	64892
PCID2	PCI domain containing 2	13	113831891	113863029	31139
PCK1	phosphoenolpyruvate carboxykinase 1	20	56136136	56141513	5378
PCLO	piccolo presynaptic cytomatrix protein	7	82383329	82792246	408918
PCM1	pericentriolar material 1	8	17780349	17885478	105130
PCMTD1	protein-L-isoaspartate (D-aspartate) O-methyltransferase domain containing 1	8	52730140	52811735	81596
PCNA	proliferating cell nuclear antigen	20	5095599	5107272	11674
**PCP4**	Purkinje cell protein 4	21	41239243	41301322	62080
PCSK5	proprotein convertase subtilisin/kexin type 5	9	78505560	78977255	471696
PCSK6	proprotein convertase subtilisin/kexin type 6	15	101840818	102065405	224588
PCSK9	proprotein convertase subtilisin/kexin type 9	1	55505221	55530525	25305
PDCD10	programmed cell death 10	3	167401086	167452727	51642
PDCD4	programmed cell death 4	10	112631565	112659764	28200

PDE10A	phosphodiesterase 10A	6	165740776	166400091	659316
PDE11A	phosphodiesterase 11A	2	178487980	178973066	485087
PDE1A	phosphodiesterase 1A	2	183004763	183387919	383157
PDE1C	phosphodiesterase 1C	7	31790793	32338941	548149
PDE3A	phosphodiesterase 3A	12	20522179	20837315	315137
PDE4B	phosphodiesterase 4B	1	66258197	66840259	582063
PDE4D	phosphodiesterase 4D	5	58264865	59817947	1553083
**PDE5A**	phosphodiesterase 5A	4	120415550	120550146	134597
PDE7A	phosphodiesterase 7A	8	66629745	66754557	124813
PDE7B	phosphodiesterase 7B	6	136172834	136516712	343879
PDE8A	phosphodiesterase 8A	15	85523671	85682376	158706
PDF	peptide deformylase (mitochondrial)	16	69362524	69364498	1975
PDGFB	platelet derived growth factor subunit B	22	39619364	39640756	21393
PDGFC	platelet derived growth factor C	4	157681606	157892546	210941
PDGFD	platelet derived growth factor D	11	103777914	104035107	257194
PDGFRB	platelet derived growth factor receptor beta	5	149493400	149535435	42036
PDGFRL	platelet derived growth factor receptor like	8	17433942	17501580	67639
PDLIM3	PDZ and LIM domain 3	4	186422851	186456766	33916
PDPR	pyruvate dehydrogenase phosphatase regulatory subunit	16	70147529	70195203	47675
PDS5A	PDS5 cohesin associated factor A	4	39824483	39979576	155094
PDS5B	PDS5 cohesin associated factor B	13	33160564	33352157	191594
PDSS2	decaprenyl diphosphate synthase subunit 2	6	107473761	107780768	307008
PDXDC1	pyridoxal dependent decarboxylase domain containing 1	16	15068448	15233196	164749
PDXK	pyridoxal kinase	21	45138975	45182188	43214
PDYN	prodynorphin	20	1959403	1974732	15330
PDZD2	PDZ domain containing 2	5	31639517	32111037	471521
PDZRN3	PDZ domain containing ring finger 3	3	73431584	73674091	242508
PDZRN4	PDZ domain containing ring finger 4	12	41582250	41968392	386143
PEAK1	pseudopodium enriched atypical kinase 1	15	77400471	77712486	312016
PEBP4	phosphatidylethanolamine binding protein 4	8	22570769	22857513	286745
**PELP1**	proline, glutamate and leucine rich protein 1	17	4574679	4607632	32954
PEX10	peroxisomal biogenesis factor 10	1	2336236	2345236	9001
PEX12	peroxisomal biogenesis factor 12	17	33901814	33905882	4069
PEX14	peroxisomal biogenesis factor 14	1	10532345	10690815	158471
PEX19	peroxisomal biogenesis factor 19	1	160246602	160256138	9537
PEX5L	peroxisomal biogenesis factor 5 like	3	179512746	179754841	242096
PFAS	phosphoribosylformylglycinamidine synthase	17	8150936	8173809	22874
**PFN1**	profilin 1	17	4848947	4852356	3410
PGAP1	post-GPI attachment to proteins 1	2	197697728	197792520	94793
PGBD1	piggyBac transposable element derived 1	6	28249314	28270326	21013
PGGT1B	protein geranylgeranyltransferase type I subunit beta	5	114546527	114598569	52043
PGM1	phosphoglucomutase 1	1	64058947	64125916	66970
PGM2L1	phosphoglucomutase 2 like 1	11	74041363	74109518	68156
PGM5	phosphoglucomutase 5	9	70943224	71145977	202754
PGR	progesterone receptor	11	100900355	101001255	100901
PHACTR1	phosphatase and actin regulator 1	6	12717893	13288645	570753
PHACTR2	phosphatase and actin regulator 2	6	143857982	144152322	294341
PHACTR3	phosphatase and actin regulator 3	20	58152564	58422766	270203
PHC3	polyhomeotic homolog 3	3	169804520	169899537	95018
PHEX	phosphate regulating endopeptidase homolog, X-linked	X	22050559	22269427	218869
PHF20	PHD finger protein 20	20	34359896	34538303	178408
PHF8	PHD finger protein 8	X	53963109	54075391	112283
PHIP	pleckstrin homology domain interacting protein	6	79645584	79787953	142370
PHKB	phosphorylase kinase regulatory subunit beta	16	47495034	47735434	240401
PHLPP1	PH domain and leucine rich repeat protein phosphatase 1	18	60382672	60647666	264995
PHLPP2	PH domain and leucine rich repeat protein phosphatase 2	16	71671738	71758604	86867
PHOX2B	paired like homeobox 2b	4	41746099	41750987	4889
PHTF1	putative homeodomain transcription factor 1	1	114239453	114302111	62659
PHYH	phytanoyl-CoA 2-hydroxylase	10	13319796	13344412	24617
PI4KA	phosphatidylinositol 4-kinase alpha	22	21061979	21213705	151727
PIAS2	protein inhibitor of activated STAT 2	18	44388353	44500123	111771
PIBF1	progesterone immunomodulatory binding factor 1	13	73356197	73590591	234395
PICALM	phosphatidylinositol binding clathrin assembly protein	11	85668727	85780924	112198
PID1	phosphotyrosine interaction domain containing 1	2	229715242	230136001	420760
PIEZO2	piezo type mechanosensitive ion channel component 2	18	10666480	11148587	482108
PIGA	phosphatidylinositol glycan anchor biosynthesis class A	X	15337573	15353676	16104
PIGK	phosphatidylinositol glycan anchor biosynthesis class K	1	77554675	77685115	130441
PIGL	phosphatidylinositol glycan anchor biosynthesis class L	17	16120505	16252115	131611
PIGN	phosphatidylinositol glycan anchor biosynthesis class N	18	59710800	59854351	143552
PIGS	phosphatidylinositol glycan anchor biosynthesis class S	17	26880401	26898890	18490
PIK3C2A	phosphatidylinositol-4-phosphate 3-kinase catalytic subunit type 2 alpha	11	17099277	17229530	130254
PIK3C2B	phosphatidylinositol-4-phosphate 3-kinase catalytic subunit type 2 beta	1	204391756	204463852	72097
PIK3C2G	phosphatidylinositol-4-phosphate 3-kinase catalytic subunit type 2 gamma	12	18400548	18801348	400801
PIK3C3	phosphatidylinositol 3-kinase catalytic subunit type 3	18	39535171	39667794	132624

PIK3CA	phosphatidylinositol-4,5-bisphosphate 3-kinase catalytic subunit alpha	3	178865902	178957881	91980
PIK3CG	phosphatidylinositol-4,5-bisphosphate 3-kinase catalytic subunit gamma	7	106505723	106547590	41868
PIK3R1	phosphoinositide-3-kinase regulatory subunit 1	5	67511548	67597649	86102
PIK3R5	phosphoinositide-3-kinase regulatory subunit 5	17	8782233	8869029	86797
PIN1	peptidylprolyl cis/trans isomerase, NIMA-interacting 1	19	9945933	9960358	14426
PINK1	PTEN induced putative kinase 1	1	20959948	20978004	18057
PINK1-AS	PINK1 antisense RNA	1	20969150	20978686	9537
PINX1	PIN2/TERF1 interacting, telomerase inhibitor 1	8	10622473	10697394	74922
PIP4K2A	phosphatidylinositol-5-phosphate 4-kinase type 2 alpha	10	22823778	23003484	179707
PIP5K1B	phosphatidylinositol-4-phosphate 5-kinase type 1 beta	9	71320575	71624092	303518
PITPNA	phosphatidylinositol transfer protein alpha	17	1421012	1466110	45099
PITPNC1	phosphatidylinositol transfer protein, cytoplasmic 1	17	65373575	65693372	319798
PITX1	paired like homeodomain 1	5	134363425	134370503	7079
PITX3	paired like homeodomain 3	10	103989943	104001231	11289
**PIWIL2**	piwi like RNA-mediated gene silencing 2	8	22132810	22215076	82267
PIWIL3	piwi like RNA-mediated gene silencing 3	22	25115001	25170687	55687
PJA2	praja ring finger ubiquitin ligase 2	5	108670410	108745695	75286
PKD1	polycystin 1, transient receptor potential channel interacting	16	2138711	2185899	47189
PKD2	polycystin 2, transient receptor potential cation channel	4	88928820	88998929	70110
PKHD1	PKHD1, fibrocystin/polyductin	6	51480098	51952423	472326
PKIG	cAMP-dependent protein kinase inhibitor gamma	20	43160426	43252888	92463
PKN2	protein kinase N2	1	89149905	89301938	152034
PLA1A	phospholipase A1 member A	3	119316689	119348658	31970
PLA2G16	phospholipase A2 group XVI	11	63340667	63384355	43689
PLA2G1B	phospholipase A2 group IB	12	120759914	120765592	5679
PLA2G4A	phospholipase A2 group IVA	1	186798085	186958113	160029
PLA2G4E	phospholipase A2 group IVE	15	42273780	42343388	69609
PLA2G6	phospholipase A2 group VI	22	38507502	38601697	94196
PLAA	phospholipase A2 activating protein	9	26904081	26947461	43381
PLAC1	placenta specific 1	X	133699868	133898352	198485
PLAC8	placenta specific 8	4	84011201	84058228	47028
PLAG1	PLAG1 zinc finger	8	57073463	57123883	50421
PLAGL1	PLAG1 like zinc finger 1	6	144261437	144385735	124299
PLAT	plasminogen activator, tissue type	8	42032236	42065242	33007
PLB1	phospholipase B1	2	28680012	28866654	186643
PLCB1	phospholipase C beta 1	20	8112824	8949003	836180
PLCB4	phospholipase C beta 4	20	9049410	9461889	412480
PLCE1	phospholipase C epsilon 1	10	95753746	96092580	338835
PLCG2	phospholipase C gamma 2	16	81772702	81991899	219198
PLCH1	phospholipase C eta 1	3	155093369	155462856	369488
PLCL1	phospholipase C like 1	2	198669426	199437305	767880
PLCL2	phospholipase C like 2	3	16844159	17132086	287928
PLD1	phospholipase D1	3	171318195	171528740	210546
PLD5	phospholipase D family member 5	1	242246288	242687998	441711
PLEC	plectin	8	144989321	145050902	61582
PLEKHA1	pleckstrin homology domain containing A1	10	124134212	124191867	57656
PLEKHA5	pleckstrin homology domain containing A5	12	19282648	19529334	246687
PLEKHA7	pleckstrin homology domain containing A7	11	16799842	17035990	236149
PLEKHG1	pleckstrin homology and RhoGEF domain containing G1	6	150920999	151164799	243801
**PLEKHG5**	pleckstrin homology and RhoGEF domain containing G5	1	6526152	6580121	53970
PLEKHM1	pleckstrin homology and RUN domain containing M1	17	43513266	43568115	54850
PLEKHM3	pleckstrin homology domain containing M3	2	208693027	208890284	197258
PLG	plasminogen	6	161123270	161174347	51078
PLOD2	procollagen-lysine,2-oxoglutarate 5-dioxygenase 2	3	145787227	145881440	94214
PLP1	proteolipid protein 1	X	103028647	103047548	18902
PLS1	plastin 1	3	142315229	142432506	117278
PLXDC2	plexin domain containing 2	10	20105168	20578785	473618
PLXNA2	plexin A2	1	208195587	208417665	222079
PLXNA4	plexin A4	7	131808091	132333447	525357
PLXNC1	plexin C1	12	94542499	94701451	158953
PM20D1	peptidase M20 domain containing 1	1	205797150	205819260	22111
PMP22	peripheral myelin protein 22	17	15133095	15168643	35549
PNKP	polynucleotide kinase 3'-phosphatase	19	50364461	50371166	6706
PNMA2	paraneoplastic Ma antigen 2	8	26362202	26371608	9407
PNMT	phenylethanolamine N-methyltransferase	17	37824234	37826728	2495
PNP	purine nucleoside phosphorylase	14	20937113	20945253	8141
PNPO	pyridoxamine 5'-phosphate oxidase	17	46018872	46025654	6783
POC1B	POC1 centriolar protein B	12	89813495	89919801	106307
POC5	POC5 centriolar protein	5	74969949	75013313	43365
PODXL	podocalyxin like	7	131185021	131242976	57956
POF1B	premature ovarian failure, 1B	X	84532402	84634748	102347
POGZ	pogo transposable element derived with ZNF domain	1	151375200	151431941	56742
POLA1	DNA polymerase alpha 1, catalytic subunit	X	24712036	25015103	303068
POLA2	DNA polymerase alpha 2, accessory subunit	11	65029233	65073060	43828
POLDIP3	DNA polymerase delta interacting protein 3	22	42979727	43010968	31242
POLE2	DNA polymerase epsilon 2, accessory subunit	14	50110273	50155140	44868

POLG	DNA polymerase gamma, catalytic subunit	15	89859534	89878092	18559
POLI	DNA polymerase iota	18	51795774	51847636	51863
POLN	DNA polymerase nu	4	2073645	2243848	170204
POLQ	DNA polymerase theta	3	121150278	121264853	114576
POLR1A	RNA polymerase I subunit A	2	86247339	86333278	85940
POLR1B	RNA polymerase I subunit B	2	113299492	113334635	35144
POLR2C	RNA polymerase II subunit C	16	57496299	57505922	9624
POLR2F	RNA polymerase II subunit F	22	38348614	38437922	89309
POLR3A	RNA polymerase III subunit A	10	79734907	79789303	54397
POLR3B	RNA polymerase III subunit B	12	106751436	106903976	152541
POLR3E	RNA polymerase III subunit E	16	22308730	22346424	37695
POMC	proopiomelanocortin	2	25383722	25391772	8051
POMGNT1	protein O-linked mannose N-acetylglucosaminyltransferase 1 (beta 1,2-)	1	46654354	46685977	31624
POMT1	protein O-mannosyltransferase 1	9	134378289	134399193	20905
POMT2	protein O-mannosyltransferase 2	14	77741299	77787227	45929
**PON1**	paraoxonase 1	7	94926988	95025673	98686
**PON2**	paraoxonase 2	7	95034175	95064510	30336
**PON3**	paraoxonase 3	7	94989256	95025680	36425
PORCN	porcupine homolog (Drosophila)	X	48367350	48379202	11853
POT1	protection of telomeres 1	7	124462440	124570037	107598
POU2F1	POU class 2 homeobox 1	1	167190066	167396582	206517
POU6F2	POU class 6 homeobox 2	7	39017598	39532694	515097
**PPA2**	pyrophosphatase (inorganic) 2	4	106290234	106395238	105005
PPARA	peroxisome proliferator activated receptor alpha	22	46546424	46639653	93230
PPARD	peroxisome proliferator activated receptor delta	6	35310335	35395968	85634
PPARG	peroxisome proliferator activated receptor gamma	3	12328867	12475855	146989
PPARGC1A	PPARG coactivator 1 alpha	4	23756664	23905712	149049
PPARGC1B	PPARG coactivator 1 beta	5	149109861	149234585	124725
PPAT	phosphoribosyl pyrophosphate amidotransferase	4	57259528	57301781	42254
PPFIA1	PTPRF interacting protein alpha 1	11	70116806	70230509	113704
PPFIA2	PTPRF interacting protein alpha 2	12	81652045	82153332	501288
PPHLN1	periphilin 1	12	42632249	42853517	221269
PPIG	peptidylprolyl isomerase G	2	170440850	170497916	57067
PPIL2	peptidylprolyl isomerase like 2	22	22006559	22054304	47746
PPM1D	protein phosphatase, Mg2+/Mn2+ dependent 1D	17	58677544	58741849	64306
PPM1H	protein phosphatase, Mg2+/Mn2+ dependent 1H	12	63037762	63328817	291056
PPM1K	protein phosphatase, Mg2+/Mn2+ dependent 1K	4	89178772	89205921	27150
PPM1L	protein phosphatase, Mg2+/Mn2+ dependent 1L	3	160473390	160796695	323306
PPP1CC	protein phosphatase 1 catalytic subunit gamma	12	111157485	111180744	23260
PPP1R10	protein phosphatase 1 regulatory subunit 10	6	30568177	30586389	18213
**PPP1R13B**	protein phosphatase 1 regulatory subunit 13B	14	104200089	104313927	113839
PPP1R14D	protein phosphatase 1 regulatory inhibitor subunit 14D	15	41107650	41120907	13258
PPP1R17	protein phosphatase 1 regulatory subunit 17	7	31726329	31748069	21741
PPP1R1C	protein phosphatase 1 regulatory inhibitor subunit 1C	2	182818968	182996125	177158
PPP1R3A	protein phosphatase 1 regulatory subunit 3A	7	113516832	113715975	199144
PPP1R3F	protein phosphatase 1 regulatory subunit 3F	X	49126306	49157929	31624
PPP1R9A	protein phosphatase 1 regulatory subunit 9A	7	94536514	94925727	389214
PPP2R1A	protein phosphatase 2 scaffold subunit Aalpha	19	52693292	52730687	37396
PPP2R2B	protein phosphatase 2 regulatory subunit Bbeta	5	145967936	146464347	496412
PPP2R2C	protein phosphatase 2 regulatory subunit Bgamma	4	6322305	6565327	243023
PPP2R5C	protein phosphatase 2 regulatory subunit B'gamma	14	102228135	102394326	166192
PPP2R5D	protein phosphatase 2 regulatory subunit B'delta	6	42952237	42980080	27844
PPP2R5E	protein phosphatase 2 regulatory subunit B'epsilon	14	63838075	64010092	172018
PPP3CA	protein phosphatase 3 catalytic subunit alpha	4	101944566	102269435	324870
PPP3CC	protein phosphatase 3 catalytic subunit gamma	8	22298332	22398652	100321
PPP3R1	protein phosphatase 3 regulatory subunit B, alpha	2	68405989	68483369	77381
PPP6R1	protein phosphatase 6 regulatory subunit 1	19	55741148	55770363	29216
PPP6R2	protein phosphatase 6 regulatory subunit 2	22	50781733	50883514	101782
PPP6R3	protein phosphatase 6 regulatory subunit 3	11	68228186	68382802	154617
PQLC1	PQ loop repeat containing 1	18	77662420	77711664	49245
PRC1	protein regulator of cytokinesis 1	15	91509270	91538859	29590
PRCP	prolylcarboxypeptidase	11	82534544	82681626	147083
PRDM10	PR/SET domain 10	11	129769601	129872730	103130
PRDM15	PR/SET domain 15	21	43218385	43299591	81207
PRDM16	PR/SET domain 16	1	2985732	3355185	369454
PRDM2	PR/SET domain 2	1	14026693	14151574	124882
PRDM4	PR/SET domain 4	12	108126643	108155049	28407
**PRDM5**	PR/SET domain 5	4	121606074	121844025	237952
PRDX6	peroxiredoxin 6	1	173446405	173457946	11542
PREPL	prolyl endopeptidase-like	2	44543420	44589001	45582
PREX1	phosphatidylinositol-3,4,5-trisphosphate dependent Rac exchange factor 1	20	47240790	47444420	203631
PREX2	phosphatidylinositol-3,4,5-trisphosphate dependent Rac exchange factor 2	8	68864353	69149265	284913
PRICKLE1	prickle planar cell polarity protein 1	12	42852140	42984157	132018
PRIM2	primase (DNA) subunit 2	6	57179603	57513375	333773

PRIMPOL	primase and DNA directed polymerase	4	185570767	185616117	45351
PRKAA1	protein kinase AMP-activated catalytic subunit alpha 1	5	40759481	40798476	38996
PRKAA2	protein kinase AMP-activated catalytic subunit alpha 2	1	57110995	57181008	70014
PRKAG2	protein kinase AMP-activated non-catalytic subunit gamma 2	7	151253197	151574210	321014
PRKAR1B	protein kinase cAMP-dependent type I regulatory subunit beta	7	588834	767287	178454
PRKAR2A	protein kinase cAMP-dependent type II regulatory subunit alpha	3	48782030	48885279	103250
PRKAR2B	protein kinase cAMP-dependent type II regulatory subunit beta	7	106685094	106802256	117163
PRKCA	protein kinase C alpha	17	64298754	64806861	508108
PRKCB	protein kinase C beta	16	23847322	24231932	384611
PRKCE	protein kinase C epsilon	2	45878484	46415129	536646
PRKCG	protein kinase C gamma	19	54382444	54410906	28463
PRKCH	protein kinase C eta	14	61654277	62017694	363418
PRKCQ	protein kinase C theta	10	6469105	6622263	153159
PRKCSH	protein kinase C substrate 80K-H	19	11546109	11561783	15675
PRKCZ	protein kinase C zeta	1	1981909	2116834	134926
**PRKD1**	protein kinase D1	14	30045687	30661104	615418
PRKD3	protein kinase D3	2	37477645	37551951	74307
PRKDC	protein kinase, DNA-activated, catalytic polypeptide	8	48685669	48872743	187075
PRKG1	protein kinase, cGMP-dependent, type I	10	52750945	54058110	1307166
PRKX	protein kinase, X-linked	X	3522411	3631649	109239
PRKY	protein kinase, Y-linked, pseudogene	Y	7142013	7249589	107577
PRLR	prolactin receptor	5	35048861	35230794	181934
PRMT1	protein arginine methyltransferase 1	19	50179043	50192286	13244
PRMT2	protein arginine methyltransferase 2	21	48055079	48085036	29958
PRND	prion like protein doppel	20	4702556	4709106	6551
PRNP	prion protein	20	4666882	4682236	15355
PROCA1	protein interacting with cyclin A1	17	27030215	27038872	8658
PRODH	proline dehydrogenase 1	22	18900294	18924066	23773
PROSER2	proline and serine rich 2	10	11865338	11914276	48939
PROX1	prospero homeobox 1	1	214156524	214214595	58072
PRPF3	pre-mRNA processing factor 3	1	150293925	150325671	31747
PRPF4	pre-mRNA processing factor 4	9	116037623	116055185	17563
PRPF40A	pre-mRNA processing factor 40 homolog A	2	153508107	153574511	66405
PRPF4B	pre-mRNA processing factor 4B	6	4021501	4065217	43717
PRPF6	pre-mRNA processing factor 6	20	62612488	62664453	51966
PRPS1	phosphoribosyl pyrophosphate synthetase 1	X	106871737	106894256	22520
PRPSAP2	phosphoribosyl pyrophosphate synthetase associated protein 2	17	18743398	18834581	91184
PRR11	proline rich 11	17	57232860	57282066	49207
PRR16	proline rich 16	5	119799973	120023027	223055
PRR5L	proline rich 5 like	11	36317838	36486754	168917
PRRG1	proline rich and Gla domain 1	X	37208528	37316548	108021
PRRG4	proline rich and Gla domain 4	11	32851489	32879669	28181
PRRT2	proline rich transmembrane protein 2	16	29823177	29827201	4025
PRSS23	protease, serine 23	11	86502101	86663952	161852
PRSS53	protease, serine 53	16	31094746	31100949	6204
PRSS55	protease, serine 55	8	10383056	10411676	28621
**PRUNE2**	prune homolog 2	9	79226292	79521003	294712
PRX	periaxin	19	40899675	40919273	19599
PRY	PTPN13-like, Y-linked	Y	24636544	24660784	24241
PSAP	prosaposin	10	73576055	73611126	35072
PSD3	pleckstrin and Sec7 domain containing 3	8	18384811	18942240	557430
**PSEN1**	presenilin 1	14	73603126	73690399	87274
PSEN2	presenilin 2	1	227057885	227083806	25922
PSENEN	presenilin enhancer gamma-secretase subunit	19	36236015	36237911	1897
PSMA1	proteasome subunit alpha 1	11	14515329	14665181	149853
PSMA4	proteasome subunit alpha 4	15	78832747	78841604	8858
PSMA5	proteasome subunit alpha 5	1	109941653	109969062	27410
PSMB1	proteasome subunit beta 1	6	170844205	170862429	18225
PSMB9	proteasome subunit beta 9	6	32811913	32827362	15450
PSMC4	proteasome 26S subunit, ATPase 4	19	40476912	40487348	10437
PSMD1	proteasome 26S subunit, non-ATPase 1	2	231921578	232037541	115964
PSMD14	proteasome 26S subunit, non-ATPase 14	2	162164549	162268228	103680
PSMD6	proteasome 26S subunit, non-ATPase 6	3	63996225	64009658	13434
PSMG1	proteasome assembly chaperone 1	21	40546695	40555777	9083
PSMG3-AS1	PSMG3 antisense RNA 1 (head to head)	7	1609709	1629262	19554
PSPC1	paraspeckle component 1	13	20248896	20357142	108247
PSPH	phosphoserine phosphatase	7	56078744	56119297	40554
PSTK	phosphoseryl-tRNA kinase	10	124713897	124757029	43133
PSTPIP2	proline-serine-threonine phosphatase interacting protein 2	18	43563502	43652238	88737
PTAR1	protein prenyltransferase alpha subunit repeat containing 1	9	72324438	72374875	50438
PTCRA	pre T-cell antigen receptor alpha	6	42883727	42893573	9847
PTEN	phosphatase and tensin homolog	10	89622870	89731687	108818
**PTGER3**	prostaglandin E receptor 3	1	71318036	71513491	195456
PTGS1	prostaglandin-endoperoxide synthase 1	9	125132824	125157982	25159
PTGS2	prostaglandin-endoperoxide synthase 2	1	186640923	186649559	8637
PTK2	protein tyrosine kinase 2	8	141667999	142012315	344317
PTK7	protein tyrosine kinase 7 (inactive)	6	43044006	43129457	85452

PTN	pleiotrophin	7	136912088	137028611	116524
PTPDC1	protein tyrosine phosphatase domain containing 1	9	96793076	96872138	79063
PTPN1	protein tyrosine phosphatase, non-receptor type 1	20	49126891	49201299	74409
PTPN11	protein tyrosine phosphatase, non-receptor type 11	12	112856155	112947717	91563
PTPN13	protein tyrosine phosphatase, non-receptor type 13	4	87515468	87736324	220857
PTPN14	protein tyrosine phosphatase, non-receptor type 14	1	214522039	214725792	203754
PTPN21	protein tyrosine phosphatase, non-receptor type 21	14	88932122	89021077	88956
PTPN4	protein tyrosine phosphatase, non-receptor type 4	2	120517207	120741394	224188
PTPRC	protein tyrosine phosphatase, receptor type C	1	198607801	198726545	118745
PTPRD	protein tyrosine phosphatase, receptor type D	9	8314246	10612723	2298478
PTPRE	protein tyrosine phosphatase, receptor type E	10	129705325	129884119	178795
PTPRG	protein tyrosine phosphatase, receptor type G	3	61547243	62283288	736046
PTPRK	protein tyrosine phosphatase, receptor type K	6	128289924	128841870	551947
**PTPRM**	protein tyrosine phosphatase, receptor type M	18	7566780	8406859	840080
PTPRN2	protein tyrosine phosphatase, receptor type N2	7	157331750	158380480	1048731
PTPRQ	protein tyrosine phosphatase, receptor type Q	12	80799774	81072802	273029
PTPRR	protein tyrosine phosphatase, receptor type R	12	71031853	71314623	282771
**PTPRT**	protein tyrosine phosphatase, receptor type T	20	40701392	41818610	1117219
PTPRZ1	protein tyrosine phosphatase, receptor type Z1	7	121513143	121702090	188948
PTTG1IP	PTTG1 interacting protein	21	46269500	46293752	24253
PUM1	pumilio RNA binding family member 1	1	31404353	31538838	134486
PURG	purine rich element binding protein G	8	30853321	30891231	37911
PUS10	pseudouridylate synthase 10	2	61167357	61245394	78038
**PVR**	poliovirus receptor	19	45147098	45166850	19753
PVT1	Pvt1 oncogene (non-protein coding)	8	128806779	129113499	306721
PXDNL	peroxidasin like	8	52232138	52722005	489868
PXK	PX domain containing serine/threonine kinase like	3	58318607	58411748	93142
PXT1	peroxisomal, testis specific 1	6	36358328	36410666	52339
PYGO1	pygopus family PHD finger 1	15	55831088	55881145	50058
PYHIN1	pyrin and HIN domain family member 1	1	158900586	158946844	46259
PYY	peptide YY	17	42030106	42081837	51732
PZP	PZP, alpha-2-macroglobulin like	12	9301436	9360966	59531
QRICH2	glutamine rich 2	17	74270130	74303761	33632
R3HCC1L	R3H domain and coiled-coil containing 1 like	10	99894387	100004654	110268
R3HDM1	R3H domain containing 1	2	136289025	136482840	193816
R3HDM2	R3H domain containing 2	12	57643392	57824788	181397
RAB18	RAB18, member RAS oncogene family	10	27793197	27831143	37947
RAB22A	RAB22A, member RAS oncogene family	20	56884752	56942563	57812
RAB25	RAB25, member RAS oncogene family	1	156030951	156040295	9345
RAB27A	RAB27A, member RAS oncogene family	15	55495164	55611311	116148
RAB31	RAB31, member RAS oncogene family	18	9708162	9862548	154387
RAB37	RAB37, member RAS oncogene family	17	72666717	72743474	76758
RAB38	RAB38, member RAS oncogene family	11	87846410	87908635	62226
RAB39B	RAB39B, member RAS oncogene family	X	154487526	154493874	6349
RAB3A	RAB3A, member RAS oncogene family	19	18307594	18314884	7291
RAB3GAP1	RAB3 GTPase activating protein catalytic subunit 1	2	135809835	135933964	124130
RAB3GAP2	RAB3 GTPase activating non-catalytic protein subunit 2	1	220321635	220445796	124162
RAB40C	RAB40C, member RAS oncogene family	16	639357	679272	39916
RAB44	RAB44, member RAS oncogene family	6	36683256	36699870	16615
RAB7A	RAB7A, member RAS oncogene family	3	128444965	128533639	88675
RAB8A	RAB8A, member RAS oncogene family	19	16222439	16245044	22606
**RAB9A**	RAB9A, member RAS oncogene family	X	13707244	13728625	21382
RABGAP1L	RAB GTPase activating protein 1 like	1	174128548	174964445	835898
RABL3	RAB, member of RAS oncogene family like 3	3	120405528	120461840	56313
RAC2	ras-related C3 botulinum toxin substrate 2 (rho family, small GTP binding protein Rac2)	22	37621301	37640488	19188
RAD18	RAD18, E3 ubiquitin protein ligase	3	8817088	9005457	188370
RAD21	RAD21 cohesin complex component	8	117858174	117887105	28932
RAD50	RAD50 double strand break repair protein	5	131891711	131980313	88603
RAD51	RAD51 recombinase	15	40986972	41024354	37383
RAD51B	RAD51 paralog B	14	68286496	69196935	910440
RADIL	Rap associating with DIL domain	7	4836686	4923350	86665
RAET1E-AS1	RAET1E antisense RNA 1	6	150205567	150240644	35078
RAI1	retinoic acid induced 1	17	17584787	17714767	129981
RAI14	retinoic acid induced 14	5	34656342	34832732	176391
RALB	RAS like proto-oncogene B	2	120997640	121052289	54650
RALBP1	ralA binding protein 1	18	9475007	9538114	63108
**RALGAPA1**	Ral GTPase activating protein catalytic alpha subunit 1	14	36007558	36278510	270953
RALGAPA2	Ral GTPase activating protein catalytic alpha subunit 2	20	20370196	20693131	322936
RALGPS1	Ral GEF with PH domain and SH3 binding motif 1	9	129677053	129985445	308393
RALY	RALY heterogeneous nuclear ribonucleoprotein	20	32581452	32696114	114663
RALYL	RALY RNA binding protein-like	8	85095022	85834079	739058
**RAMP3**	receptor activity modifying protein 3	7	45197390	45225901	28512
RANBP17	RAN binding protein 17	5	170288874	170727019	438146
RAP1GAP2	RAP1 GTPase activating protein 2	17	2680350	2941033	260684
RAP1GDS1	Rap1 GTPase-GDP dissociation stimulator 1	4	99182535	99365012	182478
RAP2C	RAP2C, member of RAS oncogene family	X	131337053	131353471	16419

RAPGEF1	Rap guanine nucleotide exchange factor 1	9	134452157	134615461	163305
RAPGEF4	Rap guanine nucleotide exchange factor 4	2	173600002	173917621	317620
RAPGEF5	Rap guanine nucleotide exchange factor 5	7	22157856	22396763	238908
RAPGEF6	Rap guanine nucleotide exchange factor 6	5	130759614	130970929	211316
**RARB**	retinoic acid receptor beta	3	25215823	25639423	423601
RARS2	arginyl-tRNA synthetase 2, mitochondrial	6	88224096	88299721	75626
RASA2	RAS p21 protein activator 2	3	141205889	141334184	128296
RASA3	RAS p21 protein activator 3	13	114747194	114898086	150893
RASAL2	RAS protein activator like 2	1	178062864	178448644	385781
RASD2	RASD family member 2	22	35936915	35950048	13134
RASGRF1	Ras protein specific guanine nucleotide releasing factor 1	15	79252289	79383115	130827
RASGRF2	Ras protein specific guanine nucleotide releasing factor 2	5	80256491	80525975	269485
RASGRP1	RAS guanyl releasing protein 1	15	38780304	38857776	77473
RASGRP3	RAS guanyl releasing protein 3	2	33661391	33789817	128427
RASSF3	Ras association domain family member 3	12	65004293	65091347	87055
RASSF4	Ras association domain family member 4	10	45454855	45491339	36485
RASSF5	Ras association domain family member 5	1	206680879	206762616	81738
RBBP6	RB binding protein 6, ubiquitin ligase	16	24549014	24584184	35171
RBBP7	RB binding protein 7, chromatin remodeling factor	X	16857406	16888537	31132
RBFOX1	RNA binding protein, fox-1 homolog 1	16	6069095	7763340	1694246
RBFOX2	RNA binding protein, fox-1 homolog 2	22	36134783	36424473	289691
RBFOX3	RNA binding protein, fox-1 homolog 3	17	77085427	77613550	528124
RBKS	ribokinase	2	28004231	28113965	109735
RBL1	RB transcriptional corepressor like 1	20	35624752	35724398	99647
RBM18	RNA binding motif protein 18	9	124999903	125027118	27216
RBM26	RNA binding motif protein 26	13	79885962	79980612	94651
RBM27	RNA binding motif protein 27	5	145583113	145718814	135702
RBM33	RNA binding motif protein 33	7	155437145	155574179	137035
RBM4	RNA binding motif protein 4	11	66384097	66434153	50057
RBM47	RNA binding motif protein 47	4	40425272	40632892	207621
RBM6	RNA binding motif protein 6	3	49977440	50137478	160039
**RBMS1**	RNA binding motif single stranded interacting protein 1	2	161128662	161350305	221644
RBMS3	RNA binding motif single stranded interacting protein 3	3	29322473	30051886	729414
RBP4	retinol binding protein 4	10	95351444	95361501	10058
RBPJ	recombination signal binding protein for immunoglobulin kappa J region	4	26165077	26436541	271465
RCAN1	regulator of calcineurin 1	21	35885440	35987441	102002
RCAN2	regulator of calcineurin 2	6	46188475	46459709	271235
RCAN3	RCAN family member 3	1	24829387	24867530	38144
RCC2	regulator of chromosome condensation 2	1	17733256	17766220	32965
RCOR1	REST corepressor 1	14	103058998	103196913	137916
RCOR3	REST corepressor 3	1	211431719	211489727	58009
RD3	retinal degeneration 3	1	211649864	211666259	16396
RDH13	retinol dehydrogenase 13	19	55550476	55582659	32184
RDX	radixin	11	110045605	110167447	121843
RECK	reversion inducing cysteine rich protein with kazal motifs	9	36036430	36124448	88019
**RECQL**	RecQ like helicase	12	21621845	21654603	32759
REEP1	receptor accessory protein 1	2	86441116	86565206	124091
REEP2	receptor accessory protein 2	5	137774706	137782658	7953
REEP3	receptor accessory protein 3	10	65281123	65384883	103761
REG4	regenerating family member 4	1	120336641	120354283	17643
REL	REL proto-oncogene, NF-kB subunit	2	61108656	61158745	50090
RELA	RELA proto-oncogene, NF-kB subunit	11	65421067	65430565	9499
RELB	RELB proto-oncogene, NF-kB subunit	19	45504688	45541452	36765
RELN	reelin	7	103112231	103629963	517733
REN	renin	1	204123944	204135465	11522
REPS1	RALBP1 associated Eps domain containing 1	6	139224630	139309398	84769
REPS2	RALBP1 associated Eps domain containing 2	X	16964814	17171395	206582
RER1	retention in endoplasmic reticulum sorting receptor 1	1	2323267	2336883	13617
RERE	arginine-glutamic acid dipeptide repeats	1	8412457	8877702	465246
RET	ret proto-oncogene	10	43572475	43625799	53325
REV3L	REV3 like, DNA directed polymerase zeta catalytic subunit	6	111620234	111804918	184685
RFC3	replication factor C subunit 3	13	34392186	34540695	148510
RFTN1	raftlin, lipid raft linker 1	3	16355081	16555533	200453
RFTN2	raftlin family member 2	2	198432948	198540769	107822
RFX1	regulatory factor X1	19	14072350	14117851	45502
RFX2	regulatory factor X2	19	5993175	6199583	206409
RFX3	regulatory factor X3	9	3218297	3526004	307708
RFX6	regulatory factor X6	6	117198375	117253326	54952
RFX7	regulatory factor X7	15	56379478	56535483	156006
RFX8	RFX family member 8, lacking RFX DNA binding domain	2	102013823	102091165	77343
RGPD1	RANBP2-like and GRIP domain containing 1	2	87135076	87241104	106029
RGS12	regulator of G protein signaling 12	4	3294755	3441640	146886
RGS17	regulator of G protein signaling 17	6	153325594	153452384	126791
RGS20	regulator of G protein signaling 20	8	54764368	54871863	107496
RGS21	regulator of G protein signaling 21	1	192286122	192336415	50294
RGS22	regulator of G protein signaling 22	8	100973164	101143496	170333

RGS4	regulator of G protein signaling 4	1	163038565	163046592	8028
RGS5	regulator of G protein signaling 5	1	163080911	163291577	210667
RGS6	regulator of G protein signaling 6	14	72399156	73030654	631499
RGS7	regulator of G protein signaling 7	1	240931554	241520530	588977
**RHAG**	Rh-associated glycoprotein	6	49572871	49604552	31682
RHBDD2	rhomboid domain containing 2	7	75471920	75518244	46325
RHCG	Rh family C glycoprotein	15	89998680	90039844	41165
RHEB	Ras homolog enriched in brain	7	151163098	151217206	54109
RHOA	ras homolog family member A	3	49396578	49450431	53854
RHOBTB1	Rho related BTB domain containing 1	10	62629196	62761198	132003
RHOJ	ras homolog family member J	14	63670832	63759937	89106
RHOQ	ras homolog family member Q	2	46768945	46810260	41316
RHOU	ras homolog family member U	1	228870824	228882416	11593
RIC8B	RIC8 guanine nucleotide exchange factor B	12	107168373	107283090	114718
RICTOR	RPTOR independent companion of MTOR complex 2	5	38938021	39074510	136490
RIMS1	regulating synaptic membrane exocytosis 1	6	72596406	73112845	516440
**RIMS2**	regulating synaptic membrane exocytosis 2	8	104512976	105268322	755347
RIMS4	regulating synaptic membrane exocytosis 4	20	43380449	43438979	58531
RIOK2	RIO kinase 2	5	96496571	96518964	22394
RIT2	Ras like without CAAX 2	18	40323192	40695657	372466
RLF	rearranged L-myc fusion	1	40627045	40706593	79549
**RNASE2**	ribonuclease A family member 2	14	21423611	21424595	985
RNASEH2A	ribonuclease H2 subunit A	19	12917394	12924452	7059
RNASEH2B	ribonuclease H2 subunit B	13	51483814	51544592	60779
RNASEH2B-AS1	RNASEH2B antisense RNA 1	13	51450822	51484848	34027
RNASEH2C	ribonuclease H2 subunit C	11	65482367	65488418	6052
RNF10	ring finger protein 10	12	120971283	121015397	44115
RNF103-CHMP3	RNF103-CHMP3 readthrough	2	86732791	86948245	215455
RNF11	ring finger protein 11	1	51701943	51739127	37185
RNF113A	ring finger protein 113A	X	119004497	119005791	1295
RNF114	ring finger protein 114	20	48552948	48570429	17482
RNF123	ring finger protein 123	3	49726932	49758962	32031
RNF128	ring finger protein 128, E3 ubiquitin protein ligase	X	105937024	106040223	103200
RNF130	ring finger protein 130	5	179338651	179499118	160468
RNF135	ring finger protein 135	17	29295803	29326929	31127
RNF138	ring finger protein 138	18	29671818	29711524	39707
RNF145	ring finger protein 145	5	158584417	158637061	52645
RNF149	ring finger protein 149	2	101887681	101925163	37483
RNF152	ring finger protein 152	18	59475296	59561480	86185
RNF17	ring finger protein 17	13	25338290	25454059	115770
RNF170	ring finger protein 170	8	42704780	42752433	47654
RNF180	ring finger protein 180	5	63461671	63668696	207026
RNF185	ring finger protein 185	22	31556168	31603005	46838
**RNF19A**	ring finger protein 19A, RBR E3 ubiquitin protein ligase	8	101269288	101348446	79159
RNF19B	ring finger protein 19B	1	33402046	33430286	28241
RNF217	ring finger protein 217	6	125283691	125413779	130089
RNF219-AS1	RNF219 antisense RNA 1	13	78493824	79191463	697640
RNF31	ring finger protein 31	14	24615892	24629870	13979
RNF32	ring finger protein 32	7	156432975	156469824	36850
RNF41	ring finger protein 41	12	56598285	56615717	17433
RNF43	ring finger protein 43	17	56429861	56494956	65096
RNFT2	ring finger protein, transmembrane 2	12	117176096	117291436	115341
**RNGTT**	RNA guanylyltransferase and 5'-phosphatase	6	89319985	89673348	353364
RNLS	renalase, FAD dependent amine oxidase	10	90033621	90344287	310667
RNU5F-1	RNA, U5F small nuclear 1	1	45187458	45187574	117
RNU6-19P	RNA, U6 small nuclear 19, pseudogene	15	65845419	65845525	107
RNU6-53P	RNA, U6 small nuclear 53, pseudogene	13	29277839	29277944	106
RNU6-71P	RNA, U6 small nuclear 71, pseudogene	13	36841305	36841411	107
RNU6-83P	RNA, U6 small nuclear 83, pseudogene	13	99677488	99677599	112
ROBO1	roundabout guidance receptor 1	3	78646390	79816965	1170576
ROBO2	roundabout guidance receptor 2	3	75955846	77699115	1743270
ROCK1	Rho associated coiled-coil containing protein kinase 1	18	18526867	18691812	164946
ROCK2	Rho associated coiled-coil containing protein kinase 2	2	11319887	11488456	168570
ROGDI	rogdi homolog	16	4846969	4852951	5983
ROR1	receptor tyrosine kinase like orphan receptor 1	1	64239693	64647181	407489
ROR2	receptor tyrosine kinase like orphan receptor 2	9	94325373	94712444	387072
RORA	RAR related orphan receptor A	15	60780483	61521518	741036
RORB	RAR related orphan receptor B	9	77112281	77308093	195813
RORC	RAR related orphan receptor C	1	151778547	151804348	25802
ROS1	ROS proto-oncogene 1, receptor tyrosine kinase	6	117609463	117747018	137556
RPA2	replication protein A2	1	28218035	28241257	23223
RPGRIP1L	RPGRIP1 like	16	53631595	53737850	106256
RPH3A	rabphilin 3A	12	113008184	113336686	328503
RPN1	ribophorin I	3	128338817	128399918	61102
RPRD1A	regulation of nuclear pre-mRNA domain containing 1A	18	33564350	33647539	83190
RPS23	ribosomal protein S23	5	81569177	81574396	5220
RPS25	ribosomal protein S25	11	118886422	118889401	2980

RPS6KA2	ribosomal protein S6 kinase A2	6	166822852	167319939	497088
RPS6KA5	ribosomal protein S6 kinase A5	14	91336799	91526980	190182
RPS6KA6	ribosomal protein S6 kinase A6	X	83318984	83442933	123950
RPS6KC1	ribosomal protein S6 kinase C1	1	213224589	213448116	223528
RPTOR	regulatory associated protein of MTOR complex 1	17	78518619	78940171	421553
RRAGC	Ras related GTP binding C	1	39303870	39325495	21626
RRAGD	Ras related GTP binding D	6	90074355	90121989	47635
RRAS2	related RAS viral (r-ras) oncogene homolog 2	11	14299472	14386052	86581
RRP15	ribosomal RNA processing 15 homolog	1	218458629	218511325	52697
RRP8	ribosomal RNA processing 8, methyltransferase, homolog (yeast)	11	6616305	6624850	8546
RS1	retinoschisin 1	X	18658030	18690229	32200
RSF1	remodeling and spacing factor 1	11	77371041	77532063	161023
RSPO2	R-spondin 2	8	108911544	109095913	184370
RSPRY1	ring finger and SPRY domain containing 1	16	57220049	57274387	54339
RSRC1	arginine and serine rich coiled-coil 1	3	157823644	158263519	439876
RSU1	Ras suppressor protein 1	10	16632610	16859527	226918
RTN1	reticulon 1	14	60062694	60337684	274991
RTN2	reticulon 2	19	45988547	46000319	11773
RTN3	reticulon 3	11	63448918	63527363	78446
RUFY2	RUN and FYVE domain containing 2	10	70100864	70167051	66188
RUFY3	RUN and FYVE domain containing 3	4	71569921	71673032	103112
RUNX1	runt related transcription factor 1	21	36160098	37376965	1216868
RUNX1T1	RUNX1 translocation partner 1	8	92967203	93115514	148312
RUNX2	runt related transcription factor 2	6	45295894	45632086	336193
RUNX3	runt related transcription factor 3	1	25226002	25291612	65611
RXFP1	relaxin/insulin like family peptide receptor 1	4	159236463	159574524	338062
RXRA	retinoid X receptor alpha	9	137208944	137332431	123488
RXRB	retinoid X receptor beta	6	33161365	33168630	7266
RXRG	retinoid X receptor gamma	1	165370159	165414433	44275
RYK	receptor-like tyrosine kinase	3	133784147	133969689	185543
RYR2	ryanodine receptor 2	1	237205505	237997288	791784
RYR3	ryanodine receptor 3	15	33603163	34158303	555141
S100A10	S100 calcium binding protein A10	1	151955391	151966866	11476
S100A14	S100 calcium binding protein A14	1	153586731	153589462	2732
S100B	S100 calcium binding protein B	21	48018875	48025121	6247
S100Z	S100 calcium binding protein Z	5	76145826	76217475	71650
SACM1L	SAC1 suppressor of actin mutations 1 like (yeast)	3	45730548	45786916	56369
SACS	sacsin molecular chaperone	13	23902965	24007841	104877
SAFB2	scaffold attachment factor B2	19	5587010	5624057	37048
SAMD12	sterile alpha motif domain containing 12	8	119201698	119634234	432537
SAMD3	sterile alpha motif domain containing 3	6	130465460	130686570	221111
SAMD4A	sterile alpha motif domain containing 4A	14	55033815	55260033	226219
SAMHD1	SAM and HD domain containing deoxynucleoside triphosphate triphosphohydrolase 1	20	35518632	35580246	61615
SAMSN1	SAM domain, SH3 domain and nuclear localization signals 1	21	15857549	15955723	98175
SAP130	Sin3A associated protein 130	2	128698791	128785694	86904
SAP30	Sin3A associated protein 30	4	174291120	174298683	7564
SAP30BP	SAP30 binding protein	17	73663196	73704142	40947
SAR1A	secretion associated Ras related GTPase 1A	10	71907045	71930279	23235
**SARM1**	sterile alpha and TIR motif containing 1	17	26691378	26728065	36688
SASH1	SAM and SH3 domain containing 1	6	148593440	148873186	279747
SATB1	SATB homeobox 1	3	18386879	18487080	100202
SATB2	SATB homeobox 2	2	200134223	200335989	201767
SBF2	SET binding factor 2	11	9800214	10315754	515541
SBF2-AS1	SBF2 antisense RNA 1	11	9779839	9832866	53028
SBNO2	strawberry notch homolog 2	19	1107636	1174282	66647
SC5D	sterol-C5-desaturase	11	121163162	121179403	16242
SCAF1	SR-related CTD associated factor 1	19	50145382	50161899	16518
SCAF11	SR-related CTD associated factor 11	12	46312914	46385903	72990
SCAF4	SR-related CTD associated factor 4	21	33043346	33104388	61043
SCAF8	SR-related CTD associated factor 8	6	155054459	155155192	100734
SCAI	suppressor of cancer cell invasion	9	127704887	127905785	200899
SCAP	SREBF chaperone	3	47455203	47518616	63414
SCAPER	S-phase cyclin A associated protein in the ER	15	76640526	77197785	557260
SCARB1	scavenger receptor class B member 1	12	125261402	125367214	105813
SCARB2	scavenger receptor class B member 2	4	77079890	77135046	55157
SCCPDH	saccharopine dehydrogenase (putative)	1	246887349	246931439	44091
SCD	stearoyl-CoA desaturase	10	102106881	102124591	17711
SCEL	sciellin	13	78109809	78219398	109590
SCFD1	sec1 family domain containing 1	14	31091318	31205018	113701
SCFD2	sec1 family domain containing 2	4	53739149	54232242	493094
SCG5	secretogranin V	15	32933877	32989299	55423
SCGB1D1	secretoglobin family 1D member 1	11	61957688	61961011	3324
SCLT1	sodium channel and clathrin linker 1	4	129786076	130014764	228689
SCN10A	sodium voltage-gated channel alpha subunit 10	3	38738293	38835501	97209
SCN1A	sodium voltage-gated channel alpha subunit 1	2	166845670	166984523	138854
SCN1B	sodium voltage-gated channel beta subunit 1	19	35521588	35531352	9765

SCN2A	sodium voltage-gated channel alpha subunit 2	2	166095912	166248818	152907
SCN3A	sodium voltage-gated channel alpha subunit 3	2	165944032	166060577	116546
**SCN7A**	sodium voltage-gated channel alpha subunit 7	2	167260083	167350757	90675
SCN8A	sodium voltage-gated channel alpha subunit 8	12	51984050	52206648	222599
SCN9A	sodium voltage-gated channel alpha subunit 9	2	167051695	167232503	180809
SCNN1B	sodium channel epithelial 1 beta subunit	16	23289552	23392620	103069
SCOC	short coiled-coil protein	4	141178440	141306880	128441
SCP2	sterol carrier protein 2	1	53392901	53517375	124475
SCUBE1	signal peptide, CUB domain and EGF like domain containing 1	22	43593289	43739394	146106
SCUBE2	signal peptide, CUB domain and EGF like domain containing 2	11	9041071	9159661	118591
SCUBE3	signal peptide, CUB domain and EGF like domain containing 3	6	35182190	35220856	38667
SDC1	syndecan 1	2	20400558	20425194	24637
SDCCAG8	serologically defined colon cancer antigen 8	1	243419320	243663394	244075
**SDK1**	sidekick cell adhesion molecule 1	7	3341080	4308632	967553
SDK2	sidekick cell adhesion molecule 2	17	71330523	71640228	309706
SDR16C6P	short chain dehydrogenase/reductase family 16C member 6, pseudogene	8	57285623	57307924	22302
SEC14L1	SEC14 like lipid binding 1	17	75082798	75213179	130382
SEC14L2	SEC14 like lipid binding 2	22	30792846	30821305	28460
SEC16A	SEC16 homolog A, endoplasmic reticulum export factor	9	139334549	139372141	37593
SEC22C	SEC22 homolog C, vesicle trafficking protein	3	42589461	42642572	53112
SEC23A	Sec23 homolog A, coat complex II component	14	39501123	39578850	77728
SEC24C	SEC24 homolog C, COPII coat complex component	10	75504120	75531919	27800
SEC24D	SEC24 homolog D, COPII coat complex component	4	119643978	119759838	115861
SEL1L2	SEL1L2 ERAD E3 ligase adaptor subunit	20	13829893	13977089	147197
**SELL**	selectin L	1	169659808	169680839	21032
SELP	selectin P	1	169558087	169599431	41345
SEMA3A	semaphorin 3A	7	83585093	84122040	536948
SEMA3E	semaphorin 3E	7	82993222	83278326	285105
SEMA4D	semaphorin 4D	9	91975702	92113045	137344
SEMA5A	semaphorin 5A	5	9035138	9546187	511050
**SEMA6A**	semaphorin 6A	5	115779312	115910630	131319
SEMA6D	semaphorin 6D	15	47476298	48066420	590123
SENP1	SUMO1/sentrin specific peptidase 1	12	48436681	48500091	63411
SENP6	SUMO1/sentrin specific peptidase 6	6	76311225	76427997	116773
SEPSECS	Sep (O-phosphoserine) tRNA:Sec (selenocysteine) tRNA synthase	4	25121627	25162204	40578
SEPT3	septin 3	22	42372276	42394225	21950
SEPT9	septin 9	17	75276651	75496678	220028
SERAC1	serine active site containing 1	6	158530536	158589312	58777
SERGEF	secretion regulating guanine nucleotide exchange factor	11	17809595	18034709	225115
SERINC5	serine incorporator 5	5	79407050	79551898	144849
SERPINA1	serpin family A member 1	14	94843084	94857030	13947
SERPINA13P	serpin family A member 13, pseudogene	14	95107062	95113331	6270
SERPINA3	serpin family A member 3	14	95078714	95090392	11679
SERPINA7	serpin family A member 7	X	105277197	105282729	5533
SERPINB1	serpin family B member 1	6	2832566	2842240	9675
SERPINB5	serpin family B member 5	18	61143994	61172318	28325
SERPINB7	serpin family B member 7	18	61420169	61472604	52436
SERPINE1	serpin family E member 1	7	100770370	100782547	12178
SERPINE2	serpin family E member 2	2	224839829	224904036	64208
SERPINE3	serpin family E member 3	13	51909909	51938871	28963
SERPINF2	serpin family F member 2	17	1646130	1658562	12433
SERTAD4	SERTA domain containing 4	1	210406144	210419976	13833
SESN1	sestrin 1	6	109307640	109416022	108383
SESTD1	SEC14 and spectrin domain containing 1	2	179966419	180129517	163099
SET	SET nuclear proto-oncogene	9	131445703	131458679	12977
SETBP1	SET binding protein 1	18	42260138	42648475	388338
SETD2	SET domain containing 2	3	47057919	47205457	147539
SETD3	SET domain containing 3	14	99864083	99947216	83134
**SETX**	senataxin	9	135136743	135230372	93630
SEZ6L	seizure related 6 homolog like	22	26565440	26779562	214123
SEZ6L2	seizure related 6 homolog like 2	16	29882480	29910868	28389
SF1	splicing factor 1	11	64532078	64546258	14181
SF3A1	splicing factor 3a subunit 1	22	30727977	30752936	24960
SF3B1	splicing factor 3b subunit 1	2	198254508	198299815	45308
SFMBT2	Scm-like with four mbt domains 2	10	7200586	7453450	252865
SFRP1	secreted frizzled related protein 1	8	41119481	41167016	47536
SFRP2	secreted frizzled related protein 2	4	154701744	154710272	8529
SFSWAP	splicing factor SWAP homolog	12	132195626	132284282	88657
SFTA3	surfactant associated 3	14	36942493	36983034	40542
SFTPA1	surfactant protein A1	10	81370695	81375196	4502
SFXN5	sideroflexin 5	2	73169165	73302747	133583
SGCA	sarcoglycan alpha	17	48241575	48253292	11718
SGCB	sarcoglycan beta	4	52886872	52904648	17777
SGCD	sarcoglycan delta	5	155297354	156194799	897446
SGCG	sarcoglycan gamma	13	23755091	23899304	144214
SGCZ	sarcoglycan zeta	8	13947373	15095848	1148476

SGK1	serum/glucocorticoid regulated kinase 1	6	134490384	134639250	148867
SGMS1	sphingomyelin synthase 1	10	52065360	52384923	319564
SGPL1	sphingosine-1-phosphate lyase 1	10	72575717	72640930	65214
SGPP1	sphingosine-1-phosphate phosphatase 1	14	64150932	64194757	43826
SGTB	small glutamine rich tetratricopeptide repeat containing beta	5	64961755	65018862	57108
SH2B1	SH2B adaptor protein 1	16	28857921	28885526	27606
SH2D2A	SH2 domain containing 2A	1	156776035	156786654	10620
SH2D4A	SH2 domain containing 4A	8	19171128	19253729	82602
SH2D4B	SH2 domain containing 4B	10	82297658	82406316	108659
SH3GL2	SH3 domain containing GRB2 like 2, endophilin A1	9	17579080	17797127	218048
SH3GL3	SH3 domain containing GRB2 like 3, endophilin A3	15	84115980	84287495	171516
SH3GLB2	SH3 domain containing GRB2 like, endophilin B2	9	131769315	131790582	21268
SH3KBP1	SH3 domain containing kinase binding protein 1	X	19552093	19905719	353627
SH3RF1	SH3 domain containing ring finger 1	4	170015407	170192256	176850
SH3RF2	SH3 domain containing ring finger 2	5	145316142	145461354	145213
SH3RF3	SH3 domain containing ring finger 3	2	109745804	110262207	516404
SH3TC2	SH3 domain and tetratricopeptide repeats 2	5	148303202	148442726	139525
SHANK2	SH3 and multiple ankyrin repeat domains 2	11	70313961	70963623	649663
SHC3	SHC adaptor protein 3	9	91628060	91793682	165623
SHC4	SHC adaptor protein 4	15	49115932	49255641	139710
SHCBP1L	SHC binding and spindle associated 1 like	1	182869000	182922660	53661
SHH	sonic hedgehog	7	155592680	155604967	12288
SHISA6	shisa family member 6	17	11144580	11467380	322801
SHISA9	shisa family member 9	16	12995477	13334272	338796
SHQ1	SHQ1, H/ACA ribonucleoprotein assembly factor	3	72798428	72911065	112638
SHROOM2	shroom family member 2	X	9754496	9917483	162988
SHROOM3	shroom family member 3	4	77356253	77704406	348154
SHROOM4	shroom family member 4	X	50334647	50557302	222656
SIAH2	siah E3 ubiquitin protein ligase 2	3	150458914	150481264	22351
SIGLEC8	sialic acid binding Ig like lectin 8	19	51954101	51961710	7610
SIGLEC9	sialic acid binding Ig like lectin 9	19	51628165	51639908	11744
**SIGMAR1**	sigma non-opioid intracellular receptor 1	9	34634719	34637806	3088
SIK3	SIK family kinase 3	11	116714118	116969153	255036
SIL1	SIL1 nucleotide exchange factor	5	138282409	138629246	346838
SIN3A	SIN3 transcription regulator family member A	15	75661720	75748183	86464
SIPA1L1	signal induced proliferation associated 1 like 1	14	71787166	72207946	420781
SIPA1L3	signal induced proliferation associated 1 like 3	19	38397868	38699012	301145
SIRPG	signal regulatory protein gamma	20	1609798	1638425	28628
SIRT1	sirtuin 1	10	69644427	69678147	33721
SIRT3	sirtuin 3	11	215458	236931	21474
SKA2	spindle and kinetochore associated complex subunit 2	17	57187312	57232630	45319
SKAP1	src kinase associated phosphoprotein 1	17	46210802	46507637	296836
SKAP2	src kinase associated phosphoprotein 2	7	26706681	27034858	328178
SKOR2	SKI family transcriptional corepressor 2	18	44746293	44775554	29262
SKP1	S-phase kinase associated protein 1	5	133484633	133512729	28097
SLC10A1	solute carrier family 10 member 1	14	70242134	70264006	21873
SLC11A2	solute carrier family 11 member 2	12	51373184	51422349	49166
SLC12A2	solute carrier family 12 member 2	5	127419458	127525380	105923
SLC12A6	solute carrier family 12 member 6	15	34525460	34630261	104802
SLC13A3	solute carrier family 13 member 3	20	45186463	45304714	118252
SLC14A2	solute carrier family 14 member 2	18	42792960	43263072	470113
SLC15A5	solute carrier family 15 member 5	12	16341419	16430619	89201
SLC16A10	solute carrier family 16 member 10	6	111408781	111552397	143617
SLC16A12	solute carrier family 16 member 12	10	91190051	91316398	126348
SLC16A13	solute carrier family 16 member 13	17	6939394	6943440	4047
SLC16A14	solute carrier family 16 member 14	2	230899698	230933715	34018
SLC16A4	solute carrier family 16 member 4	1	110905470	110933704	28235
SLC17A3	solute carrier family 17 member 3	6	25833294	25882514	49221
SLC17A5	solute carrier family 17 member 5	6	74303102	74363878	60777
SLC17A7	solute carrier family 17 member 7	19	49932658	49945617	12960
SLC18A2	solute carrier family 18 member A2	10	119000604	119038941	38338
SLC19A3	solute carrier family 19 member 3	2	228549926	228582728	32803
**SLC1A2**	solute carrier family 1 member 2	11	35272753	35441610	168858
SLC1A3	solute carrier family 1 member 3	5	36606457	36688436	81980
SLC20A2	solute carrier family 20 member 2	8	42273993	42397069	123077
SLC22A10	solute carrier family 22 member 10	11	62905339	63137190	231852
SLC22A2	solute carrier family 22 member 2	6	160592093	160698670	106578
SLC22A23	solute carrier family 22 member 23	6	3269196	3457256	188061
SLC22A3	solute carrier family 22 member 3	6	160769300	160876014	106715
SLC22A8	solute carrier family 22 member 8	11	62756626	62783311	26686
SLC23A2	solute carrier family 23 member 2	20	4833002	4990939	157938
SLC24A2	solute carrier family 24 member 2	9	19507450	19786926	279477
SLC24A3	solute carrier family 24 member 3	20	19193290	19703581	510292
SLC25A13	solute carrier family 25 member 13	7	95749532	95951459	201928
SLC25A15	solute carrier family 25 member 15	13	41363548	41384247	20700
SLC25A19	solute carrier family 25 member 19	17	73269073	73285591	16519
SLC25A21	solute carrier family 25 member 21	14	37147636	37642071	494436

SLC25A22	solute carrier family 25 member 22	11	790475	798316	7842
SLC25A25	solute carrier family 25 member 25	9	130830480	130871524	41045
SLC25A27	solute carrier family 25 member 27	6	46620678	46645930	25253
**SLC25A43**	solute carrier family 25 member 43	X	118533023	118588441	55419
SLC25A46	solute carrier family 25 member 46	5	110073837	110100857	27021
SLC25A48	solute carrier family 25 member 48	5	135170338	135224326	53989
SLC26A4	solute carrier family 26 member 4	7	107301080	107358254	57175
SLC26A7	solute carrier family 26 member 7	8	92221722	92410378	188657
SLC26A8	solute carrier family 26 member 8	6	35911291	35992645	81355
SLC27A6	solute carrier family 27 member 6	5	127873706	128369335	495630
SLC2A1	solute carrier family 2 member 1	1	43391052	43424530	33479
SLC2A10	solute carrier family 2 member 10	20	45338126	45364965	26840
SLC2A13	solute carrier family 2 member 13	12	40148823	40499891	351069
SLC2A9	solute carrier family 2 member 9	4	9772777	10056560	283784
SLC30A6	solute carrier family 30 member 6	2	32390933	32449448	58516
SLC33A1	solute carrier family 33 member 1	3	155538813	155572218	33406
SLC35A1	solute carrier family 35 member A1	6	88180341	88222054	41714
SLC35A2	solute carrier family 35 member A2	X	48760459	48769235	8777
SLC35A3	solute carrier family 35 member A3	1	100435345	100492535	57191
SLC35C1	solute carrier family 35 member C1	11	45825623	45834566	8944
SLC35F1	solute carrier family 35 member F1	6	118228689	118638839	410151
SLC35F3	solute carrier family 35 member F3	1	234040679	234460262	419584
SLC35F4	solute carrier family 35 member F4	14	58030640	58448912	418273
SLC37A1	solute carrier family 37 member 1	21	43916118	44001550	85433
SLC37A3	solute carrier family 37 member 3	7	139993493	140104233	110741
SLC38A1	solute carrier family 38 member 1	12	46576846	46663800	86955
SLC38A11	solute carrier family 38 member 11	2	165752696	165812035	59340
SLC38A4	solute carrier family 38 member 4	12	47158546	47226191	67646
SLC38A6	solute carrier family 38 member 6	14	61447832	61550451	102620
**SLC39A11**	solute carrier family 39 member 11	17	70642088	71088851	446764
SLC39A12	solute carrier family 39 member 12	10	18240768	18332221	91454
SLC39A7	solute carrier family 39 member 7	6	33168222	33172216	3995
SLC39A9	solute carrier family 39 member 9	14	69864732	69929105	64374
SLC41A1	solute carrier family 41 member 1	1	205758221	205782876	24656
SLC41A3	solute carrier family 41 member 3	3	125725198	125820404	95207
SLC44A5	solute carrier family 44 member 5	1	75667816	76076801	408986
SLC45A3	solute carrier family 45 member 3	1	205626979	205649587	22609
SLC46A1	solute carrier family 46 member 1	17	26721661	26734215	12555
SLC46A3	solute carrier family 46 member 3	13	29274201	29293107	18907
SLC4A10	solute carrier family 4 member 10	2	162280843	162841792	560950
SLC4A4	solute carrier family 4 member 4	4	72053003	72437804	384802
SLC4A5	solute carrier family 4 member 5	2	74443369	74570541	127173
SLC4A7	solute carrier family 4 member 7	3	27414214	27525911	111698
SLC4A8	solute carrier family 4 member 8	12	51785101	51902980	117880
SLC5A12	solute carrier family 5 member 12	11	26688566	26744974	56409
SLC5A3	solute carrier family 5 member 3	21	35445870	35478561	32692
SLC5A7	solute carrier family 5 member 7	2	108602979	108630450	27472
SLC5A8	solute carrier family 5 member 8	12	101549271	101604185	54915
SLC6A1	solute carrier family 6 member 1	3	11034410	11080933	46524
SLC6A14	solute carrier family 6 member 14	X	115567790	115592625	24836
SLC6A16	solute carrier family 6 member 16	19	49792895	49828482	35588
SLC6A19	solute carrier family 6 member 19	5	1201710	1225232	23523
SLC6A2	solute carrier family 6 member 2	16	55689516	55740104	50589
SLC6A3	solute carrier family 6 member 3	5	1392909	1445545	52637
SLC6A4	solute carrier family 6 member 4	17	28521337	28563020	41684
SLC6A5	solute carrier family 6 member 5	11	20620946	20680831	59886
SLC7A1	solute carrier family 7 member 1	13	30083547	30169825	86279
SLC7A13	solute carrier family 7 member 13	8	87226281	87333375	107095
SLC7A14	solute carrier family 7 member 14	3	170177372	170303863	126492
SLC8A1-AS1	SLC8A1 antisense RNA 1	2	40013593	40482349	468757
SLC8A3	solute carrier family 8 member A3	14	70510934	70655787	144854
SLC9A1	solute carrier family 9 member A1	1	27425306	27493472	68167
SLC9A6	solute carrier family 9 member A6	X	135067598	135129423	61826
SLC9A7	solute carrier family 9 member A7	X	46464753	46618490	153738
SLC9A8	solute carrier family 9 member A8	20	48429250	48508779	79530
SLC9A9	solute carrier family 9 member A9	3	142984064	143567373	583310
SLC9C1	solute carrier family 9 member C1	3	111859734	112013105	153372
SLC9C2	solute carrier family 9 member C2 (putative)	1	173469603	173572233	102631
SLCO1A2	solute carrier organic anion transporter family member 1A2	12	21417534	21572528	154995
**SLCO1B1**	solute carrier organic anion transporter family member 1B1	12	21284136	21392180	108045
SLCO3A1	solute carrier organic anion transporter family member 3A1	15	92396925	92715665	318741
**SLCO4C1**	solute carrier organic anion transporter family member 4C1	5	101569690	101632253	62564
SLCO5A1	solute carrier organic anion transporter family member 5A1	8	70579282	70747299	168018
SLIT2	slit guidance ligand 2	4	20254883	20622184	367302
SLIT3	slit guidance ligand 3	5	168088745	168728133	639389
**SLK**	STE20 like kinase	10	105726959	105788991	62033
SLMAP	sarcolemma associated protein	3	57741177	57914895	173719

SMAD1	SMAD family member 1	4	146402346	146479231	76886
SMAD2	SMAD family member 2	18	45357922	45457515	99594
SMAD3	SMAD family member 3	15	67356101	67487533	131433
SMAD9	SMAD family member 9	13	37418968	37494902	75935
SMARCB1	SWI/SNF related, matrix associated, actin dependent regulator of chromatin, subfamily b, member 1	22	24129150	24176703	47554
SMARCC2	SWI/SNF related, matrix associated, actin dependent regulator of chromatin subfamily c member 2	12	56556767	56583351	26585
SMC1B	structural maintenance of chromosomes 1B	22	45739944	45809500	69557
SMC3	structural maintenance of chromosomes 3	10	112327449	112364394	36946
SMC4	structural maintenance of chromosomes 4	3	160117062	160152750	35689
SMCHD1	structural maintenance of chromosomes flexible hinge domain containing 1	18	2655737	2805015	149279
SMCR8	Smith-Magenis syndrome chromosome region, candidate 8	17	18218624	18226517	7894
SMG1	SMG1, nonsense mediated mRNA decay associated PI3K related kinase	16	18816175	18937776	121602
SMG6	SMG6, nonsense mediated mRNA decay factor	17	1963133	2207065	243933
**SMN1**	survival of motor neuron 1, telomeric	5	70220768	70249769	29002
SMOC2	SPARC related modular calcium binding 2	6	168841831	169073984	232154
SMOX	spermine oxidase	20	4101627	4168394	66768
SMS	spermine synthase	X	21958691	22025798	67108
SMURF1	SMAD specific E3 ubiquitin protein ligase 1	7	98625061	98741723	116663
SMYD3	SET and MYND domain containing 3	1	245912642	246670614	757973
SMYD4	SET and MYND domain containing 4	17	1682779	1733928	51150
SNAP91	synaptosome associated protein 91	6	84262599	84419410	156812
SNCA	synuclein alpha	4	90645250	90759466	114217
SNCAIP	synuclein alpha interacting protein	5	121647049	121799914	152866
SNCB	synuclein beta	5	176047085	176057530	10446
**SNCG**	synuclein gamma	10	88718375	88723017	4643
SND1	staphylococcal nuclease and tudor domain containing 1	7	127292234	127732661	440428
SNRNP200	small nuclear ribonucleoprotein U5 subunit 200	2	96940074	96971297	31224
SNRNP40	small nuclear ribonucleoprotein U5 subunit 40	1	31732417	31769662	37246
SNRPB2	small nuclear ribonucleoprotein polypeptide B2	20	16710606	16722421	11816
SNRPN	small nuclear ribonucleoprotein polypeptide N	15	25068794	25223870	155077
SNTB1	syntrophin beta 1	8	121547985	121825513	277529
SNTG1	syntrophin gamma 1	8	50822349	51706678	884330
SNTG2	syntrophin gamma 2	2	946554	1371385	424832
SNX17	sorting nexin 17	2	27593389	27599995	6607
SNX18	sorting nexin 18	5	53813589	53842415	28827
SNX2	sorting nexin 2	5	122110691	122165803	55113
SNX24	sorting nexin 24	5	122179134	122365049	185916
SNX25	sorting nexin 25	4	186125391	186291339	165949
**SNX29**	sorting nexin 29	16	12070594	12668146	597553
SNX29P2	sorting nexin 29 pseudogene 2	16	29262829	29519817	256989
SNX30	sorting nexin family member 30	9	115513118	115643951	130834
SNX4	sorting nexin 4	3	125165495	125239041	73547
SNX9	sorting nexin 9	6	158244296	158366109	121814
SOAT1	sterol O-acyltransferase 1	1	179262925	179327815	64891
SOBP	sine oculis binding protein homolog	6	107811162	107981357	170196
**SOD1**	superoxide dismutase 1	21	33031935	33041244	9310
**SOD2**	superoxide dismutase 2	6	160090089	160183561	93473
SORBS1	sorbin and SH3 domain containing 1	10	97071528	97321171	249644
SORBS2	sorbin and SH3 domain containing 2	4	186506598	186877806	371209
SORCS1	sortilin related VPS10 domain containing receptor 1	10	108333421	108924292	590872
SORCS2	sortilin related VPS10 domain containing receptor 2	4	7194265	7744554	550290
**SORCS3**	sortilin related VPS10 domain containing receptor 3	10	106400859	107024993	624135
SORD	sorbitol dehydrogenase	15	45315302	45369383	54082
SORL1	sortilin related receptor 1	11	121322912	121504402	181491
SOS1	SOS Ras/Rac guanine nucleotide exchange factor 1	2	39208537	39351486	142950
SOS2	SOS Ras/Rho guanine nucleotide exchange factor 2	14	50583847	50698276	114430
SOX2-OT	SOX2 overlapping transcript	3	180707558	181554668	847111
SOX4	SRY-box 4	6	21593972	21598847	4876
**SOX5**	SRY-box 5	12	23682440	24103966	421527
SOX6	SRY-box 6	11	15987995	16761138	773144
SOX9-AS1	SOX9 antisense RNA 1	17	70033455	70217977	184523
SP1	Sp1 transcription factor	12	53773960	53810230	36271
SP140	SP140 nuclear body protein	2	231067826	231223762	155937
SPAG16	sperm associated antigen 16	2	214149113	215275225	1126113
SPAG5-AS1	SPAG5 antisense RNA 1	17	26925808	26944393	18586
SPAG9	sperm associated antigen 9	17	49039535	49198226	158692
SPANXA2-OT1	SPANXA2 overlapping transcript 1	X	140590843	140738057	147215
**SPAST**	spastin	2	32288680	32382706	94027
SPATA13	spermatogenesis associated 13	13	24553944	24881212	327269
SPATA16	spermatogenesis associated 16	3	172607148	172859058	251911
SPATA17	spermatogenesis associated 17	1	217804666	218045038	240373
SPATA18	spermatogenesis associated 18	4	52917497	52963458	45962
SPATA5	spermatogenesis associated 5	4	123844229	124240605	396377
SPATA6	spermatogenesis associated 6	1	48761044	48937845	176802

SPATS2	spermatogenesis associated serine rich 2	12	49760367	49921205	160839
SPCS2	signal peptidase complex subunit 2	11	74660292	74690076	29785
SPECC1L	sperm antigen with calponin homology and coiled-coil domains 1 like	22	24666786	24813708	146923
SPECC1L-ADORA2A	SPECC1L-ADORA2A readthrough (NMD candidate)	22	24666866	24838324	171459
SPEF2	sperm flagellar 2	5	35617946	35814713	196768
SPEN	spen family transcriptional repressor	1	16174359	16266955	92597
**SPG11**	SPG11, spatacsin vesicle trafficking associated	15	44854894	44955876	100983
SPG20	spastic paraplegia 20 (Troyer syndrome)	13	36875775	36944317	68543
SPG21	SPG21, maspardin	15	65255362	65282648	27287
**SPG7**	SPG7, paraplegin matrix AAA peptidase subunit	16	89557325	89624176	66852
SPIDR	scaffolding protein involved in DNA repair	8	48173167	48648868	475702
SPINK1	serine peptidase inhibitor, Kazal type 1	5	147204131	147211349	7219
SPINK5	serine peptidase inhibitor, Kazal type 5	5	147405246	147516852	111607
SPIRE1	spire type actin nucleation factor 1	18	12446511	12658133	211623
**SPOCK1**	SPARC/osteonectin, cwcv and kazal like domains proteoglycan 1	5	136310987	136934068	623082
SPOCK3	SPARC/osteonectin, cwcv and kazal like domains proteoglycan 3	4	167654535	168155947	501413
SPON1	spondin 1	11	13983914	14289646	305733
SPOP	speckle type BTB/POZ protein	17	47676246	47755596	79351
SPPL3	signal peptide peptidase like 3	12	121200313	121342174	141862
SPR	sepiapterin reductase (7,8-dihydrobiopterin:NADP+ oxidoreductase)	2	73114489	73119287	4799
SPRED2	sprouty related EVH1 domain containing 2	2	65537985	65659771	121787
SPRY4	sprouty RTK signaling antagonist 4	5	141689992	141706020	16029
SPTB	spectrin beta, erythrocytic	14	65213002	65346601	133600
SPTBN1	spectrin beta, non-erythrocytic 1	2	54683422	54896812	213391
SPTBN2	spectrin beta, non-erythrocytic 2	11	66452719	66496697	43979
SPTBN4	spectrin beta, non-erythrocytic 4	19	40972148	41082370	110223
SPTLC1	serine palmitoyltransferase long chain base subunit 1	9	94794281	94877666	83386
SPTLC2	serine palmitoyltransferase long chain base subunit 2	14	77972340	78083116	110777
SPTLC3	serine palmitoyltransferase long chain base subunit 3	20	12989627	13147411	157785
SPTSSA	serine palmitoyltransferase small subunit A	14	34901995	34931562	29568
SPTY2D1	SPT2 chromatin protein domain containing 1	11	18627948	18656338	28391
**SQSTM1**	sequestosome 1	5	179233388	179265078	31691
SRBD1	S1 RNA binding domain 1	2	45615819	45839304	223486
SRC	SRC proto-oncogene, non-receptor tyrosine kinase	20	35973088	36034453	61366
SRCIN1	SRC kinase signaling inhibitor 1	17	36686251	36762183	75933
SRD5A1	steroid 5 alpha-reductase 1	5	6633456	6669675	36220
SRD5A2	steroid 5 alpha-reductase 2	2	31747550	31806136	58587
SREBF1	sterol regulatory element binding transcription factor 1	17	17713713	17740325	26613
SREBF2	sterol regulatory element binding transcription factor 2	22	42229109	42303312	74204
SRGAP1	SLIT-ROBO Rho GTPase activating protein 1	12	64238073	64541613	303541
SRGAP3	SLIT-ROBO Rho GTPase activating protein 3	3	9022275	9404737	382463
SRPK2	SRSF protein kinase 2	7	104751151	105039755	288605
SRPX	sushi repeat containing protein, X-linked	X	38008589	38080696	72108
SRPX2	sushi repeat containing protein, X-linked 2	X	99899215	99926296	27082
SRRM4	serine/arginine repetitive matrix 4	12	119419300	119600856	181557
SRSF3	serine and arginine rich splicing factor 3	6	36562145	36573377	11233
SRSF4	serine and arginine rich splicing factor 4	1	29474255	29508499	34245
**SS18L1**	SS18L1, nBAF chromatin remodeling complex subunit	20	60718822	60757540	38719
SSBP2	single stranded DNA binding protein 2	5	80708840	81047616	338777
SSBP3	single stranded DNA binding protein 3	1	54691105	54879152	188048
SSH2	slingshot protein phosphatase 2	17	27952956	28257294	304339
SST	somatostatin	3	187386694	187388187	1494
SSU72	SSU72 homolog, RNA polymerase II CTD phosphatase	1	1477053	1510249	33197
**SSX2IP**	SSX family member 2 interacting protein	1	85109390	85156486	47097
SSX5	SSX family member 5	X	48045656	48056199	10544
ST18	ST18, C2H2C-type zinc finger	8	53023399	53373519	350121
ST3GAL3	ST3 beta-galactoside alpha-2,3-sialyltransferase 3	1	44171495	44396831	225337
ST3GAL5	ST3 beta-galactoside alpha-2,3-sialyltransferase 5	2	86066267	86116137	49871
**ST3GAL6**	ST3 beta-galactoside alpha-2,3-sialyltransferase 6	3	98451080	98540045	88966
ST5	suppression of tumorigenicity 5	11	8714898	8932498	217601
ST6GAL2	ST6 beta-galactoside alpha-2,6-sialyltransferase 2	2	107418056	107503564	85509
ST6GALNAC3	ST6 N-acetylgalactosaminide alpha-2,6-sialyltransferase 3	1	76540404	77100286	559883
ST6GALNAC5	ST6 N-acetylgalactosaminide alpha-2,6-sialyltransferase 5	1	77333126	77531396	198271
ST7	suppression of tumorigenicity 7	7	116593292	116870157	276866
STAB2	stabilin 2	12	103981051	104160505	179455
STAC	SH3 and cysteine rich domain	3	36421836	36589499	167664
STAG1	stromal antigen 1	3	136055077	136471220	416144
STAG2	stromal antigen 2	X	123094062	123556514	462453
STAG3	stromal antigen 3	7	99775186	99819111	43926
STAM	signal transducing adaptor molecule	10	17686124	17757913	71790
STAM2	signal transducing adaptor molecule 2	2	152973315	153032506	59192
STAMBP	STAM binding protein	2	74056086	74100786	44701
STAP1	signal transducing adaptor family member 1	4	68424446	68473055	48610
STARD10	StAR related lipid transfer domain containing 10	11	72465774	72504726	38953
**STARD13**	StAR related lipid transfer domain containing 13	13	33677272	33924767	247496
STARD4-AS1	STARD4 antisense RNA 1	5	110847924	111075423	227500
STAT3	signal transducer and activator of transcription 3	17	40465342	40540586	75245

STAT4	signal transducer and activator of transcription 4	2	191894302	192016322	122021
STAU1	staufen double-stranded RNA binding protein 1	20	47729878	47804904	75027
STAU2	staufen double-stranded RNA binding protein 2	8	74332604	74659943	327340
STEAP2	STEAP2 metalloreductase	7	89796904	89867451	70548
STIL	SCL/TAL1 interrupting locus	1	47715811	47779819	64009
STIM1	stromal interaction molecule 1	11	3875757	4114439	238683
STIM2	stromal interaction molecule 2	4	26859300	27027003	167704
STK11	serine/threonine kinase 11	19	1189406	1228428	39023
STK16	serine/threonine kinase 16	2	220110177	220115059	4883
STK24	serine/threonine kinase 24	13	99102455	99230194	127740
STK3	serine/threonine kinase 3	8	99413631	99955055	541425
STK32B	serine/threonine kinase 32B	4	5053169	5502725	449557
**STK39**	serine/threonine kinase 39	2	168810530	169104651	294122
STK4	serine/threonine kinase 4	20	43595115	43708600	113486
STON1-GTF2A1L	STON1-GTF2A1L readthrough	2	48757064	49003654	246591
STON2	stonin 2	14	81727000	81902809	175810
STPG1	sperm tail PG-rich repeat containing 1	1	24683489	24743424	59936
STPG2	sperm tail PG-rich repeat containing 2	4	98105244	99064391	959148
STRADA	STE20-related kinase adaptor alpha	17	61780192	61819330	39139
STRBP	spermatid perinuclear RNA binding protein	9	125871779	126030855	159077
STRN	striatin	2	37070783	37193615	122833
STRN3	striatin 3	14	31363005	31495607	132603
**STS**	steroid sulfatase	X	7137497	7272851	135355
**STX17**	syntaxin 17	9	102668915	102732618	63704
STX18	syntaxin 18	4	4417469	4544073	126605
STX8	syntaxin 8	17	9153788	9479908	326121
STXBP1	syntaxin binding protein 1	9	130374544	130457460	82917
STXBP4	syntaxin binding protein 4	17	53046088	53241646	195559
STXBP5-AS1	STXBP5 antisense RNA 1	6	147163037	147525750	362714
STXBP5L	syntaxin binding protein 5 like	3	120626919	121143608	516690
STXBP6	syntaxin binding protein 6	14	25278862	25519503	240642
STYK1	serine/threonine/tyrosine kinase 1	12	10771538	10826917	55380
SUCLG2	succinate-CoA ligase GDP-forming beta subunit	3	67410884	67705038	294155
SUDS3	SDS3 homolog, SIN3A corepressor complex component	12	118814185	118855840	41656
SUFU	SUFU negative regulator of hedgehog signaling	10	104263744	104393292	129549
SUGCT	succinyl-CoA:glutarate-CoA transferase	7	40174575	40900362	725788
SUGP2	SURP and G-patch domain containing 2	19	19101697	19144832	43136
SULT1C3	sulfotransferase family 1C member 3	2	108863651	108881807	18157
**SUN3**	Sad1 and UNC84 domain containing 3	7	48026745	48068716	41972
SUPT3H	SPT3 homolog, SAGA and STAGA complex component	6	44777054	45345690	568637
SUPV3L1	Suv3 like RNA helicase	10	70939988	70968855	28868
SUSD4	sushi domain containing 4	1	223394161	223537544	143384
SUSD5	sushi domain containing 5	3	33191537	33260707	69171
SUZ12	SUZ12 polycomb repressive complex 2 subunit	17	30264037	30328064	64028
SV2B	synaptic vesicle glycoprotein 2B	15	91643180	91844539	201360
SVEP1	sushi, von Willebrand factor type A, EGF and pentraxin domain containing 1	9	113127531	113342160	214630
SVIL	supervillin	10	29746267	30025710	279444
SVOP	SV2 related protein	12	109304658	109459045	154388
SWT1	SWT1, RNA endoribonuclease homolog	1	185126212	185260897	134686
SYAP1	synapse associated protein 1	X	16737755	16783459	45705
SYCP1	synaptonemal complex protein 1	1	115397424	115537991	140568
SYCP2	synaptonemal complex protein 2	20	58438618	58508710	70093
SYF2	SYF2 pre-mRNA splicing factor	1	25549170	25558993	9824
**SYK**	spleen associated tyrosine kinase	9	93564069	93660831	96763
SYN1	synapsin I	X	47431303	47479252	47950
SYN2	synapsin II	3	12045876	12232900	187025
**SYN3**	synapsin III	22	32908539	33454358	545820
SYNCRIP	synaptotagmin binding cytoplasmic RNA interacting protein	6	86318053	86353510	35458
SYNDIG1	synapse differentiation inducing 1	20	24449835	24647252	197418
**SYNE1**	spectrin repeat containing nuclear envelope protein 1	6	152442819	152958936	516118
SYNE2	spectrin repeat containing nuclear envelope protein 2	14	64319683	64693165	373483
SYNGAP1	synaptic Ras GTPase activating protein 1	6	33387847	33421466	33620
SYNJ1	synaptojanin 1	21	34001069	34100359	99291
SYNPO2	synaptopodin 2	4	119809996	119982402	172407
SYNPR	synaptoporin	3	63213991	63602597	388607
SYT1	synaptotagmin 1	12	79257773	79845788	588016
SYT10	synaptotagmin 10	12	33527173	33592754	65582
SYT14	synaptotagmin 14	1	210111538	210337636	226099
SYT16	synaptotagmin 16	14	62453803	62568431	114629
**SYT9**	synaptotagmin 9	11	7260009	7490273	230265
SYTL2	synaptotagmin like 2	11	85405267	85522184	116918
SYTL4	synaptotagmin like 4	X	99929488	99987110	57623
SZT2	seizure threshold 2 homolog (mouse)	1	43855553	43918321	62769
TAAR2	trace amine associated receptor 2 (gene/pseudogene)	6	132938161	132945414	7254
TACC2	transforming acidic coiled-coil containing protein 2	10	123748689	124014060	265372
TACR1	tachykinin receptor 1	2	75273590	75426826	153237

TACR3	tachykinin receptor 3	4	104507188	104640973	133786
**TAF15**	TATA-box binding protein associated factor 15	17	34136459	34191619	55161
TAF3	TATA-box binding protein associated factor 3	10	7860467	8058590	198124
TAF7	TATA-box binding protein associated factor 7	5	140698057	140700330	2274
TAGAP	T-cell activation RhoGTPase activating protein	6	159455500	159466184	10685
TANC1	tetratricopeptide repeat, ankyrin repeat and coiled-coil containing 1	2	159825146	160089170	264025
TANGO6	transport and golgi organization 6 homolog	16	68877507	69119083	241577
TAOK1	TAO kinase 1	17	27717482	27878922	161441
TAP2	transporter 2, ATP binding cassette subfamily B member	6	32781544	32806599	25056
TAPBPL	TAP binding protein like	12	6560856	6575683	14828
TARBP1	TAR (HIV-1) RNA binding protein 1	1	234527059	234614849	87791
**TARDBP**	TAR DNA binding protein	1	11072414	11085796	13383
TASP1	taspase 1	20	13246709	13619587	372879
TATDN2	TatD DNase domain containing 2	3	10289707	10322902	33196
TBC1D1	TBC1 domain family member 1	4	37892708	38140796	248089
TBC1D16	TBC1 domain family member 16	17	77906142	78009647	103506
TBC1D2	TBC1 domain family member 2	9	100961311	101017915	56605
TBC1D22A	TBC1 domain family member 22A	22	47158518	47571336	412819
TBC1D24	TBC1 domain family member 24	16	2525147	2555735	30589
TBC1D2B	TBC1 domain family member 2B	15	78276378	78370066	93689
TBC1D32	TBC1 domain family member 32	6	121400640	121655891	255252
TBC1D4	TBC1 domain family member 4	13	75858808	76056250	197443
**TBC1D5**	TBC1 domain family member 5	3	17198654	18486309	1287656
TBC1D9	TBC1 domain family member 9	4	141541919	141677274	135356
TBCA	tubulin folding cofactor A	5	76986991	77164604	177614
TBCK	TBC1 domain containing kinase	4	106962756	107242652	279897
**TBK1**	TANK binding kinase 1	12	64845660	64895888	50229
TBL1X	transducin beta like 1X-linked	X	9431335	9687780	256446
TBL1Y	transducin beta like 1, Y-linked	Y	6778727	6959724	180998
TBP	TATA-box binding protein	6	170863390	170881958	18569
TBX18	T-box 18	6	85397069	85474237	77169
TBX5	T-box 5	12	114791736	114846247	54512
TC2N	tandem C2 domains, nuclear	14	92246095	92333880	87786
TCAIM	T-cell activation inhibitor, mitochondrial	3	44379611	44450943	71333
TCAP	titin-cap	17	37820440	37822808	2369
TCERG1L	transcription elongation regulator 1 like	10	132890654	133109984	219331
TCF12	transcription factor 12	15	57210821	57591479	380659
TCF4	transcription factor 4	18	52889562	53332018	442457
TCF7L1	transcription factor 7 like 1	2	85360533	85537511	176979
TCF7L2	transcription factor 7 like 2	10	114710009	114927437	217429
TCL1A	T-cell leukemia/lymphoma 1A	14	96176304	96180533	4230
TCN1	transcobalamin 1	11	59620273	59634048	13776
TCN2	transcobalamin 2	22	31002825	31023265	20441
TDP1	tyrosyl-DNA phosphodiesterase 1	14	90421283	90511106	89824
TDP2	tyrosyl-DNA phosphodiesterase 2	6	24650205	24667261	17057
TDRD15	tudor domain containing 15	2	21346789	21366144	19356
TDRD7	tudor domain containing 7	9	100174232	100258407	84176
TDRD9	tudor domain containing 9	14	104394799	104519004	124206
TDRP	testis development related protein	8	439803	495781	55979
TEAD1	TEA domain transcription factor 1	11	12695969	12966298	270330
TECPR2	tectonin beta-propeller repeat containing 2	14	102829300	102968818	139519
TECR	trans-2,3-enoyl-CoA reductase	19	14627897	14676792	48896
TECTA	tectorin alpha	11	120971882	121062202	90321
TECTB	tectorin beta	10	114043493	114064793	21301
TEF	TEF, PAR bZIP transcription factor	22	41763337	41795330	31994
TEK	TEK receptor tyrosine kinase	9	27109139	27230173	121035
TEKT5	tektin 5	16	10721358	10788802	67445
TENM1	teneurin transmembrane protein 1	X	123509753	124097666	587914
TENM2	teneurin transmembrane protein 2	5	166711804	167691162	979359
TENM3	teneurin transmembrane protein 3	4	183065140	183724177	659038
TENM4	teneurin transmembrane protein 4	11	78363876	79151992	788117
TEPP	testis, prostate and placenta expressed	16	58010339	58022020	11682
TERF2	telomeric repeat binding factor 2	16	69389464	69442474	53011
TET1	tet methylcytosine dioxygenase 1	10	70320413	70454239	133827
TET2	tet methylcytosine dioxygenase 2	4	106067032	106200973	133942
TEX11	testis expressed 11	X	69748790	70128581	379792
TEX2	testis expressed 2	17	62224587	62340661	116075
TEX41	testis expressed 41 (non-protein coding)	2	145425534	145940216	514683
TF	transferrin	3	133464800	133497850	33051
TFAM	transcription factor A, mitochondrial	10	60144782	60158981	14200
TFAP2D	transcription factor AP-2 delta	6	50681541	50740701	59161
TFB1M	transcription factor B1, mitochondrial	6	155578643	155635627	56985
TFB2M	transcription factor B2, mitochondrial	1	246703862	246729626	25765
TFCP2	transcription factor CP2	12	51487446	51566926	79481
TFDP1	transcription factor Dp-1	13	114239013	114295785	56773
TFDP2	transcription factor Dp-2	3	141663277	141868386	205110
TFR2	transferrin receptor 2	7	100218039	100240402	22364

TG	thyroglobulin	8	133879203	134147147	267945
TGFA	transforming growth factor alpha	2	70674412	70781325	106914
TGFB1	transforming growth factor beta 1	19	41807492	41859816	52325
TGFBR3	transforming growth factor beta receptor 3	1	92145902	92371892	225991
TGFBRAP1	transforming growth factor beta receptor associated protein 1	2	105880871	105946491	65621
TGM6	transglutaminase 6	20	2361554	2413399	51846
TGS1	trimethylguanosine synthase 1	8	56685701	56738007	52307
TH	tyrosine hydroxylase	11	2185159	2193107	7949
**THADA**	THADA, armadillo repeat containing	2	43393800	43823185	429386
THBD	thrombomodulin	20	23026270	23030378	4109
THBS2	thrombospondin 2	6	169615875	169654139	38265
THEMIS	thymocyte selection associated	6	128029217	128239776	210560
**THOC1**	THO complex 1	18	214520	268050	53531
THOC2	THO complex 2	X	122734412	122866906	132495
THRA	thyroid hormone receptor, alpha	17	38214543	38250120	35578
THRB	thyroid hormone receptor beta	3	24158651	24536773	378123
THSD1	thrombospondin type 1 domain containing 1	13	52951305	52980629	29325
THSD4	thrombospondin type 1 domain containing 4	15	71389291	72075722	686432
THSD7A	thrombospondin type 1 domain containing 7A	7	11409984	11871824	461841
THSD7B	thrombospondin type 1 domain containing 7B	2	137523115	138435287	912173
TIAM1	T-cell lymphoma invasion and metastasis 1	21	32490734	32932290	441557
TIAM2	T-cell lymphoma invasion and metastasis 2	6	155153831	155578857	425027
TIMM23B	translocase of inner mitochondrial membrane 23 homolog B	10	51371390	51387768	16379
**TIMP2**	TIMP metallopeptidase inhibitor 2	17	76849059	76921469	72411
TINAG	tubulointerstitial nephritis antigen	6	54172657	54254950	82294
TIPARP	TCDD inducible poly(ADP-ribose) polymerase	3	156391024	156424559	33536
TIPIN	TIMELESS interacting protein	15	66628544	66679084	50541
TJP1	tight junction protein 1	15	29991571	30261068	269498
TJP2	tight junction protein 2	9	71736209	71870124	133916
TJP3	tight junction protein 3	19	3708107	3750811	42705
TK2	thymidine kinase 2, mitochondrial	16	66541906	66586447	44542
TLDC1	TBC/LysM-associated domain containing 1	16	84511681	84587639	75959
TLDC2	TBC/LysM-associated domain containing 2	20	35504524	35522638	18115
TLK1	tousled like kinase 1	2	171847333	172087824	240492
TLK2	tousled like kinase 2	17	60536019	60692842	156824
TLL1	tolloid like 1	4	166794410	167025047	230638
**TLN2**	talin 2	15	62682725	63136830	454106
TLR4	toll like receptor 4	9	120466610	120479149	12540
TLX1NB	TLX1 neighbor	10	102849078	102890883	41806
TM9SF3	transmembrane 9 superfamily member 3	10	98277866	98347209	69344
TMC1	transmembrane channel like 1	9	75136717	75451267	314551
TMC5	transmembrane channel like 5	16	19421818	19510435	88618
TMCC1	transmembrane and coiled-coil domain family 1	3	129366635	129612419	245785
TMCC3	transmembrane and coiled-coil domain family 3	12	94960900	95044338	83439
TMED5	transmembrane p24 trafficking protein 5	1	93615299	93646285	30987
TMED6	transmembrane p24 trafficking protein 6	16	69377151	69385712	8562
TMEFF2	transmembrane protein with EGF like and two follistatin like domains 2	2	192813769	193060435	246667
TMEM106B	transmembrane protein 106B	7	12250867	12282993	32127
TMEM108	transmembrane protein 108	3	132757235	133116636	359402
TMEM117	transmembrane protein 117	12	44229770	44783545	553776
TMEM131	transmembrane protein 131	2	98372799	98612388	239590
TMEM132B	transmembrane protein 132B	12	125671382	126146917	475536
TMEM132C	transmembrane protein 132C	12	128751948	129192460	440513
TMEM132D	transmembrane protein 132D	12	129556270	130388211	831942
TMEM135	transmembrane protein 135	11	86748886	87034800	285915
TMEM138	transmembrane protein 138	11	61129473	61136981	7509
TMEM161B	transmembrane protein 161B	5	87485450	87565293	79844
TMEM161B-AS1	TMEM161B antisense RNA 1	5	87564712	87732502	167791
TMEM163	transmembrane protein 163	2	135213330	135476570	263241
TMEM165	transmembrane protein 165	4	56262124	56319564	57441
TMEM17	transmembrane protein 17	2	62727356	62739029	11674
TMEM170A	transmembrane protein 170A	16	75476952	75499395	22444
TMEM178A	transmembrane protein 178A	2	39892122	39945103	52982
TMEM178B	transmembrane protein 178B	7	140774032	141180180	406149
TMEM19	transmembrane protein 19	12	72079867	72097836	17970
TMEM192	transmembrane protein 192	4	165995574	166129701	134128
TMEM2	transmembrane protein 2	9	74298282	74431606	133325
TMEM216	transmembrane protein 216	11	61159159	61166335	7177
TMEM217	transmembrane protein 217	6	37179956	37225931	45976
TMEM218	transmembrane protein 218	11	124966398	124981659	15262
TMEM219	transmembrane protein 219	16	29952206	29984373	32168
TMEM231	transmembrane protein 231	16	75572015	75590184	18170
TMEM232	transmembrane protein 232	5	109624934	110074657	449724
TMEM241	transmembrane protein 241	18	20777108	21017925	240818
TMEM242	transmembrane protein 242	6	157710418	157744633	34216
TMEM26	transmembrane protein 26	10	63166401	63213208	46808

TMEM260	transmembrane protein 260	14	56955072	57117324	162253
TMEM5	transmembrane protein 5	12	64173583	64203338	29756
TMEM52B	transmembrane protein 52B	12	10323141	10344400	21260
TMEM59	transmembrane protein 59	1	54497347	54519177	21831
TMEM65	transmembrane protein 65	8	125324231	125384933	60703
TMEM67	transmembrane protein 67	8	94767072	94831462	64391
TMEM68	transmembrane protein 68	8	56608983	56685966	76984
TMEM70	transmembrane protein 70	8	74884672	74895018	10347
TMEM71	transmembrane protein 71	8	133697253	133772958	75706
TMEM97	transmembrane protein 97	17	26646121	26655707	9587
TMEM98	transmembrane protein 98	17	31254928	31272124	17197
TMF1	TATA element modulatory factor 1	3	69068978	69101484	32507
TMLHE	trimethyllysine hydroxylase, epsilon	X	154719776	154899605	179830
TMOD3	tropomodulin 3	15	52121825	52239492	117668
TMPRSS11F	transmembrane protease, serine 11F	4	68918916	68995598	76683
TMPRSS15	transmembrane protease, serine 15	21	19641433	19858197	216765
TMPRSS2	transmembrane protease, serine 2	21	42836478	42903043	66566
TMPRSS4-AS1	TMPRSS4 antisense RNA 1	11	117886487	117957508	71022
TMPRSS6	transmembrane protease, serine 6	22	37461476	37505603	44128
TMTC1	transmembrane and tetratricopeptide repeat containing 1	12	29653773	29937692	283920
TMTC2	transmembrane and tetratricopeptide repeat containing 2	12	83080659	83528649	447991
TMX3	thioredoxin related transmembrane protein 3	18	66340925	66382535	41611
TNC	tenascin C	9	117782806	117880536	97731
TNF	tumor necrosis factor	6	31543344	31546113	2770
TNFAIP8	TNF alpha induced protein 8	5	118604387	118735383	130997
TNFAIP8L3	TNF alpha induced protein 8 like 3	15	51348795	51397473	48679
TNFRSF10B	TNF receptor superfamily member 10b	8	22877646	22926692	49047
TNFRSF1A	TNF receptor superfamily member 1A	12	6437923	6451280	13358
TNFRSF1B	TNF receptor superfamily member 1B	1	12227060	12269285	42226
TNFSF18	TNF superfamily member 18	1	173009100	173020103	11004
TNIK	TRAF2 and NCK interacting kinase	3	170779128	171178197	399070
**TNIP3**	TNFAIP3 interacting protein 3	4	122052563	122148621	96059
TNK1	tyrosine kinase non receptor 1	17	7283853	7293093	9241
TNK2	tyrosine kinase non receptor 2	3	195590235	195638816	48582
TNKS	tankyrase	8	9413424	9639856	226433
TNMD	tenomodulin	X	99839799	99854882	15084
TNPO3	transportin 3	7	128594948	128695198	100251
TNR	tenascin R	1	175284330	175712906	428577
TNRC6A	trinucleotide repeat containing 6A	16	24741016	24838953	97938
TNRC6B	trinucleotide repeat containing 6B	22	40440821	40731812	290992
TNRC6C	trinucleotide repeat containing 6C	17	76000249	76104916	104668
TNS3	tensin 3	7	47314752	47622156	307405
TOM1L2	target of myb1 like 2 membrane trafficking protein	17	17746828	17875736	128909
TOMM40	translocase of outer mitochondrial membrane 40	19	45393826	45406946	13121
TOP2A	topoisomerase (DNA) II alpha	17	38544768	38574202	29435
TOP2B	topoisomerase (DNA) II beta	3	25639475	25706398	66924
TOP3A	topoisomerase (DNA) III alpha	17	18174742	18218321	43580
TOR1A	torsin family 1 member A	9	132575223	132586413	11191
TOR1AIP2	torsin 1A interacting protein 2	1	179809102	179846938	37837
TOX	thymocyte selection associated high mobility group box	8	59717977	60031767	313791
TOX2	TOX high mobility group box family member 2	20	42543504	42698256	154753
TOX3	TOX high mobility group box family member 3	16	52471917	52581714	109798
TP53	tumor protein p53	17	7565097	7590856	25760
TP63	tumor protein p63	3	189349205	189615068	265864
TP73	tumor protein p73	1	3569084	3652765	83682
TPD52L1	tumor protein D52 like 1	6	125440195	125585553	145359
TPH1	tryptophan hydroxylase 1	11	18039111	18063973	24863
TPK1	thiamin pyrophosphokinase 1	7	144149034	144533488	384455
TPM2	tropomyosin 2 (beta)	9	35681989	35691017	9029
TPO	thyroid peroxidase	2	1377995	1547483	169489
TPP1	tripeptidyl peptidase 1	11	6634000	6640692	6693
TPRG1	tumor protein p63 regulated 1	3	188665003	189043093	378091
TPRKB	TP53RK binding protein	2	73956231	73964527	8297
TPRXL	tetrapeptide repeat homeobox like	3	13978756	14124311	145556
TPST1	tyrosylprotein sulfotransferase 1	7	65670186	65885530	215345
TPTE2	transmembrane phosphoinositide 3-phosphatase and tensin homolog 2	13	19997017	20110903	113887
TPX2	TPX2, microtubule nucleation factor	20	30327074	30389608	62535
TRABD2A	TraB domain containing 2A	2	85048774	85134132	85359
TRABD2B	TraB domain containing 2B	1	48226200	48462567	236368
TRAF2	TNF receptor associated factor 2	9	139776364	139821059	44696
TRAF3IP2-AS1	TRAF3IP2 antisense RNA 1	6	111804714	111919505	114792
TRAF4	TNF receptor associated factor 4	17	27071002	27077974	6973
TRAFD1	TRAF-type zinc finger domain containing 1	12	112563305	112591407	28103
TRAIP	TRAF interacting protein	3	49866034	49894007	27974
TRAK1	trafficking kinesin protein 1	3	42055294	42267381	212088
TRAK2	trafficking kinesin protein 2	2	202241930	202316302	74373

TRAM1	translocation associated membrane protein 1	8	71485677	71520622	34946
TRAM2-AS1	TRAM2 antisense RNA 1 (head to head)	6	52442105	52448783	6679
TRAP1	TNF receptor associated protein 1	16	3701640	3767598	65959
TRAPPC11	trafficking protein particle complex 11	4	184580420	184634745	54326
TRAPPC4	trafficking protein particle complex 4	11	118889142	118896164	7023
TRAPPC8	trafficking protein particle complex 8	18	29409136	29533099	123964
TRAPPC9	trafficking protein particle complex 9	8	140742586	141468678	726093
**TRDN**	triadin	6	123537483	123958238	420756
TREM2	triggering receptor expressed on myeloid cells 2	6	41126244	41130924	4681
TRERF1	transcriptional regulating factor 1	6	42192669	42419789	227121
TREX1	three prime repair exonuclease 1	3	48506445	48509044	2600
TRHDE	thyrotropin releasing hormone degrading enzyme	12	72481046	73059422	578377
TRIM11	tripartite motif containing 11	1	228581374	228594541	13168
TRIM16	tripartite motif containing 16	17	15531274	15587625	56352
TRIM2	tripartite motif containing 2	4	154073494	154260472	186979
TRIM24	tripartite motif containing 24	7	138145079	138274738	129660
TRIM25	tripartite motif containing 25	17	54965270	54991399	26130
TRIM33	tripartite motif containing 33	1	114935399	115053781	118383
TRIM37	tripartite motif containing 37	17	57059999	57184282	124284
TRIM5	tripartite motif containing 5	11	5684425	5959849	275425
TRIM55	tripartite motif containing 55	8	67039131	67087720	48590
TRIM66	tripartite motif containing 66	11	8633584	8693413	59830
TRIM9	tripartite motif containing 9	14	51441980	51562779	120800
TRIO	trio Rho guanine nucleotide exchange factor	5	14143811	14532235	388425
TRIP12	thyroid hormone receptor interactor 12	2	230628554	230787955	159402
TRIP13	thyroid hormone receptor interactor 13	5	892758	919472	26715
TRIP4	thyroid hormone receptor interactor 4	15	64679947	64747502	67556
TRIT1	tRNA isopentenyltransferase 1	1	40306723	40349183	42461
TRMT2B	tRNA methyltransferase 2 homolog B	X	100264335	100307105	42771
TRMT44	tRNA methyltransferase 44 homolog (S. cerevisiae)	4	8437867	8495258	57392
TRPC4	transient receptor potential cation channel subfamily C member 4	13	38210773	38444562	233790
TRPC4AP	transient receptor potential cation channel subfamily C member 4 associated protein	20	33590207	33680674	90468
TRPC5	transient receptor potential cation channel subfamily C member 5	X	111017543	111326004	308462
TRPC6	transient receptor potential cation channel subfamily C member 6	11	101322295	101743293	420999
TRPC7	transient receptor potential cation channel subfamily C member 7	5	135548999	135732730	183732
TRPM3	transient receptor potential cation channel subfamily M member 3	9	73143979	74061820	917842
**TRPM7**	transient receptor potential cation channel subfamily M member 7	15	50844670	50979012	134343
TRPM8	transient receptor potential cation channel subfamily M member 8	2	234826043	234928166	102124
TRPS1	transcriptional repressor GATA binding 1	8	116420724	116821899	401176
TRPV4	transient receptor potential cation channel subfamily V member 4	12	110220890	110271212	50323
TSC1	tuberous sclerosis 1	9	135766735	135820020	53286
TSC2	tuberous sclerosis 2	16	2097466	2138716	41251
TSC22D2	TSC22 domain family member 2	3	150126122	150184218	58097
TSC22D3	TSC22 domain family member 3	X	106956451	107020572	64122
TSG101	tumor susceptibility 101	11	18489883	18548779	58897
TSGA10	testis specific 10	2	99613724	99771427	157704
TSHZ1	teashirt zinc finger homeobox 1	18	72922710	73001905	79196
TSHZ2	teashirt zinc finger homeobox 2	20	51588946	52111869	522924
TSHZ3	teashirt zinc finger homeobox 3	19	31765851	31840453	74603
TSIX	TSIX transcript, XIST antisense RNA	X	73012040	73049066	37027
TSNARE1	t-SNARE domain containing 1	8	143293441	143484601	191161
TSNAX-DISC1	TSNAX-DISC1 readthrough (NMD candidate)	1	231664399	231954990	290592
TSPAN18	tetraspanin 18	11	44748015	44953972	205958
TSPAN5	tetraspanin 5	4	99391518	99579780	188263
TSPAN7	tetraspanin 7	X	38420623	38548169	127547
TSPAN9	tetraspanin 9	12	3186521	3395730	209210
TSPEAR	thrombospondin type laminin G domain and EAR repeats	21	45917775	46131495	213721
TSSC1	tumor suppressing subtransferable candidate 1	2	3192696	3381653	188958
TTBK2	tau tubulin kinase 2	15	43030932	43213007	182076
TTC12	tetratricopeptide repeat domain 12	11	113185251	113254266	69016
TTC13	tetratricopeptide repeat domain 13	1	231041989	231114621	72633
**TTC23**	tetratricopeptide repeat domain 23	15	99676528	99791428	114901
TTC26	tetratricopeptide repeat domain 26	7	138818490	138876732	58243
TTC28	tetratricopeptide repeat domain 28	22	28374004	29075853	701850
TTC28-AS1	TTC28 antisense RNA 1	22	28315364	28404569	89206
TTC29	tetratricopeptide repeat domain 29	4	147627790	147867034	239245
TTC37	tetratricopeptide repeat domain 37	5	94799599	94890711	91113
TTC38	tetratricopeptide repeat domain 38	22	46663858	46689905	26048
TTC7B	tetratricopeptide repeat domain 7B	14	91006932	91282823	275892
TTI1	TELO2 interacting protein 1	20	36611409	36661870	50462
TTK	TTK protein kinase	6	80713604	80752244	38641
TTLL11	tubulin tyrosine ligase like 11	9	124584207	124855885	271679
TTLL4	tubulin tyrosine ligase like 4	2	219575568	219620139	44572
TTLL5	tubulin tyrosine ligase like 5	14	76099968	76421421	321454
TTLL6	tubulin tyrosine ligase like 6	17	46839597	46894576	54980
TTLL7	tubulin tyrosine ligase like 7	1	84330711	84464833	134123

TTN	titin	2	179390716	179695529	304814
TTPA	alpha tocopherol transfer protein	8	63961112	63998612	37501
TTR	transthyretin	18	29171689	29178974	7286
TTTY10	testis-specific transcript, Y-linked 10 (non-protein coding)	Y	22627554	22681114	53561
TTTY14	testis-specific transcript, Y-linked 14 (non-protein coding)	Y	21034387	21239302	204916
**TUBA4A**	tubulin alpha 4a	2	220114433	220142892	28460
TUBB3	tubulin beta 3 class III	16	89987800	90005169	17370
TUBB6	tubulin beta 6 class V	18	12307668	12344319	36652
TUBGCP3	tubulin gamma complex associated protein 3	13	113139325	113242481	103157
TUBGCP4	tubulin gamma complex associated protein 4	15	43661419	43699293	37875
TULP4	tubby like protein 4	6	158733692	158932860	199169
TUSC3	tumor suppressor candidate 3	8	15274724	15624158	349435
TWISTNB	TWIST neighbor	7	19735085	19748710	13626
TXK	TXK tyrosine kinase	4	48068410	48136273	67864
TXLNB	taxilin beta	6	139561198	139613276	52079
TXNL1	thioredoxin like 1	18	54264439	54318831	54393
TYR	tyrosinase	11	88910620	89028927	118308
TYW1	tRNA-yW synthesizing protein 1 homolog	7	66460160	66704501	244342
TYW1B	tRNA-yW synthesizing protein 1 homolog B	7	72023729	72298777	275049
U2AF2	U2 small nuclear RNA auxiliary factor 2	19	56165512	56186081	20570
UBA6	ubiquitin like modifier activating enzyme 6	4	68478370	68566897	88528
UBASH3A	ubiquitin associated and SH3 domain containing A	21	43824008	43867791	43784
UBASH3B	ubiquitin associated and SH3 domain containing B	11	122526383	122685181	158799
UBD	ubiquitin D	6	29523292	29527702	4411
UBE2A	ubiquitin conjugating enzyme E2 A	X	118708501	118718381	9881
UBE2B	ubiquitin conjugating enzyme E2 B	5	133706870	133727683	20814
UBE2D1	ubiquitin conjugating enzyme E2 D1	10	60094735	60130513	35779
UBE2E2	ubiquitin conjugating enzyme E2 E2	3	23244511	23633284	388774
UBE2E3	ubiquitin conjugating enzyme E2 E3	2	181831975	181941312	109338
UBE2H	ubiquitin conjugating enzyme E2 H	7	129470572	129592789	122218
**UBE2I**	ubiquitin conjugating enzyme E2 I	16	1355548	1377019	21472
UBE2J2	ubiquitin conjugating enzyme E2 J2	1	1189289	1209265	19977
UBE2L3	ubiquitin conjugating enzyme E2 L3	22	21903736	21978323	74588
UBE2Q2P1	ubiquitin conjugating enzyme E2 Q2 pseudogene 1	15	85070012	85114447	44436
UBE2R2	ubiquitin conjugating enzyme E2 R2	9	33817565	33920402	102838
UBE2V1	ubiquitin conjugating enzyme E2 V1	20	48697661	48732496	34836
UBE2W	ubiquitin conjugating enzyme E2 W	8	74692332	74791145	98814
UBE3A	ubiquitin protein ligase E3A	15	25582381	25684128	101748
UBE3B	ubiquitin protein ligase E3B	12	109915207	109974507	59301
UBE3C	ubiquitin protein ligase E3C	7	156931607	157062066	130460
UBE3D	ubiquitin protein ligase E3D	6	83602117	83775560	173444
**UBE4B**	ubiquitination factor E4B	1	10092890	10241297	148408
UBP1	upstream binding protein 1 (LBP-1a)	3	33429828	33482863	53036
UBQLN1	ubiquilin 1	9	86274878	86323118	48241
**UBQLN2**	ubiquilin 2	X	56590026	56593443	3418
UBQLN4	ubiquilin 4	1	156005092	156023585	18494
UBR1	ubiquitin protein ligase E3 component n-recognin 1	15	43235095	43398311	163217
UBR3	ubiquitin protein ligase E3 component n-recognin 3 (putative)	2	170683968	170940641	256674
UBR4	ubiquitin protein ligase E3 component n-recognin 4	1	19401000	19536770	135771
UBR5	ubiquitin protein ligase E3 component n-recognin 5	8	103265240	103425069	159830
UBXN2B	UBX domain protein 2B	8	59323823	59364060	40238
UBXN7	UBX domain protein 7	3	196074533	196159345	84813
UCA1	urothelial cancer associated 1 (non-protein coding)	19	15939771	15947130	7360
UCHL1	ubiquitin C-terminal hydrolase L1	4	41258430	41270472	12043
UGGT2	UDP-glucose glycoprotein glucosyltransferase 2	13	96453834	96705736	251903
UGT3A1	UDP glycosyltransferase family 3 member A1	5	35951112	36001130	50019
UHRF1BP1L	UHRF1 binding protein 1 like	12	100422233	100536626	114394
UHRF2	ubiquitin like with PHD and ring finger domains 2	9	6413151	6507054	93904
UIMC1	ubiquitin interaction motif containing 1	5	176332006	176449634	117629
ULK2	unc-51 like autophagy activating kinase 2	17	19674142	19771249	97108
ULK4	unc-51 like kinase 4	3	41288090	42003922	715833
**UNC13A**	unc-13 homolog A	19	17712137	17799401	87265
UNC13B	unc-13 homolog B	9	35161999	35405335	243337
UNC13C	unc-13 homolog C	15	54305101	54920806	615706
UNC5C	unc-5 netrin receptor C	4	96083655	96470357	386703
UNC5D	unc-5 netrin receptor D	8	35092975	35654068	561094
UNC79	unc-79 homolog (C. elegans)	14	93799565	94174222	374658
UNC80	unc-80 homolog, NALCN activator	2	210636717	210864024	227308
UNKL	unkempt family like zinc finger	16	1413206	1464752	51547
UPB1	beta-ureidopropionase 1	22	24863206	24924358	61153
UQCC1	ubiquinol-cytochrome c reductase complex assembly factor 1	20	33890369	33999944	109576
URB1	URB1 ribosome biogenesis 1 homolog (S. cerevisiae)	21	33683329	33765335	82007
URB2	URB2 ribosome biogenesis 2 homolog (S. cerevisiae)	1	229761981	229795946	33966
USF1	upstream transcription factor 1	1	161009041	161015767	6727
USF2	upstream transcription factor 2, c-fos interacting	19	35759881	35770724	10844
USH2A	usherin	1	215796236	216596738	800503
USO1	USO1 vesicle transport factor	4	76649777	76735382	85606

USP10	ubiquitin specific peptidase 10	16	84733584	84813528	79945
USP15	ubiquitin specific peptidase 15	12	62654119	62811211	157093
USP20	ubiquitin specific peptidase 20	9	132596977	132644107	47131
USP24	ubiquitin specific peptidase 24	1	55532032	55680786	148755
USP28	ubiquitin specific peptidase 28	11	113668596	113746292	77697
USP31	ubiquitin specific peptidase 31	16	23072727	23160591	87865
USP32	ubiquitin specific peptidase 32	17	58256455	58499831	243377
USP34	ubiquitin specific peptidase 34	2	61414598	61697904	283307
USP40	ubiquitin specific peptidase 40	2	234384166	234475428	91263
USP48	ubiquitin specific peptidase 48	1	22004791	22110099	105309
USP49	ubiquitin specific peptidase 49	6	41757634	41863099	105466
**USP53**	ubiquitin specific peptidase 53	4	120133742	120216672	82931
USP54	ubiquitin specific peptidase 54	10	75257296	75385711	128416
**USP6NL**	USP6 N-terminal like	10	11495945	11653753	157809
UTP15	UTP15, small subunit processome component	5	72861268	72877794	16527
UTP6	UTP6, small subunit processome component	17	30187923	30228784	40862
UTRN	utrophin	6	144606837	145174170	567334
UTY	ubiquitously transcribed tetratricopeptide repeat containing, Y-linked	Y	15360259	15592553	232295
UVRAG	UV radiation resistance associated	11	75526212	75854239	328028
VAC14	Vac14, PIKFYVE complex component	16	70721342	70835064	113723
VAMP1	vesicle associated membrane protein 1	12	6571403	6580153	8751
VAMP4	vesicle associated membrane protein 4	1	171669300	171711387	42088
VAPA	VAMP associated protein A	18	9913999	9960018	46020
**VAPB**	VAMP associated protein B and C	20	56964178	57026157	61980
VAV1	vav guanine nucleotide exchange factor 1	19	6772725	6857377	84653
VAV3	vav guanine nucleotide exchange factor 3	1	108113782	108507766	393985
VBP1	VHL binding protein 1	X	154425284	154468098	42815
VCAN	versican	5	82767284	82878122	110839
**VCP**	valosin containing protein	9	35056061	35073246	17186
VCPIP1	valosin containing protein interacting protein 1	8	67540722	67579452	38731
VCPKMT	valosin containing protein lysine methyltransferase	14	50575350	50583318	7969
VDAC1	voltage dependent anion channel 1	5	133307606	133340824	33219
**VDR**	vitamin D (1,25- dihydroxyvitamin D3) receptor	12	48235320	48336831	101512
**VEGFA**	vascular endothelial growth factor A	6	43737921	43754224	16304
VEPH1	ventricular zone expressed PH domain containing 1	3	156977531	157251408	273878
VGLL4	vestigial like family member 4	3	11597544	11762220	164677
VIL1	villin 1	2	219283815	219318018	34204
VKORC1L1	vitamin K epoxide reductase complex subunit 1 like 1	7	65338254	65424550	86297
VLDLR	very low density lipoprotein receptor	9	2621834	2660053	38220
VMP1	vacuole membrane protein 1	17	57784553	57919616	135064
VOPP1	vesicular, overexpressed in cancer, prosurvival protein 1	7	55503749	55640681	136933
VPS13A	vacuolar protein sorting 13 homolog A	9	79792269	80036457	244189
VPS13A-AS1	VPS13A antisense RNA 1	9	79791672	79792910	1239
VPS13B	vacuolar protein sorting 13 homolog B	8	100025494	100889808	864315
VPS13C	vacuolar protein sorting 13 homolog C	15	62144588	62352672	208085
VPS13D	vacuolar protein sorting 13 homolog D	1	12290124	12572099	281976
VPS35	VPS35, retromer complex component	16	46690054	46723430	33377
VPS37A	VPS37A, ESCRT-I subunit	8	17104080	17159936	55857
VPS41	VPS41, HOPS complex subunit	7	38762563	38971994	209432
VPS4B	vacuolar protein sorting 4 homolog B	18	61056423	61089716	33294
VPS53	VPS53, GARP complex subunit	17	411908	624957	213050
**VPS54**	VPS54, GARP complex subunit	2	64119280	64246206	126927
VPS8	VPS8, CORVET complex subunit	3	184529931	184770402	240472
VSTM4	V-set and transmembrane domain containing 4	10	50222290	50323554	101265
**VTCN1**	V-set domain containing T-cell activation inhibitor 1	1	117686209	117753556	67348
VTI1A	vesicle transport through interaction with t-SNAREs 1A	10	114206756	114578503	371748
VWA3B	von Willebrand factor A domain containing 3B	2	98703579	98929762	226184
VWA5B1	von Willebrand factor A domain containing 5B1	1	20617412	20681387	63976
VWC2L	von Willebrand factor C domain containing protein 2 like	2	215275789	215443683	167895
VWF	von Willebrand factor	12	6058040	6233936	175897
WAC	WW domain containing adaptor with coiled-coil	10	28821422	28912041	90620
WARS	tryptophanyl-tRNA synthetase	14	100800125	100843142	43018
WARS2	tryptophanyl tRNA synthetase 2, mitochondrial	1	119573839	119683294	109456
WASF1	WAS protein family member 1	6	110421022	110501207	80186
WASF2	WAS protein family member 2	1	27730730	27816669	85940
WASF3	WAS protein family member 3	13	27131840	27263085	131246
WBSCR17	Williams-Beuren syndrome chromosome region 17	7	70597155	71178585	581431
WDFY2	WD repeat and FYVE domain containing 2	13	52158644	52336171	177528
WDFY3	WD repeat and FYVE domain containing 3	4	85590704	85887544	296841
WDFY4	WDFY family member 4	10	49892921	50191001	298081
WDPCP	WD repeat containing planar cell polarity effector	2	63348518	64054977	706460
WDR13	WD repeat domain 13	X	48448430	48463581	15152
WDR27	WD repeat domain 27	6	169857307	170102159	244853
WDR33	WD repeat domain 33	2	128458596	128568761	110166
WDR43	WD repeat domain 43	2	29117509	29171088	53580
WDR44	WD repeat domain 44	X	117480036	117583924	103889
WDR45	WD repeat domain 45	X	48929385	48958108	28724

WDR53	WD repeat domain 53	3	196281056	196295545	14490
WDR60	WD repeat domain 60	7	158649269	158749438	100170
WDR62	WD repeat domain 62	19	36545783	36596008	50226
WDR64	WD repeat domain 64	1	241815580	241965435	149856
WDR7	WD repeat domain 7	18	54318574	54698828	380255
WDR70	WD repeat domain 70	5	37379314	37753537	374224
WDR72	WD repeat domain 72	15	53805938	54055075	249138
WDR82	WD repeat domain 82	3	52288437	52322036	33600
WDR93	WD repeat domain 93	15	90234028	90286869	52842
WDTC1	WD and tetratricopeptide repeats 1	1	27561007	27635110	74104
WDYHV1	WDYHV motif containing 1	8	124428965	124479470	50506
WFDC1	WAP four-disulfide core domain 1	16	84328252	84363450	35199
WFS1	wolframin ER transmembrane glycoprotein	4	6271576	6304992	33417
WHAMMP2	WAS protein homolog associated with actin, golgi membranes and microtubules pseudogene 2	15	28982729	29002537	19809
WIF1	WNT inhibitory factor 1	12	65444406	65515346	70941
WIPF3	WAS/WASL interacting protein family member 3	7	29846102	29956682	110581
WIPI2	WD repeat domain, phosphoinositide interacting 2	7	5229819	5273457	43639
WLS	wntless Wnt ligand secretion mediator	1	68564142	68698803	134662
WNK1	WNK lysine deficient protein kinase 1	12	861759	1020618	158860
WNK2	WNK lysine deficient protein kinase 2	9	95947198	96082854	135657
WNT11	Wnt family member 11	11	75897369	75921780	24412
WNT7B	Wnt family member 7B	22	46316242	46373009	56768
WNT8B	Wnt family member 8B	10	102222798	102243501	20704
WT1-AS	WT1 antisense RNA	11	32457064	32480315	23252
WTAPP1	Wilms tumor 1 associated protein pseudogene 1	11	102617699	102707497	89799
WWC1	WW and C2 domain containing 1	5	167718656	167899308	180653
WWC3	WWC family member 3	X	9983602	10112518	128917
WWOX	WW domain containing oxidoreductase	16	78133310	79246564	1113255
XDH	xanthine dehydrogenase	2	31557187	31637581	80395
XG	Xg blood group	X	2670091	2734539	64449
XIRP2	xin actin binding repeat containing 2	2	167744997	168116263	371267
XKR4	XK related 4	8	56014949	56454613	439665
XKR6	XK related 6	8	10753555	11058875	305321
XPA	XPA, DNA damage recognition and repair factor	9	100437191	100459639	22449
XPC	XPC complex subunit, DNA damage recognition and repair factor	3	14186647	14220283	33637
XPNPEP2	X-prolyl aminopeptidase 2	X	128872950	128903514	30565
XPNPEP3	X-prolyl aminopeptidase 3	22	41253081	41363838	110758
XPO5	exportin 5	6	43490072	43543812	53741
XPO6	exportin 6	16	28109300	28223241	113942
XPO7	exportin 7	8	21777180	21864096	86917
XPR1	xenotropic and polytropic retrovirus receptor 1	1	180601140	180859387	258248
XRCC1	X-ray repair cross complementing 1	19	44047192	44084625	37434
XRCC3	X-ray repair cross complementing 3	14	104163946	104181841	17896
XRCC5	X-ray repair cross complementing 5	2	216972187	217071026	98840
XRCC6P5	X-ray repair cross complementing 6 pseudogene 5	X	98716600	98976156	259557
XRN1	5'-3' exoribonuclease 1	3	142025449	142166904	141456
XXYLT1	xyloside xylosyltransferase 1	3	194789008	194991896	202889
XYLT1	xylosyltransferase 1	16	17195626	17564738	369113
YAF2	YY1 associated factor 2	12	42550906	42632151	81246
YARS	tyrosyl-tRNA synthetase	1	33240840	33283754	42915
YES1	YES proto-oncogene 1, Src family tyrosine kinase	18	721588	812547	90960
YIPF7	Yip1 domain family member 7	4	44624086	44680573	56488
YME1L1	YME1 like 1 ATPase	10	27399383	27444195	44813
YTHDC1	YTH domain containing 1	4	69176105	69215807	39703
YTHDF1	YTH N6-methyladenosine RNA binding protein 1	20	61826781	61847586	20806
YWHAG	tyrosine 3-monooxygenase/tryptophan 5-monooxygenase activation protein gamma	7	75956116	75988348	32233
YWHAH	tyrosine 3-monooxygenase/tryptophan 5-monooxygenase activation protein eta	22	32340447	32353590	13144
ZBBX	zinc finger B-box domain containing	3	166958075	167099134	141060
ZBTB16	zinc finger and BTB domain containing 16	11	113930315	114121398	191084
ZBTB20	zinc finger and BTB domain containing 20	3	114056941	114866118	809178
ZBTB26	zinc finger and BTB domain containing 26	9	125677845	125693779	15935
ZBTB37	zinc finger and BTB domain containing 37	1	173837220	173872687	35468
ZBTB41	zinc finger and BTB domain containing 41	1	197122810	197169672	46863
ZBTB44	zinc finger and BTB domain containing 44	11	130096572	130184581	88010
ZBTB46	zinc finger and BTB domain containing 46	20	62375019	62462597	87579
ZBTB7C	zinc finger and BTB domain containing 7C	18	45553044	45937123	384080
ZBTB8A	zinc finger and BTB domain containing 8A	1	33005028	33071540	66513
ZBTB8B	zinc finger and BTB domain containing 8B	1	32930670	32962287	31618
ZC2HC1C	zinc finger C2HC-type containing 1C	14	75530873	75545126	14254
ZC3H12D	zinc finger CCCH-type containing 12D	6	149768794	149806197	37404
ZC3H13	zinc finger CCCH-type containing 13	13	46528600	46626894	98295
ZC3H18	zinc finger CCCH-type containing 18	16	88636789	88698374	61586
ZC3H7A	zinc finger CCCH-type containing 7A	16	11844442	11891123	46682
ZCCHC11	zinc finger CCHC-type containing 11	1	52873954	53019159	145206

ZCCHC14	zinc finger CCHC-type containing 14	16	87439852	87525651	85800
ZCCHC24	zinc finger CCHC-type containing 24	10	81142081	81205383	63303
ZCCHC4	zinc finger CCHC-type containing 4	4	25314407	25372005	57599
ZCCHC7	zinc finger CCHC-type containing 7	9	37120536	37358146	237611
ZCWPW1	zinc finger CW-type and PWWP domain containing 1	7	99998449	100026615	28167
ZCWPW2	zinc finger CW-type and PWWP domain containing 2	3	28390637	28579613	188977
ZDHHC1	zinc finger DHHC-type containing 1	16	67428322	67450736	22415
ZDHHC13	zinc finger DHHC-type containing 13	11	19138646	19197969	59324
ZDHHC14	zinc finger DHHC-type containing 14	6	157802165	158099178	297014
ZDHHC15	zinc finger DHHC-type containing 15	X	74588262	74743337	155076
ZDHHC17	zinc finger DHHC-type containing 17	12	77157368	77247476	90109
ZDHHC5	zinc finger DHHC-type containing 5	11	57435219	57468659	33441
ZEB1	zinc finger E-box binding homeobox 1	10	31607424	31818742	211319
ZEB2	zinc finger E-box binding homeobox 2	2	145141648	145282147	140500
ZFAND2A	zinc finger AN1-type containing 2A	7	1191707	1200395	8689
ZFAND3	zinc finger AN1-type containing 3	6	37787275	38122400	335126
ZFAND5	zinc finger AN1-type containing 5	9	74966341	74980163	13823
ZFAND6	zinc finger AN1-type containing 6	15	80351910	80430735	78826
ZFAT	zinc finger and AT-hook domain containing	8	135490031	135725292	235262
ZFHX3	zinc finger homeobox 3	16	72816784	73093597	276814
**ZFP14**	ZFP14 zinc finger protein	19	36827162	36870101	42940
ZFP3	ZFP3 zinc finger protein	17	4981543	4999669	18127
**ZFP64**	ZFP64 zinc finger protein	20	50668202	50820847	152646
ZFP90	ZFP90 zinc finger protein	16	68563993	68609975	45983
ZFP91-CNTF	ZFP91-CNTF readthrough (NMD candidate)	11	58346645	58392112	45468
ZFPM2	zinc finger protein, FOG family member 2	8	106330920	106816760	485841
ZFR	zinc finger RNA binding protein	5	32354456	32444867	90412
ZFYVE26	zinc finger FYVE-type containing 26	14	68194091	68283307	89217
ZFYVE27	zinc finger FYVE-type containing 27	10	99496878	99520664	23787
ZFYVE28	zinc finger FYVE-type containing 28	4	2271309	2420390	149082
ZHX3	zinc fingers and homeoboxes 3	20	39807088	39946312	139225
ZMAT1	zinc finger matrin-type 1	X	101137262	101187004	49743
ZMAT4	zinc finger matrin-type 4	8	40388109	40755352	367244
ZMYM6	zinc finger MYM-type containing 6	1	35449523	35497569	48047
ZNF10	zinc finger protein 10	12	133707161	133736051	28891
ZNF106	zinc finger protein 106	15	42705021	42783321	78301
ZNF12	zinc finger protein 12	7	6728064	6746554	18491
ZNF135	zinc finger protein 135	19	58570607	58597677	27071
ZNF14	zinc finger protein 14	19	19821280	19843906	22627
ZNF143	zinc finger protein 143	11	9481866	9550071	68206
ZNF148	zinc finger protein 148	3	124944405	125094198	149794
ZNF180	zinc finger protein 180	19	44979854	45004576	24723
ZNF182	zinc finger protein 182	X	47834250	47863377	29128
ZNF19	zinc finger protein 19	16	71498453	71598992	100540
ZNF200	zinc finger protein 200	16	3272325	3286221	13897
ZNF223	zinc finger protein 223	19	44555520	44572144	16625
ZNF236	zinc finger protein 236	18	74534563	74682683	148121
ZNF248	zinc finger protein 248	10	38091751	38147034	55284
ZNF25	zinc finger protein 25	10	38238500	38265561	27062
ZNF251	zinc finger protein 251	8	145946298	145981802	35505
ZNF280C	zinc finger protein 280C	X	129336685	129402873	66189
ZNF286B	zinc finger protein 286B	17	18561742	18585575	23834
ZNF287	zinc finger protein 287	17	16454701	16472520	17820
ZNF292	zinc finger protein 292	6	87862551	87973914	111364
ZNF318	zinc finger protein 318	6	43274872	43337216	62345
ZNF35	zinc finger protein 35	3	44690219	44702283	12065
ZNF385B	zinc finger protein 385B	2	180306709	180726232	419524
ZNF385D	zinc finger protein 385D	3	21459915	22414812	954898
ZNF407	zinc finger protein 407	18	72265106	72777627	512522
ZNF41	zinc finger protein 41	X	47305278	47342345	37068
ZNF423	zinc finger protein 423	16	49521435	49891830	370396
ZNF438	zinc finger protein 438	10	31109136	31320866	211731
ZNF490	zinc finger protein 490	19	12688775	12750912	62138
ZNF503	zinc finger protein 503	10	77157588	77161664	4077
ZNF507	zinc finger protein 507	19	32836500	32878573	42074
**ZNF512B**	zinc finger protein 512B	20	62588055	62680113	92059
ZNF518B	zinc finger protein 518B	4	10441498	10459034	17537
ZNF521	zinc finger protein 521	18	22641890	22932154	290265
ZNF534	zinc finger protein 534	19	52932440	52955568	23129
ZNF536	zinc finger protein 536	19	30719197	31204445	485249
ZNF541	zinc finger protein 541	19	48023942	48059113	35172
**ZNF556**	zinc finger protein 556	19	2867333	2878515	11183
ZNF560	zinc finger protein 560	19	9577183	9609283	32101
ZNF567	zinc finger protein 567	19	37178514	37218603	40090
**ZNF578**	zinc finger protein 578	19	52956829	53015407	58579
ZNF585A	zinc finger protein 585A	19	37597636	37663643	66008
ZNF596	zinc finger protein 596	8	182137	197342	15206

ZNF597	zinc finger protein 597	16	3486104	3493542	7439
ZNF622	zinc finger protein 622	5	16451628	16465901	14274
ZNF638	zinc finger protein 638	2	71503691	71662199	158509
ZNF644	zinc finger protein 644	1	91380859	91487829	106971
ZNF652	zinc finger protein 652	17	47366568	47439835	73268
ZNF677	zinc finger protein 677	19	53731577	53758151	26575
ZNF678	zinc finger protein 678	1	227751244	227865144	113901
**ZNF682**	zinc finger protein 682	19	20107867	20150315	42449
ZNF691	zinc finger protein 691	1	43312280	43318148	5869
ZNF697	zinc finger protein 697	1	120162045	120190396	28352
ZNF704	zinc finger protein 704	8	81540686	81787016	246331
ZNF710	zinc finger protein 710	15	90544624	90625438	80815
ZNF718	zinc finger protein 718	4	124386	157779	33394
ZNF721	zinc finger protein 721	4	419604	492945	73342
**ZNF746**	zinc finger protein 746	7	149169885	149194908	25024
ZNF75D	zinc finger protein 75D	X	134382867	134478012	95146
ZNF786	zinc finger protein 786	7	148766735	148787874	21140
ZNF800	zinc finger protein 800	7	126986844	127071978	85135
ZNF804A	zinc finger protein 804A	2	185463093	185804219	341127
ZNF804B	zinc finger protein 804B	7	88388682	88966346	577665
ZNF815P	zinc finger protein 815, pseudogene	7	5862791	5894066	31276
ZNF827	zinc finger protein 827	4	146678779	146859787	181009
ZNF843	zinc finger protein 843	16	31445569	31454346	8778
ZNF862	zinc finger protein 862	7	149535456	149564568	29113
ZNF99	zinc finger protein 99	19	22939007	22966909	27903
ZNHIT6	zinc finger HIT-type containing 6	1	86115106	86174116	59011
ZNRF3	zinc and ring finger 3	22	29279580	29453475	173896
ZP1	zona pellucida glycoprotein 1	11	60635035	60643166	8132
ZRANB3	zinc finger RANBP2-type containing 3	2	135894486	136288806	394321
ZSWIM2	zinc finger SWIM-type containing 2	2	187692562	187713935	21374
**ZSWIM7**	zinc finger SWIM-type containing 7	17	15879874	15903031	23158
**ZUFSP**	zinc finger with UFM1 specific peptidase domain	6	116956781	116989957	33177
ZWILCH	zwilch kinetochore protein	15	66797297	66842115	44819
ZWINT	ZW10 interacting kinetochore protein	10	58116989	58121036	4048
ZYG11B	zyg-11 family member B, cell cycle regulator	1	53192126	53293014	100889

Supplementary Table 3. Comparison of the data obtained for 5 selected genes by *NeuroArray* aCGH analysis and Real-Time RT-PCR

Gene ID	SALS1		SALS2		*NeuroArray* probe ID	Forward primer (5'-3')	Reverse primer (5'-3')
	aCGH (Median LogRatio)	RT-PCR (ddCt method)	aCGH (Median LogRatio)	RT-PCR (ddCt method)			
**GAA**	0.50	1.52	-	-	A_16_P34263128	GCGTGCGGGTTGTTCTCT	CCACGCTGTAGAGTGGGGAC
**KIF1A**	0.71	2.22	0.73	3.26	A_16_P00630878	CAACAGAGACAGACAAGGAGCC	TGTCTCCCCAAGTATGAGGGTC
**CSF1**	-	-	0.92	1.30	A_16_P30247855	GTAGACCAGGAACAGTTGGTGA	ACCCATGGATCCTCACACTCT
**TRAF2**	-	-	0.78	1.66	A_16_P32584215	AGATCTCAGACTTCGCCAGGA	TTGGGAGCCATCCTATGTGGT
**HSPA5**	-1.21	-2.02	-1.17	-1.63	A_16_P32536366	CAAGCAACCAAAGACGCTGG	GCTGCCGTACTATTAGATTGAAAAA

Supplementary Table 4. Chromosomal regions with amplifications and deletions present in > 10% of SALS patients.

Duplications

Chromosome	Start	Stop	Aberration Size (bps)	Frequency (%)
14	31552632	31552690	59	76.666664
17	17716576	17720711	4136	70
X	122318451	122320113	1663	60
17	17720711	17726812	6102	56.666668
X	122318291	122318451	161	56.666668
X	122320113	122336599	16487	56.666668
X	122318031	122318291	261	53.333332
9	131388073	131394672	6600	50
7	100493729	100493862	134	46.666668
17	17726812	17726864	53	46.666668
22	24376158	24384300	8143	46.666668
9	129376726	129455813	79088	43.333332
19	50766570	50795492	28923	43.333332
X	122336599	122459975	123377	43.333332
2	241657356	241728764	71409	40
9	129272014	129376726	104713	40
9	129455813	129458220	2408	40
2	127807997	128431400	623404	36.666668
7	100488043	100490289	2247	36.666668
7	100493373	100493729	357	36.666668
17	17715816	17716576	761	36.666668
2	128431400	128439169	7770	33.333332
7	100490289	100493373	3085	33.333332
10	99511112	99519055	7944	33.333332
19	50795492	50795529	38	33.333332
5	176857805	176869527	11723	30
9	131394672	131395293	622	30
10	99504592	99511112	6521	30
17	17726864	17740072	13209	30
19	50795529	50796566	1038	30
20	23614298	23618518	4221	30
X	153127628	153602907	475280	30
1	55331123	55527185	196063	26.666666
1	208202135	208257698	55564	26.666666
2	127806098	127807997	1900	26.666666
5	37834861	37834984	124	26.666666
9	129191716	129272014	80299	26.666666
9	129458220	129458296	77	26.666666
15	89381841	90198653	816813	26.666666
17	56350271	56357941	7671	26.666666
20	62037795	62599159	561365	26.666666
X	122459975	122460062	88	26.666666
1	2337815	6557050	4219236	23.333334
1	165218854	165377453	158600	23.333334
1	205012286	205041558	29273	23.333334
1	208201489	208202135	647	23.333334
5	176853852	176857805	3954	23.333334
14	105238845	105259939	21095	23.333334
16	28488776	28502793	14018	23.333334
17	77086157	77097800	11644	23.333334
19	50760516	50766570	6055	23.333334
20	23618518	23618656	139	23.333334
20	61992392	62037795	45404	23.333334
Y	4114337	8158564	4044228	23.333334
1	16348551	22216964	5868414	20
1	165175298	165218854	43557	20
1	165377453	165377539	87	20
2	128439169	128439407	239	20
2	241656421	241657356	936	20
5	37834984	37835437	454	20
6	31919858	32148778	228921	20
7	4823028	4830775	7748	20
9	87283500	87284663	1164	20
11	17415944	17450206	34263	20
15	89346786	89381841	35056	20
16	28502793	28503629	837	20
17	77097800	77099247	1448	20
19	50364748	50760516	395769	20
19	50796566	50836689	40124	20
20	1963661	2384209	420549	20
Y	18149636	21882048	3732413	20
1	22216964	22222489	5526	16.666666
1	165377539	165378926	1388	16.666666
1	205041558	205041736	179	16.666666
1	208257698	208257977	280	16.666666
2	20818975	20850933	31959	16.666666
2	241728764	241737212	8449	16.666666
4	102268216	102315609	47394	16.666666
5	137777186	137781394	4209	16.666666
5	168179999	168180168	170	16.666666

Deletions

Chromosome	Start	Stop	Aberration Size (bps)	Frequency (%)
20	33986975	35569474	1582500	80
1	47767175	47770585	3411	76.666664
20	35569474	35575306	5833	76.666664
3	1.56E+08	1.56E+08	8949	73.333336
1	47716828	47767175	50348	70
1	47770585	47770755	171	70
3	1.56E+08	1.56E+08	3669	70
3	1.56E+08	1.56E+08	58	70
1	47435653	47716828	281176	63.333332
1	47770755	47775972	5218	63.333332
2	32339724	32409410	69687	63.333332
2	32323849	32339724	15876	60
2	32409410	32429834	20425	60
9	1.28E+08	1.28E+08	607	60
2	32314495	32323849	9355	56.666668
2	32429834	32432100	2267	56.666668
3	1.56E+08	1.56E+08	103	56.666668
9	1.28E+08	1.28E+08	122189	56.666668
12	1.12E+08	1.12E+08	34973	56.666668
1	46685884	47435653	749770	53.333332
1	47775972	47776133	162	53.333332
3	1.56E+08	1.56E+08	101	53.333332
5	69372472	70247951	875480	53.333332
10	1.21E+08	1.21E+08	421	53.333332
12	1.12E+08	1.12E+08	2539	53.333332
14	92527804	92560197	32394	53.333332
2	32312639	32314495	1857	50
5	69372317	69372472	156	50
10	1.21E+08	1.21E+08	111447	50
12	1.12E+08	1.12E+08	38	50
16	46697028	46703042	6015	50
2	32312627	32312639	13	46.666668
2	32432100	32434737	2638	46.666668
14	92525408	92527804	2397	46.666668
14	92560197	92562372	2176	46.666668
16	46696885	46697028	144	46.666668
17	2541457	2569364	27908	46.666668
18	9117815	9134199	16385	46.666668
22	32156586	32188108	31523	46.666668
15	50924954	50955264	30311	43.333332
16	46695793	46696885	1093	43.333332
17	2569364	2569504	141	43.333332
22	32154622	32156586	1965	43.333332
22	32188108	32200902	12795	43.333332
2	64143925	64169715	25791	40
3	1.56E+08	1.56E+08	2765	40
15	50911927	50924954	13028	40
15	50955264	50975081	19818	40
16	46694461	46695793	1333	40
17	2541347	2541457	111	40
18	9117738	9117815	78	40
22	32154613	32154622	10	40
22	32200902	32205658	4757	40
2	64169715	64169735	21	36.666668
2	64169735	64193249	23515	36.666668
6	32486277	32489896	3620	36.666668
12	1.12E+08	1.12E+08	156	36.666668
14	92413787	92525408	111622	36.666668
16	46703042	46712898	9857	36.666668
22	32205658	32206586	929	36.666668
2	64124630	64143925	19296	33.333332
2	64193249	64199380	6132	33.333332
5	70247951	70307077	59127	33.333332
5	70307077	70307124	48	33.333332
6	32485872	32486277	406	33.333332
6	32489896	32489935	40	33.333332
15	21200700	22432687	1231988	33.333332
17	2569504	2580007	10504	33.333332
1	63876791	63885210	8420	30
2	64119802	64124630	4829	30
5	70307124	70309855	2732	30
10	70432579	70441196	8618	30
12	1.12E+08	1.12E+08	29476	30
15	22432687	23044610	611924	30
15	50872974	50911927	38954	30
16	46712898	46713139	242	30
17	2580007	4605227	2025221	30
17	19753054	19769087	16034	30
20	33834793	33986975	152183	30
22	32206586	32206711	126	30

6	32485851	32485872	22	16.666666
6	32485872	32486277	406	16.666666
6	32486277	32489896	3620	16.666666
6	32489896	32489935	40	16.666666
7	2281893	4823028	2541136	16.666666
8	144635580	145024441	388862	16.666666
9	87284663	87285883	1221	16.666666
9	138594020	138641871	47852	16.666666
9	138660447	138661983	1537	16.666666
11	2161421	2161583	163	16.666666
11	17408294	17415944	7651	16.666666
11	17450206	17464859	14654	16.666666
12	57587270	57605854	18585	16.666666
15	42678271	42695967	17697	16.666666
16	57685031	57698265	13235	16.666666
17	17740072	17740236	165	16.666666
17	75369546	77086157	1716612	16.666666
17	77099247	78092622	993376	16.666666
19	50364607	50364748	142	16.666666
20	2384209	2411082	26874	16.666666
22	37622611	37640194	17584	16.666666
Y	8158564	8694578	536015	16.666666
Y	17890214	18149636	259423	16.666666
Y	21882048	22086267	204220	16.666666
1	22222489	22222904	416	13.333333
1	208257977	208276459	18483	13.333333
1	227075891	227173012	97122	13.333333
4	41700207	41748255	48049	13.333333
4	102315609	103422921	1107313	13.333333
5	37824072	37834861	10790	13.333333
5	137776953	137777186	234	13.333333
5	137781394	137781792	399	13.333333
5	168233667	168620579	386913	13.333333
6	32148778	32149461	684	13.333333
6	32166526	32191834	25309	13.333333
6	32485173	32485851	679	13.333333
6	32489935	32489967	33	13.333333
7	2281791	2281893	103	13.333333
8	42037953	42065260	27308	13.333333
8	145024441	145049522	25082	13.333333
9	136390729	138594020	2203292	13.333333
9	138641871	138660447	18577	13.333333
9	138661983	140062358	1400376	13.333333
11	792284	2161421	1369138	13.333333
11	2161583	4056596	1895014	13.333333
11	4056596	7324468	3267873	13.333333
11	17464859	17470241	5383	13.333333
11	45906997	45907487	491	13.333333
12	125348132	125348422	291	13.333333
14	99641841	100193575	551735	13.333333
15	42695967	42703427	7461	13.333333
15	50978890	51201129	222240	13.333333
16	1250785	1359337	108553	13.333333
16	10273981	10276571	2591	13.333333
16	57653630	57685031	31402	13.333333
17	8791519	8794231	2713	13.333333
17	75277604	75369546	91943	13.333333
20	60718894	60718952	59	13.333333
20	61987803	61992392	4590	13.333333
21	44495820	44496400	581	13.333333
22	24384300	24828385	444086	13.333333
22	37621845	37622611	767	13.333333
Y	8694578	9072145	377568	13.333333
Y	17493425	17890214	396790	13.333333
Y	22086267	23799790	1713524	13.333333
1	1956561	2337815	381255	10
1	22222904	22379326	156423	10
1	55527185	55529886	2702	10
1	65854982	65855380	399	10
1	110280992	110467801	186810	10
1	156060447	156851294	790848	10
1	165175086	165175298	213	10
1	165378926	165406335	27410	10
1	204124931	205012286	887356	10
1	208276459	208417579	141121	10
1	227058314	227075891	17578	10
2	20818280	20818975	696	10
2	74589902	74590293	392	10
2	74596602	74603884	7283	10
2	127805579	127806098	520	10
2	152954807	152955141	335	10
2	171673289	171687463	14175	10
3	128202684	128212098	9415	10
4	41216529	41259802	43274	10
4	102100184	102268216	168033	10
4	184580356	184580630	275	10

1	63836485	63876791	40307	26.666666
1	63885210	63902525	17316	26.666666
1	2.47E+08	2.47E+08	140	26.666666
5	70309855	70320678	10824	26.666666
6	32485851	32485872	22	26.666666
7	22771002	23017142	246141	26.666666
10	76154068	76768859	614792	26.666666
14	1.04E+08	1.04E+08	62601	26.666666
15	20575646	21200700	625055	26.666666
15	44912321	44951263	38943	26.666666
17	19752773	19753054	282	26.666666
17	19769087	19769218	132	26.666666
17	30212981	30226829	13849	26.666666
1	63836432	63836485	54	23.333334
1	63902525	63903304	780	23.333334
1	2.08E+08	2.08E+08	19860	23.333334
5	70320678	71554990	1234313	23.333334
6	32485173	32485851	679	23.333334
7	23017142	23017909	768	23.333334
9	1.35E+08	1.35E+08	33833	23.333334
10	76099253	76154068	54816	23.333334
11	85714533	85737381	22849	23.333334
14	1.04E+08	1.04E+08	27229	23.333334
14	1.04E+08	1.04E+08	178091	23.333334
15	23044610	23046598	1989	23.333334
15	44900781	44912321	11541	23.333334
15	50849380	50872974	23595	23.333334
16	46693906	46694461	556	23.333334
17	19741803	19752773	10971	23.333334
17	29110052	29114424	4373	23.333334
17	30190437	30212981	22545	23.333334
17	30226829	30228745	1917	23.333334
17	30228745	30326197	97453	23.333334
18	9134199	9134357	159	23.333334
1	47776133	47778637	2505	20
1	2.08E+08	2.08E+08	2109	20
1	2.47E+08	2.47E+08	6692	20
6	31783348	31797880	14533	20
6	32412763	32485173	72411	20
6	74303319	74351597	48279	20
9	1.08E+08	1.08E+08	289	20
9	1.27E+08	1.28E+08	1188578	20
9	1.35E+08	1.35E+08	7254	20
10	75960468	76099253	138786	20
11	77814989	77815111	123	20
11	85714505	85714533	29	20
12	32778695	32786500	7806	20
12	1.12E+08	1.12E+08	2013	20
15	23046598	23048496	1899	20
15	44951263	44952618	1356	20
15	93487795	93489253	1459	20
17	19729339	19741803	12465	20
17	29114424	29123314	8891	20
17	30190276	30190437	162	20
17	30326197	30327458	1262	20
17	44302758	44302802	45	20
1	44173154	44201940	28787	16.666666
1	1.74E+08	1.74E+08	29292	16.666666
1	2.47E+08	2.47E+08	3431	16.666666
1	2.47E+08	2.47E+08	5171	16.666666
2	32434737	32610141	175405	16.666666
2	2.22E+08	2.22E+08	77	16.666666
5	61645975	61657411	11437	16.666666
5	69288477	69372317	83841	16.666666
6	31797880	31865577	67698	16.666666
6	32489935	32489967	33	16.666666
6	74351597	74354308	2712	16.666666
8	94777910	94793916	16007	16.666666
9	1.08E+08	1.08E+08	15844	16.666666
9	1.26E+08	1.27E+08	742981	16.666666
9	1.28E+08	1.28E+08	128	16.666666
9	1.35E+08	1.35E+08	2167	16.666666
9	1.35E+08	1.35E+08	1452	16.666666
10	94373202	94405187	31986	16.666666
10	96540191	96580305	40115	16.666666
11	77814020	77814989	970	16.666666
12	32751551	32778695	27145	16.666666
14	50597479	50666488	69010	16.666666
15	23048496	23052587	4092	16.666666
15	41535920	41562793	26874	16.666666
15	44898259	44900781	2523	16.666666
15	50975081	50978890	3810	16.666666
15	50978890	51285562	306673	16.666666
15	64226370	65275796	1049427	16.666666
15	78732085	78770736	38652	16.666666
15	93487634	93487795	162	16.666666

5	37835437	37835564	128	10
5	121412746	121647970	235225	10
5	167719205	168179999	460795	10
5	168180168	168233667	53500	10
5	168620579	168678536	57958	10
6	29910241	29910772	532	10
6	32149461	32166526	17066	10
6	32191834	32191906	73	10
7	100487735	100488043	309	10
8	145049522	145050879	1358	10
9	35068399	35072760	4362	10
9	87283404	87283500	97	10
9	131395293	131395544	252	10
9	135810367	136390729	580363	10
11	424565	792284	367720	10
11	7324468	7324653	186	10
11	45907487	45925785	18299	10
11	117263667	117265845	2179	10
12	57549817	57587270	37454	10
12	57605854	57606775	922	10
13	51483764	51484234	471	10
15	27017550	27018935	1386	10
15	42703427	42703603	177	10
16	1203457	1245002	41546	10
16	1359337	2147716	788380	10
16	7703763	7715070	11308	10
16	10273832	10273981	150	10
16	57698265	57698622	358	10
16	74808415	74808756	342	10
17	1648642	1658070	9429	10
17	8790922	8791519	598	10
17	17715423	17715816	394	10
17	17740236	17872902	132667	10
17	26698927	26711380	12454	10
17	34198604	34417495	218892	10
17	56349278	56350271	994	10
17	56357941	56358113	173	10
19	1000387	1549191	548805	10
19	50836689	50885869	49181	10
20	1963574	1963661	88	10
20	2411082	2636794	225713	10
20	3869789	3870396	608	10
20	60718952	61987803	1268852	10
21	43693384	44495820	802437	10
21	44496400	46931240	2434841	10
22	18907140	18918487	11348	10
22	42486755	42526821	40067	10
X	47433998	53321220	5887223	10
X	62875495	63005325	129831	10
X	153602907	153911715	308809	10
Y	23799790	24108431	308642	10

17	29123314	29123434	121	16.666666
17	29663929	29667773	3845	16.666666
17	44270188	44270247	60	16.666666
17	44270247	44302758	32512	16.666666
17	61560736	61560846	111	16.666666
22	32206711	32215105	8395	16.666666
1	1.74E+08	1.74E+08	38	13.333333
1	1.74E+08	1.74E+08	933	13.333333
1	1.86E+08	1.86E+08	14397	13.333333
1	2.08E+08	2.08E+08	1585	13.333333
1	2.08E+08	2.08E+08	381214	13.333333
1	2.47E+08	2.47E+08	49107	13.333333
2	39249998	39286024	36027	13.333333
2	61223914	61848845	624932	13.333333
3	1.56E+08	1.56E+08	11871	13.333333
4	1810306	3434075	1623770	13.333333
4	84193142	84200133	6992	13.333333
5	61645765	61645975	211	13.333333
5	61657411	61682922	25512	13.333333
5	68911643	69288477	376835	13.333333
5	71554990	72337850	782861	13.333333
6	31777500	31783348	5849	13.333333
6	32412626	32412763	138	13.333333
6	73497268	74303319	806052	13.333333
8	38092084	38099747	7664	13.333333
8	94767910	94777910	10001	13.333333
8	94793916	94830376	36461	13.333333
9	1.35E+08	1.35E+08	87	13.333333
10	94369290	94373202	3913	13.333333
10	94405187	94409813	4627	13.333333
11	77812193	77814020	1828	13.333333
11	85714349	85714505	157	13.333333
12	32751385	32751551	167	13.333333
12	32786500	32797205	10706	13.333333
14	50666488	50666621	134	13.333333
15	41562793	41961373	398581	13.333333
15	44892847	44898259	5413	13.333333
15	51285562	51293272	7711	13.333333
15	63673002	64226370	553369	13.333333
15	65275796	65276001	206	13.333333
15	78770736	78834731	63996	13.333333
16	46713139	46715275	2137	13.333333
17	19728497	19729339	843	13.333333
17	34151164	34169312	18149	13.333333
17	44270027	44270188	162	13.333333
22	40758971	42373034	1614064	13.333333
22	42373034	42373060	27	13.333333
1	1.74E+08	1.75E+08	725691	10
1	1.86E+08	1.86E+08	3400	10
1	1.86E+08	1.86E+08	58	10
1	2.06E+08	2.06E+08	5482	10
1	2.08E+08	2.08E+08	224	10
1	2.08E+08	2.08E+08	1314	10
1	2.47E+08	2.47E+08	156	10
2	9658018	9683500	25483	10
2	32289292	32312627	23336	10
2	39240588	39249998	9411	10
2	39286024	39294884	8861	10
2	60834239	61223914	389676	10
2	61848845	64119802	2270958	10
3	1.56E+08	1.57E+08	1088256	10
4	84200133	84205674	5542	10
5	68711926	68911643	199718	10
5	72337850	72660687	322838	10
6	31625489	31777500	152012	10
6	74354308	74530628	176321	10
7	1.57E+08	1.57E+08	487530	10
8	38099747	38099800	54	10
8	94830376	94831418	1043	10
9	39140211	41979303	2839093	10
9	1.28E+08	1.28E+08	566	10
9	1.31E+08	1.31E+08	7978	10
10	94215352	94333786	118435	10
10	94333786	94369290	35505	10
10	94409813	94415113	5301	10
10	1.02E+08	1.02E+08	4014	10
11	63486224	63523594	37371	10
11	77812064	77812193	130	10
11	85687650	85714349	26700	10
11	1.08E+08	1.08E+08	30441	10
12	32797205	34591668	1794464	10
14	50596637	50597479	843	10
14	50666621	50671026	4406	10
14	73822253	73876874	54622	10
15	20190548	20575646	385099	10
15	23052587	23060821	8235	10

15	63579805	63673002	93198	10
16	70551635	70553577	1943	10
17	19728354	19728497	144	10
17	29123434	29124350	917	10
17	29667773	29669971	2199	10
17	29704134	30190276	486143	10
17	34151035	34151164	130	10
17	34169312	34169542	231	10
17	44248362	44269718	21357	10
17	44269718	44270027	310	10
17	44302802	44788332	485531	10
19	11200038	11230886	30849	10
19	37207281	38919986	1712706	10
20	43059979	44350752	1290774	10
21	44496313	44496400	88	10
22	40758929	40758971	43	10
22	42373060	42373066	7	10
X	40460110	41599792	1139683	10
X	1.2E+08	1.2E+08	3840	10

Supplementary Table 5. Chromosomal regions with gains and losses present in > 10% of SALS1 patients.

Duplications

Chromosome	Start	Stop	Aberration Size (bps)	Frequency (%)
14	31552632	31552690	59	76.47059
17	17716576	17720711	4136	70.588234
X	1.22E+08	1.22E+08	18149	70.588234
X	1.22E+08	1.22E+08	161	64.70588
7	1E+08	1E+08	134	58.82353
X	1.22E+08	1.22E+08	261	58.82353
X	1.22E+08	1.22E+08	123377	58.82353
17	17720711	17726812	6102	52.941177
7	1E+08	1E+08	2247	47.058823
7	1E+08	1E+08	357	47.058823
9	1.31E+08	1.31E+08	6600	47.058823
17	17726812	17726864	53	47.058823
7	1E+08	1E+08	3085	41.17647
19	50766570	50795492	28923	41.17647
2	2.42E+08	2.42E+08	71409	35.294117
9	1.29E+08	1.29E+08	79088	35.294117
X	1.22E+08	1.22E+08	88	35.294117
2	1.28E+08	1.28E+08	623404	29.411764
9	1.29E+08	1.29E+08	104713	29.411764
9	1.29E+08	1.29E+08	2408	29.411764
9	1.31E+08	1.31E+08	622	29.411764
10	99511112	99519055	7944	29.411764
17	17726864	17740072	13209	29.411764
22	24376158	24384300	8143	29.411764
2	1.28E+08	1.28E+08	7770	23.529411
5	37834861	37835437	577	23.529411
9	1.29E+08	1.29E+08	77	23.529411
10	99504592	99511112	6521	23.529411
17	17715816	17716576	761	23.529411
17	56350271	56357941	7671	23.529411
17	77086157	77097800	11644	23.529411
19	50795492	50796566	1075	23.529411
X	1.53E+08	1.54E+08	475280	23.529411
1	1.65E+08	1.65E+08	158600	17.647058
1	2.05E+08	2.05E+08	29273	17.647058
1	2.08E+08	2.08E+08	56210	17.647058
2	1.28E+08	1.28E+08	1900	17.647058
2	1.28E+08	1.28E+08	239	17.647058
2	2.42E+08	2.42E+08	936	17.647058
2	2.42E+08	2.42E+08	8449	17.647058
5	1.68E+08	1.68E+08	170	17.647058
5	1.77E+08	1.77E+08	11723	17.647058
9	1.29E+08	1.29E+08	80299	17.647058
15	89381841	90198653	816813	17.647058
17	17740072	17740236	165	17.647058
17	34198604	34417495	218892	17.647058
17	77097800	77099247	1448	17.647058
20	1963661	2384209	420549	17.647058
20	23614298	23618518	4221	17.647058
22	18907140	18918487	11348	17.647058
Y	4114337	8158564	4044228	17.647058
Y	18149636	21882048	3732413	17.647058
1	2337815	6557050	4219236	11.764706
1	16348551	22222904	5874354	11.764706
1	55331123	55527185	196063	11.764706
1	1.65E+08	1.65E+08	43557	11.764706
1	1.65E+08	1.65E+08	1474	11.764706
1	2.04E+08	2.05E+08	887356	11.764706
1	2.05E+08	2.05E+08	179	11.764706
2	20818975	20850933	31959	11.764706
2	74596602	74603884	7283	11.764706
2	1.28E+08	1.28E+08	520	11.764706
4	41700207	41748255	48049	11.764706
4	1.02E+08	1.02E+08	47394	11.764706
5	37824072	37834861	10790	11.764706
5	37835437	37835564	128	11.764706
5	1.38E+08	1.38E+08	4840	11.764706
6	31919858	32148778	228921	11.764706
6	32485851	32485872	22	11.764706
6	32485872	32486277	406	11.764706
6	32486277	32489896	3620	11.764706

Deletions

Chromosome	Start	Stop	Aberration Size (bps)	Frequency (%)
1	47716828	47775972	59145	88.23529
20	33986975	35569474	1582500	88.23529
20	35569474	35575306	5833	82.35294
1	47435653	47716828	281176	76.47059
2	32339724	32429834	90111	76.47059
3	1.56E+08	1.56E+08	8949	76.47059
9	1.28E+08	1.28E+08	607	76.47059
1	47775972	47776133	162	70.588234
2	32323849	32339724	15876	70.588234
2	32429834	32432100	2267	70.588234
3	1.56E+08	1.56E+08	3669	70.588234
3	1.56E+08	1.56E+08	58	70.588234
9	1.28E+08	1.28E+08	122189	70.588234
1	46685884	47435653	749770	64.70588
2	32314495	32323849	9355	64.70588
3	1.56E+08	1.56E+08	101	64.70588
5	69372472	70247951	875480	64.70588
10	1.21E+08	1.21E+08	421	64.70588
2	32312639	32314495	1857	58.82353
3	1.56E+08	1.56E+08	103	58.82353
5	69372317	69372472	156	58.82353
10	1.21E+08	1.21E+08	111447	58.82353
12	1.12E+08	1.12E+08	35010	58.82353
14	92527804	92560197	32394	58.82353
2	32312627	32312639	13	52.941177
2	32432100	32434737	2638	52.941177
5	70247951	70307124	59174	52.941177
12	1.12E+08	1.12E+08	2539	52.941177
14	92525408	92527804	2397	52.941177
16	46697028	46703042	6015	52.941177
22	32156586	32188108	31523	52.941177
2	64143925	64169715	25791	47.058823
3	1.56E+08	1.56E+08	2765	47.058823
5	70307124	70309855	2732	47.058823
14	92560197	92562372	2176	47.058823
15	50924954	50975081	50128	47.058823
16	46696885	46697028	144	47.058823
17	2541457	2569364	27908	47.058823
22	32154622	32156586	1965	47.058823
22	32188108	32200902	12795	47.058823
2	64124630	64143925	19296	41.17647
2	64169715	64169735	21	41.17647
2	64169735	64193249	23515	41.17647
5	70309855	70320678	10824	41.17647
6	32486277	32489896	3620	41.17647
12	1.12E+08	1.12E+08	156	41.17647
15	50911927	50924954	13028	41.17647
16	46694461	46696885	2425	41.17647
17	2541347	2541457	111	41.17647
17	2569364	2569504	141	41.17647
20	33834793	33986975	152183	41.17647
22	32154613	32154622	10	41.17647
22	32200902	32205658	4757	41.17647
1	63876791	63885210	8420	35.294117
1	2.47E+08	2.47E+08	140	35.294117
2	64119802	64124630	4829	35.294117
2	64193249	64199380	6132	35.294117
5	70320678	71554990	1234313	35.294117
6	32485872	32486277	406	35.294117
6	32489896	32489935	40	35.294117
7	22771002	23017142	246141	35.294117
12	1.12E+08	1.12E+08	29476	35.294117
14	92413787	92525408	111622	35.294117
14	1.04E+08	1.04E+08	62601	35.294117
15	21200700	22432687	1231988	35.294117
15	50872974	50911927	38954	35.294117
16	46703042	46712898	9857	35.294117
17	19753054	19769087	16034	35.294117
18	9117738	9134199	16462	35.294117
22	32205658	32206586	929	35.294117
1	47776133	47778637	2505	29.411764

6	32489896	32489935	40	11.764706
6	32489935	32489967	33	11.764706
6	32489967	32498046	8080	11.764706
6	1.53E+08	1.53E+08	764	11.764706
7	4823028	4830775	7748	11.764706
7	94953690	94983307	29618	11.764706
7	1E+08	1E+08	309	11.764706
8	42037953	42065260	27308	11.764706
8	1.45E+08	1.45E+08	388862	11.764706
9	87283500	87284663	1164	11.764706
9	1.39E+08	1.39E+08	47852	11.764706
9	1.39E+08	1.39E+08	1537	11.764706
10	99504442	99504592	151	11.764706
11	2161421	2161583	163	11.764706
11	1.17E+08	1.17E+08	2179	11.764706
12	52096760	52145163	48404	11.764706
12	57587270	57605854	18585	11.764706
12	1.03E+08	1.03E+08	60690	11.764706
14	1.05E+08	1.05E+08	21095	11.764706
15	42678271	42695967	17697	11.764706
16	10273981	10276571	2591	11.764706
16	28488776	28503629	14854	11.764706
16	57653630	57698265	44636	11.764706
16	89987339	89988654	1316	11.764706
17	8791519	8794231	2713	11.764706
17	17740236	17927922	187687	11.764706
17	34171478	34198604	27127	11.764706
17	56349278	56350271	994	11.764706
17	56357941	56358113	173	11.764706
17	75369546	77086157	1716612	11.764706
17	77099247	78092622	993376	11.764706
19	50760516	50766570	6055	11.764706
19	50796566	50836689	40124	11.764706
20	2384209	2411082	26874	11.764706
20	23618518	23618656	139	11.764706
20	60718894	60718952	59	11.764706
20	61987803	62599159	611357	11.764706
22	18901042	18907140	6099	11.764706
22	18918487	19929407	1010921	11.764706
22	24384300	24828385	444086	11.764706
22	37621845	37640194	18350	11.764706
X	1.54E+08	1.54E+08	890923	11.764706
Y	3713948	4114337	400390	11.764706
Y	8158564	9072145	913582	11.764706
Y	17493425	18149636	656212	11.764706
Y	21882048	23799790	1917743	11.764706

1	63836432	63876791	40360	29.411764
1	63885210	63902525	17316	29.411764
6	31783348	31797880	14533	29.411764
7	23017142	23017909	768	29.411764
9	1.27E+08	1.28E+08	1188578	29.411764
10	76099253	76768859	669607	29.411764
11	85714505	85737381	22877	29.411764
14	1.04E+08	1.04E+08	27229	29.411764
14	1.04E+08	1.04E+08	178091	29.411764
15	20575646	21200700	625055	29.411764
15	22432687	23046598	613912	29.411764
15	93487634	93489253	1620	29.411764
17	2569504	4605227	2035724	29.411764
17	19741803	19753054	11252	29.411764
17	19769087	19769218	132	29.411764
17	30190437	30226829	36393	29.411764
22	32206586	32206711	126	29.411764
1	63902525	63903304	780	23.529411
1	2.47E+08	2.47E+08	6692	23.529411
5	61645975	61682922	36948	23.529411
5	69288477	69372317	83841	23.529411
5	71554990	72337850	782861	23.529411
6	31797880	31865577	67698	23.529411
6	32485851	32485872	22	23.529411
9	1.26E+08	1.27E+08	742981	23.529411
9	1.28E+08	1.28E+08	128	23.529411
10	75960468	76099253	138786	23.529411
10	96540191	96580305	40115	23.529411
11	77814989	77815111	123	23.529411
12	32778695	32786500	7806	23.529411
15	23046598	23048496	1899	23.529411
15	50849380	50872974	23595	23.529411
16	46693906	46694461	556	23.529411
16	46712898	46713139	242	23.529411
17	19729339	19741803	12465	23.529411
17	29110052	29114424	4373	23.529411
17	30190276	30190437	162	23.529411
17	30226829	30228745	1917	23.529411
17	30228745	30327458	98714	23.529411
17	44270188	44302802	32615	23.529411
22	32206711	32215105	8395	23.529411
1	44173154	44201940	28787	17.647058
1	2.08E+08	2.08E+08	19860	17.647058
1	2.47E+08	2.47E+08	3431	17.647058
1	2.47E+08	2.47E+08	5171	17.647058
2	32289292	32312627	23336	17.647058
2	32434737	32610141	175405	17.647058
2	61223914	61848845	624932	17.647058
5	61645765	61645975	211	17.647058
5	68711926	69288477	576552	17.647058
5	72337850	72660687	322838	17.647058
6	31777500	31783348	5849	17.647058
6	32485173	32485851	679	17.647058
6	32489935	32489967	33	17.647058
8	94777910	94793916	16007	17.647058
9	1.08E+08	1.08E+08	16132	17.647058
9	1.35E+08	1.35E+08	33833	17.647058
10	70432579	70441196	8618	17.647058
11	77814020	77814989	970	17.647058
11	85687650	85714505	26856	17.647058
12	32751551	32778695	27145	17.647058
12	32786500	32797205	10706	17.647058
12	1.12E+08	1.12E+08	2013	17.647058
14	50597479	50671026	73548	17.647058
14	73822253	73876874	54622	17.647058
15	23048496	23060821	12326	17.647058
15	41535920	41562793	26874	17.647058
15	44900781	44951263	50483	17.647058
15	50975081	50978890	3810	17.647058
15	50978890	51293272	314383	17.647058
15	63673002	65276001	1603000	17.647058
15	78732085	78770736	38652	17.647058
17	19728354	19729339	986	17.647058
17	29114424	29123314	8891	17.647058
17	44270027	44270188	162	17.647058
19	11200038	11230886	30849	17.647058

1	10434465	10635417	200953	11.764706
1	1.74E+08	1.74E+08	30261	11.764706
1	2.08E+08	2.08E+08	2109	11.764706
1	2.27E+08	2.27E+08	251	11.764706
1	2.47E+08	2.47E+08	49107	11.764706
1	2.47E+08	2.47E+08	156	11.764706
2	9658018	9694645	36628	11.764706
2	32249023	32289292	40270	11.764706
2	60834239	61223914	389676	11.764706
2	61848845	64119802	2270958	11.764706
2	2.22E+08	2.22E+08	77	11.764706
3	1.56E+08	1.56E+08	11871	11.764706
4	1810306	3434075	1623770	11.764706
4	84193142	84205674	12533	11.764706
5	61682922	65272011	3589090	11.764706
5	72660687	72980685	319999	11.764706
5	78111435	78111897	463	11.764706
6	31625102	31777500	152399	11.764706
6	32412763	32485173	72411	11.764706
6	43112641	43738977	626337	11.764706
6	43738977	43739768	792	11.764706
6	88187257	88224182	36926	11.764706
6	88265277	88279303	14027	11.764706
7	1.57E+08	1.57E+08	487530	11.764706
8	94767910	94777910	10001	11.764706
8	94793916	94831517	37602	11.764706
9	39140211	41979303	2839093	11.764706
9	1.28E+08	1.28E+08	566	11.764706
9	1.35E+08	1.35E+08	9420	11.764706
9	1.35E+08	1.35E+08	1538	11.764706
10	89653839	89717626	63788	11.764706
10	94215352	94415113	199762	11.764706
11	63486224	63523594	37371	11.764706
11	77812193	77814020	1828	11.764706
11	85687540	85687650	111	11.764706
12	32751385	32751551	167	11.764706
12	32797205	34591668	1794464	11.764706
14	20942890	20943222	333	11.764706
14	50596637	50597479	843	11.764706
14	50671026	50671179	154	11.764706
14	50671179	50768742	97564	11.764706
14	73789851	73822253	32403	11.764706
14	73876874	74425061	548188	11.764706
14	1.03E+08	1.03E+08	45679	11.764706
15	41562793	41961373	398581	11.764706
15	44898259	44900781	2523	11.764706
15	44951263	44952618	1356	11.764706
15	63579635	63673002	93368	11.764706
15	65276001	65281708	5708	11.764706
15	78770736	78834731	63996	11.764706
15	93487545	93487634	90	11.764706
15	93489253	93496573	7321	11.764706
16	46713139	46715275	2137	11.764706
16	70551635	70553577	1943	11.764706
17	15880430	17218532	1338103	11.764706
17	19720206	19728354	8149	11.764706
17	29123314	29123434	121	11.764706
17	29663929	29669971	6043	11.764706
17	29704134	30190276	486143	11.764706
17	44248362	44269718	21357	11.764706
17	44269718	44270027	310	11.764706
17	44302802	44788332	485531	11.764706
17	61560736	61560846	111	11.764706
18	9134199	9134357	159	11.764706
19	11230886	11230989	104	11.764706
20	8770749	8770961	213	11.764706
20	31706596	33834793	2128198	11.764706
21	44496313	44496400	88	11.764706
22	40758971	42373034	1614064	11.764706
22	42373034	42373066	33	11.764706
X	40460110	41599792	1139683	11.764706

Supplementary Table 6. Chromosomal regions with gains and losses present in > 10% of SALS2 patients.

Duplications

Chromosome	Start	Stop	Aberration Size (bps)	Frequency (%)
14	31552632	31552690	59	76.92308
17	17716576	17720711	4136	69.23077
22	24376158	24384300	8143	69.23077
17	17720711	17726812	6102	61.53846
9	1.29E+08	1.29E+08	186207	53.846153
9	1.31E+08	1.31E+08	6600	53.846153
17	17715816	17716576	761	53.846153
1	55331123	55527185	196063	46.153847
2	1.28E+08	1.28E+08	631173	46.153847
2	2.42E+08	2.42E+08	71409	46.153847
5	1.77E+08	1.77E+08	15676	46.153847
17	17726812	17726864	53	46.153847
19	50766570	50795529	28960	46.153847
20	23614298	23618518	4221	46.153847
20	62037795	62599159	561365	46.153847
X	1.22E+08	1.22E+08	2083	46.153847
1	2337815	6557050	4219236	38.46154
1	2.08E+08	2.08E+08	55564	38.46154
2	1.28E+08	1.28E+08	1900	38.46154
9	1.29E+08	1.29E+08	80299	38.46154
10	99504592	99519055	14464	38.46154
11	17408294	17450206	41913	38.46154
14	1.05E+08	1.05E+08	21095	38.46154
15	89346786	90198653	851868	38.46154
16	28488776	28502793	14018	38.46154
19	50364748	50766570	401823	38.46154
19	50795529	50796566	1038	38.46154
20	23618518	23618656	139	38.46154
20	61992392	62037795	45404	38.46154
X	1.22E+08	1.22E+08	16487	38.46154
X	1.53E+08	1.54E+08	475280	38.46154
1	16348551	22216964	5868414	30.76923
1	1.65E+08	1.65E+08	202242	30.76923
1	2.05E+08	2.05E+08	29273	30.76923
1	2.08E+08	2.08E+08	647	30.76923
1	2.08E+08	2.08E+08	280	30.76923
1	2.27E+08	2.27E+08	97122	30.76923
5	37834861	37834984	124	30.76923
6	31919858	32148778	228921	30.76923
7	2281791	4830775	2548985	30.76923
7	1E+08	1E+08	134	30.76923
9	87283500	87285883	2384	30.76923
9	1.29E+08	1.29E+08	77	30.76923
9	1.31E+08	1.31E+08	622	30.76923
11	17450206	17464859	14654	30.76923
16	28502793	28503629	837	30.76923
17	17726864	17740072	13209	30.76923
17	56350271	56357941	7671	30.76923
19	50364607	50364748	142	30.76923
19	50796566	50836689	40124	30.76923
Y	4114337	8158564	4044228	30.76923
1	22216964	22222489	5526	23.076923
1	1.56E+08	1.57E+08	790848	23.076923
1	1.65E+08	1.65E+08	1388	23.076923
1	2.05E+08	2.05E+08	179	23.076923
1	2.08E+08	2.08E+08	18483	23.076923
1	2.27E+08	2.27E+08	17578	23.076923
2	20818975	20850933	31959	23.076923
2	1.28E+08	1.28E+08	239	23.076923
2	2.42E+08	2.42E+08	936	23.076923
4	1.02E+08	1.03E+08	1154706	23.076923
5	1.38E+08	1.38E+08	4209	23.076923
5	1.68E+08	1.69E+08	386913	23.076923
6	29910241	29910772	532	23.076923
6	32148778	32149461	684	23.076923
6	32166526	32191834	25309	23.076923
6	32485173	32489935	4763	23.076923
7	1E+08	1E+08	5687	23.076923
8	1.45E+08	1.45E+08	413943	23.076923
9	1.31E+08	1.31E+08	252	23.076923

Deletions

Chromosome	Start	Stop	Aberration Size (bps)	Frequency (%)
3	1.56E+08	1.56E+08	12674	69.23077
20	33986975	35575306	1588332	69.23077
1	47767175	47770585	3411	61.53846
18	9117815	9134199	16385	61.53846
3	1.56E+08	1.56E+08	103	53.846153
12	1.12E+08	1.12E+08	37511	53.846153
1	47435653	47767175	331523	46.153847
1	47770585	47770755	171	46.153847
2	32314495	32409410	94916	46.153847
10	70432579	70441196	8618	46.153847
14	92527804	92562372	34569	46.153847
16	46695793	46703042	7250	46.153847
17	2541457	2569504	28048	46.153847
18	9117738	9117815	78	46.153847
1	46685884	47435653	749770	38.46154
2	32312627	32314495	1869	38.46154
2	32409410	32434737	25328	38.46154
3	1.56E+08	1.56E+08	101	38.46154
5	69372317	70247951	875635	38.46154
6	74303319	74351597	48279	38.46154
9	1.28E+08	1.28E+08	122795	38.46154
10	1.21E+08	1.21E+08	111867	38.46154
12	1.12E+08	1.12E+08	38	38.46154
14	92413787	92527804	114018	38.46154
15	44912321	44951263	38943	38.46154
15	50911927	50955264	43338	38.46154
16	46694461	46695793	1333	38.46154
16	46703042	46713139	10098	38.46154
17	2541347	2541457	111	38.46154
17	2569504	2580007	10504	38.46154
18	9134199	9134357	159	38.46154
22	32154613	32206586	51974	38.46154
1	47770755	47776133	5379	30.76923
1	2.08E+08	2.08E+08	21968	30.76923
2	64143925	64199380	55456	30.76923
3	1.56E+08	1.56E+08	2765	30.76923
6	32412763	32485173	72411	30.76923
6	32485173	32489935	4763	30.76923
6	73497268	74303319	806052	30.76923
6	74351597	74354308	2712	30.76923
9	1.35E+08	1.35E+08	41086	30.76923
12	1.12E+08	1.12E+08	156	30.76923
15	21200700	23044610	1843911	30.76923
15	44900781	44912321	11541	30.76923
15	44951263	44952618	1356	30.76923
15	50955264	50975081	19818	30.76923
17	2580007	4605227	2025221	30.76923
22	32206586	32206711	126	30.76923
1	63836485	63903304	66820	23.076923
1	1.74E+08	1.74E+08	29292	23.076923
1	1.86E+08	1.86E+08	14397	23.076923
1	2.08E+08	2.08E+08	1585	23.076923
1	2.08E+08	2.08E+08	381214	23.076923
2	39249998	39286024	36027	23.076923
2	64119802	64143925	24124	23.076923
2	2.22E+08	2.22E+08	77	23.076923
6	32412626	32412763	138	23.076923
6	74354308	74530628	176321	23.076923
8	38092084	38099800	7717	23.076923
9	1.08E+08	1.08E+08	289	23.076923
9	1.35E+08	1.35E+08	2167	23.076923
9	1.35E+08	1.35E+08	1452	23.076923
10	76154068	76768859	614792	23.076923
10	94373202	94405187	31986	23.076923
12	1.12E+08	1.12E+08	31488	23.076923
15	20575646	21200700	625055	23.076923
15	44892847	44900781	7935	23.076923
15	50849380	50911927	62548	23.076923
16	46693906	46694461	556	23.076923
17	19752773	19769218	16446	23.076923

9	1.36E+08	1.4E+08	3671630	23.076923
11	424565	7324653	6900089	23.076923
11	17464859	17470241	5383	23.076923
11	45906997	45925785	18789	23.076923
12	57587270	57605854	18585	23.076923
12	1.25E+08	1.25E+08	291	23.076923
14	99641841	1E+08	551735	23.076923
15	42678271	42703427	25157	23.076923
15	50978890	51201129	222240	23.076923
16	1250785	2147716	896932	23.076923
16	57685031	57698265	13235	23.076923
17	75277604	78092622	2815019	23.076923
19	50836689	50885869	49181	23.076923
20	1963661	2411082	447422	23.076923
21	43693384	44496400	803017	23.076923
21	44496400	46931240	2434841	23.076923
22	37622611	37640194	17584	23.076923
X	1.22E+08	1.22E+08	123377	23.076923
Y	8158564	8694578	536015	23.076923
Y	17890214	22086267	4196054	23.076923
1	1950820	1950907	88	15.384615
1	1950907	2337815	386909	15.384615
1	15834248	16348551	514304	15.384615
1	22222489	22379326	156838	15.384615
1	55527185	55529886	2702	15.384615
1	65854982	65855380	399	15.384615
1	1.1E+08	1.1E+08	186810	15.384615
1	1.65E+08	1.65E+08	213	15.384615
1	1.65E+08	1.65E+08	27410	15.384615
1	2.08E+08	2.08E+08	141121	15.384615
1	2.27E+08	2.27E+08	462663	15.384615
2	20818280	20818975	696	15.384615
2	74589902	74590293	392	15.384615
2	1.53E+08	1.53E+08	335	15.384615
2	1.72E+08	1.72E+08	14175	15.384615
2	2.42E+08	2.42E+08	8449	15.384615
3	1.28E+08	1.28E+08	9415	15.384615
4	473916	1810305	1336390	15.384615
4	3434076	9785385	6351310	15.384615
4	41216529	41259802	43274	15.384615
4	41700207	41748255	48049	15.384615
4	1.02E+08	1.02E+08	168033	15.384615
4	1.85E+08	1.85E+08	275	15.384615
5	37824072	37834861	10790	15.384615
5	37834984	37835437	454	15.384615
5	70307077	70309855	2779	15.384615
5	1.21E+08	1.22E+08	235225	15.384615
5	1.38E+08	1.38E+08	2289	15.384615
5	1.38E+08	1.38E+08	399	15.384615
5	1.68E+08	1.68E+08	514463	15.384615
5	1.69E+08	1.77E+08	7939684	15.384615
6	29910772	29911268	497	15.384615
6	32149461	32166526	17066	15.384615
6	32191834	32191906	73	15.384615
6	32489935	32489967	33	15.384615
7	4830775	4832078	1304	15.384615
8	42037953	42065260	27308	15.384615
8	1.45E+08	1.45E+08	1358	15.384615
9	35068399	35072760	4362	15.384615
9	87283404	87283500	97	15.384615
9	1.16E+08	1.16E+08	12322	15.384615
9	1.36E+08	1.36E+08	580363	15.384615
10	94333786	94353243	19458	15.384615
11	45832404	45906997	74594	15.384615
11	45925785	46750928	825144	15.384615
12	57549817	57587270	37454	15.384615
12	57605854	57606775	922	15.384615
12	1.25E+08	1.25E+08	42	15.384615
13	51483764	51484234	471	15.384615
13	99064338	99099142	34805	15.384615
14	98514914	99641841	1126928	15.384615
15	27017550	27018935	1386	15.384615
15	42703427	42703603	177	15.384615
16	931290	1250785	319496	15.384615
16	2147716	6069877	3922162	15.384615
17	29110052	29124350	14299	23.076923
17	29663929	29667773	3845	23.076923
17	30212981	30228745	15765	23.076923
17	30228745	30326197	97453	23.076923
17	34151035	34169542	18508	23.076923
17	61560736	61560846	111	23.076923
20	43059979	44350752	1290774	23.076923
1	44173154	44201940	28787	15.384615
1	63836432	63836485	54	15.384615
1	1.74E+08	1.74E+08	1427	15.384615
1	1.74E+08	1.79E+08	5492971	15.384615
1	1.86E+08	1.86E+08	3400	15.384615
1	1.86E+08	1.86E+08	58	15.384615
1	2.06E+08	2.06E+08	5691	15.384615
1	2.08E+08	2.08E+08	224	15.384615
1	2.08E+08	2.08E+08	1314	15.384615
1	2.47E+08	2.47E+08	64537	15.384615
2	32434737	32610141	175405	15.384615
2	39240556	39249998	9443	15.384615
2	39286024	39294940	8917	15.384615
3	1.56E+08	1.57E+08	1100126	15.384615
4	1810306	3434075	1623770	15.384615
4	84193142	84200133	6992	15.384615
6	32489935	32489967	33	15.384615
6	74530628	76884915	2354288	15.384615
7	22771002	23018107	247106	15.384615
8	38090450	38092084	1635	15.384615
8	38099800	38899455	799656	15.384615
8	94767910	94830376	62467	15.384615
9	1.08E+08	1.08E+08	15844	15.384615
9	1.31E+08	1.31E+08	14542	15.384615
9	1.35E+08	1.35E+08	87	15.384615
10	75960468	76154068	193601	15.384615
10	94369290	94373202	3913	15.384615
10	94405187	94409813	4627	15.384615
10	1.02E+08	1.02E+08	4014	15.384615
11	77812064	77815111	3048	15.384615
11	85714533	85737381	22849	15.384615
11	1.08E+08	1.08E+08	51012	15.384615
12	32751385	32786500	35116	15.384615
12	49692322	51495933	1803612	15.384615
12	64178732	64895282	716551	15.384615
14	50597479	50666488	69010	15.384615
14	1.04E+08	1.04E+08	267919	15.384615
15	20190548	20575646	385099	15.384615
15	23044610	23052587	7978	15.384615
15	41535920	41961373	425454	15.384615
15	50975081	50978890	3810	15.384615
15	50978890	51285562	306673	15.384615
15	64226370	65275796	1049427	15.384615
15	78732085	78893680	161596	15.384615
16	46713139	46715275	2137	15.384615
17	19729339	19752773	23435	15.384615
17	29663223	29663929	707	15.384615
17	30190276	30212981	22706	15.384615
17	30326197	30327458	1262	15.384615
17	34144751	34151035	6285	15.384615
17	34169542	34171318	1777	15.384615
17	44302758	44302802	45	15.384615
19	37207281	38919986	1712706	15.384615
20	33834793	33986975	152183	15.384615
20	44350752	44732891	382140	15.384615
22	40758929	42373034	1614106	15.384615
22	42373034	42373060	27	15.384615
X	1.2E+08	1.2E+08	5016	15.384615

16	7680488	7743336	62849	15.384615
16	10273832	10276571	2740	15.384615
16	57653630	57685031	31402	15.384615
16	57698265	57698622	358	15.384615
16	70323409	70517903	194495	15.384615
16	72093075	74808756	2715682	15.384615
17	1648197	1658560	10364	15.384615
17	8785037	8808289	23253	15.384615
17	17715423	17715816	394	15.384615
17	17740072	17740236	165	15.384615
17	26083763	27076535	992773	15.384615
18	77439765	77496404	56640	15.384615
19	1000387	1549191	548805	15.384615
20	1963574	1963661	88	15.384615
20	2411082	2636794	225713	15.384615
20	3869789	3870616	828	15.384615
20	60718894	61992392	1273499	15.384615
21	46931240	46933629	2390	15.384615
22	24384300	24828385	444086	15.384615
22	37621845	37622611	767	15.384615
22	37640194	38577837	937644	15.384615
22	42486755	42526821	40067	15.384615
X	47433998	53349964	5915967	15.384615
X	62875495	63005325	129831	15.384615
X	1.22E+08	1.22E+08	88	15.384615
Y	8694578	9072145	377568	15.384615
Y	14389646	17890214	3500569	15.384615
Y	22086267	24874322	2788056	15.384615

Supplementary Table 7. List of genes located in the most recurrent CNVs of SALS patients (pentrance > 10%).

Duplications						Deletions					
Gene name	Gene description	Chromosome	Gene start (bp)	Gene end (bp)	Size (bp)	Gene name	Gene description	Chromosome	Gene start (bp)	Gene end (bp)	Size (bp)
ABCA2	ATP binding cassette subfamily A member 2	9	139901686	139923367	21682	ACE	angiotensin I converting enzyme	17	61554422	61599205	44784
ABCC8	ATP binding cassette subfamily C member 8	11	17414432	17498449	84018	ADAM17	ADAM metallopeptidase domain 17	2	9628615	9695921	67307
ABCG1	ATP binding cassette subfamily G member 1	21	43619799	43717354	97556	**ADK**	adenosine kinase	10	75910960	76469061	558102
ABHD2	abhydrolase domain containing 2	15	89630690	89745591	114902	ADSL	adenylosuccinate lyase	22	40742507	40786467	43961
ACAN	aggrecan	15	89346674	89418585	71912	ALG6	ALG6, alpha-1,3-glucosyltransferase	1	63833261	63904233	70973
ACHE	acetylcholinesterase (Cartwright blood group)	7	100487615	100494594	6980	AP4E1	adaptor related protein complex 4 epsilon 1 subunit	15	51200869	51298097	97229
ADARB1	adenosine deaminase, RNA specific B1	21	46493768	46646478	152711	APH1B	aph-1 homolog B, gamma-secretase subunit	15	63568217	63601325	33109
ADGRG1	adhesion G protein-coupled receptor G1	16	57653442	57698944	45503	APOM	apolipoprotein M	6	31620193	31625987	5795
AGER	advanced glycosylation end-product specific receptor	6	32148745	32152101	3357	ATM	ATM serine/threonine kinase	11	108093211	108239829	146619
AKAP4	A-kinase anchoring protein 4	X	49955406	49965664	10259	ATP6AP2	ATPase H+ transporting accessory protein 2	X	40440146	40465889	25744
AKT1	AKT serine/threonine kinase 1	14	105235686	105262088	26403	**ATXN2**	ataxin 2	12	111890018	112037480	147463
ALDH4A1	aldehyde dehydrogenase 4 family member A1	1	19197926	19229275	31350	ATXN3	ataxin 3	14	92524896	92572965	48070
ALPL	alkaline phosphatase, liver/bone/kidney	1	21835858	21904905	69048	B3GNT2	UDP-GlcNAc:betaGal beta-1,3-N-acetylglucosaminyltransferase 2	2	62423248	62451866	28619
ANO9	anoctamin 9	11	417933	442011	24079	BDP1	B double prime 1, subunit of RNA polymerase III transcription initiation factor IIIB	5	70751442	70863649	112208
AP2A2	adaptor related protein complex 2 alpha 2 subunit	11	924894	1012239	87346	BIRC6	baculoviral IAP repeat containing 6	2	32582096	32843966	261871
AP4E1	adaptor related protein complex 4 epsilon 1 subunit	15	51200869	51298097	97229	CA12	carbonic anhydrase 12	15	63613577	63674360	60784
AP4S1	adaptor related protein complex 4 sigma 1 subunit	14	31494312	31562818	68507	CASK	calcium/calmodulin dependent serine protein kinase	X	41374187	41782716	408530
AP5Z1	adaptor related protein complex 5 zeta 1 subunit	7	4815253	4833943	18691	CBS	cystathionine-beta-synthase	21	44473301	44497053	23753
APBB1	amyloid beta precursor protein binding family B member 1	11	6416355	6440644	24290	CCT4	chaperonin containing TCP1 subunit 4	2	62095224	62115939	20716
APBB2	amyloid beta precursor protein binding family B member 2	4	40812044	41218731	406688	CD109	CD109 molecule	6	74405508	74538040	132533
ARHGEF9	Cdc42 guanine nucleotide exchange factor 9	X	62854847	63005426	150580	CHD2	chromodomain helicase DNA binding protein 2	15	93426526	93571237	144712
ATP13A2	ATPase 13A2	1	17312453	17338423	25971	CHP1	calcineurin like EF-hand protein 1	15	41523037	41574043	51007
BANK1	B-cell scaffold protein with ankyrin repeats 1	4	102332443	102995969	663527	CNTNAP3	contactin associated protein-like 3	9	39072764	39288312	215549
**BCL11B**	B-cell CLL/lymphoma 11B	14	99635624	99737861	102238	COG4	component of oligomeric golgi complex 4	16	70514471	70557468	42998
BIN1	bridging integrator 1	2	127805603	127864931	59329	COQ2	coenzyme Q2, polyprenyltransferase	4	84182689	84206067	23379
BRD3	bromodomain containing 3	9	136895427	136933657	38231	CR1	complement C3b/C4b receptor 1 (Knops blood group)	1	207669492	207813992	144501
C1QTNF1	C1q and TNF related 1	17	77018896	77045870	26975	CRLF3	cytokine receptor like factor 3	17	29096406	29151794	55389
C1QTNF1-AS1	C1QTNF1 antisense RNA 1	17	77015291	77023737	8447	CSNK1G1	casein kinase 1 gamma 1	15	64457716	64648442	190727
C4A	complement C4A (Rodgers blood group)	6	31949801	31970458	20658	CUL4B	cullin 4B	X	119658464	119709649	51186
CABIN1	calcineurin binding protein 1	22	24407642	24574596	166955	CYP2C19	cytochrome P450 family 2 subfamily C member 19	10	96447911	96613017	165107
**CACNA1H**	calcium voltage-gated channel subunit alpha1 H	16	1203241	1271771	68531	DAPK2	death associated protein kinase 2	15	64199235	64364232	164998
CACNB4	calcium voltage-gated channel auxiliary subunit beta 4	2	152689290	152955593	266304	DARS2	aspartyl-tRNA synthetase 2, mitochondrial	1	173793641	173827684	34044
CAMSAP1	calmodulin regulated spectrin associated protein 1	9	138700333	138799074	98742	DDHD2	DDHD domain containing 2	8	38082736	38133076	50341
CAPN3	calpain 3	15	42640301	42704516	64216	DDX3X	DEAD-box helicase 3, X-linked	X	41192651	41223725	31075
CARD11	caspase recruitment domain family member 11	7	2945775	3083579	137805	DENND1A	DENN domain containing 1A	9	126141933	126692431	550499

CBS	cystathionine-beta-synthase	21	44473301	44497053	23753	DEPDC5	DEP domain containing 5	22	32149944	32303012	153069
CCDC120	coiled-coil domain containing 120	X	48911101	48927509	16409	DLGAP4	DLG associated protein 4	20	34894258	35157040	262783
CCKBR	cholecystokinin B receptor	11	6280966	6293357	12392	DNAJB6	DnaJ heat shock protein family (Hsp40) member B6	7	157128075	157210133	82059
CCL3	C-C motif chemokine ligand 3	17	34415602	34417515	1914	EHBP1	EH domain binding protein 1	2	62900986	63273622	372637
CCL5	C-C motif chemokine ligand 5	17	34198495	34207797	9303	EHMT2	euchromatic histone lysine methyltransferase 2	6	31847536	31865464	17929
CD24	CD24 molecule	6	107417708	107422630	4923	EPB41L1	erythrocyte membrane protein band 4.1 like 1	20	34679426	34820721	141296
CDA	cytidine deaminase	1	20915441	20945401	29961	**EPHA4**	EPH receptor A4	2	222282747	222438922	156176
CEP104	centrosomal protein 104	1	3728645	3773778	45134	ERLIN1	ER lipid raft associated 1	10	101909851	101948091	38241
CEP164	centrosomal protein 164	11	117185273	117283984	98712	FAM126A	family with sequence similarity 126 member A	7	22980878	23053749	72872
CHD5	chromodomain helicase DNA binding protein 5	1	6161853	6240183	78331	FAM193A	family with sequence similarity 193 member A	4	2626988	2734292	107305
CHRNA4	cholinergic receptor nicotinic alpha 4 subunit	20	61975420	62009753	34334	FBLN5	fibulin 5	14	92335756	92414331	78576
CIRBP	cold inducible RNA binding protein	19	1259384	1274879	15496	FCHO2	FCH domain only 2	5	72251808	72386349	134542
CLCNKA	chloride voltage-gated channel Ka	1	16345370	16360545	15176	FGD4	FYVE, RhoGEF and PH domain containing 4	12	32552463	32798984	246522
CLCNKB	chloride voltage-gated channel Kb	1	16370272	16383803	13532	FGFR3	fibroblast growth factor receptor 3	4	1795034	1810599	15566
CLN3	CLN3, battenin	16	28477983	28506896	28914	FKTN	fukutin	9	108320411	108403399	82989
CNTN2	contactin 2	1	205012325	205047627	35303	GRK5	G protein-coupled receptor kinase 5	10	120967101	121215131	248031
COL18A1	collagen type XVIII alpha 1 chain	21	46825052	46933634	108583	GUSBP3	glucuronidase, beta pseudogene 3	5	68790040	69006341	216302
CSF1	colony stimulating factor 1	1	110452864	110473614	20751	HERC1	HECT and RLD domain containing E3 ubiquitin protein ligase family member 1	15	63900817	64126141	225325
**CST3**	cystatin C	20	23608534	23619110	10577	HLA-DRA	major histocompatibility complex, class II, DR alpha	6	32407619	32412823	5205
CSTB	cystatin B	21	45192393	45196326	3934	HLA-DRB5	major histocompatibility complex, class II, DR beta 5	6	32485120	32498064	12945
CTSD	cathepsin D	11	1773982	1785222	11241	HNF4A	hepatocyte nuclear factor 4 alpha	20	42984340	43061485	77146
**CYP2D6**	cytochrome P450 family 2 subfamily D member 6	22	42522501	42526908	4408	HSPA1A	heat shock protein family A (Hsp70) member 1A	6	31783291	31785723	2433
CYP46A1	cytochrome P450 family 46 subfamily A member 1	14	100150641	100193638	42998	HSPA1B	heat shock protein family A (Hsp70) member 1B	6	31795512	31798031	2520
CYTH1	cytohesin 1	17	76670130	76778379	108250	HSPA1L	heat shock protein family A (Hsp70) member 1 like	6	31777396	31783437	6042
**DBH**	dopamine beta-hydroxylase	9	136501482	136524466	22985	HSPA5	heat shock protein family A (Hsp70) member 5	9	127997132	128003609	6478
**DCTN1**	dynactin subunit 1	2	74588281	74619214	30934	HTT	huntingtin	4	3076408	3245676	169269
DDOST	dolichyl-diphosphooligosaccharide--protein glycosyltransferase non-catalytic subunit	1	20978270	20988000	9731	HYKK	hydroxylysine kinase	15	78799906	78829714	29809
**DEAF1**	DEAF1, transcription factor	11	644233	706715	62483	IDE	insulin degrading enzyme	10	94211441	94333833	122393
DHCR24	24-dehydrocholesterol reductase	1	55315306	55352891	37586	IL6	interleukin 6	7	22765503	22771621	6119
DIDO1	death inducer-obliterator 1	20	61509090	61569304	60215	IREB2	iron responsive element binding protein 2	15	78729773	78793798	64026
DNAH17	dynein axonemal heavy chain 17	17	76419778	76573476	153699	ITGAE	integrin subunit alpha E	17	3617922	3704537	86616
DNAJC6	DnaJ heat shock protein family (Hsp40) member C6	1	65713902	65881552	167651	KANSL1	KAT8 regulatory NSL complex subunit 1	17	44107282	44302733	195452
**DRD4**	dopamine receptor D4	11	637293	640706	3414	KAT6B	lysine acetyltransferase 6B	10	76585340	76792380	207041
ECE1	endothelin converting enzyme 1	1	21543740	21671997	128258	KCNAB1	potassium voltage-gated channel subfamily A member regulatory beta subunit 1	3	155755490	156256545	501056
EDF1	endothelial differentiation related factor 1	9	139756571	139760738	4168	**KCNQ5**	potassium voltage-gated channel subfamily Q member 5	6	73331520	73908574	577055
**EEF1D**	eukaryotic translation elongation factor 1 delta	8	144661867	144681711	19845	KIF11	kinesin family member 11	10	94353043	94415150	62108
EIF4G3	eukaryotic translation initiation factor 4 gamma 3	1	21132963	21503377	370415	KIF2A	kinesin family member 2A	5	61601989	61833076	231088

ELK1	ELK1, ETS transcription factor	X	47494920	47510003	15084	LDLR	low density lipoprotein receptor	19	11200038	11244492	44455
FA2H	fatty acid 2-hydroxylase	16	74746853	74808729	61877	LEKR1	leucine, glutamate and lysine rich 1	3	156543270	156763918	220649
FAM50A	family with sequence similarity 50 member A	X	153672473	153679002	6530	MARVELD2	MARVEL domain containing 2	5	68710939	68740157	29219
FANCI	Fanconi anemia complementation group I	15	89787180	89860492	73313	MED14	mediator complex subunit 14	X	40507558	40595110	87553
**FBXO42**	F-box protein 42	1	16573334	16678949	105616	MGA	MGA, MAX dimerization protein	15	41913422	42062141	148720
FLNA	filamin A	X	153576892	153603006	26115	MIR1268A	microRNA 1268a	15	22513229	22513280	52
GAA	glucosidase alpha, acid	17	78075355	78093678	18324	MIR548F1	microRNA 548f-1	10	56367634	56367717	84
GAB3	GRB2 associated binding protein 3	X	153903529	153979858	76330	MKL1	megakaryoblastic leukemia (translocation) 1	22	40806285	41032706	226422
GABRB3	gamma-aminobutyric acid type A receptor beta3 subunit	15	26788693	27184686	395994	MKNK1	MAP kinase interacting serine/threonine kinase 1	1	47023090	47082515	59426
GABRD	gamma-aminobutyric acid type A receptor delta subunit	1	1950780	1962192	11413	MMP24	matrix metallopeptidase 24	20	33814457	33864801	50345
GAD1	glutamate decarboxylase 1	2	171669723	171717661	47939	MRPS27	mitochondrial ribosomal protein S27	5	71515236	71616473	101238
GAMT	guanidinoacetate N-methyltransferase	19	1397091	1401569	4479	**NAIP**	NLR family apoptosis inhibitory protein	5	70264310	70320941	56632
GATA2	GATA binding protein 2	3	128198270	128212028	13759	NDRG3	NDRG family member 3	20	35280169	35374481	94313
GATA2-AS1	GATA2 antisense RNA 1	3	128208036	128216768	8733	NDUFAF1	NADH:ubiquinone oxidoreductase complex assembly factor 1	15	41679551	41694717	15167
GDNF	glial cell derived neurotrophic factor	5	37812779	37839788	27010	NDUFV2	NADH:ubiquinone oxidoreductase core subunit V2	18	9102628	9134343	31716
GNA12	G protein subunit alpha 12	7	2767746	2883958	116213	NF1	neurofibromin 1	17	29421945	29709134	287190
GPSM3	G protein signaling modulator 3	6	32158543	32163300	4758	NFS1	NFS1, cysteine desulfurase	20	34255977	34287281	31305
GRIA3	glutamate ionotropic receptor AMPA type subunit 3	X	122318006	122624766	306761	**NIPA1**	non imprinted in Prader-Willi/Angelman syndrome 1	15	23043277	23100005	56729
GRIN1	glutamate ionotropic receptor NMDA type subunit 1	9	140032842	140063207	30366	NIPA2	non imprinted in Prader-Willi/Angelman syndrome 2	15	23004684	23034427	29744
GRIN2A	glutamate ionotropic receptor NMDA type subunit 2A	16	9852376	10276611	424236	NOP14	NOP14 nucleolar protein	4	2939660	2965112	25453
GRIN3B	glutamate ionotropic receptor NMDA type subunit 3B	19	1000418	1009731	9314	NR6A1	nuclear receptor subfamily 6 group A member 1	9	127279554	127533589	254036
GRK6	G protein-coupled receptor kinase 6	5	176830205	176869902	39698	NSF	N-ethylmaleimide sensitive factor, vesicle fusing ATPase	17	44668035	44834830	166796
**GSDMD**	gasdermin D	8	144635377	144645232	9856	NSUN4	NOP2/Sun RNA methyltransferase family member 4	1	46805849	46830824	24976
GSPT2	G1 to S phase transition 2	X	51486481	51489324	2844	NUCKS1	nuclear casein kinase and cyclin dependent kinase substrate 1	1	205681947	205719404	37458
GSTM3	glutathione S-transferase mu 3	1	110276554	110284384	7831	NUMB	NUMB, endocytic adaptor protein	14	73741815	73930348	188534
GSTT1	glutathione S-transferase theta 1	22	24376133	24384680	8548	NUSAP1	nucleolar and spindle associated protein 1	15	41624892	41673248	48357
HBB	hemoglobin subunit beta	11	5246694	5250625	3932	PAFAH1B1	platelet activating factor acetylhydrolase 1b regulatory subunit 1	17	2496504	2588909	92406
HDAC6	histone deacetylase 6	X	48659784	48683392	23609	**PELP1**	proline, glutamate and leucine rich protein 1	17	4574679	4607632	32954
HLA-A	major histocompatibility complex, class I, A	6	29909037	29913661	4625	PHF20	PHD finger protein 20	20	34359896	34538303	178408
HLA-DRB5	major histocompatibility complex, class II, DR beta 5	6	32485120	32498064	12945	PICALM	phosphatidylinositol binding clathrin assembly protein	11	85668727	85780924	112198
HS1BP3	HCLS1 binding protein 3	2	20760208	20850849	90642	PKIG	cAMP-dependent protein kinase inhibitor gamma	20	43160426	43252888	92463
HSF2BP	heat shock transcription factor 2 binding protein	21	44949072	45079374	130303	PLXNA2	plexin A2	1	208195587	208417665	222079
HSPG2	heparan sulfate proteoglycan 2	1	22148738	22263790	115053	POLN	DNA polymerase nu	4	2073645	2243848	170204
HTR6	5-hydroxytryptamine receptor 6	1	19991780	20006055	14276	POMGNT1	protein O-linked mannose N-acetylglucosaminyltransf erase 1 (beta 1,2-)	1	46654354	46685977	31624
IGF2	insulin like growth factor 2	11	2150342	2170833	20492	**PPP1R13B**	protein phosphatase 1 regulatory subunit 13B	14	104200089	104313927	113839
IGSF21	immunoglobin superfamily member 21	1	18434240	18704977	270738	PSMA4	proteasome subunit alpha 4	15	78832747	78841604	8858
INS-IGF2	INS-IGF2 readthrough	11	2153768	2182439	28672	PUS10	pseudouridylate synthase 10	2	61167357	61245394	78038

IQSEC2	IQ motif and Sec7 domain 2	X	53262058	53350522	88465	RABGAP1L	RAB GTPase activating protein 1 like	1	174128548	174964445	835898
ITGB2	integrin subunit beta 2	21	46305868	46351904	46037	RAP1GAP2	RAP1 GTPase activating protein 2	17	2680350	2941033	260684
IZUMO2	IZUMO family member 2	19	50655805	50666452	10648	REL	REL proto-oncogene, NF-kB subunit	2	61108656	61158745	50090
KCNC3	potassium voltage-gated channel subfamily C member 3	19	50815194	50836772	21579	RGS12	regulator of G protein signaling 12	4	3294755	3441640	146886
KCNJ11	potassium voltage-gated channel subfamily J member 11	11	17407406	17410878	3473	RIMS4	regulating synaptic membrane exocytosis 4	20	43380449	43438979	58531
KCNQ1	potassium voltage-gated channel subfamily Q member 1	11	2465914	2870339	404426	RNU6-83P	RNA, U6 small nuclear 83, pseudogene	13	99677488	99677599	112
KCNQ2	potassium voltage-gated channel subfamily Q member 2	20	62037542	62103993	66452	RTN3	reticulon 3	11	63448918	63527363	78446
KCNT1	potassium sodium-activated channel subfamily T member 1	9	138594031	138684992	90962	SAMHD1	SAM and HD domain containing deoxynucleoside triphosphate triphosphohydrolase 1	20	35518632	35580246	61615
KDM5C	lysine demethylase 5C	X	53220503	53254604	34102	SCAI	suppressor of cancer cell invasion	9	127704887	127905785	200899
KDM5D	lysine demethylase 5D	Y	21865751	21906825	41075	**SETX**	senataxin	9	135136743	135230372	93630
KIF1A	kinesin family member 1A	2	241653181	241759725	106545	SIPA1L3	signal induced proliferation associated 1 like 3	19	38397868	38699012	301145
KIF7	kinesin family member 7	15	90152020	90198682	46663	SLC17A5	solute carrier family 17 member 5	6	74303102	74363878	60777
KRTAP5-AS1	KRTAP5-1/KRTAP5-2 antisense RNA 1	11	1592583	1620414	27832	SLC30A6	solute carrier family 30 member 6	2	32390933	32449448	58516
L1CAM	L1 cell adhesion molecule	X	153126969	153174677	47709	SLC33A1	solute carrier family 33 member 1	3	155538813	155572218	33406
LDLRAD2	low density lipoprotein receptor class A domain containing 2	1	22138758	22151714	12957	**SMN1**	survival of motor neuron 1, telomeric	5	70220768	70249769	29002
LIMCH1	LIM and calponin homology domains 1	4	41361624	41702061	340438	SMYD3	SET and MYND domain containing 3	1	245912642	246670614	757973
LIMS2	LIM zinc finger domain containing 2	2	128395956	128439360	43405	SOS1	SOS Ras/Rac guanine nucleotide exchange factor 1	2	39208537	39351486	142950
LINC01284	long intergenic non-protein coding RNA 1284	X	51095844	51171400	75557	SOS2	SOS Ras/Rho guanine nucleotide exchange factor 2	14	50583847	50698276	114430
LMNA	lamin A/C	1	156052364	156109880	57517	**SPAST**	spastin	2	32288680	32382706	94027
LMX1A	LIM homeobox transcription factor 1 alpha	1	165171104	165325952	154849	**SPG11**	SPG11, spatacsin vesicle trafficking associated	15	44854894	44955876	100983
LMX1B	LIM homeobox transcription factor 1 beta	9	129376722	129463311	86590	SPG21	SPG21, maspardin	15	65255362	65282648	27287
**LOX**	lysyl oxidase	5	121398890	121413980	15091	SREBF2	sterol regulatory element binding transcription factor 2	22	42229109	42303312	74204
LRP1	LDL receptor related protein 1	12	57522276	57607134	84859	ST3GAL3	ST3 beta-galactoside alpha-2,3-sialyltransferase 3	1	44171495	44396831	225337
MAN1B1	mannosidase alpha class 1B member 1	9	139981379	140003635	22257	STIL	SCL/TAL1 interrupting locus	1	47715811	47779819	64009
MAPK8IP1	mitogen-activated protein kinase 8 interacting protein 1	11	45907202	45928016	20815	STK4	serine/threonine kinase 4	20	43595115	43708600	113486
MECP2	methyl-CpG binding protein 2	X	153287024	153363212	76189	STRBP	spermatid perinuclear RNA binding protein	9	125871779	126030855	159077
MIR1-1HG	MIR1-1 host gene	20	61147660	61167971	20312	SUZ12	SUZ12 polycomb repressive complex 2 subunit	17	30264037	30328064	64028
MIR1268A	microRNA 1268a	15	22513229	22513280	52	SYT10	synaptotagmin 10	12	33527173	33592754	65582
MMEL1	membrane metalloendopeptidase like 1	1	2522078	2564481	42404	**TAF15**	TATA-box binding protein associated factor 15	17	34136459	34191619	55161
MORN1	MORN repeat containing 1	1	2252692	2323146	70455	TDRD9	tudor domain containing 9	14	104394799	104519004	124206
MPO	myeloperoxidase	17	56347217	56358296	11080	TEF	TEF, PAR bZIP transcription factor	22	41763337	41795330	31994
MVB12B	multivesicular body subunit 12B	9	129089128	129269320	180193	TET1	tet methylcytosine dioxygenase 1	10	70320413	70454239	133827
MYH14	myosin heavy chain 14	19	50691443	50813802	122360	TFB2M	transcription factor B2, mitochondrial	1	246703862	246729626	25765
NAP1L4	nucleosome assembly protein 1 like 4	11	2965667	3013607	47941	TIPARP	TCDD inducible poly(ADP-ribose) polymerase	3	156391024	156424559	33536
NDUFA6	NADH:ubiquinone oxidoreductase subunit A6	22	42481529	42486959	5431	TLDC2	TBC/LysM-associated domain containing 2	20	35504524	35522638	18115
NDUFS7	NADH:ubiquinone oxidoreductase core subunit S7	19	1383526	1395583	12058	TMEM17	transmembrane protein 17	2	62727356	62739029	11674
NELFE	negative elongation factor complex member E	6	31919864	31926887	7024	TMEM67	transmembrane protein 67	8	94767072	94831462	64391
NFKB1	nuclear factor kappa B subunit 1	4	103422486	103538459	115974	TRIP4	thyroid hormone receptor interactor 4	15	64679947	64747502	67556

NOP56	NOP56 ribonucleoprotein	20	2632791	2639039	6249
NOTCH4	notch 4	6	32162620	32191844	29225
NPHP4	nephrocystin 4	1	5922871	6052533	129663
NR1H2	nuclear receptor subfamily 1 group H member 2	19	50832910	50886239	53330
NTRK1	neurotrophic receptor tyrosine kinase 1	1	156785432	156851642	66211
NTRK2	neurotrophic receptor tyrosine kinase 2	9	87283466	87638505	355040
NUDT1	nudix hydrolase 1	7	2281857	2290781	8925
PANK2	pantothenate kinase 2	20	3869486	3907605	38120
PCDH11Y	protocadherin 11 Y-linked	Y	4868267	5610265	741999
PCSK9	proprotein convertase subtilisin/kexin type 9	1	55505221	55530525	25305
PDXK	pyridoxal kinase	21	45138975	45182188	43214
PDYN	prodynorphin	20	1959403	1974732	15330
PEX10	peroxisomal biogenesis factor 10	1	2336236	2345236	9001
PHOX2B	paired like homeobox 2b	4	41746099	41750987	4889
PIK3C2B	phosphatidylinositol-4-phosphate 3-kinase catalytic subunit type 2 beta	1	204391756	204463852	72097
PIK3R5	phosphoinositide-3-kinase regulatory subunit 5	17	8782233	8869029	86797
PINK1	PTEN induced putative kinase 1	1	20959948	20978004	18057
PINK1-AS	PINK1 antisense RNA	1	20969150	20978686	9537
PKD1	polycystin 1, transient receptor potential channel interacting	16	2138711	2185899	47189
PLAT	plasminogen activator, tissue type	8	42032236	42065242	33007
PLEC	plectin	8	144989321	145050902	61582
**PLEKHG5**	pleckstrin homology and RhoGEF domain containing G5	1	6526152	6580121	53970
PLXNA2	plexin A2	1	208195587	208417665	222079
PNKP	polynucleotide kinase 3'-phosphatase	19	50364461	50371166	6706
POLG	DNA polymerase gamma, catalytic subunit	15	89859534	89878092	18559
PORCN	porcupine homolog (Drosophila)	X	48367350	48379202	11853
PPP1R3F	protein phosphatase 1 regulatory subunit 3F	X	49126306	49157929	31624
PPP3CA	protein phosphatase 3 catalytic subunit alpha	4	101944566	102269435	324870
PRDM16	PR/SET domain 16	1	2985732	3355185	369454
PRKCZ	protein kinase C zeta	1	1981909	2116834	134926
PRKY	protein kinase, Y-linked, pseudogene	Y	7142013	7249589	107577
PRODH	proline dehydrogenase 1	22	18900294	18924066	23773
PSEN2	presenilin 2	1	227057885	227083806	25922
PTTG1IP	PTTG1 interacting protein	21	46269500	46293752	24253
RAC2	ras-related C3 botulinum toxin substrate 2 (rho family, small GTP binding protein Rac2)	22	37621301	37640488	19188
RBFOX1	RNA binding protein, fox-1 homolog 1	16	6069095	7763340	1694246
RBFOX3	RNA binding protein, fox-1 homolog 3	17	77085427	77613550	528124
RCC2	regulator of chromosome condensation 2	1	17733256	17766220	32965
REEP2	receptor accessory protein 2	5	137774706	137782658	7953
REN	renin	1	204123944	204135465	11522
RER1	retention in endoplasmic reticulum sorting receptor 1	1	2323267	2336883	13617
RHCG	Rh family C glycoprotein	15	89998680	90039844	41165
RNASEH2B-AS1	RNASEH2B antisense RNA 1	13	51450822	51484848	34027
RRP8	ribosomal RNA processing 8, methyltransferase, homolog (yeast)	11	6616305	6624850	8546
RXRA	retinoid X receptor alpha	9	137208944	137332431	123488
**TRPM7**	transient receptor potential cation channel subfamily M member 7	15	50844670	50979012	134343
UBE3C	ubiquitin protein ligase E3C	7	156931607	157062066	130460
ULK2	unc-51 like autophagy activating kinase 2	17	19674142	19771249	97108
UQCC1	ubiquinol-cytochrome c reductase complex assembly factor 1	20	33890369	33999944	109576
USP34	ubiquitin specific peptidase 34	2	61414598	61697904	283307
UTP6	UTP6, small subunit processome component	17	30187923	30228784	40862
VPS35	VPS35, retromer complex component	16	46690054	46723430	33377
**VPS54**	VPS54, GARP complex subunit	2	64119280	64246206	126927
WDPCP	WD repeat containing planar cell polarity effector	2	63348518	64054977	706460
XPNPEP3	X-prolyl aminopeptidase 3	22	41253081	41363838	110758
ZBTB37	zinc finger and BTB domain containing 37	1	173837220	173872687	35468
ZFYVE28	zinc finger FYVE-type containing 28	4	2271309	2420390	149082
ZNF567	zinc finger protein 567	19	37178514	37218603	40090
ZNF585A	zinc finger protein 585A	19	37597636	37663643	66008

RXRG	retinoid X receptor gamma	1	165370159	165414433	44275
**SARM1**	sterile alpha and TIR motif containing 1	17	26691378	26728065	36688
SBNO2	strawberry notch homolog 2	19	1107636	1174282	66647
SCARB1	scavenger receptor class B member 1	12	125261402	125367214	105813
**SDK1**	sidekick cell adhesion molecule 1	7	3341080	4308632	967553
SEC16A	SEC16 homolog A, endoplasmic reticulum export factor	9	139334549	139372141	37593
SEPT3	septin 3	22	42372276	42394225	21950
SEPT9	septin 9	17	75276651	75496678	220028
SERPINF2	serpin family F member 2	17	1646130	1658562	12433
SETD3	SET domain containing 3	14	99864083	99947216	83134
SH2D2A	SH2 domain containing 2A	1	156776035	156786654	10620
SHROOM4	shroom family member 4	X	50334647	50557302	222656
SLC25A22	solute carrier family 25 member 22	11	790475	798316	7842
SLC35A2	solute carrier family 35 member A2	X	48760459	48769235	8777
SLC37A1	solute carrier family 37 member 1	21	43916118	44001550	85433
SLIT3	slit guidance ligand 3	5	168088745	168728133	639389
SNCAIP	synuclein alpha interacting protein	5	121647049	121799914	152866
SPECC1L	sperm antigen with calponin homology and coiled-coil domains 1 like	22	24666786	24813708	146923
SPECC1L-ADORA2A	SPECC1L-ADORA2A readthrough (NMD candidate)	22	24666866	24838324	171459
SREBF1	sterol regulatory element binding transcription factor 1	17	17713713	17740325	26613
**SS18L1**	SS18L1, nBAF chromatin remodeling complex subunit	20	60718822	60757540	38719
SSX5	SSX family member 5	X	48045656	48056199	10544
STIM1	stromal interaction molecule 1	11	3875757	4114439	238683
STK11	serine/threonine kinase 11	19	1189406	1228428	39023
SYN1	synapsin I	X	47431303	47479252	47950
**SYT9**	synaptotagmin 9	11	7260009	7490273	230265
TBC1D16	TBC1 domain family member 16	17	77906142	78009647	103506
TBL1Y	transducin beta like 1, Y-linked	Y	6778727	6959724	180998
TGM6	transglutaminase 6	20	2361554	2413399	51846
TH	tyrosine hydroxylase	11	2185159	2193107	7949
**TIMP2**	TIMP metallopeptidase inhibitor 2	17	76849059	76921469	72411
TNRC6C	trinucleotide repeat containing 6C	17	76000249	76104916	104668
TOM1L2	target of myb1 like 2 membrane trafficking protein	17	17746828	17875736	128909
TP73	tumor protein p73	1	3569084	3652765	83682
TPP1	tripeptidyl peptidase 1	11	6634000	6640692	6693
TRAF2	TNF receptor associated factor 2	9	139776364	139821059	44696
TRIM5	tripartite motif containing 5	11	5684425	5959849	275425
**TRPM7**	transient receptor potential cation channel subfamily M member 7	15	50844670	50979012	134343
TSC1	tuberous sclerosis 1	9	135766735	135820020	53286
TSC2	tuberous sclerosis 2	16	2097466	2138716	41251
TSPEAR	thrombospondin type laminin G domain and EAR repeats	21	45917775	46131495	213721
TTTY10	testis-specific transcript, Y-linked 10 (non-protein coding)	Y	22627554	22681114	53561
TTTY14	testis-specific transcript, Y-linked 14 (non-protein coding)	Y	21034387	21239302	204916
UBASH3A	ubiquitin associated and SH3 domain containing A	21	43824008	43867791	43784
**UBE2I**	ubiquitin conjugating enzyme E2 I	16	1355548	1377019	21472
UBR4	ubiquitin protein ligase E3 component n-recognin 4	1	19401000	19536770	135771
UCHL1	ubiquitin C-terminal hydrolase L1	4	41258430	41270472	12043
UNKL	unkempt family like zinc finger	16	1413206	1464752	51547

USP48	ubiquitin specific peptidase 48	1	22004791	22110099	105309
**VCP**	valosin containing protein	9	35056061	35073246	17186
VWA5B1	von Willebrand factor A domain containing 5B1	1	20617412	20681387	63976
WDR13	WD repeat domain 13	X	48448430	48463581	15152
WDR45	WD repeat domain 45	X	48929385	48958108	28724
WWC1	WW and C2 domain containing 1	5	167718656	167899308	180653
YTHDF1	YTH N6-methyladenosine RNA binding protein 1	20	61826781	61847586	20806
ZBTB46	zinc finger and BTB domain containing 46	20	62375019	62462597	87579
ZFYVE27	zinc finger FYVE-type containing 27	10	99496878	99520664	23787
ZNF182	zinc finger protein 182	X	47834250	47863377	29128
**ZNF512B**	zinc finger protein 512B	20	62588055	62680113	92059

Supplementary Table 8. List of genes located in the most recurrent CNVs of SALS1 patients (pentrance > 10%).

Duplications

Gene name	Gene description	Chromosome	Gene start (bp)	Gene end (bp)	Size (bp)	Log2Ratio (Mean)
ABHD2	abhydrolase domain containing 2	15	89630690	89745591	114902	0.723228
ACAN	aggrecan	15	89346674	89418585	71912	0.723228
ACHE	acetylcholinesterase (Cartwright blood group)	7	100487615	100494594	6980	0.7050535
ADGRG1	adhesion G protein-coupled receptor G1	16	57653442	57698944	45503	0.701157
AGER	advanced glycosylation end-product specific receptor	6	32148745	32152101	3357	0.618968
AKT1	AKT serine/threonine kinase 1	14	105235686	105262088	26403	0.660989
ALDH4A1	aldehyde dehydrogenase 4 family member A1	1	19197926	19229275	31350	0.641693
ALPL	alkaline phosphatase, liver/bone/kidney	1	21835858	21904905	69048	0.641693
AP4S1	adaptor related protein complex 4 sigma 1 subunit	14	31494312	31562818	68507	1.361322
AP5Z1	adaptor related protein complex 5 zeta 1 subunit	7	4815253	4833943	18691	0.718
ATP13A2	ATPase 13A2	1	17312453	17338423	25971	0.641693
ATPAF2	ATP synthase mitochondrial F1 complex assembly factor 2	17	17880723	17942523	61801	0.73833333
BIN1	bridging integrator 1	2	127805603	127864931	59329	0.69319
C1QTNF1	C1q and TNF related 1	17	77018896	77045870	26975	0.596693
C1QTNF1-AS1	C1QTNF1 antisense RNA 1	17	77015291	77023737	8447	0.596693
C4A	complement C4A (Rodgers blood group)	6	31949801	31970458	20658	0.618968
CABIN1	calcineurin binding protein 1	22	24407642	24574596	166955	0.575321
CAPN3	calpain 3	15	42640301	42704516	64216	0.637835
CCL3	C-C motif chemokine ligand 3	17	34415602	34417515	1914	0.648784
CCL5	C-C motif chemokine ligand 5	17	34198495	34207797	9303	0.648784
CD24	CD24 molecule	6	107417708	107422630	4923	1.08
CDA	cytidine deaminase	1	20915441	20945401	29961	0.641693
CEP104	centrosomal protein 104	1	3728645	3773778	45134	0.526325
CEP164	centrosomal protein 164	11	117185273	117283984	98712	0.541087
CHD5	chromodomain helicase DNA binding protein 5	1	6161853	6240183	78331	0.526325
CHRNA4	cholinergic receptor nicotinic alpha 4 subunit	20	61975420	62009753	34334	0.625377
CLCNKA	chloride voltage-gated channel Ka	1	16345370	16360545	15176	0.641693
CLCNKB	chloride voltage-gated channel Kb	1	16370272	16383803	13532	0.641693
CLN3	CLN3, battenin	16	28477983	28506896	28914	0.701157
CLTCL1	clathrin heavy chain like 1	22	19166986	19279239	112254	0.575321
CNTN2	contactin 2	1	205012325	205047627	35303	0.332593
**CST3**	cystatin C	20	23608534	23619110	10577	0.625377
CYTH1	cytohesin 1	17	76670130	76778379	108250	0.596693
**DCTN1**	dynactin subunit 1	2	74588281	74619214	30934	0.5347745
DDOST	dolichyl-diphosphooligosaccharide--protein glycosyltransferase non-catalytic subunit	1	20978270	20988000	9731	0.641693
DHCR24	24-dehydrocholesterol reductase	1	55315306	55352891	37586	0.883039
DNAH17	dynein axonemal heavy chain 17	17	76419778	76573476	153699	0.596693
ECE1	endothelin converting enzyme 1	1	21543740	21671997	128258	0.641693
**EEF1D**	eukaryotic translation elongation factor 1 delta	8	144661867	144681711	19845	0.557456
EIF4G3	eukaryotic translation initiation factor 4 gamma 3	1	21132963	21503377	370415	0.641693
F8	coagulation factor VIII	X	154064063	154255215	191153	0.533195
FAM50A	family with sequence similarity 50 member A	X	153672473	153679002	6530	0.533195

Deletions

Gene name	Gene description	Chromosome	Gene start (bp)	Gene end (bp)	Size (bp)	Log2Ratio (Mean)
ACE	angiotensin I converting enzyme	17	61554422	61599205	44784	-1.544
ADAM17	ADAM metallopeptidase domain 17	2	9628615	9695921	67307	-0.526407
ADAMTS6	ADAM metallopeptidase with thrombospondin type 1 motif 6	5	64444563	64777747	333185	-0.525298
**ADK**	adenosine kinase	10	75910960	76469061	558102	-0.53911
ADSL	adenylosuccinate lyase	22	40742507	40786467	43961	-1.262164
AHCY	adenosylhomocysteinase	20	32868074	32899608	31535	-0.559453
ALG6	ALG6, alpha-1,3-glucosyltransferase	1	63833261	63904233	70973	-0.663621
AP4E1	adaptor related protein complex 4 epsilon 1 subunit	15	51200869	51298097	97229	-0.607008
APH1B	aph-1 homolog B, gamma-secretase subunit	15	63568217	63601325	33109	-0.541159
APOM	apolipoprotein M	6	31620193	31625987	5795	-0.297285
**ARHGEF28**	Rho guanine nucleotide exchange factor 28	5	72921983	73237818	315836	-0.773799
ARSB	arylsulfatase B	5	78073032	78281910	208879	-0.773799
ATP6AP2	ATPase H+ transporting accessory protein 2	X	40440146	40465889	25744	-0.67002
**ATXN2**	ataxin 2	12	111890018	112037480	147463	-0.484845
ATXN3	ataxin 3	14	92524896	92572965	48070	-0.654345
B3GNT2	UDP-GlcNAc:betaGal beta-1,3-N-acetylglucosaminyltransferase 2	2	62423248	62451866	28619	-0.682618
BDP1	B double prime 1, subunit of RNA polymerase III transcription initiation factor IIIB	5	70751442	70863649	112208	-0.773799
BIRC6	baculoviral IAP repeat containing 6	2	32582096	32843966	261871	-0.634216
CA12	carbonic anhydrase 12	15	63613577	63674360	60784	-0.624229
CASK	calcium/calmodulin dependent serine protein kinase	X	41374187	41782716	408530	-0.67002
CBFA2T2	CBFA2/RUNX1 translocation partner 2	20	32077881	32237842	159962	-0.559453
CBS	cystathionine-beta-synthase	21	44473301	44497053	23753	-0.956621
CCDC144A	coiled-coil domain containing 144A	17	16592851	16707767	114917	-0.543799
CCT4	chaperonin containing TCP1 subunit 4	2	62095224	62115939	20716	-0.682618
CHD2	chromodomain helicase DNA binding protein 2	15	93426526	93571237	144712	-0.624229
CHMP4B	charged multivesicular body protein 4B	20	32399110	32442172	43063	-0.559453
CHP1	calcineurin like EF-hand protein 1	15	41523037	41574043	51007	-0.624229
CNTNAP3	contactin associated protein-like 3	9	39072764	39288312	215549	-1.295
COG4	component of oligomeric golgi complex 4	16	70514471	70557468	42998	-0.657947
COQ2	coenzyme Q2, polyprenyltransferase	4	84182689	84206067	23379	-0.608374
COQ6	coenzyme Q6, monooxygenase	14	74416629	74430373	13745	-0.531832
CR1	complement C3b/C4b receptor 1 (Knops blood group)	1	207669492	207813992	144501	-0.677425
CRLF3	cytokine receptor like factor 3	17	29096406	29151794	55389	-0.956621
CSNK1G1	casein kinase 1 gamma 1	15	64457716	64648442	190727	-0.624229
CWC27	CWC27 spliceosome associated protein homolog	5	64064757	64314590	249834	-0.525298
CYP2C19	cytochrome P450 family 2 subfamily C member 19	10	96447911	96613017	165107	-0.952624
DAPK2	death associated protein kinase 2	15	64199235	64364232	164998	-0.624229
DARS2	aspartyl-tRNA synthetase 2, mitochondrial	1	173793641	173827684	34044	-0.584528
DDX3X	DEAD-box helicase 3, X-linked	X	41192651	41223725	31075	-0.67002
DENND1A	DENN domain containing 1A	9	126141933	126692431	550499	-0.605549
DEPDC5	DEP domain containing 5	22	32149944	32303012	153069	-0.591894
DLGAP4	DLG associated protein 4	20	34894258	35157040	262783	-0.737749

FANCI	Fanconi anemia complementation group I	15	89787180	89860492	73313	0.723228
**FBXO42**	F-box protein 42	1	16573334	16678949	105616	0.641693
FLNA	filamin A	X	153576892	153603006	26115	0.5389445
GAA	glucosidase alpha, acid	17	78075355	78093678	18324	0.596693
GAB3	GRB2 associated binding protein 3	X	153903529	153979858	76330	0.533195
GDNF	glial cell derived neurotrophic factor	5	37812779	37839788	27010	1.336
GRIA3	glutamate ionotropic receptor AMPA type subunit 3	X	122318006	122624766	306761	0.71429825
GRIN2A	glutamate ionotropic receptor NMDA type subunit 2A	16	9852376	10276611	424236	0.545872
GRK6	G protein-coupled receptor kinase 6	5	176830205	176869902	39698	0.625797
**GSDMD**	gasdermin D	8	144635377	144645232	9856	0.557456
GSTT1	glutathione S-transferase theta 1	22	24376133	24384680	8548	1.1011605
HIRA	histone cell cycle regulator	22	19318221	19435224	117004	0.575321
HLA-DRB5	major histocompatibility complex, class II, DR beta 5	6	32485120	32498064	12945	0.309484
HS1BP3	HCLS1 binding protein 3	2	20760208	20850849	90642	0.10316133
HSPG2	heparan sulfate proteoglycan 2	1	22148738	22263790	115053	0.641693
HTR6	5-hydroxytryptamine receptor 6	1	19991780	20006055	14276	0.641693
IGF1	insulin like growth factor 1	12	102789645	102874423	84779	1.068
IGF2	insulin like growth factor 2	11	2150342	2170833	20492	0.541087
IGSF21	immunoglobin superfamily member 21	1	18434240	18704977	270738	0.641693
KCNC3	potassium voltage-gated channel subfamily C member 3	19	50815194	50836772	21579	0.731731
KCNQ2	potassium voltage-gated channel subfamily Q member 2	20	62037542	62103993	66452	0.625377
KCNT1	potassium sodium-activated channel subfamily T member 1	9	138594031	138684992	90962	0.9877315
KDM5D	lysine demethylase 5D	Y	21865751	21906825	41075	1.08
KIF1A	kinesin family member 1A	2	241653181	241759725	106545	0.6180155
KIF7	kinesin family member 7	15	90152020	90198682	46663	0.723228
L1CAM	L1 cell adhesion molecule	X	153126969	153174677	47709	0.5389445
LDLRAD2	low density lipoprotein receptor class A domain containing 2	1	22138758	22151714	12957	0.641693
LIMCH1	LIM and calponin homology domains 1	4	41361624	41702061	340438	0.894707
LIMS2	LIM zinc finger domain containing 2	2	128395956	128439360	43405	0.69319
LMX1A	LIM homeobox transcription factor 1 alpha	1	165171104	165325952	154849	0.641693
LMX1B	LIM homeobox transcription factor 1 beta	9	129376722	129463311	86590	0.711392
LRP1	LDL receptor related protein 1	12	57522276	57607134	84859	0.634824
MC1R	melanocortin 1 receptor	16	89978527	89987385	8859	0.545872
MECP2	methyl-CpG binding protein 2	X	153287024	153363212	76189	0.5389445
MIR1268A	microRNA 1268a	15	22513229	22513280	52	
MMEL1	membrane metalloendopeptidase like 1	1	2522078	2564481	42404	0.526325
MPO	myeloperoxidase	17	56347217	56358296	11080	0.557864
MVB12B	multivesicular body subunit 12B	9	129089128	129269320	180193	0.835427
MYH14	myosin heavy chain 14	19	50691443	50813802	122360	0.731731
NELFE	negative elongation factor complex member E	6	31919864	31926887	7024	0.618968
NPHP4	nephrocystin 4	1	5922871	6052533	129663	0.526325
NTRK2	neurotrophic receptor tyrosine kinase 2	9	87283466	87638505	355040	0.835427
PCDH11Y	protocadherin 11 Y-linked	Y	4868267	5610265	741999	1.517
PCSK9	proprotein convertase subtilisin/kexin type 9	1	55505221	55530525	25305	0.883039
PDYN	prodynorphin	20	1959403	1974732	15330	0.625377
PEX10	peroxisomal biogenesis factor 10	1	2336236	2345236	9001	0.526325
PHOX2B	paired like homeobox 2b	4	41746099	41750987	4889	0.894707

DNAJB6	DnaJ heat shock protein family (Hsp40) member B6	7	157128075	157210133	82059	-0.562186
DPY30	dpy-30, histone methyltransferase complex regulatory subunit	2	32092878	32264881	172004	-0.682618
EHBP1	EH domain binding protein 1	2	62900986	63273622	372637	-0.682618
EHMT2	euchromatic histone lysine methyltransferase 2	6	31847536	31865464	17929	-0.594569
EPB41L1	erythrocyte membrane protein band 4.1 like 1	20	34679426	34820721	141296	-0.737749
**EPHA4**	EPH receptor A4	2	222282747	222438922	156176	-1.037
FAM126A	family with sequence similarity 126 member A	7	22980878	23053749	72872	-0.598219
FAM161B	family with sequence similarity 161 member B	14	74398204	74417117	18914	-0.531832
FAM193A	family with sequence similarity 193 member A	4	2626988	2734292	107305	-0.708963
FBLN5	fibulin 5	14	92335756	92414331	78576	-0.682712
FCHO2	FCH domain only 2	5	72251808	72386349	134542	-0.618199
FGD4	FYVE, RhoGEF and PH domain containing 4	12	32552463	32798984	246522	-0.961062
FGFR3	fibroblast growth factor receptor 3	4	1795034	1810599	15566	-0.708963
FKTN	fukutin	9	108320411	108403399	82989	-0.758742
GRK5	G protein-coupled receptor kinase 5	10	120967101	121215131	248031	-1.3632
GUSBP3	glucuronidase, beta pseudogene 3	5	68790040	69006341	216302	-0.618199
HERC1	HECT and RLD domain containing E3 ubiquitin protein ligase family member 1	15	63900817	64126141	225325	-0.624229
HLA-DRA	major histocompatibility complex, class II, DR alpha	6	32407619	32412823	5205	-0.40774
HLA-DRB5	major histocompatibility complex, class II, DR beta 5	6	32485120	32498064	12945	-0.91158
HSPA1A	heat shock protein family A (Hsp70) member 1A	6	31783291	31785723	2433	-0.696205
HSPA1B	heat shock protein family A (Hsp70) member 1B	6	31795512	31798031	2520	-0.696205
HSPA1L	heat shock protein family A (Hsp70) member 1 like	6	31777396	31783437	6042	-0.696205
HSPA5	heat shock protein family A (Hsp70) member 5	9	127997132	128003609	6478	-0.758742
HTT	huntingtin	4	3076408	3245676	169269	-0.708963
HYKK	hydroxylysine kinase	15	78799906	78829714	29809	-0.677136
IDE	insulin degrading enzyme	10	94211441	94333833	122393	-0.60181
IL6	interleukin 6	7	22765503	22771621	6119	-0.598219
IPO11	importin 11	5	61699799	61924409	224611	-0.525298
IREB2	iron responsive element binding protein 2	15	78729773	78793798	64026	-0.677136
ITGAE	integrin subunit alpha E	17	3617922	3704537	86616	-0.551457
KANSL1	KAT8 regulatory NSL complex subunit 1	17	44107282	44302733	195452	-0.551457
KAT6B	lysine acetyltransferase 6B	10	76585340	76792380	207041	-0.60181
KIF11	kinesin family member 11	10	94353043	94415150	62108	-0.60181
KIF1B	kinesin family member 1B	1	10270863	10441661	170799	-0.76503
KIF2A	kinesin family member 2A	5	61601989	61833076	231088	-0.525298
L2HGDH	L-2-hydroxyglutarate dehydrogenase	14	50704281	50779266	74986	-0.586852
LDLR	low density lipoprotein receptor	19	11200038	11244492	44455	-0.57591
MARVELD2	MARVEL domain containing 2	5	68710939	68740157	29219	-0.618199
MED14	mediator complex subunit 14	X	40507558	40595110	87553	-0.67002
MGA	MGA, MAX dimerization protein	15	41913422	42062141	148720	-0.645528
MKL1	megakaryoblastic leukemia (translocation) 1	22	40806285	41032706	226422	-0.585327
MKNK1	MAP kinase interacting serine/threonine kinase 1	1	47023090	47082515	59426	-0.687345
MMP24	matrix metallopeptidase 24	20	33814457	33864801	50345	-0.727813
MPRIP	myosin phosphatase Rho interacting protein	17	16945859	17120993	175135	-0.543799
MRPS27	mitochondrial ribosomal protein S27	5	71515236	71616473	101238	-0.773799
MYH7B	myosin heavy chain 7B	20	33563206	33590240	27035	-0.559453
**NAIP**	NLR family apoptosis inhibitory protein	5	70264310	70320941	56632	-0.773799

PIK3C2B	phosphatidylinositol-4-phosphate 3-kinase catalytic subunit type 2 beta	1	204391756	204463852	72097	0.665186
PIK3R5	phosphoinositide-3-kinase regulatory subunit 5	17	8782233	8869029	86797	0.596693
PINK1	PTEN induced putative kinase 1	1	20959948	20978004	18057	0.641693
PINK1-AS	PINK1 antisense RNA	1	20969150	20978686	9537	0.641693
PLAT	plasminogen activator, tissue type	8	42032236	42065242	33007	0.603083
PLEC	plectin	8	144989321	145050902	61582	0.557456
**PLEKHG5**	pleckstrin homology and RhoGEF domain containing G5	1	6526152	6580121	53970	0.526325
PLXNA2	plexin A2	1	208195587	208417665	222079	0.6498045
POLG	DNA polymerase gamma, catalytic subunit	15	89859534	89878092	18559	0.723228
**PON1**	paraoxonase 1	7	94926988	95025673	98686	0.7050535
PPP3CA	protein phosphatase 3 catalytic subunit alpha	4	101944566	102269435	324870	0.894707
PRDM16	PR/SET domain 16	1	2985732	3355185	369454	0.526325
PRKY	protein kinase, Y-linked, pseudogene	Y	7142013	7249589	107577	1.517
PRODH	proline dehydrogenase 1	22	18900294	18924066	23773	0.575321
RAB39B	RAB39B, member RAS oncogene family	X	154487526	154493874	6349	0.533195
RAC2	ras-related C3 botulinum toxin substrate 2 (rho family, small GTP binding protein Rac2)	22	37621301	37640488	19188	0.575321
RBFOX3	RNA binding protein, fox-1 homolog 3	17	77085427	77613550	528124	0.93128
RCC2	regulator of chromosome condensation 2	1	17733256	17766220	32965	0.641693
REEP2	receptor accessory protein 2	5	137774706	137782658	7953	0.630557
REN	renin	1	204123944	204135465	11522	0.665186
RHCG	Rh family C glycoprotein	15	89998680	90039844	41165	0.723228
RXRG	retinoid X receptor gamma	1	165370159	165414433	44275	
SCN8A	sodium voltage-gated channel alpha subunit 8	12	51984050	52206648	222599	0.898473
SEPT9	septin 9	17	75276651	75496678	220028	0.596693
SLIT3	slit guidance ligand 3	5	168088745	168728133	639389	0.630557
SPECC1L	sperm antigen with calponin homology and coiled-coil domains 1 like	22	24666786	24813708	146923	0.575321
SPECC1L-ADORA2A	SPECC1L-ADORA2A readthrough (NMD candidate)	22	24666866	24838324	171459	0.575321
SREBF1	sterol regulatory element binding transcription factor 1	17	17713713	17740325	26613	0.93128
**SS18L1**	SS18L1, nBAF chromatin remodeling complex subunit	20	60718822	60757540	38719	0.625377
**SYNE1**	spectrin repeat containing nuclear envelope protein 1	6	152442819	152958936	516118	0.618968
**TAF15**	TATA-box binding protein associated factor 15	17	34136459	34191619	55161	0.324392
TBC1D16	TBC1 domain family member 16	17	77906142	78009647	103506	0.596693
TBL1Y	transducin beta like 1, Y-linked	Y	6778727	6959724	180998	1.517
TGM6	transglutaminase 6	20	2361554	2413399	51846	0.625377
**TIMP2**	TIMP metallopeptidase inhibitor 2	17	76849059	76921469	72411	0.596693
TNRC6C	trinucleotide repeat containing 6C	17	76000249	76104916	104668	0.596693
TOM1L2	target of myb1 like 2 membrane trafficking protein	17	17746828	17875736	128909	0.596693
TP73	tumor protein p73	1	3569084	3652765	83682	0.526325
TTTY10	testis-specific transcript, Y-linked 10 (non-protein coding)	Y	22627554	22681114	53561	1.08
TTTY14	testis-specific transcript, Y-linked 14 (non-protein coding)	Y	21034387	21239302	204916	1.08
TUBB3	tubulin beta 3 class III	16	89987800	90005169	17370	0.545872

NDRG3	NDRG family member 3	20	35280169	35374481	94313	-0.737749
NDUFAF1	NADH:ubiquinone oxidoreductase complex assembly factor 1	15	41679551	41694717	15167	-0.645528
NDUFV2	NADH:ubiquinone oxidoreductase core subunit V2	18	9102628	9134343	31716	-0.771303
NF1	neurofibromin 1	17	29421945	29709134	287190	-0.737749
NFS1	NFS1, cysteine desulfurase	20	34255977	34287281	31305	-0.559453
**NIPA1**	non imprinted in Prader-Willi/Angelman syndrome 1	15	23043277	23100005	56729	-1.103854
NIPA2	non imprinted in Prader-Willi/Angelman syndrome 2	15	23004684	23034427	29744	-1.103854
NOP14	NOP14 nucleolar protein	4	2939660	2965112	25453	-0.708963
NR6A1	nuclear receptor subfamily 6 group A member 1	9	127279554	127533589	254036	-0.605549
NSF	N-ethylmaleimide sensitive factor, vesicle fusing ATPase	17	44668035	44834830	166796	-0.551457
NSUN4	NOP2/Sun RNA methyltransferase family member 4	1	46805849	46830824	24976	-0.687345
NT5M	5',3'-nucleotidase, mitochondrial	17	17206649	17250977	44329	-0.543799
NUMB	NUMB, endocytic adaptor protein	14	73741815	73930348	188534	-0.681062
NUSAP1	nucleolar and spindle associated protein 1	15	41624892	41673248	48357	-0.645528
PAFAH1B1	platelet activating factor acetylhydrolase 1b regulatory subunit 1	17	2496504	2588909	92406	-0.551457
PARP1	poly(ADP-ribose) polymerase 1	1	226548392	226595780	47389	-0.930865
**PELP1**	proline, glutamate and leucine rich protein 1	17	4574679	4607632	32954	-0.551457
PEX14	peroxisomal biogenesis factor 14	1	10532345	10690815	158471	-0.76503
PHF20	PHD finger protein 20	20	34359896	34538303	178408	-0.737749
PICALM	phosphatidylinositol binding clathrin assembly protein	11	85668727	85780924	112198	-0.683973
PIGL	phosphatidylinositol glycan anchor biosynthesis class L	17	16120505	16252115	131611	-0.543799
PLCB1	phospholipase C beta 1	20	8112824	8949003	836180	-0.737749
PNP	purine nucleoside phosphorylase	14	20937113	20945253	8141	-0.953144
POLN	DNA polymerase nu	4	2073645	2243848	170204	-0.708963
POMGNT1	protein O-linked mannose N-acetylglucosaminyltransferase 1 (beta 1,2-)	1	46654354	46685977	31624	-0.687345
**PPP1R13B**	protein phosphatase 1 regulatory subunit 13B	14	104200089	104313927	113839	-0.697744
PSMA4	proteasome subunit alpha 4	15	78832747	78841604	8858	-0.624229
PTEN	phosphatase and tensin homolog	10	89622870	89731687	108818	-0.60181
PTK7	protein tyrosine kinase 7 (inactive)	6	43044006	43129457	85452	-0.564755
PUS10	pseudouridylate synthase 10	2	61167357	61245394	78038	-0.649384
RALY	RALY heterogeneous nuclear ribonucleoprotein	20	32581452	32696114	114663	-0.559453
RAP1GAP2	RAP1 GTPase activating protein 2	17	2680350	2941033	260684	-0.551457
RARS2	arginyl-tRNA synthetase 2, mitochondrial	6	88224096	88299721	75626	-0.860462
REL	REL proto-oncogene, NF-kB subunit	2	61108656	61158745	50090	-0.649384
RGS12	regulator of G protein signaling 12	4	3294755	3441640	146886	-0.708963
RNF180	ring finger protein 180	5	63461671	63668696	207026	-0.525298
RNU6-83P	RNA, U6 small nuclear 83, pseudogene	13	99677488	99677599	112	-0.674938
RTN3	reticulon 3	11	63448918	63527363	78446	-0.683973
SAMHD1	SAM and HD domain containing deoxynucleoside triphosphate triphosphohydrolase 1	20	35518632	35580246	61615	-0.737749
SCAI	suppressor of cancer cell invasion	9	127704887	127905785	200899	-0.697678
SEPT3	septin 3	22	42372276	42394225	21950	-0.585327

UBR4	ubiquitin protein ligase E3 component n-recognin 4	1	19401000	19536770	135771	0.641693
USP48	ubiquitin specific peptidase 48	1	22004791	22110099	105309	0.641693
VBP1	VHL binding protein 1	X	154425284	154468098	42815	0.533195
VWA5B1	von Willebrand factor A domain containing 5B1	1	20617412	20681387	63976	0.641693
ZBTB46	zinc finger and BTB domain containing 46	20	62375019	62462597	87579	0.625377
ZFYVE27	zinc finger FYVE-type containing 27	10	99496878	99520664	23787	0.799744
**ZNF512B**	zinc finger protein 512B	20	63956702	63969865	13164	0.625377

**SETX**	senataxin	9	135136743	135230372	93630	-0.637964
SGTB	small glutamine rich tetratricopeptide repeat containing beta	5	64961755	65018862	57108	-0.525298
SLC30A6	solute carrier family 30 member 6	2	32390933	32449448	58516	-0.649384
SLC33A1	solute carrier family 33 member 1	3	155538813	155572218	33406	-0.853769
SLC35A1	solute carrier family 35 member A1	6	88180341	88222054	41714	-0.564755
**SMN1**	survival of motor neuron 1, telomeric	5	70220768	70249769	29002	-0.773799
SMYD3	SET and MYND domain containing 3	1	245912642	246670614	757973	-0.929971
SOS2	SOS Ras/Rho guanine nucleotide exchange factor 2	14	50583847	50698276	114430	-0.659072
**SPAST**	spastin	2	32288680	32382706	94027	-0.649384
**SPG11**	SPG11, spatacsin vesicle trafficking associated	15	44854894	44955876	100983	-0.609004
SPG21	SPG21, maspardin	15	65255362	65282648	27287	-0.624229
SREBF2	sterol regulatory element binding transcription factor 2	22	42229109	42303312	74204	-0.585327
ST3GAL3	ST3 beta-galactoside alpha-2,3-sialyltransferase 3	1	44171495	44396831	225337	-0.640765
STIL	SCL/TAL1 interrupting locus	1	47715811	47779819	64009	-0.640765
STRBP	spermatid perinuclear RNA binding protein	9	125871779	126030855	159077	-0.605549
SUZ12	SUZ12 polycomb repressive complex 2 subunit	17	30264037	30328064	64028	-0.657847
SYT10	synaptotagmin 10	12	33527173	33592754	65582	-0.568094
TDRD9	tudor domain containing 9	14	104394799	104519004	124206	-0.697744
TECPR2	tectonin beta-propeller repeat containing 2	14	102829300	102968818	139519	-0.753573
TEF	TEF, PAR bZIP transcription factor	22	41763337	41795330	31994	-0.585327
TET1	tet methylcytosine dioxygenase 1	10	70320413	70454239	133827	-0.60181
TFB2M	transcription factor B2, mitochondrial	1	246703862	246729626	25765	-1.365486
TLDC2	TBC/LysM-associated domain containing 2	20	35504524	35522638	18115	-0.559453
TMEM17	transmembrane protein 17	2	62727356	62739029	11674	-0.649384
TMEM67	transmembrane protein 67	8	94767072	94831462	64391	-0.791099
TRIP4	thyroid hormone receptor interactor 4	15	64679947	64747502	67556	-0.624229
TRPC4AP	transient receptor potential cation channel subfamily C member 4 associated protein	20	33590207	33680674	90468	-0.559453
**TRPM7**	transient receptor potential cation channel subfamily M member 7	15	50844670	50979012	134343	-0.598606
UBE3C	ubiquitin protein ligase E3C	7	156931607	157062066	130460	-0.562186
ULK2	unc-51 like autophagy activating kinase 2	17	19674142	19771249	97108	-0.683327
UQCC1	ubiquinol-cytochrome c reductase complex assembly factor 1	20	33890369	33999944	109576	-0.559453
USP34	ubiquitin specific peptidase 34	2	61414598	61697904	283307	-0.649384
UTP15	UTP15, small subunit processome component	5	72861268	72877794	16527	-0.648573
UTP6	UTP6, small subunit processome component	17	30187923	30228784	40862	-0.657847
**VEGFA**	vascular endothelial growth factor A	6	43737921	43754224	16304	-0.564755
VPS35	VPS35, retromer complex component	16	46690054	46723430	33377	-0.657947
**VPS54**	VPS54, GARP complex subunit	2	64119280	64246206	126927	-0.368232
WDPCP	WD repeat containing planar cell polarity effector	2	63348518	64054977	706460	-0.368232
XPNPEP3	X-prolyl aminopeptidase 3	22	41253081	41363838	110758	-0.585327
XPO5	exportin 5	6	43490072	43543812	53741	-0.564755
ZFYVE28	zinc finger FYVE-type containing 28	4	2271309	2420390	149082	-0.708963
ZNF287	zinc finger protein 287	17	16454701	16472520	17820	-0.543799
ZNF318	zinc finger protein 318	6	43274872	43337216	62345	-0.564755
**ZSWIM7**	zinc finger SWIM-type containing 7	17	15879874	15903031	23158	-0.543799

Supplementary Table 9. List of genes located in the most recurrent CNVs of SALS2 patients (pentrance > 10%).

Duplications

Gene name	Gene description	Chromosome	Gene start (bp)	Gene end (bp)	Size (bp)	Log2Ratio (Mean)
ABCA2	ATP binding cassette subfamily A member 2	9	139901686	139923367	21682	0.694392
ABCA3	ATP binding cassette subfamily A member 3	16	2325882	2390747	64866	0.551909
ABCC8	ATP binding cassette subfamily C member 8	11	17414432	17498449	84018	0.5762887
ABCG1	ATP binding cassette subfamily G member 1	21	43619799	43717354	97556	0.6119098
ABHD2	abhydrolase domain containing 2	15	89630690	89745591	114902	0.5753199
ABLIM2	actin binding LIM protein family member 2	4	7967039	8160559	193521	0.5575473
ACAN	aggrecan	15	89346674	89418585	71912	0.5753199
ACHE	acetylcholinesterase (Cartwright blood group)	7	100487615	100494594	6980	0.835491
ADARB1	adenosine deaminase, RNA specific B1	21	46493768	46646478	152711	0.6119098
ADCY9	adenylate cyclase 9	16	4003388	4166186	162799	0.551909
ADGRG1	adhesion G protein-coupled receptor G1	16	57653442	57698944	45503	0.551909
AFAP1	actin filament associated protein 1	4	7760441	7941653	181213	0.5575473
AGER	advanced glycosylation end-product specific receptor	6	32148745	32152101	3357	0.6979757
AKAP4	A-kinase anchoring protein 4	X	49955406	49965664	10259	0.58026
AKT1	AKT serine/threonine kinase 1	14	105235686	105262088	26403	0.6005069
**ALAD**	aminolevulinate dehydratase	9	116148597	116163613	15017	0.590879
ALDH4A1	aldehyde dehydrogenase 4 family member A1	1	19197926	19229275	31350	0.5043603
ALG1	ALG1, chitobiosyldiphosphodolichol beta-mannosyltransferase	16	5083703	5137380	53678	0.551909
ALPL	alkaline phosphatase, liver/bone/kidney	1	21835858	21904905	69048	0.5884203
AMBRA1	autophagy and beclin 1 regulator 1	11	46417964	46615675	197712	0.609723
ANO9	anoctamin 9	11	417933	442011	24079	0.589939
AP2A2	adaptor related protein complex 2 alpha 2 subunit	11	924894	1012239	87346	0.589939
AP4E1	adaptor related protein complex 4 epsilon 1 subunit	15	51200869	51298097	97229	0.5420542
AP4S1	adaptor related protein complex 4 sigma 1 subunit	14	31494312	31562818	68507	1.1897469
AP5Z1	adaptor related protein complex 5 zeta 1 subunit	7	4815253	4833943	18691	0.589939
APBB1	amyloid beta precursor protein binding family B member 1	11	6416355	6440644	24290	0.9882033
APBB2	amyloid beta precursor protein binding family B member 2	4	40812044	41218731	406688	0.8738957
ARHGEF9	Cdc42 guanine nucleotide exchange factor 9	X	62854847	63005426	150580	0.5884203
ATP13A2	ATPase 13A2	1	17312453	17338423	25971	0.033088
BANK1	B-cell scaffold protein with ankyrin repeats 1	4	102332443	102995969	663527	0.8441654
**BCL11B**	B-cell CLL/lymphoma 11B	14	99635624	99737861	102238	0.5981354
BIN1	bridging integrator 1	2	127805603	127864931	59329	0.644462
BRD3	bromodomain containing 3	9	136895427	136933657	38231	0.694392
**C14orf177**	chromosome 14 open reading frame 177	14	99177950	99184098	6149	0.5073495
C16orf89	chromosome 16 open reading frame 89	16	5094123	5116111	21989	0.551909
C1QTNF1	C1q and TNF related 1	17	77018896	77045870	26975	0.621791
C1QTNF1-AS1	C1QTNF1 antisense RNA 1	17	77015291	77023737	8447	0.621791
C4A	complement C4A (Rodgers blood group)	6	31949801	31970458	20658	0.6979757
CABIN1	calcineurin binding protein 1	22	24407642	24574596	166955	0.7796157
**CACNA1H**	calcium voltage-gated channel subunit alpha1 H	16	1203241	1271771	68531	0.7071723
CACNB4	calcium voltage-gated channel auxiliary subunit beta 4	2	152689290	152955593	266304	0.5448835
CAMSAP1	calmodulin regulated spectrin associated protein 1	9	138700333	138799074	98742	0.694392
CAPN3	calpain 3	15	42640301	42704516	64216	0.3195894
CARD11	caspase recruitment domain family member 11	7	2945775	3083579	137805	0.5919394
CASP9	caspase 9	1	15817327	15853029	35703	0.4600978
CBS	cystathionine-beta-synthase	21	44473301	44497053	23753	0.7725278
CCDC120	coiled-coil domain containing 120	X	48911101	48927509	16409	0.58026
CCKBR	cholecystokinin B receptor	11	6280966	6293357	12392	0.589939

Deletions

Gene name	Gene description	Chromosome	Gene start (bp)	Gene end (bp)	Size (bp)
ABL2	ABL proto-oncogene 2, non-receptor tyrosine kinase	1	179068462	179198819	130358
ACE	angiotensin I converting enzyme	17	61554422	61599205	44784
ADAM9	ADAM metallopeptidase domain 9	8	38854388	38962663	108276
**ADK**	adenosine kinase	10	75910960	76469061	558102
ADSL	adenylosuccinate lyase	22	40742507	40786467	43961
ALG6	ALG6, alpha-1,3-glucosyltransferase	1	63833261	63904233	70973
AP4E1	adaptor related protein complex 4 epsilon 1 subunit	15	51200869	51298097	97229
ASTN1	astrotactin 1	1	176826438	177134109	307672
ATM	ATM serine/threonine kinase	11	108093211	108239829	146619
**ATXN2**	ataxin 2	12	111890018	112037480	147463
ATXN3	ataxin 3	14	92524896	92572965	48070
BIRC6	baculoviral IAP repeat containing 6	2	32582096	32843966	261871
C12orf56	chromosome 12 open reading frame 56	12	64660217	64784972	124756
CD109	CD109 molecule	6	74405508	74538040	132533
CHP1	calcineurin like EF-hand protein 1	15	41523037	41574043	51007
CHRNA3	cholinergic receptor nicotinic alpha 3 subunit	15	78885394	78913637	28244
CHRNA5	cholinergic receptor nicotinic alpha 5 subunit	15	78857862	78887611	29750
COQ2	coenzyme Q2, polyprenyltransferase	4	84182689	84206067	23379
CR1	complement C3b/C4b receptor 1 (Knops blood group)	1	207669492	207813992	144501
CRLF3	cytokine receptor like factor 3	17	29096406	29151794	55389
CSNK1G1	casein kinase 1 gamma 1	15	64457716	64648442	190727
CUL4B	cullin 4B	X	119658464	119709649	51186
DAPK2	death associated protein kinase 2	15	64199235	64364232	164998
DARS2	aspartyl-tRNA synthetase 2, mitochondrial	1	173793641	173827684	34044
DDHD2	DDHD domain containing 2	8	38082736	38133076	50341
DEPDC5	DEP domain containing 5	22	32149944	32303012	153069
DIP2B	disco interacting protein 2 homolog B	12	50898768	51142450	243683
DLGAP4	DLG associated protein 4	20	34894258	35157040	262783
EPB41L1	erythrocyte membrane protein band 4.1 like 1	20	34679426	34820721	141296
**EPHA4**	EPH receptor A4	2	222282747	222438922	156176
ERLIN1	ER lipid raft associated 1	10	101909851	101948091	38241
FAM126A	family with sequence similarity 126 member A	7	22980878	23053749	72872
FAM193A	family with sequence similarity 193 member A	4	2626988	2734292	107305
FBLN5	fibulin 5	14	92335756	92414331	78576
FGD4	FYVE, RhoGEF and PH domain containing 4	12	32552463	32798984	246522
FGFR1	fibroblast growth factor receptor 1	8	38268656	38326352	57697
FGFR3	fibroblast growth factor receptor 3	4	1795034	1810599	15566
FILIP1	filamin A interacting protein 1	6	76001575	76203454	201880
FKTN	fukutin	9	108320411	108403399	82989
GRK5	G protein-coupled receptor kinase 5	10	120967101	121215131	248031
GUSBP3	glucuronidase, beta pseudogene 3	5	68790040	69006341	216302
HLA-DRA	major histocompatibility complex, class II, DR alpha	6	32407619	32412823	5205
HLA-DRB5	major histocompatibility complex, class II, DR beta 5	6	32485120	32498064	12945
HNF4A	hepatocyte nuclear factor 4 alpha	20	42984340	43061485	77146
HSPA5	heat shock protein family A (Hsp70) member 5	9	127997132	128003609	6478
HTT	huntingtin	4	3076408	3245676	169269
HYKK	hydroxylysine kinase	15	78799906	78829714	29809
IL6	interleukin 6	7	22765503	22771621	6119

CD24	CD24 molecule	6	107417708	107422630	4923	1.0052812	IREB2	iron responsive element binding protein 2	15	78729773	78793798	64026
CDA	cytidine deaminase	1	20915441	20945401	29961	0.5884203	ITGAE	integrin subunit alpha E	17	3617922	3704537	86616
CEP104	centrosomal protein 104	1	3728645	3773778	45134	0.5884203	KAT6B	lysine acetyltransferase 6B	10	76585340	76792380	207041
CHD5	chromodomain helicase DNA binding protein 5	1	6161853	6240183	78331	0.5861476	KCNAB1	potassium voltage-gated channel subfamily A member regulatory beta subunit 1	3	155755490	156256545	501056
CHRNA4	cholinergic receptor nicotinic alpha 4 subunit	20	61975420	62009753	34334	0.6292466	**KCNQ5**	potassium voltage-gated channel subfamily Q member 5	6	73331520	73908574	577055
CIRBP	cold inducible RNA binding protein	19	1259384	1274879	15496	0.7471475	KIF11	kinesin family member 11	10	94353043	94415150	62108
CLCNKA	chloride voltage-gated channel Ka	1	16345370	16360545	15176	0.5988849	LEKR1	leucine, glutamate and lysine rich 1	3	156543270	156763918	220649
CLCNKB	chloride voltage-gated channel Kb	1	16370272	16383803	13532	0.5988849	LETMD1	LETM1 domain containing 1	12	51441745	51454207	12463
CLN3	CLN3, battenin	16	28477983	28506896	28914	0.5629104	MGA	MGA, MAX dimerization protein	15	41913422	42062141	148720
CNTN2	contactin 2	1	205012325	205047627	35303	0.636841	MIR1268A	microRNA 1268a	15	22513229	22513280	52
COG4	component of oligomeric golgi complex 4	16	70514471	70557468	42998	0.4390636	MIR548F1	microRNA 548f-1	10	56367634	56367717	84
COL18A1	collagen type XVIII alpha 1 chain	21	46825052	46933634	108583	0.6119098	MKL1	megakaryoblastic leukemia (translocation) 1	22	40806285	41032706	226422
CPEB4	cytoplasmic polyadenylation element binding protein 4	5	173315283	173388979	73697	0.5299	MKNK1	MAP kinase interacting serine/threonine kinase 1	1	47023090	47082515	59426
**CPZ**	carboxypeptidase Z	4	8594387	8621488	27102	0.5575473	MMP24	matrix metallopeptidase 24	20	33814457	33864801	50345
CREBBP	CREB binding protein	16	3775055	3930727	155673	0.551909	MMP9	matrix metallopeptidase 9	20	44637547	44645200	7654
CRMP1	collapsin response mediator protein 1	4	5749811	5894785	144975	0.5575473	MYO6	myosin VI	6	76458909	76629254	170346
CRY2	cryptochrome circadian clock 2	11	45868669	45904798	36130	0.609723	NDRG3	NDRG family member 3	20	35280169	35374481	94313
CSF1	colony stimulating factor 1	1	110452864	110473614	20751	0.5301805	NDUFAF1	NADH:ubiquinone oxidoreductase complex assembly factor 1	15	41679551	41694717	15167
**CST3**	cystatin C	20	23608534	23619110	10577	0.9021142	NDUFV2	NADH:ubiquinone oxidoreductase core subunit V2	18	9102628	9134343	31716
CSTB	cystatin B	21	45192393	45196326	3934	0.6119098	NF1	neurofibromin 1	17	29421945	29709134	287190
CTDP1	CTD phosphatase subunit 1	18	77439801	77514510	74710	0.626131	NFS1	NFS1, cysteine desulfurase	20	34255977	34287281	31305
CTSD	cathepsin D	11	1773982	1785222	11241	0.589939	**NIPA1**	non imprinted in Prader-Willi/Angelman syndrome 1	15	23043277	23100005	56729
**CYP2D6**	cytochrome P450 family 2 subfamily D member 6	22	42522501	42526908	4408	0.6372778	NIPA2	non imprinted in Prader-Willi/Angelman syndrome 2	15	23004684	23034427	29744
CYP46A1	cytochrome P450 family 46 subfamily A member 1	14	100150641	100193638	42998	0.5981354	NOP14	NOP14 nucleolar protein	4	2939660	2965112	25453
CYTH1	cytohesin 1	17	76670130	76778379	108250	0.621791	NSUN4	NOP2/Sun RNA methyltransferase family member 4	1	46805849	46830824	24976
**DBH**	dopamine beta-hydroxylase	9	136501482	136524466	22985	0.694392	NUCKS1	nuclear casein kinase and cyclin dependent kinase substrate 1	1	205681947	205719404	37458
**DCTN1**	dynactin subunit 1	2	74588281	74619214	30934	0.63725	NUSAP1	nucleolar and spindle associated protein 1	15	41624892	41673248	48357
DDOST	dolichyl-diphosphooligosaccharide--protein glycosyltransferase non-catalytic subunit	1	20978270	20988000	9731	0.5884203	PAFAH1B1	platelet activating factor acetylhydrolase 1b regulatory subunit 1	17	2496504	2588909	92406
**DEAF1**	DEAF1, transcription factor	11	644233	706715	62483	0.589939	PAPPA2	pappalysin 2	1	176432307	176814735	382429
DGKQ	diacylglycerol kinase theta	4	952675	980683	28009	0.5575473	**PELP1**	proline, glutamate and leucine rich protein 1	17	4574679	4607632	32954
DHCR24	24-dehydrocholesterol reductase	1	55315306	55352891	37586	0.6525684	PHF20	PHD finger protein 20	20	34359896	34538303	178408
DIDO1	death inducer-obliterator 1	20	61509090	61569304	60215	0.6180608	PICALM	phosphatidylinositol binding clathrin assembly protein	11	85668727	85780924	112198
DNAH17	dynein axonemal heavy chain 17	17	76419778	76573476	153699	0.621791	PKIG	cAMP-dependent protein kinase inhibitor gamma	20	43160426	43252888	92463
DNAJC6	DnaJ heat shock protein family (Hsp40) member C6	1	65713902	65881552	167651	0.995453	PLXNA2	plexin A2	1	208195587	208417665	222079
DOCK2	dedicator of cytokinesis 2	5	169064251	169510386	446136	0.5299	POLN	DNA polymerase nu	4	2073645	2243848	170204
DRD1	dopamine receptor D1	5	174867042	174871211	4170	0.5299	POMGNT1	protein O-linked mannose N-acetylglucosaminyltransferase 1 (beta 1,2-)	1	46654354	46685977	31624
**DRD4**	dopamine receptor D4	11	637293	640706	3414	0.589939	**PPP1R13B**	protein phosphatase 1 regulatory subunit 13B	14	104200089	104313927	113839
DRD5	dopamine receptor D5	4	9783258	9785632	2375	0.540562	PSMA4	proteasome subunit alpha 4	15	78832747	78841604	8858
ECE1	endothelin converting enzyme 1	1	21543740	21671997	128258	0.5884203	RABGAP1L	RAB GTPase activating protein 1 like	1	174128548	174964445	835898
EDF1	endothelial differentiation related factor 1	9	139756571	139760738	4168	0.694392	RAP1GAP2	RAP1 GTPase activating protein 2	17	2680350	2941033	260684
**EEF1D**	eukaryotic translation elongation factor 1 delta	8	144661867	144681711	19845	0.6315766	RASAL2	RAS protein activator like 2	1	178062864	178448644	385781
EIF4G3	eukaryotic translation initiation factor 4 gamma 3	1	21132963	21503377	370415	0.5884203	RGS12	regulator of G protein signaling 12	4	3294755	3441640	146886
ELFN2	extracellular leucine rich repeat and fibronectin type III domain containing 2	22	37764000	37823505	59506	0.5478177	RIMS4	regulating synaptic membrane exocytosis 4	20	43380449	43438979	58531
ELK1	ELK1, ETS transcription factor	X	47494920	47510003	15084	0.58026	RNU6-83P	RNA, U6 small nuclear 83, pseudogene	13	99677488	99677599	112
F2	coagulation factor II, thrombin	11	46740730	46761056	20327	0.4572923	SAMHD1	SAM and HD domain containing deoxynucleoside triphosphate triphosphohydrolase 1	20	35518632	35580246	61615

FA2H	fatty acid 2-hydroxylase	16	74746853	74808729	61877	0.6553103
FANCI	Fanconi anemia complementation group I	15	89787180	89860492	73313	0.5753199
FARP1	FERM, ARH/RhoGEF and pleckstrin domain protein 1	13	98794816	99102027	307212	0.6639247
**FBXO42**	F-box protein 42	1	16573334	16678949	105616	0.5884203
FBXW11	F-box and WD repeat domain containing 11	5	171288553	171433877	145325	0.5299
FLNA	filamin A	X	153576892	153603006	26115	0.6237213
GAA	glucosidase alpha, acid	17	78075355	78093678	18324	0.621791
GABRB3	gamma-aminobutyric acid type A receptor beta3 subunit	15	26788693	27184686	395994	0.8187364
GABRD	gamma-aminobutyric acid type A receptor delta subunit	1	1950780	1962192	11413	0.4622
GAD1	glutamate decarboxylase 1	2	171669723	171717661	47939	0.7396482
GAK	cyclin G associated kinase	4	843064	926161	83098	0.5575473
GAMT	guanidinoacetate N-methyltransferase	19	1397091	1401569	4479	0.7471475
GATA2	GATA binding protein 2	3	128198270	128212028	13759	0.7199733
GATA2-AS1	GATA2 antisense RNA 1	3	128208036	128216768	8733	0.7199733
GDNF	glial cell derived neurotrophic factor	5	37812779	37839788	27010	1.1191921
GLG1	golgi glycoprotein 1	16	74485856	74641012	155157	0.5259785
GLYR1	glyoxylate reductase 1 homolog	16	4853204	4897343	44140	0.551909
GNA12	G protein subunit alpha 12	7	2767746	2883958	116213	0.5919394
GPSM3	G protein signaling modulator 3	6	32158543	32163300	4758	0.655532
GRIA3	glutamate ionotropic receptor AMPA type subunit 3	X	122318006	122624766	306761	0.7209059
GRIN1	glutamate ionotropic receptor NMDA type subunit 1	9	140032842	140063207	30366	0.694392
GRIN2A	glutamate ionotropic receptor NMDA type subunit 2A	16	9852376	10276611	424236	0.8357534
GRIN3B	glutamate ionotropic receptor NMDA type subunit 3B	19	1000418	1009731	9314	0.7471475
GRK6	G protein-coupled receptor kinase 6	5	176830205	176869902	39698	0.5927958
**GSDMD**	gasdermin D	8	144635377	144645232	9856	0.6315766
GSPT2	G1 to S phase transition 2	X	51486481	51489324	2844	0.58026
GSTM3	glutathione S-transferase mu 3	1	110276554	110284384	7831	0.5301805
GSTT1	glutathione S-transferase theta 1	22	24376133	24384680	8548	1.1054935
HBB	hemoglobin subunit beta	11	5246694	5250625	3932	0.589939
HDAC6	histone deacetylase 6	X	48659784	48683392	23609	0.58026
HLA-A	major histocompatibility complex, class I, A	6	29909037	29913661	4625	0.9732702
HLA-DRB5	major histocompatibility complex, class II, DR beta 5	6	32485120	32498064	12945	0.5135375
HMOX2	heme oxygenase 2	16	4524691	4560348	35658	0.551909
HP	haptoglobin	16	72088491	72094954	6464	0.5259785
HS1BP3	HCLS1 binding protein 3	2	20760208	20850849	90642	0.6359472
HSF2BP	heat shock transcription factor 2 binding protein	21	44949072	45079374	130303	0.6119098
HSPG2	heparan sulfate proteoglycan 2	1	22148738	22263790	115053	0.5781296
HTR6	5-hydroxytryptamine receptor 6	1	19991780	20006055	14276	0.5884203
IDE	insulin degrading enzyme	10	94211441	94333833	122393	0.2374174
IGF2	insulin like growth factor 2	11	2150342	2170833	20492	0.7673512
IGSF21	immunoglobin superfamily member 21	1	18434240	18704977	270738	0.5884203
INS-IGF2	INS-IGF2 readthrough	11	2153768	2182439	28672	0.7673512
IQSEC2	IQ motif and Sec7 domain 2	X	53262058	53350522	88465	0.58026
ITGB2	integrin subunit beta 2	21	46305868	46351904	46037	0.6119098
ITPKB	inositol-trisphosphate 3-kinase B	1	226819391	226927024	107634	0.6475943
IZUMO2	IZUMO family member 2	19	50655805	50666452	10648	0.588151
JAKMIP1	janus kinase and microtubule interacting protein 1	4	6027926	6202318	174393	0.5575473
KCNC3	potassium voltage-gated channel subfamily C member 3	19	50815194	50836772	21579	0.588151
KCNIP1	potassium voltage-gated channel interacting protein 1	5	169780491	170163636	383146	0.5299
KCNJ11	potassium voltage-gated channel subfamily J member 11	11	17407406	17410878	3473	0.5815642

SCAI	suppressor of cancer cell invasion	9	127704887	127905785	200899
SENP6	SUMO1/sentrin specific peptidase 6	6	76311225	76427997	116773
**SETX**	senataxin	9	135136743	135230372	93630
SIPA1L3	signal induced proliferation associated 1 like 3	19	38397868	38699012	301145
SLC11A2	solute carrier family 11 member 2	12	51373184	51422349	49166
SLC17A5	solute carrier family 17 member 5	6	74303102	74363878	60777
SLC30A6	solute carrier family 30 member 6	2	32390933	32449448	58516
SLC33A1	solute carrier family 33 member 1	3	155538813	155572218	33406
**SMN1**	survival of motor neuron 1, telomeric	5	70220768	70249769	29002
SMYD3	SET and MYND domain containing 3	1	245912642	246670614	757973
SOAT1	sterol O-acyltransferase 1	1	179262925	179327815	64891
SOS1	SOS Ras/Rac guanine nucleotide exchange factor 1	2	39208537	39351486	142950
SOS2	SOS Ras/Rho guanine nucleotide exchange factor 2	14	50583847	50698276	114430
**SPAST**	spastin	2	32288680	32382706	94027
SPATS2	spermatogenesis associated serine rich 2	12	49760367	49921205	160839
**SPG11**	SPG11, spatacsin vesicle trafficking associated	15	44854894	44955876	100983
SPG21	SPG21, maspardin	15	65255362	65282648	27287
SREBF2	sterol regulatory element binding transcription factor 2	22	42229109	42303312	74204
SRGAP1	SLIT-ROBO Rho GTPase activating protein 1	12	64238073	64541613	303541
ST3GAL3	ST3 beta-galactoside alpha-2,3-sialyltransferase 3	1	44171495	44396831	225337
STIL	SCL/TAL1 interrupting locus	1	47715811	47779819	64009
STK4	serine/threonine kinase 4	20	43595115	43708600	113486
SUZ12	SUZ12 polycomb repressive complex 2 subunit	17	30264037	30328064	64028
**TAF15**	TATA-box binding protein associated factor 15	17	34136459	34191619	55161
**TBK1**	TANK binding kinase 1	12	64845660	64895888	50229
TDRD9	tudor domain containing 9	14	104394799	104519004	124206
TEF	TEF, PAR bZIP transcription factor	22	41763337	41795330	31994
TET1	tet methylcytosine dioxygenase 1	10	70320413	70454239	133827
TFB2M	transcription factor B2, mitochondrial	1	246703862	246729626	25765
TFCP2	transcription factor CP2	12	51487446	51566926	79481
TIPARP	TCDD inducible poly(ADP-ribose) polymerase	3	156391024	156424559	33536
TLDC2	TBC/LysM-associated domain containing 2	20	35504524	35522638	18115
TMEM5	transmembrane protein 5	12	64173583	64203338	29756
TMEM67	transmembrane protein 67	8	94767072	94831462	64391
TNR	tenascin R	1	175284330	175712906	428577
TRIP4	thyroid hormone receptor interactor 4	15	64679947	64747502	67556
**TRPM7**	transient receptor potential cation channel subfamily M member 7	15	50844670	50979012	134343
ULK2	unc-51 like autophagy activating kinase 2	17	19674142	19771249	97108
UQCC1	ubiquinol-cytochrome c reductase complex assembly factor 1	20	33890369	33999944	109576
UTP6	UTP6, small subunit processome component	17	30187923	30228784	40862
VPS35	VPS35, retromer complex component	16	46690054	46723430	33377
**VPS54**	VPS54, GARP complex subunit	2	64119280	64246206	126927
XPNPEP3	X-prolyl aminopeptidase 3	22	41253081	41363838	110758
ZBTB37	zinc finger and BTB domain containing 37	1	173837220	173872687	35468
ZFYVE28	zinc finger FYVE-type containing 28	4	2271309	2420390	149082
ZNF567	zinc finger protein 567	19	36687612	36727701	40090
ZNF585A	zinc finger protein 585A	19	37106734	37172741	66008

KCNQ1	potassium voltage-gated channel subfamily Q member 1	11	2465914	2870339	404426	0.589939
KCNQ2	potassium voltage-gated channel subfamily Q member 2	20	62037542	62103993	66452	0.623586
KCNT1	potassium sodium-activated channel subfamily T member 1	9	138594031	138684992	90962	0.7767136
KDM5C	lysine demethylase 5C	X	53220503	53254604	34102	0.58026
KDM5D	lysine demethylase 5D	Y	21865751	21906825	41075	1.0052812
KIAA0232	KIAA0232	4	6783102	6885897	102796	0.5575473
KIAA1191	KIAA1191	5	175773064	175788971	15908	0.5299
KIF11	kinesin family member 11	10	94353043	94415150	62108	0.1978478
KIF1A	kinesin family member 1A	2	241653181	241759725	106545	0.5962652
KIF7	kinesin family member 7	15	90152020	90198682	46663	0.5753199
KRTAP5-AS1	KRTAP5-1/KRTAP5-2 antisense RNA 1	11	1592583	1620414	27832	0.7673512
L1CAM	L1 cell adhesion molecule	X	153126969	153174677	47709	0.6237213
LDLRAD2	low density lipoprotein receptor class A domain containing 2	1	22138758	22151714	12957	0.5781296
LIMCH1	LIM and calponin homology domains 1	4	41361624	41702061	340438	1.1705494
LIMS2	LIM zinc finger domain containing 2	2	128395956	128439360	43405	0.644462
LINC00955	long intergenic non-protein coding RNA 955	4	3578596	3592438	13843	1.1705494
LINC01284	long intergenic non-protein coding RNA 1284	X	50844121	50844465	345	0.58026
LINC01411	long intergenic non-protein coding RNA 1411	5	173953231	173954142	912	0.1368968
LINC01568	long intergenic non-protein coding RNA 1568	16	73420704	73455295	34592	0.5259785
LINC01572	long intergenic non-protein coding RNA 1572	16	72302913	72324506	21594	0.5259785
LMF1	lipase maturation factor 1	16	903634	1031318	127685	0.6570818
LMNA	lamin A/C	1	156052364	156109880	57517	0.6092605
LMX1A	LIM homeobox transcription factor 1 alpha	1	165171104	165325952	154849	0.5950276
LMX1B	LIM homeobox transcription factor 1 beta	9	129376722	129463311	86590	0.7284717
**LOX**	lysyl oxidase	5	121398890	121413980	15091	0.7595988
LRP1	LDL receptor related protein 1	12	57522276	57607134	84859	0.6543884
LRPAP1	LDL receptor related protein associated protein 1	4	3508103	3534286	26184	0.5575473
LYAR	Ly1 antibody reactive	4	4269428	4291896	22469	0.5575473
MAN1B1	mannosidase alpha class 1B member 1	9	139981379	140003635	22257	0.694392
MAPK8IP1	mitogen-activated protein kinase 8 interacting protein 1	11	45907202	45928016	20815	0.7521324
MECP2	methyl-CpG binding protein 2	X	153287024	153363212	76189	0.6237213
MEFV	MEFV, pyrin innate immunity regulator	16	3292028	3306627	14600	0.551909
MIR1-1HG	MIR1-1 host gene	20	61147660	61167971	20312	0.565306
MIR1268A	microRNA 1268a	15	22513229	22513280	52	0.2710271
MMEL1	membrane metalloendopeptidase like 1	1	2522078	2564481	42404	0.5861476
MORN1	MORN repeat containing 1	1	2252692	2323146	70455	0.1048694
MPO	myeloperoxidase	17	56347217	56358296	11080	0.6162667
MVB12B	multivesicular body subunit 12B	9	129089128	129269320	180193	0.7145743
MYH14	myosin heavy chain 14	19	50691443	50813802	122360	0.6069893
**NAIP**	NLR family apoptosis inhibitory protein	5	70264310	70320941	56632	0.1368968
NAP1L4	nucleosome assembly protein 1 like 4	11	2965667	3013607	47941	0.589939
NDUFA6	NADH:ubiquinone oxidoreductase subunit A6	22	42481529	42486959	5431	0.6372778
NDUFS7	NADH:ubiquinone oxidoreductase core subunit S7	19	1383526	1395583	12058	0.7471475
NELFE	negative elongation factor complex member E	6	31919864	31926887	7024	0.6273893
NFKB1	nuclear factor kappa B subunit 1	4	103422486	103538459	115974	0.8441654
NLGN4Y	neuroligin 4, Y-linked	Y	16634518	16957530	323013	0.902015
NMRAL1	NmrA like redox sensor 1	16	4511681	4545764	34084	0.551909
NOP56	NOP56 ribonucleoprotein	20	2632791	2639039	6249	0.4282188
NOS2	nitric oxide synthase 2	17	26083792	26127525	43734	0.5250283
NOTCH4	notch 4	6	32162620	32191844	29225	0.6273893
NPHP4	nephrocystin 4	1	5922871	6052533	129663	0.5861476
NR1H2	nuclear receptor subfamily 1 group H member 2	19	50832910	50886239	53330	0.5744436
NSD1	nuclear receptor binding SET domain protein 1	5	176560026	176727216	167191	0.2475641
NTRK1	neurotrophic receptor tyrosine kinase 1	1	156785432	156851642	66211	0.6092605
NTRK2	neurotrophic receptor tyrosine kinase 2	9	87283466	87638505	355040	1.0474547
NUDT1	nudix hydrolase 1	7	2281857	2290781	8925	0.5919394
PANK2	pantothenate kinase 2	20	3869486	3907605	38120	0.9746154
PCDH11Y	protocadherin 11 Y-linked	Y	4868267	5610265	741999	1.1085184
PCGF3	polycomb group ring finger 3	4	699537	764428	64892	0.5575473
PCSK9	proprotein convertase subtilisin/kexin type 9	1	55505221	55530525	25305	0.6525684
PDXK	pyridoxal kinase	21	45138975	45182188	43214	0.6119098
PDYN	prodynorphin	20	1959403	1974732	15330	0.565306
PEX10	peroxisomal biogenesis factor 10	1	2336236	2345236	9001	0.5861476
PHOX2B	paired like homeobox 2b	4	41746099	41750987	4889	1.1705494

PIGS	phosphatidylinositol glycan anchor biosynthesis class S	17	26880401	26898890	18490	0.5112525
PIK3R5	phosphoinositide-3-kinase regulatory subunit 5	17	8782233	8869029	86797	0.6240758
PINK1	PTEN induced putative kinase 1	1	20959948	20978004	18057	0.5884203
PINK1-AS	PINK1 antisense RNA	1	20969150	20978686	9537	0.5884203
PKD1	polycystin 1, transient receptor potential channel interacting	16	2138711	2185899	47189	0.5804946
PLA2G6	phospholipase A2 group VI	22	38507502	38601697	94196	0.0873912
PLAT	plasminogen activator, tissue type	8	42032236	42065242	33007	0.5478177
PLEC	plectin	8	144989321	145050902	61582	0.5544203
**PLEKHG5**	pleckstrin homology and RhoGEF domain containing G5	1	6526152	6580121	53970	0.5861476
PLXNA2	plexin A2	1	208195587	208417665	222079	0.3842927
PNKP	polynucleotide kinase 3'-phosphatase	19	50364461	50371166	6706	0.6122093
POLG	DNA polymerase gamma, catalytic subunit	15	89859534	89878092	18559	0.5753199
POLR2F	RNA polymerase II subunit F	22	38348614	38437922	89309	0.5478177
PORCN	porcupine homolog (Drosophila)	X	48367350	48379202	11853	0.58026
PPP1R3F	protein phosphatase 1 regulatory subunit 3F	X	49126306	49157929	31624	0.58026
PPP2R2C	protein phosphatase 2 regulatory subunit Bgamma	4	6322305	6565327	243023	0.5575473
PPP3CA	protein phosphatase 3 catalytic subunit alpha	4	101944566	102269435	324870	0.8441654
PRDM16	PR/SET domain 16	1	2985732	3355185	369454	0.5861476
PRKCZ	protein kinase C zeta	1	1981909	2116834	134926	0.6162667
PRKY	protein kinase, Y-linked, pseudogene	Y	7142013	7249589	107577	1.1085184
PROCA1	protein interacting with cyclin A1	17	27030215	27038872	8658	0.5112525
PRY	PTPN13-like, Y-linked	Y	24636544	24660784	24241	0.9863595
PSEN2	presenilin 2	1	227057885	227083806	25922	0.627898
PTTG1IP	PTTG1 interacting protein	21	46269500	46293752	24253	0.6119098
RAC2	ras-related C3 botulinum toxin substrate 2 (rho family, small GTP binding protein Rac2)	22	37621301	37640488	19188	0.575554
RANBP17	RAN binding protein 17	5	170288874	170727019	438146	0.5299
RBFOX1	RNA binding protein, fox-1 homolog 1	16	6069095	7763340	1694246	0.551909
RBFOX3	RNA binding protein, fox-1 homolog 3	17	77085427	77613550	528124	0.5112525
RCC2	regulator of chromosome condensation 2	1	17733256	17766220	32965	0.5884203
REEP2	receptor accessory protein 2	5	137774706	137782658	7953	0.6014213
RER1	retention in endoplasmic reticulum sorting receptor 1	1	2323267	2336883	13617	0.6162667
RHCG	Rh family C glycoprotein	15	89998680	90039844	41165	0.5753199
RNASEH2B-AS1	RNASEH2B antisense RNA 1	13	51450822	51484848	34027	0.6639247
ROGDI	rogdi homolog	16	4846969	4852951	5983	0.551909
RRP8	ribosomal RNA processing 8, methyltransferase, homolog (yeast)	11	6616305	6624850	8546	0.589939
RXRA	retinoid X receptor alpha	9	137208944	137332431	123488	0.694392
RXRG	retinoid X receptor gamma	1	165370159	165414433	44275	0.5950276
**SARM1**	sterile alpha and TIR motif containing 1	17	26691378	26728065	36688	0.617968
SBNO2	strawberry notch homolog 2	19	1107636	1174282	66647	0.7471475
SCARB1	scavenger receptor class B member 1	12	125261402	125367214	105813	1.2428278
**SDK1**	sidekick cell adhesion molecule 1	7	3341080	4308632	967553	0.5919394
SEC16A	SEC16 homolog A, endoplasmic reticulum export factor	9	139334549	139372141	37593	0.694392
SEPT9	septin 9	17	75276651	75496678	220028	0.621791
SERPINF2	serpin family F member 2	17	1646130	1658562	12433	0.7342318
SETD3	SET domain containing 3	14	99864083	99947216	83134	0.5981354
SH2D2A	SH2 domain containing 2A	1	156776035	156786654	10620	0.6092605
SHROOM4	shroom family member 4	X	50334647	50557302	222656	0.58026
SLC25A22	solute carrier family 25 member 22	11	790475	798316	7842	0.589939
SLC35A2	solute carrier family 35 member A2	X	48760459	48769235	8777	0.58026
SLC35C1	solute carrier family 35 member C1	11	45825623	45834566	8944	0.609723
SLC37A1	solute carrier family 37 member 1	21	43916118	44001550	85433	0.6119098
SLC46A1	solute carrier family 46 member 1	17	26721661	26734215	12555	0.5250283
SLIT3	slit guidance ligand 3	5	168088745	168728133	639389	0.8875385
SNCAIP	synuclein alpha interacting protein	5	121647049	121799914	152866	0.7595988
SNCB	synuclein beta	5	176047085	176057530	10446	0.5299
SORCS2	sortilin related VPS10 domain containing receptor 2	4	7194265	7744554	550290	0.5575473
SPAG5-AS1	SPAG5 antisense RNA 1	17	26925808	26944393	18586	0.5112525
SPECC1L	sperm antigen with calponin homology and coiled-coil domains 1 like	22	24666786	24813708	146923	0.7796157
SPECC1L-ADORA2A	SPECC1L-ADORA2A readthrough (NMD candidate)	22	24666866	24838324	171459	0.7796157

SPEN	spen family transcriptional repressor	1	16174359	16266955	92597	0.4600978
SREBF1	sterol regulatory element binding transcription factor 1	17	17713713	17740325	26613	0.7216414
**SS18L1**	SS18L1, nBAF chromatin remodeling complex subunit	20	60718822	60757540	38719	0.7820486
SSX5	SSX family member 5	X	48045656	48056199	10544	0.58026
STIM1	stromal interaction molecule 1	11	3875757	4114439	238683	0.589939
STK11	serine/threonine kinase 11	19	1189406	1228428	39023	0.7471475
STK32B	serine/threonine kinase 32B	4	5053169	5502725	449557	0.5575473
STX18	syntaxin 18	4	4417469	4544073	126605	0.5575473
SYN1	synapsin I	X	47431303	47479252	47950	0.58026
**SYT9**	synaptotagmin 9	11	7260009	7490273	230265	0.589939
TBC1D16	TBC1 domain family member 16	17	77906142	78009647	103506	0.621791
TBC1D24	TBC1 domain family member 24	16	2525147	2555735	30589	0.551909
TBL1Y	transducin beta like 1, Y-linked	Y	6778727	6959724	180998	1.1085184
TGM6	transglutaminase 6	20	2361554	2413399	51846	0.565306
TH	tyrosine hydroxylase	11	2185159	2193107	7949	0.589939
**TIMP2**	TIMP metallopeptidase inhibitor 2	17	76849059	76921469	72411	0.621791
TMEM97	transmembrane protein 97	17	26646121	26655707	9587	0.5250283
TNRC6C	trinucleotide repeat containing 6C	17	76000249	76104916	104668	0.621791
TP73	tumor protein p73	1	3569084	3652765	83682	0.5861476
TPP1	tripeptidyl peptidase 1	11	6634000	6640692	6693	0.589939
TRAF2	TNF receptor associated factor 2	9	139776364	139821059	44696	0.694392
TRAF4	TNF receptor associated factor 4	17	27071002	27077974	6973	0.5112525
TRAP1	TNF receptor associated protein 1	16	3701640	3767598	65959	0.551909
TRIM5	tripartite motif containing 5	11	5684425	5959849	275425	0.589939
TRMT44	tRNA methyltransferase 44 homolog (S. cerevisiae)	4	8437867	8495258	57392	0.5575473
**TRPM7**	transient receptor potential cation channel subfamily M member 7	15	50844670	50979012	134343	0.2710271
TSC1	tuberous sclerosis 1	9	135766735	135820020	53286	0.75104
TSC2	tuberous sclerosis 2	16	2097466	2138716	41251	0.5804946
TSPEAR	thrombospondin type laminin G domain and EAR repeats	21	45917775	46131495	213721	0.6119098
TTTY10	testis-specific transcript, Y-linked 10 (non-protein coding)	Y	22627554	22681114	53561	0.923349
TTTY14	testis-specific transcript, Y-linked 14 (non-protein coding)	Y	21034387	21239302	204916	1.0052812
UBASH3A	ubiquitin associated and SH3 domain containing A	21	43824008	43867791	43784	0.6119098
**UBE2I**	ubiquitin conjugating enzyme E2 I	16	1355548	1377019	21472	0.6549837
UBR4	ubiquitin protein ligase E3 component n-recognin 4	1	19401000	19536770	135771	0.5043603
UCHL1	ubiquitin C-terminal hydrolase L1	4	41258430	41270472	12043	0.9882033
UIMC1	ubiquitin interaction motif containing 1	5	176332006	176449634	117629	0.5299
UNKL	unkempt family like zinc finger	16	1413206	1464752	51547	0.5889578
USP48	ubiquitin specific peptidase 48	1	22004791	22110099	105309	0.5884203
UTY	ubiquitously transcribed tetratricopeptide repeat containing, Y-linked	Y	15360259	15592553	232295	0.902015
**VCP**	valosin containing protein	9	35056061	35073246	17186	1.1148755
VWA5B1	von Willebrand factor A domain containing 5B1	1	20617412	20681387	63976	0.5884203
WDR13	WD repeat domain 13	X	48448430	48463581	15152	0.58026
WDR45	WD repeat domain 45	X	48929385	48958108	28724	0.58026
WFS1	wolframin ER transmembrane glycoprotein	4	6271576	6304992	33417	0.5575473
WWC1	WW and C2 domain containing 1	5	167718656	167899308	180653	0.5299
YTHDF1	YTH N6-methyladenosine RNA binding protein 1	20	61826781	61847586	20806	0.6180608
ZBTB46	zinc finger and BTB domain containing 46	20	62375019	62462597	87579	0.623586
ZFHX3	zinc finger homeobox 3	16	72816784	73093597	276814	0.5259785
ZFYVE27	zinc finger FYVE-type containing 27	10	99496878	99520664	23787	0.6028431
ZNF182	zinc finger protein 182	X	47974851	48003978	29128	0.58026
ZNF200	zinc finger protein 200	16	3222325	3236221	13897	0.551909
**ZNF512B**	zinc finger protein 512B	20	63956702	63969865	13164	0.623586
ZNF597	zinc finger protein 597	16	3432422	3443542	11121	0.551909
ZNF721	zinc finger protein 721	4	425815	499156	73342	0.540562

Supplementary Table 10. List of CNV-driven differentially expressed genes in SALS1 patients.

Genes with concordant changes in CNV and GE

Region	Cytoband	Size (bp)	Gene Symbol	Gene Name	Gain/Loss	Gene expression
chr1:17733251-17766250	1p36.13	31.81	RCC2*	regulator of chromosome condensation 2	Gain	Up
chr1:19197924-19229293	1p36.13	31.37	ALDH4A1*	aldehyde dehydrogenase 4 family, member A1	Gain	Up
chr1:19401000-19536812	1p36.13	135.75	UBR4*	ubiquitin protein ligase E3 component n-recognin 4	Gain	Up
chr1:208195587-208417665	1q32.2	222.08	PLXNA2	plexin A2	Gain	Up
chr1:2985565-3355185	1p36.32	369.44	PRDM16	PR domain containing 16	Gain	Up
chr2:127805599-127864903	2q14.3	59.31	BIN1*	bridging integrator 1	Gain	Up
chr2:20817564-20850867	2p24.1	33.30	HS1BP3	HCLS1 binding protein 3	Gain	Up
chr2:241653181-241759725	2q37.3	106.55	KIF1A	kinesin family member 1A	Gain	Up
chr4:41362802-41702061	4p13	161.87	LIMCH1	LIM and calponin homology domains 1	Gain	Up
chr6:152442819-152958534	6q25.1-q25.2	515.72	**SYNE1**	spectrin repeat containing, nuclear envelope 1	Gain	Up
chr6:31919864-31926864	6p21.33	7.00	NELFE	negative elongation factor complex member E	Gain	Up
chr6:32485151-32498006	6p21.32	12.85	HLA-DRB5	major histocompatibility complex, class II, DR beta 5	Gain	Up
chr8:144661867-144679845	8q24.3	17.98	**EEF1D**	eukaryotic translation elongation factor 1 delta (guanine nucleotide exchange protein)	Gain	Up
chr15:42646545-42704515	15q15.1	52.82	CAPN3	calpain 3, (p94)	Gain	Up
chr15:89631381-89745591	15q26.1	114.21	ABHD2	abhydrolase domain containing 2	Gain	Up
chr16:89984287-89987385	16q24.3	3.10	MC1R	melanocortin 1 receptor (alpha melanocyte stimulating hormone receptor)	Gain	Up
chr16:89988417-90002505	16q24.3	12.82	TUBB3	tubulin, beta 3 class III	Gain	Up
chr17:76849059-76921472	17q25.3	72.41	**TIMP2**	TIMP metallopeptidase inhibitor 2	Gain	Up
chr17:77906142-78009657	17q25.3	103.52	TBC1D16	TBC1 domain family, member 16	Gain	Up
chr17:78075339-78093680	17q25.3	18.33	GAA	glucosidase, alpha; acid	Gain	Up
chr20:23608534-23618685	20p11.21	4.28	**CST3**	cystatin C	Gain	Up
chrX:153287025-153363188	Xq28	75.93	MECP2	methyl CpG binding protein 2 (Rett syndrome)	Gain	Up
chrX:153672473-153679002	Xq28	6.53	FAM50A	family with sequence similarity 50, member A	Gain	Up
chrY:6778727-6959977	Yp11.2	181.00	TBL1Y	transducin (beta)-like 1, Y-linked	Gain	Up
chr1:207669473-207815110:	1q32.2	145.64	CR1*	complement component (3b/4b) receptor 1 (Knops blood group)	Loss	Down
chr1:47715811-47779819:	1p33	64.01	STIL	SCL/TAL1 interrupting locus	Loss	Down
chr2:32390910-32449181:	2p22.3	58.27	SLC30A6	solute carrier family 30 (zinc transporter), member 6	Loss	Down
chr4:2627159-2734302:	4p16.3	136.48	FAM193A*	family with sequence similarity 193, member A	Loss	Down
chr4:2939663-2965233:	4p16.3	25.46	NOP14*	NOP14 nucleolar protein	Loss	Down
chr4:84184972-84206067:	4q21.23	21.09	COQ2	coenzyme Q2 4-hydroxybenzoate polyprenyltransferase	Loss	Down
chr5:72861568-72878984:	5q13.2	16.20	UTP15	UTP15, U3 small nucleolar ribonucleoprotein, homolog (S. cerevisiae)	Loss	Down
chr10:76584685-76792380:	10q22.2	206.21	KAT6B	K(lysine) acetyltransferase 6B	Loss	Down
chr14:92524896-92572965:	14q32.12	48.07	ATXN3	ataxin 3	Loss	Down
chr15:41624892-41673248:	15q15.1	48.36	NUSAP1	nucleolar and spindle associated protein 1	Loss	Down
chr22:41253085-41328823:	22q13.2	75.74	XPNPEP3	X-prolyl aminopeptidase (aminopeptidase P) 3, putative	Loss	Down

Genes with discordant changes in CNV and GE

Region	Cytoband	Size (bp)	Gene Symbol	Gene Name	Gain/Loss	Gene expression
chr1:20915444-20945400	1p36.12	29.96	CDA*	Cytidine Deaminase	Gain	Down
chr1:3569129-3652765	1p36.32	83.64	TP73	Tumor Protein P73	Gain	Down
chr5:37812779-37835929	5p13.2	23.15	GDNF	Glial Cell Derived Neurotrophic Factor	Gain	Down
chr17:76000318-76104916	17q25.3	104.60	TNRC6C	Trinucleotide Repeat Containing 6C	Gain	Down
chr20:62031567-62033370	20q13.33	1.80	KCNQ2	Potassium Voltage-Gated Channel Subfamily Q Member 2	Gain	Down
chr1:10270606-10441664	1p36.22	170.90	KIF1B	kinesin family member 1B	Loss	Up
chr1:245912642-246670644	1q44	758.00	SMYD3	SET and MYND domain containing 3	Loss	Up
chr1:44173204-44396837	1p34.1	223.63	ST3GAL3	ST3 beta-galactoside alpha-2,3-sialyltransferase 3	Loss	Up
chr1:46654353-46685977	1p34.1	31.63	POMGNT1	protein O-linked mannose N-acetylglucosaminyltransferase 1 (beta 1,2-)	Loss	Up
chr2:61108709-61171410	2p16.1	41.43	REL	v-rel avian reticuloendotheliosis viral oncogene homolog	Loss	Up
chr4:1795039-1810599	4p16.3	15.56	FGFR3	fibroblast growth factor receptor 3	Loss	Up
chr4:3315874-3441640	4p16.3	125.77	RGS12*	regulator of G-protein signaling 12	Loss	Up
chr5:64064755-64314590	5q12.3	249.84	CWC27	CWC27 spliceosome-associated protein homolog (S. cerevisiae)	Loss	Up
chr6:31782952-31785719	6p21.33	2.43	HSPA1A*	heat shock 70kDa protein 1A	Loss	Up
chr6:31847536-31865464	6p21.33	17.93	EHMT2	euchromatic histone-lysine N-methyltransferase 2	Loss	Up
chr6:32485151-32498006	6p21.32	12.85	HLA-DRB5	major histocompatibility complex, class II, DR beta 5	Loss	Up
chr7:157129692-157210133	7q36.3	80.42	DNAJB6	DnaJ (Hsp40) homolog, subfamily B, member 6	Loss	Up
chr9:127997127-128003666	9q33.3	6.54	HSPA5	heat shock 70kDa protein 5 (glucose-regulated protein, 78kDa)	Loss	Up
chr9:135136827-135232113	9q34.13	93.55	**SETX**	senataxin	Loss	Up
chr10:75910943-76469061	10q22.2	558.12	**ADK**	adenosine kinase	Loss	Up
chr14:104394776-104519004	14q32.33	124.19	TDRD9	tudor domain containing 9	Loss	Up
chr14:73741858-73925288	14q24.2-q24.3	183.37	NUMB	numb homolog (Drosophila)	Loss	Up

chr14:92335755-92414046	14q32.12	78.29	FBLN5	fibulin 5	Loss	Up
chr15:41913432-42062141	15q15.1	109.53	MGA	MGA, MAX dimerization protein	Loss	Up
chr15:64680003-64747502	15q22.31	67.50	TRIP4	thyroid hormone receptor interactor 4	Loss	Up
chr15:93442286-93571237	15q26.1	127.69	CHD2	chromodomain helicase DNA binding protein 2	Loss	Up
chr16:46693589-46723144	16q11.2	29.56	VPS35	vacuolar protein sorting 35 homolog (S. cerevisiae)	Loss	Up
chr16:70514472-70557457	16q22.1	42.99	COG4*	component of oligomeric golgi complex 4	Loss	Up
chr17:15879874-15903006	17p12	23.13	**ZSWIM7**	zinc finger, SWIM-type containing 7	Loss	Up
chr17:16945790-17095962	17p11.2	27.99	MPRIP	myosin phosphatase Rho interacting protein	Loss	Up
chr17:30190190-30228729	17q11.2	38.54	UTP6	UTP6, small subunit (SSU) processome component, homolog (yeast)	Loss	Up
chr20:32581458-32670991	20q11.22	89.53	RALY	RALY heterogeneous nuclear ribonucleoprotein	Loss	Up
chr20:33543704-33590240	20q11.22	46.54	MYH7B	myosin, heavy chain 7B, cardiac muscle, beta	Loss	Up
chr20:34359923-34538292	20q11.22-q11.23	178.37	PHF20	PHD finger protein 20	Loss	Up
chr21:44473301-44496472	21q22.3	23.17	CBS*	cystathionine-beta-synthase	Loss	Up
chrX:41192561-41209527	Xp11.4	16.87	DDX3X	DEAD (Asp-Glu-Ala-Asp) box helicase 3, X-linked	Loss	Up

*CNV-encompassed genes selectively detected in SALS patients and not in control individuals.

Supplementary Table 11. List of CNV-driven differentially expressed genes in SALS2 patients.

Genes with concordant changes in CNV and GE

Region	Cytoband	Size (bp)	Gene Symbol	Gene Name	Gain/Loss	Gene expression
chr1:110276554-110283660	1p13.3	7.107	GSTM3*	glutathione S-transferase mu 3 (brain)	Gain	Up
chr1:110453233-110473616	1p13.3	20.384	CSF1*	colony stimulating factor 1 (macrophage)	Gain	Up
chr1:165370159-165414592	1q23.3	44.434	RXRG*	retinoid X receptor, gamma	Gain	Up
chr1:205012340-205047173	1q32.1	34.832	CNTN2	contactin 2 (axonal)	Gain	Up
chr1:21835475-21904905	1p36.12	69.048	ALPL*	alkaline phosphatase, liver/bone/kidney	Gain	Up
chr1:22004791-22109688	1p36.12	104.897	USP48*	ubiquitin specific peptidase 48	Gain	Up
chr1:22148737-22263750	1p36.12	115.014	HSPG2*	heparan sulfate proteoglycan 2	Gain	Up
chr1:2251719-2323190	1p36.33-p36.32	70.298	MORN1	MORN repeat containing 1	Gain	Up
chr1:3728645-3773797	1p36.32	45.153	CEP104	centrosomal protein 104kDa	Gain	Up
chr3:128198265-128212030	3q21.3	13.766	GATA2	GATA binding protein 2	Gain	Up
chr4:3505324-3534224	4p16.3	28.901	LRPAP1	low density lipoprotein receptor-related protein associated protein 1	Gain	Up
chr4:41746099-41750987	4p13	4.889	PHOX2B	paired-like homeobox 2b	Gain	Up
chr4:5053527-5502728	4p16.2	449.199	STK32B	serine/threonine kinase 32B	Gain	Up
chr4:5822491-5894785	4p16.2	72.295	CRMP1	collapsin response mediator protein 1	Gain	Up
chr4:843065-926174	4p16.3	83.11	GAK	cyclin G associated kinase	Gain	Up
chr4:8442532-8478282	4p16.1	38.729	TRMT44	tRNA methyltransferase 44 homolog (S. cerevisiae)	Gain	Up
chr5:121647455-121799794	5q23.2	151.975	SNCAIP	synuclein, alpha interacting protein	Gain	Up
chr5:169064251-169510386	5q35.1	446.136	DOCK2	dedicator of cytokinesis 2	Gain	Up
chr5:176560026-176727214	5q35.2-q35.3	166.382	NSD1	nuclear receptor binding SET domain protein 1	Gain	Up
chr5:37812779-37839782	5p13.2	23.151	GDNF	glial cell derived neurotrophic factor	Gain	Up
chr7:2767739-2883963	7p22.3-p22.2	116.219	GNA12	guanine nucleotide binding protein (G protein) alpha 12	Gain	Up
chr7:3341080-4308632	7p22.2	967.552	**SDK1**	sidekick cell adhesion molecule 1	Gain	Up
chr8:144635383-144645232	8q24.3	4.755	**GSDMD**	gasdermin D	Gain	Up
chr8:144661867-144679845	8q24.3	17.979	**EEF1D**	eukaryotic translation elongation factor 1 delta (guanine nucleotide exchange protein)	Gain	Up
chr8:144989321-145050913	8q24.3	35.724	PLEC	plectin	Gain	Up
chr9:116148592-116163618	9q32	15.027	**ALAD**	aminolevulinate dehydratase	Gain	Up
chr9:129089123-129269320	9q33.3	180.198	MVB12B	multivesicular body subunit 12B	Gain	Up
chr9:139776341-139821853	9q34.3	40.103	TRAF2*	TNF receptor-associated factor 2	Gain	Up
chr9:139901686-139923374	9q34.3	21.06	ABCA2*	ATP-binding cassette, sub-family A (ABC1), member 2	Gain	Up
chr9:139981379-140003639	9q34.3	22.261	MAN1B1*	mannosidase, alpha, class 1B, member 1	Gain	Up
chr10:94352825-94415152	10q23.33	62.328	KIF11	kinesin family member 11	Gain	Up
chr11:2965660-3013607	11p15.4	47.948	NAP1L4	nucleosome assembly protein 1-like 4	Gain	Up
chr11:46740743-46761056	11p11.2	20.314	F2	coagulation factor II (thrombin)	Gain	Up
chr11:5676740-5706339	11p15.4	21.915	TRIM5	tripartite motif containing 5	Gain	Up
chr12:57522282-57607142	12q13.3	84.844	LRP1	low density lipoprotein receptor-related protein 1	Gain	Up
chr14:105235686-105262080	14q32.33	26.394	**AKT1**	v-akt murine thymoma viral oncogene homolog 1	Gain	Up
chr14:99864083-99947228	14q32.2	83.144	SETD3	SET domain containing 3	Gain	Up
chr16:1413206-1464750	16p13.3	51.5	UNKL	unkempt homolog (Drosophila)-like	Gain	Up
chr16:2138709-2185899	16p13.3	47.189	PKD1	polycystic kidney disease 1 (autosomal dominant)	Gain	Up
chr16:3775055-3930121	16p13.3	155.066	CREBBP	CREB binding protein	Gain	Up
chr16:4012650-4166186	16p13.3	153.537	ADCY9	adenylate cyclase 9	Gain	Up
chr17:26721661-26733230	17q11.2	11.57	SLC46A1	solute carrier family 46 (folate transporter), member 1	Gain	Up
chr17:75277492-75496678	17q25.2-q25.3	219.187	SEPT9	septin 9	Gain	Up
chr19:1107633-1174282	19p13.3	66.65	SBNO2	strawberry notch homolog 2 (Drosophila)	Gain	Up
chr19:1205798-1228434	19p13.3	22.637	STK11	serine/threonine kinase 11	Gain	Up
chr20:1959402-1974931	20p13	14.993	PDYN	prodynorphin	Gain	Up
chr20:3869486-3904538	20p13	34.761	PANK2	pantothenate kinase 2	Gain	Up
chr20:61509090-61569304	20q13.33	48.814	DIDO1	death inducer-obliterator 1	Gain	Up
chr20:61974662-62009487	20q13.33	18.087	CHRNA4	cholinergic receptor, nicotinic, alpha 4 (neuronal)	Gain	Up
chr21:43919742-44001550	21q22.3	81.809	SLC37A1	solute carrier family 37 (glucose-6-phosphate transporter), member 1	Gain	Up
chr21:44473301-44496472	21q22.3	23.172	CBS*	cystathionine-beta-synthase	Gain	Up
chr21:46494493-46646478	21q22.3	151.986	ADARB1	adenosine deaminase, RNA-specific, B1	Gain	Up
chrX:153126969-153151628	Xq28	24.66	L1CAM	L1 cell adhesion molecule	Gain	Up
chr1:174128552-174964445	1q25.1	798.776	RABGAP1L*	RAB GTPase activating protein 1-like	Loss	Down
chr1:176826441-177134024	1q25.2	303.822	ASTN1	astrotactin 1	Loss	Down
chr1:178062864-178448649	1q25.2	385.785	RASAL2	RAS protein activator like 2	Loss	Down
chr1:179262849-179327815	1q25.2	64.966	SOAT1	sterol O-acyltransferase 1	Loss	Down
chr1:205681947-205719372	1q32.1	37.426	NUCKS1	nuclear casein kinase and cyclin-dependent kinase substrate 1	Loss	Down
chr1:207669473-207815110	1q32.2	145.638	CR1*	complement component (3b/4b) receptor 1 (Knops blood group)	Loss	Down
chr1:47023079-47069966	1p33	46.888	MKNK1	MAP kinase interacting serine/threonine kinase 1	Loss	Down
chr2:222282747-222437010	2q36.1	154.264	**EPHA4**	EPH receptor A4	Loss	Down
chr2:32288660-32382706	2p22.3	94.027	**SPAST**	spastin	Loss	Down
chr3:155838337-156256927	3q25.31	418.591	KCNAB1*	potassium voltage-gated channel, shaker-related subfamily, beta member 1	Loss	Down
chr3:156392205-156424559	3q25.31	30.105	TIPARP*	TCDD-inducible poly(ADP-ribose) polymerase	Loss	Down
chr4:2271324-2420370	4p16.3	149.047	ZFYVE28*	zinc finger, FYVE domain containing 28	Loss	Down
chr4:3076408-3245687	4p16.3	169.28	HTT	huntingtin	Loss	Down
chr4:84184972-84206067	4q21.23	21.091	COQ2	coenzyme Q2 4-hydroxybenzoate polyprenyltransferase	Loss	Down
chr5:70220768-70248839	5q13.2	27.184	**SMN1**	survival of motor neuron 1, telomeric	Loss	Down

chr6:74303102-74363737	6q13	60.636	SLC17A5*	solute carrier family 17 (acidic sugar transporter), member 5	Loss	Down
chr6:74405508-74538041	6q13	132.534	CD109	CD109 molecule	Loss	Down
chr6:76004607-76203516	6q14.1	185.697	FILIP1	filamin A interacting protein 1	Loss	Down
chr6:76458893-76629254	6q14.1	170.346	MYO6	myosin VI	Loss	Down
chr7:22766766-22771621	7p15.3	4.856	IL6	interleukin 6 (interferon, beta 2)	Loss	Down
chr7:22980878-23053814	7p15.3	72.893	FAM126A	family with sequence similarity 126, member A	Loss	Down
chr8:38088861-38120351	8p11.23	30.817	DDHD2	DDHD domain containing 2	Loss	Down
chr8:38127217-38239872	8p11.23	107.23	WHSC1L1	Wolf-Hirschhorn syndrome candidate 1-like 1	Loss	Down
chr9:127704887-127905838	9q33.3	200.951	SCAI	suppressor of cancer cell invasion	Loss	Down
chr9:127997127-128003666	9q33.3	6.54	HSPA5	heat shock 70kDa protein 5 (glucose-regulated protein, 78kDa)	Loss	Down
chr9:135136827-135232113	9q34.13	93.546	**SETX**	senataxin	Loss	Down
chr12:111890018-112037480	12q24.12	147.463	**ATXN2**	ataxin 2	Loss	Down
chr12:32552518-32798984	12p11.21	143.944	FGD4	FYVE, RhoGEF and PH domain containing 4	Loss	Down
chr12:50898768-51142450	12q13.12	243.683	DIP2B	DIP2 disco-interacting protein 2 homolog B (Drosophila)	Loss	Down
chr12:51373566-51422118	12q13.12	42.284	SLC11A2	solute carrier family 11 (proton-coupled divalent metal ion transporter), member 2	Loss	Down
chr12:51442082-51454207	12q13.12	12.126	LETMD1	LETM1 domain containing 1	Loss	Down
chr12:64173583-64203338	12q14.2	29.756	TMEM5	transmembrane protein 5	Loss	Down
chr12:64845840-64895899	12q14.2	50.06	**TBK1**	TANK-binding kinase 1	Loss	Down
chr14:50583846-50698596	14q21.3	114.254	SOS2	son of sevenless homolog 2 (Drosophila)	Loss	Down
chr14:92335755-92414046	14q32.12	78.292	FBLN5	fibulin 5	Loss	Down
chr15:41523341-41574085	15q15.1	50.647	CHP1	calcineurin-like EF-hand protein 1	Loss	Down
chr15:41679547-41694658	15q15.1	15.112	NDUFAF1	NADH dehydrogenase (ubiquinone) complex I, assembly factor 1	Loss	Down
chr15:44854894-44955876	15q21.1	100.983	**SPG11**	spastic paraplegia 11 (autosomal recessive)	Loss	Down
chr15:51200780-51298097	15q21.2	97.229	AP4E1	adaptor-related protein complex 4, epsilon 1 subunit	Loss	Down
chr15:65255363-65282251	15q22.31	26.889	SPG21	spastic paraplegia 21 (autosomal recessive, Mast syndrome)	Loss	Down
chr15:78832747-78841562	15q25.1	8.817	PSMA4	proteasome (prosome, macropain) subunit, alpha type, 4	Loss	Down
chr16:46693589-46723144	16q11.2	29.556	VPS35	vacuolar protein sorting 35 homolog (S. cerevisiae)	Loss	Down
chr17:19674143-19771239	17p11.2	97.097	ULK2	unc-51 like autophagy activating kinase 2	Loss	Down
chr17:2496923-2588909	17p13.3	91.987	PAFAH1B1	platelet-activating factor acetylhydrolase 1b, regulatory subunit 1 (45kDa)	Loss	Down
chr17:29109702-29151778	17q11.2	42.077	CRLF3	cytokine receptor-like factor 3	Loss	Down
chr17:29421945-29704695	17q11.2	282.751	NF1	neurofibromin 1	Loss	Down
chr17:30264044-30328057	17q11.2	64.014	SUZ12	SUZ12 polycomb repressive complex 2 subunit	Loss	Down
chr17:34136459-34174246	17q12	37.788	**TAF15**	TAF15 RNA polymerase II, TATA box binding protein (TBP)-associated factor, 68kDa	Loss	Down
chr17:3617919-3704537	17p13.2	86.619	ITGAE	integrin, alpha E (antigen CD103, human mucosal lymphocyte antigen 1; alpha polypeptide)	Loss	Down
chr18:9102628-9134343	18p11.22	31.716	NDUFV2	NADH dehydrogenase (ubiquinone) flavoprotein 2, 24kDa	Loss	Down
chr20:34256610-34287287	20q11.22	30.678	NFS1	NFS1 cysteine desulfurase	Loss	Down
chr20:34679426-34820721	20q11.23	78.06	EPB41L1	erythrocyte membrane protein band 4.1-like 1	Loss	Down
chr20:34995444-35157040	20q11.23	161.593	DLGAP4	discs, large (Drosophila) homolog-associated protein 4	Loss	Down
chr20:43595120-43708600	20q13.12	113.474	STK4	serine/threonine kinase 4	Loss	Down
chr20:44637547-44645200	20q13.12	7.654	**MMP9**	matrix metallopeptidase 9 (gelatinase B, 92kDa gelatinase, 92kDa type IV collagenase)	Loss	Down
chr22:32149937-32303020	22q12.2-q12-3	153.012	DEPDC5	DEP domain containing 5	Loss	Down
chr22:41253085-41328823	22q13.2	75.739	XPNPEP3	X-prolyl aminopeptidase (aminopeptidase P) 3, putative	Loss	Down
chr22:42229083-42303312	22q13.2	73.27	SREBF2	sterol regulatory element binding transcription factor 2	Loss	Down
chrX:119658444-119709684	Xq24	51.239	CUL4B*	cullin 4B	Loss	Down

Genes with discordant changes in CNV and GE

Region	Cytoband	Size (bp)	Gene Symbol	Gene Name	Gain/Loss	Gene expression
chr1:1981909-2116834	1p36.33	134.93	**PRKCZ**	protein kinase C, zeta	Gain	Down
chr1:2323214-2336883	1p36.32	13.672	RER1	RER1 retention in endoplasmic reticulum 1 homolog (S. cerevisiae)	Gain	Down
chr1:3728645-3773797	1p36.32	45.153	CEP104	centrosomal protein 104kDa	Gain	Down
chr1:5922870-6052618	1p36.31	129.664	NPHP4	nephronophthisis 4	Gain	Down
chr1:6526152-6580121	1p36.31	19.378	**PLEKHG5**	pleckstrin homology domain containing, family G (with RhoGef domain) member 5	Gain	Down
chr1:16174359-16266951	1p36.21-p36.13	92.592	SPEN	spen homolog, transcriptional regulator (Drosophila)	Gain	Down
chr1:17733251-17766250	1p36.13	31.809	RCC2*	regulator of chromosome condensation 2	Gain	Down
chr1:18434240-18704977	1p36.13	270.738	IGSF21*	immunoglobin superfamily, member 21	Gain	Down
chr1:20959948-20978004	1p36.12	18.057	PINK1*	PTEN induced putative kinase 1	Gain	Down
chr1:20978260-20988037	1p36.12	9.778	DDOST*	dolichyl-diphosphooligosaccharide--protein glycosyltransferase subunit (non-catalytic)	Gain	Down
chr1:21132785-21503381	1p36.12	370.597	EIF4G3*	eukaryotic translation initiation factor 4 gamma, 3	Gain	Down
chr1:22004791-22109688	1p36.12	104.897	USP48*	ubiquitin specific peptidase 48	Gain	Down
chr1:55315300-55352921	1p32.3	37.622	DHCR24	24-dehydrocholesterol reductase	Gain	Down
chr1:110276554-110283660	1p13.3	7.107	GSTM3*	glutathione S-transferase mu 3 (brain)	Gain	Down
chr1:156776035-156786640	1q23.1	10.606	SH2D2A	SH2 domain containing 2A	Gain	Down
chr1:205012340-205047173	1q32.1	34.832	CNTN2	contactin 2 (axonal)	Gain	Down
chr1:226819391-226927028	1q42.12	107.486	ITPKB	inositol-trisphosphate 3-kinase B	Gain	Down

chr1:227057885-227083804	1q42.13	25.532	PSEN2	presenilin 2 (Alzheimer disease 4)	Gain	Down
chr2:74588281-74619214	2p13.1	19.202	**DCTN1**	dynactin 1	Gain	Down
chr2:127805599-127864903	2q14.3	59.305	BIN1*	bridging integrator 1	Gain	Down
chr2:171673200-171717661	2q31.1	44.46	GAD1	glutamate decarboxylase 1 (brain, 67kDa)	Gain	Down
chr2:241653181-241759725	2q37.3	106.545	KIF1A	kinesin family member 1A	Gain	Down
chr4:433773-492960	4p16.3	59.188	ZNF721	zinc finger protein 721	Gain	Down
chr4:699537-764428	4p16.3	64.855	PCGF3	polycomb group ring finger 3	Gain	Down
chr4:3505324-3534224	4p16.3	28.901	LRPAP1	low density lipoprotein receptor-related protein associated protein 1	Gain	Down
chr4:5822491-5894785	4p16.2	72.295	CRMP1	collapsin response mediator protein 1	Gain	Down
chr4:6322305-6565327	4p16.1	61.293	PPP2R2C	protein phosphatase 2, regulatory subunit B, gamma	Gain	Down
chr4:6784459-6885899	4p16.1	101.441	KIAA0232	KIAA0232	Gain	Down
chr4:7967037-8160559	4p16.1	193.523	ABLIM2	actin binding LIM protein family, member 2	Gain	Down
chr4:8442532-8478282	4p16.1	38.729	TRMT44	tRNA methyltransferase 44 homolog (S. cerevisiae)	Gain	Down
chr4:40812044-41218731	4p14-p13	404.592	APBB2	amyloid beta (A4) precursor protein-binding, family B, member 2	Gain	Down
chr4:41258898-41270446	4p13	11.549	UCHL1	ubiquitin carboxyl-terminal esterase L1 (ubiquitin thiolesterase)	Gain	Down
chr4:41362802-41702061	4p13	161.871	LIMCH1	LIM and calponin homology domains 1	Gain	Down
chr4:101944587-102268655	4q24	324.042	**PPP3CA**	protein phosphatase 3, catalytic subunit, alpha isozyme	Gain	Down
chr4:102711764-102995969	4q24	284.206	BANK1	B-cell scaffold protein with ankyrin repeats 1	Gain	Down
chr4:103422486-103538459	4q24	115.974	NFKB1	nuclear factor of kappa light polypeptide gene enhancer in B-cells 1	Gain	Down
chr5:137774690-137782658	5q31.2	7.969	REEP2	receptor accessory protein 2	Gain	Down
chr5:167719065-167899308	5q34	180.244	WWC1	WW and C2 domain containing 1	Gain	Down
chr5:168088738-168728133	5q34-q35.1	639.396	SLIT3	slit homolog 3 (Drosophila)	Gain	Down
chr5:171288553-171433877	5q35.1	145.322	FBXW11	F-box and WD repeat domain containing 11	Gain	Down
chr5:173315331-173387313	5q35.2	71.983	CPEB4	cytoplasmic polyadenylation element binding protein 4	Gain	Down
chr5:175773065-175788809	5q35.2	15.744	KIAA1191	KIAA1191	Gain	Down
chr5:176047210-176057557	5q35.2	10.348	SNCB	synuclein, beta	Gain	Down
chr5:176332006-176433795	5q35.2	101.79	UIMC1	ubiquitin interaction motif containing 1	Gain	Down
chr5:176560026-176727214	5q35.2-q35.3	166.382	NSD1	nuclear receptor binding SET domain protein 1	Gain	Down
chr5:176853687-176869850	5q35.3	16.164	GRK6	G protein-coupled receptor kinase 6	Gain	Down
chr6:31919864-31926864	6p21.33	7.001	NELFE	negative elongation factor complex member E	Gain	Down
chr6:32148745-32152099	6p21.32	3.355	AGER	advanced glycosylation end product-specific receptor	Gain	Down
chr6:32158543-32163300	6p21.32	4.758	GPSM3	G-protein signaling modulator 3	Gain	Down
chr6:32162620-32191844	6p21.32	29.225	NOTCH4	notch 4	Gain	Down
chr7:2281857-2290780	7p22.3	8.924	NUDT1	nudix (nucleoside diphosphate linked moiety X)-type motif 1	Gain	Down
chr8:42032236-42065242	8p11.21	32.959	PLAT	plasminogen activator, tissue	Gain	Down
chr8:144661867-144679845	8q24.3	17.979	**EEF1D**	eukaryotic translation elongation factor 1 delta (guanine nucleotide exchange protein)	Gain	Down
chr9:35056065-35072739	9p13.3	16.675	**VCP**	valosin containing protein	Gain	Down
chr9:87283417-87641985	9p21.33	355.04	NTRK2	neurotrophic tyrosine kinase, receptor, type 2	Gain	Down
chr9:137218316-137332431	9q34.2	89.297	RXRA*	retinoid X receptor, alpha	Gain	Down
chr9:139756566-139760788	9q34.3	4.168	EDF1*	endothelial differentiation-related factor 1	Gain	Down
chr9:140033609-140063214	9q34.3	29.606	GRIN1*	glutamate receptor, ionotropic, N-methyl D-aspartate 1	Gain	Down
chr10:99496878-99520664	10q24.2	23.787	ZFYVE27	zinc finger, FYVE domain containing 27	Gain	Down
chr11:637305-640706	11p15.5	3.401	**DRD4**	dopamine receptor D4	Gain	Down
chr11:644225-695740	11p15.5	51.516	**DEAF1**	DEAF1 transcription factor	Gain	Down
chr11:790475-798269	11p15.5	7.795	SLC25A22	solute carrier family 25 (mitochondrial carrier: glutamate), member 22	Gain	Down
chr11:925809-1012245	11p15.5	86.437	AP2A2	adaptor-related protein complex 2, alpha 2 subunit	Gain	Down
chr11:2150346-2179611	11p15.5	12	IGF2	insulin-like growth factor 2 (somatomedin A)	Gain	Down
chr11:2185159-2193107	11p15.5	7.877	TH	tyrosine hydroxylase	Gain	Down
chr11:3876933-4114440	11p15.4	237.508	STIM1	stromal interaction molecule 1	Gain	Down
chr11:5246696-5248301	11p15.4	1.606	HBB	hemoglobin, beta	Gain	Down
chr11:5676740-5706339	11p15.4	21.915	TRIM5	tripartite motif containing 5	Gain	Down
chr11:6621144-6624884	11p15.4	3.737	RRP8	ribosomal RNA processing 8, methyltransferase, homolog (yeast)	Gain	Down
chr11:6633997-6640692	11p15.4	6.696	TPP1	tripeptidyl peptidase I	Gain	Down
chr11:17414432-17498449	11p15.1	84.018	ABCC8	ATP-binding cassette, sub-family C (CFTR/MRP), member 8	Gain	Down
chr11:45825623-45834568	11p11.2	8.945	SLC35C1	solute carrier family 35 (GDP-fucose transporter), member C1	Gain	Down
chr11:45868669-45904799	11p11.2	35.843	CRY2	cryptochrome 2 (photolyase-like)	Gain	Down
chr12:125262174-125348519	12q24.31	86.346	SCARB1	scavenger receptor class B, member 1	Gain	Down
chr13:98794893-99102023	13q32.2	306.59	FARP1	FERM, RhoGEF (ARHGEF) and pleckstrin domain protein 1 (chondrocyte-derived)	Gain	Down
chr14:99635624-99738514	13q32.2	102.198	**BCL11B**	B-cell CLL/lymphoma 11B (zinc finger protein)	Gain	Down
chr15:42646545-42704515	15q15.1	52.818	CAPN3	calpain 3, (p94)	Gain	Down
chr15:51200780-51298097	15q21.2	97.229	AP4E1	adaptor-related protein complex 4, epsilon 1 subunit	Gain	Down
chr15:89346674-89418585	15q26.1	71.912	ACAN	aggrecan	Gain	Down
chr15:89785634-89860362	15q26.1	73.169	FANCI	Fanconi anemia, complementation group I	Gain	Down
chr16:1357420-1377019	16p13.3	17.866	**UBE2I**	ubiquitin-conjugating enzyme E2I	Gain	Down
chr16:1728201-1752073	16p13.3	23.796	HN1L	hematological and neurological expressed 1-like	Gain	Down
chr16:2525146-2555734	16p13.3	30.588	TBC1D24	TBC1 domain family, member 24	Gain	Down
chr16:3292028-3306648	16p13.3	14.6	MEFV	Mediterranean fever	Gain	Down
chr16:4524719-4560348	16p13.3	34.008	HMOX2	heme oxygenase (decycling) 2	Gain	Down

chr16:4853204-4897383	16p13.3	44.1	GLYR1	glyoxylate reductase 1 homolog (Arabidopsis)	Gain	Down
chr16:5121810-5137380	16p13.3	15.571	ALG1	ALG1, chitobiosyldiphosphodolichol beta-mannosyltransferase	Gain	Down
chr16:5289469-7763342	16p13.3	380.59	RBFOX1	RNA binding protein, fox-1 homolog (C. elegans) 1	Gain	Down
chr16:9847265-10276611	16p13.2	428.999	GRIN2A	glutamate receptor, ionotropic, N-methyl D-aspartate 2A	Gain	Down
chr16:28488600-28505897	16p11.2	15.024	CLN3	ceroid-lipofuscinosis, neuronal 3	Gain	Down
chr16:74481325-74641042	16q23.1	159.717	GLG1	golgi glycoprotein 1	Gain	Down
chr16:74746853-74808729	16q23.1	61.874	FA2H	fatty acid 2-hydroxylase	Gain	Down
chr17:26083792-26127555	17q11.2	43.764	NOS2	nitric oxide synthase 2, inducible	Gain	Down
chr17:26646121-26655711	17q11.2	9.591	TMEM97	transmembrane protein 97	Gain	Down
chr17:26721661-26733230	17q11.2	11.57	SLC46A1	solute carrier family 46 (folate transporter), member 1	Gain	Down
chr17:75277492-75496678	17q25.2-q25.3	219.187	SEPT9	septin 9	Gain	Down
chr17:76000194-76104916	17q25.3	104.599	TNRC6C	trinucleotide repeat containing 6C	Gain	Down
chr17:76670130-76778376	17q25.3	108.247	CYTH1	cytohesin 1	Gain	Down
chr19:1269267-1274809	19p13.3	3.905	CIRBP	cold inducible RNA binding protein	Gain	Down
chr19:1383526-1395588	19p13.3	11.882	NDUFS7	NADH dehydrogenase (ubiquinone) Fe-S protein 7, 20kDa (NADH-coenzyme Q reductase)	Gain	Down
chr19:1397025-1401569	19p13.3	3.76	GAMT	guanidinoacetate N-methyltransferase	Gain	Down
chr19:50706885-50813801	19q13.33	106.917	MYH14	myosin, heavy chain 14, non-muscle	Gain	Down
chr19:50818765-50832634	19q13.33	13.87	KCNC3	potassium voltage-gated channel, Shaw-related subfamily, member 3	Gain	Down
chr19:50879680-50886285	19q13.33	6.794	NR1H2	nuclear receptor subfamily 1, group H, member 2	Gain	Down
chr20:2633178-2639039	20p13	5.862	NOP56	NOP56 ribonucleoprotein	Gain	Down
chr20:61509090-61569304	20q13.33	48.814	DIDO1	death inducer-obliterator 1	Gain	Down
chr20:61826781-61847538	20q13.33	20.757	YTHDF1	YTH domain family, member 1	Gain	Down
chr20:62031561-62103993	20q13.33	1.804	KCNQ2	potassium voltage-gated channel, KQT-like subfamily, member 2	Gain	Down
chr20:62375021-62463731	20q13.33	61.836	ZBTB46	zinc finger and BTB domain containing 46	Gain	Down
chr20:62588057-62601223	20q13.33	13.167	**ZNF512B**	zinc finger protein 512B	Gain	Down
chr21:44473301-44496472	21q22.3	23.172	CBS*	cystathionine-beta-synthase	Gain	Down
chr21:45138978-45182188	21q22.3	43.211	PDXK	pyridoxal (pyridoxine, vitamin B6) kinase	Gain	Down
chr21:45193546-45196256	21q22.3	2.711	CSTB	cystatin B (stefin B)	Gain	Down
chr21:46269500-46294487	21q22.3	24.319	PTTG1IP	pituitary tumor-transforming 1 interacting protein	Gain	Down
chr22:24376135-24384284	22q21.23	8.146	GSTT1	glutathione S-transferase theta 1	Gain	Down
chr22:24407642-24574596	22q11.23	166.832	CABIN1	calcineurin binding protein 1	Gain	Down
chr22:24666785-24813708	22q11.23	146.919	SPECC1L	sperm antigen with calponin homology and coiled-coil domains 1-like	Gain	Down
chr22:37621301-37640339	22q13.1	18.996	RAC2	ras-related C3 botulinum toxin substrate 2 (rho family, small GTP binding protein Rac2)	Gain	Down
chr22:37764000-37823505	22q13.1	59.506	ELFN2	extracellular leucine-rich repeat and fibronectin type III domain containing 2	Gain	Down
chr22:38349674-38368463	22q13.1	15.646	POLR2F	polymerase (RNA) II (DNA directed) polypeptide F	Gain	Down
chr22:38507502-38577857	22q13.1	70.26	PLA2G6	phospholipase A2, group VI (cytosolic, calcium-independent)	Gain	Down
chr22:42481530-42486888	22q13.2	5.359	NDUFA6	NADH dehydrogenase (ubiquinone) 1 alpha subcomplex, 6, 14kDa	Gain	Down
chrX:47431300-47479256	Xp11.23	47.957	SYN1	synapsin I	Gain	Down
chrX:48367347-48379202	Xp11.23	11.031	PORCN	porcupine homolog (Drosophila)	Gain	Down
chrX:48455880-48463582	Xp11.23	7.703	WDR13	WD repeat domain 13	Gain	Down
chrX:48910961-48927510	Xp11.23	11.014	CCDC120	coiled-coil domain containing 120	Gain	Down
chrX:49126305-49144557	Xp11.23	18.25	PPP1R3F	protein phosphatase 1, regulatory subunit 3F	Gain	Down
chrX:53262058-53350522	Xp11.22	88.465	IQSEC2	IQ motif and Sec7 domain 2	Gain	Down
chrX:122317996-122624766	Xq25	306.671	GRIA3	glutamate receptor, ionotropic, AMPA 3	Gain	Down
chrX:153287025-153363188	Xq28	75.925	MECP2	methyl CpG binding protein 2 (Rett syndrome)	Gain	Down
chrY:16634488-16956825	Yq11.221	320.223	NLGN4Y	neuroligin 4, Y-linked	Gain	Down
chrY:21212994-21239302	Yq11.222	144.718	TTTY14	testis-specific transcript, Y-linked 14 (non-protein coding)	Gain	Down
chrY:21867301-21906825	Yq11.222	39.525	KDM5D	lysine (K)-specific demethylase 5D	Gain	Down
chr1:175291935-175712752	1q25.1	420.818	TNR	tenascin R	Loss	Up
chr1:176432307-176814737	1q25.2	379.664	PAPPA2	pappalysin 2	Loss	Up
chr2:32581735-32843966	2p22.3	261.87	BIRC6	baculoviral IAP repeat containing 6	Loss	Up
chr4:1795039-1810599	4p16.3	15.561	FGFR3*	fibroblast growth factor receptor 3	Loss	Up
chr6:76311596-76427997	6q14.1	116.373	SENP6	SUMO1/sentrin specific peptidase 6	Loss	Up
chr8:38127217-38239872	8p11.23	107.23	WHSC1L1	Wolf-Hirschhorn syndrome candidate 1-like 1	Loss	Up
chr8:38268656-38326352	8p11.23-p11.22	56.708	FGFR1	fibroblast growth factor receptor 1	Loss	Up
chr8:38854505-38962780	8p11.22	108.275	ADAM9	ADAM metallopeptidase domain 9	Loss	Up
chr9:135136827-135232113	9q34.13	93.546	**SETX**	senataxin	Loss	Up
chr10:76584685-76792380	10q22.2	206.21	KAT6B	K(lysine) acetyltransferase 6B	Loss	Up
chr10:94352825-94415152	10q23.33	62.328	KIF11	kinesin family member 11	Loss	Up
chr11:108093559-108239829	11q22.3	146.268	ATM*	ataxia telangiectasia mutated	Loss	Up
chr14:92524896-92572965	14q32.12	48.07	ATXN3	ataxin 3	Loss	Up
chr15:64199235-64363614	15q22.31	139.287	DAPK2*	death-associated protein kinase 2	Loss	Up
chr15:78885394-78913637	15q25.1	25.991	CHRNA3	cholinergic receptor, nicotinic, alpha 3 (neuronal)	Loss	Up
chr17:2659050-2941035	17p13.3	241.304	RAP1GAP2	RAP1 GTPase activating protein 2	Loss	Up
chr17:29421945-29704695	17q11.2	282.751	NF1	neurofibromin 1	Loss	Up
chr19:37638340-37663643	19q13.12	22.615	ZNF585A	zinc finger protein 585A	Loss	Up
chr20:34359923-34538292	20q11.22-q11.23	178.366	PHF20	PHD finger protein 20	Loss	Up
chr20:42984441-43061485	20q13.12	51.675	HNF4A	hepatocyte nuclear factor 4, alpha	Loss	Up

*CNV-encompassed genes selectively detected in SALS patients and not in control individuals.

Supplementary Table 12. The top 15 functional enrichment of CNV-associated DEGs in SALS.

SALS1			
GO Term	GO name	Ratio	p-value
Biological processes			
GO:0051129	negative regulation of cellular component organization	8/474	0.0002
GO:0071103	DNA conformation change	4/99	0.0004
GO:1901214	regulation of neuron death	5/225	0.0012
GO:0051346	negative regulation of hydrolase activity	6/354	0.0015
GO:0009123	nucleoside monophosphate metabolic process	6/427	0.0038
GO:0051962	positive regulation of nervous system development	5/313	0.0048
GO:0016568	chromatin modification	6/475	0.0063
GO:0010564	regulation of cell cycle process	6/481	0.0067
GO:0006457	protein folding	4/221	0.0075
GO:0010720	positive regulation of cell development	5/358	0.0084
GO:0007411	axon guidance	5/367	0.0093
GO:0097485	neuron projection guidance	5/367	0.0093
GO:0052547	regulation of peptidase activity	5/372	0.0098
GO:0046034	ATP metabolic process	5/377	0.0103
GO:0006281	DNA repair	5/403	0.0135
Molecular functions			
GO:0044389	small conjugating protein ligase binding	5/182	0.0004
GO:0031625	ubiquitin protein ligase binding	5/182	0.0004
GO:0004386	helicase activity	4/150	0.0019
GO:0016887	ATPase activity	6/395	0.0026
GO:0008017	microtubule binding	4/171	0.0031
GO:0051082	unfolded protein binding	3/101	0.0054
GO:0015631	tubulin binding	4/241	0.0101
GO:0003774	motor activity	3/132	0.0112
GO:0005524	ATP binding	11/1494	0.0142
GO:0050839	cell adhesion molecule binding	3/168	0.0211
Cellular components			
GO:0044449	contractile fiber part	4/167	0.0028
GO:0005925	focal adhesion	5/352	0.0078
GO:0005874	microtubule	5/360	0.0086
GO:0044420	extracellular matrix part	3/127	0.0100
GO:0031965	nuclear membrane	3/149	0.0154
GO:0005694	chromosome	3/166	0.0205
GO:0005730	nucleolus	11/1653	0.0279
GO:0005774	vacuolar membrane	3/208	0.0365
GO:0031301	integral component of organelle membrane	3/211	0.0378
GO:0005765	lysosomal membrane	3/196	0.0314

SALS2			
GO Term	GO name	Ratio	p-value
Biological processes			
GO:0050804	regulation of synaptic transmission	21/245	3.45E-12
GO:0007611	learning or memory	19/198	5.26503E-12
GO:1901214	regulation of neuron death	18/225	3.94422E-10
GO:0007626	locomotory behavior	16/183	1.04645E-09
GO:0048167	regulation of synaptic plasticity	12/120	2.99133E-08
GO:0048710	regulation of astrocyte differentiation	6/24	3.64235E-07
GO:0060078	regulation of postsynaptic membrane potential	8/58	5.27232E-07
GO:0050905	neuromuscular process	9/79	5.41697E-07
GO:0030036	actin cytoskeleton organization	15/253	6.0561E-07
GO:0051588	regulation of neurotransmitter transport	8/63	1.01036E-06
GO:0030029	actin filament-based process	16/303	1.18514E-06
GO:0036293	response to decreased oxygen levels	14/245	2.23185E-06
GO:0032368	regulation of lipid transport	8/83	8.32898E-06
GO:0033674	positive regulation of kinase activity	19/480	8.39154E-06
GO:0045664	regulation of neuron differentiation	17/405	1.19437E-05
Molecular functions			
GO:0016597	amino acid binding	9/107	7.00126E-06
GO:0008134	transcription factor binding	17/464	6.6232E-05
GO:0005516	calmodulin binding	9/170	0.000263527
GO:0005231	excitatory extracellular ligand-gated ion channel activity	5/48	0.000300649
GO:0005524	ATP binding	34/1494	0.000371211
GO:0048037	cofactor binding	11/266	0.000478237
GO:0015459	potassium channel regulator activity	4/40	0.001441792
GO:0008270	zinc ion binding	28/1256	0.001704809
GO:0002020	protease binding	5/77	0.002600257
GO:0043130	ubiquitin binding	5/77	0.002600257
Cellular components			
GO:0005829	cytosol	70/2529	2.48352E-11
GO:0030425	dendrite	17/236	6.04827E-09
GO:0044456	synapse part	19/395	4.83099E-07
GO:0009986	cell surface	20/437	5.24901E-07
GO:0043025	neuronal cell body	15/270	1.37172E-06
GO:0030424	axon	11/161	5.12808E-06
GO:0030426	growth cone	8/99	3.05361E-05
GO:0043235	receptor complex	13/272	3.44742E-05
GO:0005774	vacuolar membrane	11/208	5.60909E-05
GO:0005654	nucleoplasm	26/1051	0.000569112

Supplementary Table 13. The signal pathway enrichment of CNV-associated DEGs

#	Maps	min(pValue)	min(FDR)	-log(pValue)	SALS1/CTRL+				SALS2_CTRL			
					Ratio	p-value	FDR	-log(pValue)	Ratio	p-value	FDR	-log(pValue)
1	Ras signaling pathway	0.000001	0.000314	5.8570	2/227	0.188667	0.355397	0.7243	14/227	0.000001	0.000194	6.0436
2	Huntington's disease signaling	0.000002	0.000147	5.6658	9/124	0.000023	0.000751	4.6360	11/124	0.000002	0.000147	5.6658
3	Axon guidance	0.000002	0.000682	5.6223	5/515	0.034246	0.268766	1.4654	21/515	0.000002	0.000719	5.7597
4	MAPK signaling pathway	0.000026	0.001940	4.5891	2/255	0.224438	0.363538	0.6489	13/255	0.000018	0.001252	4.7555
5	mTOR signaling pathway	0.000103	0.005779	3.9857	1/68	0.303613	0.333815	0.5177	7/68	0.000103	0.005779	3.9857
6	T cell activation	0.000184	0.009671	3.7347	5/101	0.000184	0.009671	3.7347	6/101	0.005663	0.047492	2.2469
7	HMGB1/RAGE signaling pathway	0.000193	0.008624	3.7149	1/53	0.245588	0.316988	0.6098	6/53	0.000193	0.008624	3.7149
8	p53 pathway	0.000605	0.008650	3.2179	2/71	0.025693	0.081846	1.5902	5/71	0.001818	0.019700	2.7403
9	Glucocorticoid receptor signaling	0.000624	0.014958	3.2047	1/25	0.124312	0.316988	0.9055	4/25	0.000624	0.014958	3.2047
10	FGF signaling pathway	0.000636	0.008650	3.1965	1/99	0.293875	0.347679	0.5318	7/99	0.000223	0.003618	3.6524
11	TNF signaling pathway	0.000810	0.017543	3.0913	1/46	0.216912	0.316988	0.6637	5/46	0.000810	0.017543	3.0913
12	Apoptosis and survival signaling	0.003908	0.029524	2.4081	3/102	0.005505	0.081846	2.2593	4/102	0.037272	0.093179	1.4286
13	Notch signaling pathway	0.005292	0.041244	2.2763	1/48	0.155063	0.352632	0.8095	3/48	0.021175	0.085499	1.6742
14	B cell receptor signaling pathway	0.007520	0.108525	2.1238	3/192	0.029819	0.268766	1.5255	7/192	0.009809	0.088754	2.0084
15	Extracellular matrix organization	0.009173	0.108525	2.0375	3/283	0.076919	0.268766	1.1140	9/283	0.008598	0.087814	2.0656
16	VEGF signaling pathway	0.009334	0.108525	2.0299	2/328	0.319027	0.385167	0.4962	11/328	0.002573	0.051877	2.5895
17	PI3K-Akt signaling pathway	0.012219	0.068626	1.9130	1/341	0.700575	0.709332	0.1545	11/341	0.003459	0.026436	2.4611
18	Protein folding and processing	0.012450	0.068626	1.9048	3/169	0.021470	0.286433	1.6682	6/169	0.018361	0.075561	1.7361
19	Regulation of actin cytoskeleton	0.013858	0.073399	1.8583	1/214	0.529681	0.564529	0.2760	8/214	0.005117	0.032209	2.2910
20	Interleukin signaling pathway	0.030398	0.177505	1.5172	2/392	0.399958	0.448255	0.3980	11/392	0.009529	0.088754	2.0209

Supplementary Table 14. Cluster random assignment of SALS patients.

Patient code	Unsupervised Clustering	Randomized clustering assignement
1	Control	Control
2	Control	Control
3	Control	Control
4	Control	Control
5	Control	Control
6	Control	Control
7	Control	Control
8	Control	Control
9	Control	Control
10	Control	Control
11	ALS 1	Group 1
12	ALS 1	Group 1
13	ALS 1	Group 2
14	ALS 1	Group 2
15	ALS 2	Group 2
16	ALS 1	Group 2
17	ALS 1	Group 2
18	ALS 1	Group 2
19	ALS 1	Group 2
20	ALS 1	Group 2
21	ALS 1	Group 1
22	ALS 1	Group 1
23	ALS 1	Group 1
24	ALS 1	Group 2
25	ALS 1	Group 2
26	ALS 1	Group 2
27	ALS 1	Group 1
29	ALS 2	Group 1
30	ALS 2	Group 1
31	ALS 2	Group 2
32	ALS 2	Group 2
33	ALS 2	Group 2
34	ALS 2	Group 2
35	ALS 2	Group 1
36	ALS 2	Group 1
37	ALS 2	Group 1
38	ALS 2	Group 1
39	ALS 2	Group 1
40	ALS 2	Group 1
41	ALS 2	Group 1
